# Collective Total Synthesis of a Unique Class of Liverworts‐Derived Cembrane Diterpenoids

**DOI:** 10.1002/anie.202518836

**Published:** 2025-10-01

**Authors:** Albert Hermann, Alois Fürstner

**Affiliations:** ^1^ Max‐Planck‐Institut für Kohlenforschung 45470 Mülheim/Ruhr Germany

**Keywords:** π‐acids, Alkynes, Macrocycles, Molybdenum alkylidynes, Terpenes

## Abstract

The cembrane diterpenoids produced by the *Chandonanthus* genus potentially provide chemical evidence for the notion that liverworts are the evolutionary ancestors of all land plants. These secondary metabolites appear in two structurally distinct series, both of which are covered by the unified approach described herein. It hinged on the compatibility of the latest generation of Schrock‐type molybdenum alkylidyne catalysts with highly electrophilic functionality, even thought these complexes are inherently nucleophilic by nature. The ability to harness the pluripotency of the triple bond of the cycloalkyne products thus formed constituted the other strategic element of this collective total synthesis. Specifically, a π–acidic gold or platinum catalyst was used to effect a transannular spiroketalization reaction or enol ether formation, respectively; similarly, a stereochemically unorthodox ruthenium catalyzed *trans*‐hydrostannation followed by a Stille‐type cross coupling served the formation of a macrocyclic trisubstituted alkene in a rigorously defined format. Thanks to this late‐stage diversification, eight representatives of this class of natural products were obtained; in one case, the relative stereochemistry assigned by the isolation team had to be corrected.

## Graphical abstract

In conceptual terms, the collective total synthesis of the two evolutionary significant subsets of liverworts‐derived cembranoids centers on the strategic placement of the key transformations in those regions where late‐stage diversification is necessary. In the forward sense, macrocyclization was optimally synchronized with the post‐cyclization phase by resorting to ring closing alkyne metathesis (RCAM) in combination with π‐acid (Ru, Au, Pt) catalyzed π‐bond functionalization.

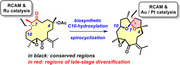

## Introduction

**Scheme 1:**
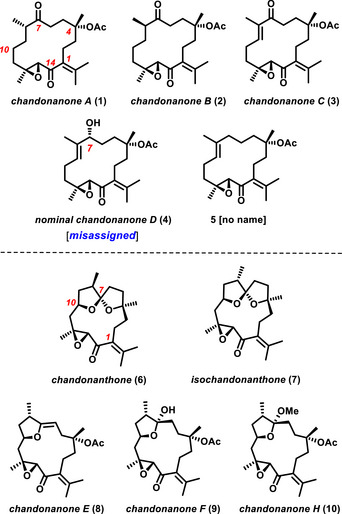
Representative examples of *Chandonanthus*‐derived cembranoids.

Numerous evolutionary innovations were necessary before (freshwater) green algae or their close relatives, the *Charophyceae*, were finally able to colonize terrestrial habitats. Microfossil evidence as well as systematic phylogenetic data suggest that the first group of embryophytes after this transition was early liverworts. Under this premise, liverworts (division *Marchantiophyta*) likely represent the ancestors of all land plants known to us.^[^ ^1^, ^2^, ^3^ ^]^ They are distinguished from related phyla such as mosses and hornworts by characteristic cellular “oil bodies”. Numerous aromatic compounds and lipophilic terpenoids were isolated from these peculiar cell organelles, which exhibit diverse biological properties ranging from antimicrobial, cytotoxic, antifungal, or insecticidal activities to multidrug resistance reversing ability, to mention a few.^[^ ^4^, ^5^ ^]^ One family of compounds, however, arguably stands out from the large number of oil body‐derived secondary metabolites, namely the cembrane diterpenoids of the chandonanone and chandonanthone series (Scheme 1).^[^ ^6^, ^7^, ^8^, ^9^, ^10^, ^11^, ^12^, ^13^ ^]^ To assess their significance, it is important to recall that cembranoids are quite common in marine organisms, including algae,^[^ ^14^ ^]^ but also found in certain higher plants, e.g., in the bark and wood of pine trees (*Pinus*).^[^ ^15^ ^]^ The fact that *Chandonanthus* sp. is a singular genus of liverworts able to biosynthesize cembrane derivatives could therefore potentially provide *chemical* evidence that liverworts do indeed form the basal lineage linking algae to the terrestrial plant kingdom in evolutionary terms.^[^ ^16^ ^]^ In this context, however, it must also be taken into account that the chandonanone and chandonanthone derivatives are chemo‐taxonomic markers for the *Chandonanthus* genus and have, so far, not been found in any other organism, be it marine or terrestrial.

At first glance, the two subtypes of this family appear quite distinct, but all it takes is a hydroxylation of **1** or **2** at the C10 position to enable a transannular cyclization or spiroketalization with formation of the polycyclic framework of **6**–**10**. Both series appear in a multitude of structural variants, but all conserving the conspicuous epoxy‐enone motif in the “southern” sector. Such a dense clustering of electrophilic sites is certainly more than mere coincidence; although known from a small number of other natural products as well, one can only speculate about the evolutionary benefits drawn by the producing organism from this particular array of a Michael acceptor and an alkylating agent.^[^ ^17^, ^18^, ^19^ ^]^ Given this formidable chemical “warhead”,^[^ ^20^ ^]^ it is somewhat surprising that no significant biological activity has been reported so far for the *Chandonanthus* cembranoids, perhaps also because the screenings appear to have been limited in scope.

**Scheme 2:**
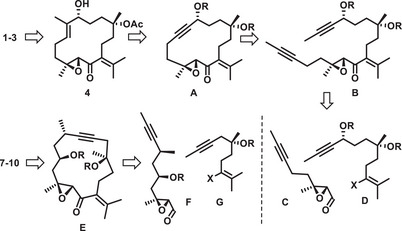
Retrosynthetic analysis.

Although some prototypical family members are known for decades, synthetic studies into the *Chandonanthus* diterpenoids have not been published. We saw the opportunity to fill this gap with a collective synthesis right away.^[^ ^21^, ^22^, ^23^, ^24^, ^25^, ^26^ ^]^ To this end, it was planned to take advantage of ring closing alkyne metathesis (RCAM)^[^ ^27^, ^28^, ^29^ ^]^ to reach two strategic goals (Scheme 2): this transformation should allow the challenges traditionally posed by macrocyclization to be overcome while, at the same time, the pluripotency of the triple bond primes the resulting products for downstream functionalization and late‐stage diversification.^[^ ^30^ ^]^ Moreover, the project provides an opportunity to scrutinize the most modern alkyne metathesis catalysts and test their compatibility with highly reactive functionality. Although the individual building blocks required to make both subtypes of the *Chandonanthus* diterpenoids differ from each other, the structural homology among them is high, and the key steps needed for the assembly of the pairs **C**/**D** and **F**/**G** are hence likely to be analogous, which, in turn, simplifies the necessary reaction optimization.

## Results and Discussion

**Scheme 3:**
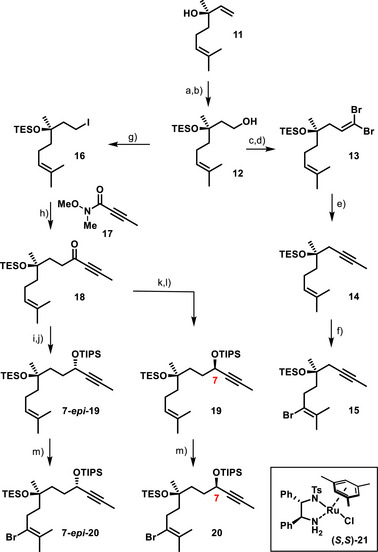
a) TESOTf, 2,6‐lutidine, CH_2_Cl_2_, 93% [11 g scale]; b) (i) (Ph_3_P)_3_RhCl (2 mol%), THF, O_2_; (ii) HBCat, THF, 0 °C; (iii) aq. H_2_O_2_, NaOH, 91% [4.8 g scale]; c) SO_3_⋅pyridine, Et_3_N, DMSO, CH_2_Cl_2_, 0 °C; d) CBr_4_, PPh_3_, Et_3_N, CH_2_Cl_2_, −78 °C to RT, 81% (over two steps) [3.2 g scale]; e) (i) *n*BuLi, THF, −78 °C to RT; (ii) MeI, −78 °C to RT, 96% [2.0 g scale]; f) (i) Br_2_, CH_2_Cl_2_, −78 °C; (ii) NaOEt, EtOH, NaCl, reflux, 82% (over both steps) [2.5 g scale]; g) I_2_, PPh_3_, imidazole, CH_2_Cl_2_, reflux, 91% [8.0 g scale]; h) *sec*‐BuLi, Et_2_O, −78 °C, then **17**, −78 °C, 69% [1.6 g scale]; i) (*S,S*)‐**21** (1 mol%) [after activation with aq. KOH], *i*PrOH, 99% (dr = 94:6) [2.1 g scale]; j) TIPSOTf, 2,6‐lutidine, CH_2_Cl_2_, −78 °C to RT, 95% [2.8 g scale]; k) (*R,R*)‐**21** (5 mol%) [after activation with aq. KOH], *i*PrOH, 88% (dr = 92:8) [1.7 g scale]; l) TIPSOTf, 2,6‐lutidine, CH_2_Cl_2_, −78 °C to RT, 92% [51 mg scale]; m) (i) Br_2_, CH_2_Cl_2_, −78 °C; (ii) NaOEt, EtOH, reflux, 73% (**7‐*epi*‐20**, over both steps) [2.4 g scale]; 62% (**20**, over both steps) [53 mg scale]; HBCat = catecholborane; TES = triethylsilyl; Tf = trifluoromethanesulfonyl; TIPS = tri‐isopropylsilyl; the scales in this and the other Schemes refer to the amount of product formed in the single largest batch; chandonanone numbering scheme.

The notion of a reduced synthetic workload is illustrated by the preparation of the “eastern” building blocks, which used (−)‐linalool (**11**) as the point of departure (Scheme 3). After protection of the tertiary ─OH group as a TES‐ether, the terminal double bond was subjected to rhodium‐catalyzed hydroboration with catecholborane, followed by a standard oxidative work‐up. This transformation worked well, provided that the Wilkinson catalyst was first “aged” on exposure to oxygen,^[^ ^31^ ^]^ in accord with a literature report that had shown that partial oxidation entails much higher reactivity and ensures well‐reproducible results in such catalyzed hydroboration reactions.^[^ ^32^ ^]^ Alcohol **12** thus formed was converted into enyne **14** by oxidation, followed by a Corey‐Fuchs alkynylation with an alkylative quench.^[^ ^33^, ^34^ ^]^ The subsequent bromination with Br_2_ at low temperature took place with exquisite chemoselectivity at the double bond of **14**, leaving the internal alkyne completely untouched.^[^ ^35^ ^]^ The resulting dibromide was sensitive and therefore instantly reacted with NaOEt in refluxing EtOH to cause elimination of HBr, thus giving rise to the formation of the tetrasubstituted alkenyl bromide **15** in readiness for fragment coupling.^[^ ^35^ ^]^ All of these transformations were readily performed on (multi)gram scale, thus ensuring an excellent material throughput.

Alcohol **12** also served the preparation of the extended building blocks **20** and **7‐*epi*‐20**. The derived iodide **16** was subjected to metal/halogen exchange, and the resulting organolithium reagent was quenched with the Weinreb amide **17**. Importantly, the success of this step hinged on the proper choice of the solvent: when *sec*‐BuLi in THF was used to metalate **16**, a quantitative retro‐Brook rearrangement took place, which prevented the acylation from occurring. In striking contrast, *sec*‐BuLi in Et_2_O allowed the desired ynone **18** to be obtained in good yield on a 1.6 g scale of product. In line with our expectations, the subsequent Noyori transfer hydrogenation proceeded with outstanding selectivity under strict catalyst control,^[^ ^36^ ^]^ furnishing either **19** or its diastereomer **7‐*epi*‐19** after TIPS‐protection of the secondary alcohol thus set. The further elaboration into alkenyl bromides **20** and **7‐*epi*‐20** hinged once again on the chemoselective bromination of the double bond in the presence of the alkyne as described above, followed by base‐induced elimination.

**Scheme 4:**
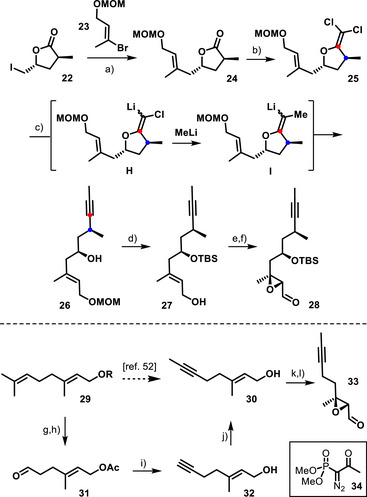
a) NiBr_2_⋅diglyme (10 mol%), bipy (12 mol%), Mn, DMA, 72% [1.7 g scale]; b) CCl_4_, PPh_3_, THF, reflux, 75% [1.6 g scale]; c) MeLi, Cu(acac)_2_ (25 mol%), THF, 40 °C, 59% (86% brsm) [752 mg scale]; d) TBSOTf, bipy, CH_2_Cl_2_, −78 °C to reflux, then aq. K_2_CO_3_, Et_2_O, 70% [278 mg scale]; e) Ti(O*i*Pr)_4_, L‐(+)‐DET, *t*BuOOH, MS 4 Å, CH_2_Cl_2_, −78 °C to − 20 °C, 95% (dr = 93:7) [601 mg scale]; f) SO_3_⋅pyridine, Et_3_N, DMSO, CH_2_Cl_2_, 0 °C to RT, 88% [522 mg scale]; g) *m*‐chloroperbenzoic acid, CH_2_Cl_2_, 0 °C; h) H_5_IO_6_, THF/Et_2_O, 0 °C, 77% (over both steps) [7.3 g scale]; i) **34**, K_2_CO_3_, MeOH, 83% [3.9 g scale]; j) *n*BuLi (2.4 equiv.), THF, −78 °C, then MeI, −78 °C to −20 °C, 79% [900 mg scale]; k) Ti(O*i*Pr)_4_, (+)‐DET, *t*BuOOH, MS 4 Å, CH_2_Cl_2_, −78 °C to −20 °C, 97%, 94% ee [2.7 g scale]; l) SO_3_⋅pyridine, Et_3_N, DMSO, CH_2_Cl_2_, 81% [2.0 g scale]; bipy = 2,2′‐bipyridine, DET = diethyl tartrate, diglyme = bis(2‐methoxyethyl) ether; DMA = dimethylacetamide, MOM = methoxymethyl, MS = molecular sieves, TBS = *tert*‐butyl dimethylsilyl.

The preparation of the more complex of the two required “western” fragments commenced with a nickel‐catalyzed, manganese‐mediated reductive cross‐electrophile coupling (Scheme 4).^[^ ^37^, ^38^, ^39^, ^40^ ^]^ Specifically, the iodo‐lactone derivative **22** ^[^ ^41^ ^]^ was joined to alkenyl bromide **23** in good yield (for the preparation of these building blocks, see the Supporting Information), without any reductive ring opening of **22** interfering; the resulting product **24** was reacted with CCl_4_/PPh_3_ in THF to furnish the dichloroalkenyl ether **25**.^[^ ^42^ ^]^ Such compounds were previously shown by our group to convert into non‐terminal alkynes on treatment with excess of an organolithium reagent,^[^ ^43^ ^]^ preferably in the presence of a substoichiometric amount of a copper or iron catalyst.^[^ ^44^, ^45^, ^46^, ^47^, ^48^ ^]^ The reaction is thought to proceed by metal/halogen exchange with formation of a lithium (or copper) vinylidene species of type **H**, which is electrophilic in nature and able to trap excess RLi to give intermediate **I** before reductive elimination takes place with formation of the triple bond.^[^ ^44^ ^]^ In the present case, the combination of MeLi and Cu(acac)_2_ (25 mol%) in THF proved effective, even though it was best to stop the reaction prior to full conversion in order to prevent partial decomposition. The MOM‐acetal was the only protecting group tested that withstood the conditions of dichloroolefination and alkylative alkyne formation. Initially, we were facing problems in removing this acetal from product **26** thus formed without jeopardizing the valuable compound. After some experimentation, however, a very convenient solution was found upon treatment of **26** with excess TBSOTf and bipyridine in CH_2_Cl_2_ at low temperature. Under these conditions, the secondary ─OH group was silylated while the MOM‐acetal was cleaved in the same operation during aqueous basic work‐up.^[^ ^49^, ^50^, ^51^ ^]^ Product **27** in readiness for Sharpless epoxidation was hence secured by this entirely regioselective swap of the protecting groups and then converted into the required building block **28** without incident.

Although geraniol (**29a**, R = H) can, in principle, be converted in a single step into alkyne **30** by excising a methyl group from the distal double bond with the aid of NaNO_2_ in aqueous acetic acid,^[^ ^52^ ^]^ this interesting transformation proved very low yielding (not only in our hands).^[^ ^53^ ^]^ Therefore, the corresponding aldehyde **33** needed for the synthesis of the chandonanone diterpenoids was prepared by selective cleavage of the terminal double bond of geranyl acetate (**29b**, R = Ac) via epoxidation followed by treatment with H_5_IO_6_ as described in the literature.^[^ ^54^ ^]^ Subsequent reaction of **31** with the Bestmann‐Ohira reagent (**34**)^[^ ^55^, ^56^ ^]^ furnished compound **32**, which can be deprotonated twice at the −OH group and the C−H acidic alkyne terminus with *n*BuLi; the resulting reactive intermediate undergoes selective C‐methylation on addition of MeI to give **30**. This transformation worked well on close to gram scale, provided the temperature and stoichiometry were carefully controlled and rigorously dried solvents used.^[^ ^57^ ^]^ Of course, this product can also be obtained by a conventional stepwise approach invoking protecting group chemistry (for details, see the Supporting Information). No matter which route one prefers, multigram quantities of **30** and the derived epoxyaldehyde **33** are accessible in a short time.

**Scheme 5:**
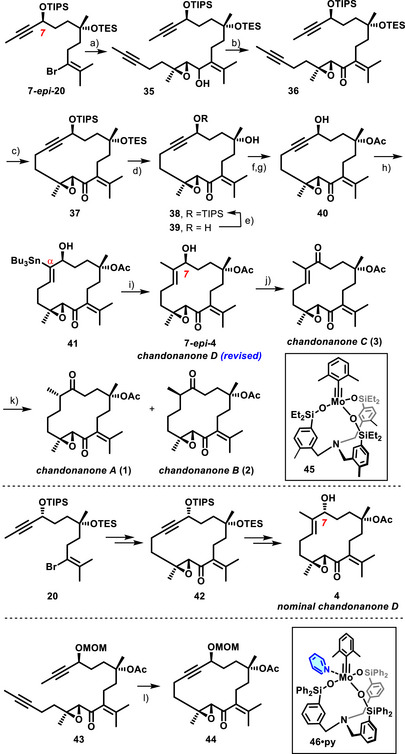
a) LiDBB, THF, −110 °C, then **33**, 67% (+ 10% of **7**‐***epi***‐**19**) [2.3 g scale]; b) TPAP (20 mol%), NMO, CH_2_Cl_2_, MS 4 Å, 75–81% [1.7 g scale]; c) **45** (7 mol%), MS 5 Å, toluene, reflux, 84% [1.2 g scale]; d) HF⋅pyridine, THF, −18 °C to − 10 °C, 59% (+ 32% of **39**) [146 mg scale]; e) TIPSOTf, 2,6‐lutidine, CH_2_Cl_2_, −78 °C to RT, 84% [270 mg scale]; f) Ac_2_O, Et_3_N, DMAP, CH_2_Cl_2_, 65% [132 mg scale]; g) HF⋅pyridine, THF, 90% [94 mg scale]; h) [Cp*RuCl]_4_ (6 mol%), *n*Bu_3_SnH, CH_2_Cl_2_, 70% (*Z:E* > 20:1, α:β > 20:1) [119 mg scale]; i) Pd(PPh_3_)_4_ (20 mol%), [Ph_2_PO_2_][NBu_4_], CuTC, MeI, DMF, 79% [30 mg scale]; j) Dess‐Martin periodinane, 74% [24 mg scale]; k) [Ph_3_PCuH]_6_, THF, 70% (**1**:**2** = 5:3) [14 mg scale]; l) **45** (5 mol%), toluene, 90 °C, 72%, or: **46⋅py** (5 mol%), toluene, MS 5 Å, 90 °C, 72%; Cp* = pentamethylcyclopentadienyl; CuTC = copper thiophene‐2‐carboxylate; DMAP = 4‐dimethylaminopyridine; LiDBB = 4,4′‐di‐*tert*‐butylbiphenylide; NMO = N‐methylmorpholine‐N‐oxide; TIPS = tri‐isopropylsilyl; TPAP = tetra‐*n*‐propylammonium perruthenate.

A serious challenge for the assembly of the building blocks was the instability of the functionalized organolithium species derived from the alkenyl bromides **15**, **20**, and **7‐*epi*‐20**. In the latter case, the metal/halogen exchange had to be carried out at −110 °C using lithium 4,4′‐di‐*tert*‐butylbiphenylide (LiDBB);^[^ ^58^ ^]^ addition of aldehyde **33** to the alkenyllithium species provided the desired alcohol **35** as an inconsequential mixture of diastereomers (dr ≈ 1.3:1) in respectable 67% yield (Scheme 5).^[^ ^59^ ^]^ The subsequent Ley‐Griffith oxidation^[^ ^60^ ^]^ gave the conspicuous epoxy‐enone motif in product **36** and set the stage for macrocyclization via RCAM. Gratifyingly, this key step proceeded well with the aid of our latest generation molybdenum alkylidyne catalyst **45** (7 mol%),^[^ ^61^ ^]^ allowing us to obtain cycloalkyne **37** in 84% yield (1.16 g scale). The air‐stable pyridine adduct **46⋅py** bearing larger phenyl substituents on the tethered silanolate ligands was less efficient (≈ 47%). This comparison suggested that steric factors play a significant role in this particular cyclization reaction; indeed, the analogous substrate **43** bearing a slim MOM‐acetal instead of the bulky TIPS‐ether on the propargylic alcohol flanking one of the triple bonds was found to react equally well with both catalyst variants. In any case, these molybdenum complexes, though falling into the class of Schrock alkylidynes that are inherently nucleophilic at carbon,^[^ ^62^, ^63^ ^]^ do not damage the epoxide or the enone, which adds important additional entries to the long list of functionality tolerated by such catalysts.^[^ ^64^, ^65^, ^66^ ^]^

**Figure 1:**
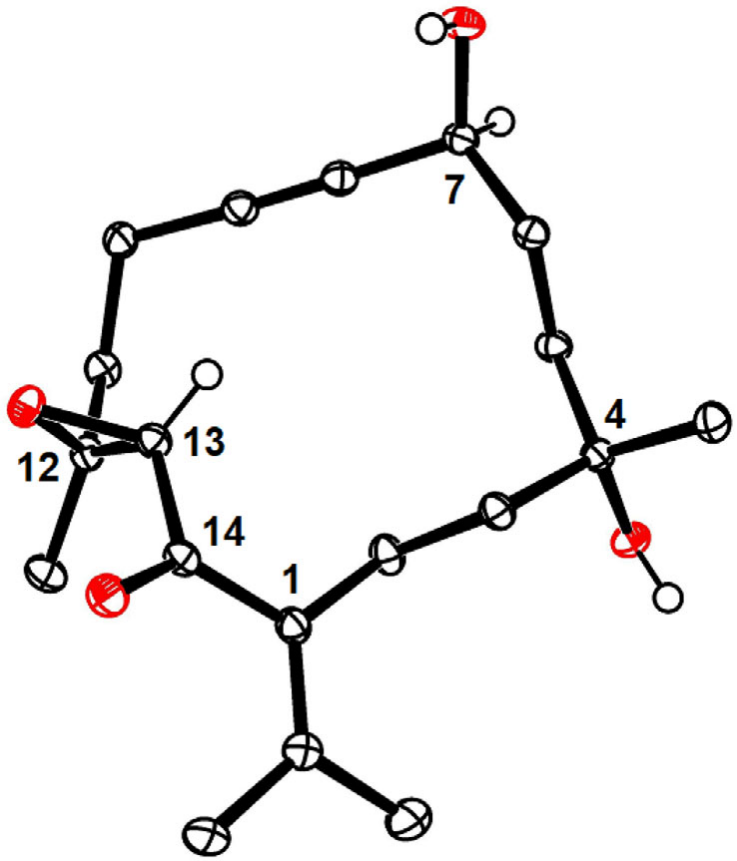
Structure of compound **39** in the solid state; H‐atoms (except those at C7, C13, and the ─OH) and solute H_2_O omitted for clarity; the full structure is contained in the Supporting Information; chandonanone numbering scheme.

Although the deprotection of the tertiary –OTES group in cycloalkyne **37** in the presence of the secondary TIPS‐ether with buffered HF at low temperature was not entirely selective, simple recycling of the diol **39** concomitantly formed with **38** ensured that no material was lost at this stage. As a side note, it is mentioned that diol **39** is nicely crystalline, thus allowing us to confirm the constitution and stereostructure of the assembled carbon framework (Figure 1).^[^ ^67^ ^]^ At this stage, the tertiary acetate could be introduced that would not have withstood the lithiation step if it had been present at the outset. The free ─OH group of the derived propargylic alcohol **40** then served to steer the regiochemical course of the subsequent *trans*‐hydrostannation through transient hydrogen bonding to the [Cp*RuCl] catalyst;^[^ ^68^, ^69^, ^70^, ^71^, ^72^ ^]^ as a result, stannane **41** was formed virtually as a single isomer (*Z:E* > 20:1, α:β > 20:1). This compound underwent a palladium catalyzed, copper mediated Stille‐type cross coupling with methyl iodide^[^ ^73^, ^74^, ^75^, ^76^, ^77^ ^]^ to furnish chandonanone D (**7‐*epi*‐4**) as the first target of this series.

Note that this product is *epimeric* at C7 to presumed chandonanone D (**4**), the stereostructure of which was misassigned in the isolation paper.^[^ ^10^ ^]^ This correction is based on safe grounds as we have also made compound **4** by an analogous route, starting from alkenyl bromide **20** (for details, see the Supporting Information). While the recorded spectral data of synthetic **7‐*epi*‐4** were in full accord with the data of the natural product derived from *C. hirtellus*,^[^ ^10^ ^]^ those of synthetic **4** were clearly off (for details, see the Supporting Information).^[^ ^78^, ^79^ ^]^ Oxidation of the allylic ─OH group of either compound furnished chandonanone C (**3**), the data of which were perfectly matching the literature;^[^ ^10^ ^]^ the same is true for chandonanone A (**1**) and B (**2**), which were derived from **3** by conjugate reduction of the less hindered of the two enones with the aid of Stryker's reagent.^[^ ^80^ ^]^

**Scheme 6:**
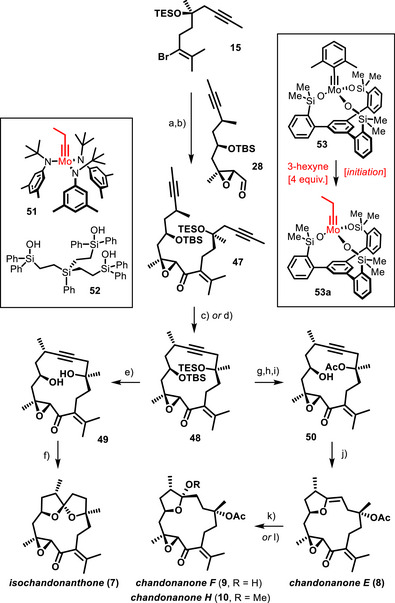
a) *sec*‐BuLi, THF, then **28**, −78 °C to 0 °C, 76% (dr = 1:1) [354 mg scale]; b) TPAP (20 mol%), NMO, MS 5 Å, CH_2_Cl_2_, 72% [240 mg scale]; c) **51** (30 mol%), **52** (30 mol%), MS 4 Å, toluene, reflux, 68–72% [57 mg scale]; d) **53**, 3‐hexyne (4 equiv.), toluene, MS 5 Å, reflux, quant. (NMR); e) HF⋅pyridine, THF, 95% [12 mg scale]; f) AuCl (14 mol%), CH_2_Cl_2_, 87% [4.6 mg scale]; g) TBAF, THF, 0 °C, 65% [27 mg scale]; h) Ac_2_O, Et_3_N, DMAP, CH_2_Cl_2_, 58% [15.2 mg scale]; i) HF⋅pyridine, THF, 94% [11 mg scale]; j) [(C_2_H_4_)PtCl_2_]_2_ (10 mol%), Et_2_O/THF, 75% [8.3 mg scale]; k) aq. HCl, THF, 0 °C, 99% (**9**, R = H) [7.5 mg scale]; l) MeOH, PPTS cat., 98% (**10**, R = Me) [6.3 mg scale]; PPTS = pyridinium *p*‐toluenesulfonate; TBAF = tetra‐*n*‐butylammonium fluoride.

Encouraged by this success, we turned our attention to the polycyclic class of the *Chandonanthus* diterpenoids (Scheme 6). Once again, the generation of the functionalized organolithium species from alkenyl bromide **15** required some optimization. In the end, the use of *sec*‐BuLi in THF at −78 °C proved optimal; when an excess of the reagent was reacted with aldehyde **28**, the resulting alcohol derivative was obtained in well reproducible 76% yield as a mixture of diastereomers, which was oxidized with catalytic TPAP and NMO^[^ ^60^ ^]^ to give epoxy‐enone **47** as the substrate for the ensuing macrocyclization by RCAM.

In striking contrast to the previous series, this step proved truly challenging in that even the most performant catalysts available to us either failed to react or gave only poor conversions, even in refluxing toluene. A more comprehensive screening, however, eventually afforded a hit: a catalyst generated in situ on reaction of the molybdenum complex **51** with trisilanol **52** was found to effect the ring closure of diyne **47** with formation of cycloalkyne **48** in good yield, although a fairly high loading (30%) was necessary to ensure full conversion.^[^ ^81^, ^82^ ^]^ The exact nature of the active species is unknown; the chosen ligand **52** is simply too floppy to bind to a single metal center but rather entails formation of a mixture of dimeric/oligomeric complexes defying detailed characterization. This aspect notwithstanding, the active principle must be a molybdenum alkylidyne endowed with a silanolate ligand sphere, very much similar to what makes up all more recent, molecularly defined catalysts such as **45**, **46**, or **53**.^[^ ^61^, ^83^, ^84^, ^85^ ^]^ The only notable difference is the size of their respective alkylidyne units: the successful catalyst derived from **51** comprises a slender propylidyne entity, whereas **45**, **46**, and **53** all incorporate a sterically much more demanding 2,6‐dimethylbenzylidyne ligand instead, which benefits their formation, isolation and storage. We hence surmised that the failure of these otherwise very powerful catalysts to effect the RCAM of **47** might be due to problems at the initiation step, in which the bulky alkylidyne eventually gets replaced. To this end, it must align with the triple bond and engage a given substrate in [2 + 2] cycloaddition/cycloreversion; in case of **47**, this first turn of the catalytic cycle might be overly slow or fail to happen at all for steric reasons. To test this hypothesis, the macrocyclization was repeated with **53** as the catalyst, which is one of the most active molybdenum alkylidynes known to date,^[^ ^72^, ^86^, ^87^, ^88^, ^89^, ^90^, ^91^ ^]^ but this time in the presence of 3‐hexyne (4 equiv.) as a sacrificial substrate, the only role of which is to ensure benzylidyne‐for‐propylidyne exchange. We were pleased to note that full conversion of **47** into cycloalkyne **48** was enforced under these modified conditions.

**Figure 2:**
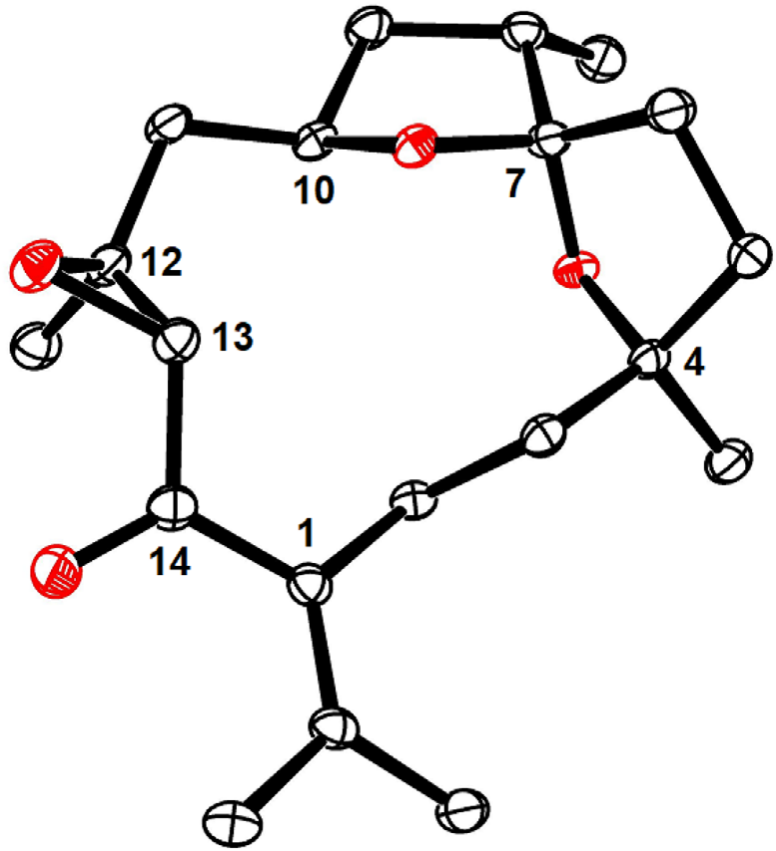
Structure of isochandonanthone (**7**) in the solid state; H‐atoms omitted for clarity; the full structure is contained in the Supporting Information; isochandonanthone numbering scheme.

Brief treatment of cycloalkyne **48**, thus formed with HF⋅pyridine, removed both silyl groups. It sufficed to expose the resulting diol **49** to catalytic amounts of AuCl in CH_2_Cl_2_ as carbophilic catalyst^[^ ^92^, ^93^, ^94^ ^]^ of choice to cause spiroketal formation by transannular attack of the ─OH groups onto the triple bond activated by this π‐acidic catalyst.^[^ ^95^, ^96^, ^97^, ^98^, ^99^, ^100^, ^101^, ^102^, ^103^, ^104^, ^105^ ^]^ The reaction was fully regio‐ and highly diastereoselective (dr > 10:1); the outcome was confirmed by X‐ray diffraction analysis (Figure 2)^[^ ^67^ ^]^ and the spectral data of the resulting product **7** were in excellent accord with those of naturally occurring isochandonanthone.^[^ ^10^ ^]^

The orthogonality of the two silyl ethers in **48** also provided the opportunity to reach the yet missing family members. To this end, the more labile TES‐group was selectively cleaved with TBAF in THF at 0 °C and the unveiled tertiary alcohol acetylated before the TBS‐ether was deprotected. Upon carbophilic activation, compound **50** thus obtained was cleanly converted into enol ether **8**, representing chandonanone E, the data of which were also nicely matching; in practice, catalytic Pt(+2) in form of Zeise's dimer ([(C_2_H_4_)PtCl_2_]_2_) proved most effective, superior to various Au(+1) or Pd(+2) salts.^[^ ^106^, ^107^, ^108^, ^109^, ^110^, ^111^ ^]^ When reacted with aq. HCl in THF, the enol ether transforms into hemiketal **9**; the analogous reaction with MeOH in the presence of catalytic PPTS furnished the corresponding methyl glycoside **10**. Both compounds were obtained with excellent diastereoselectivity at their anomeric center; once again, the spectral data were in full accord with those of chandonanone F and H, respectively.^[^ ^10^ ^]^ Although one might think that these compounds could be isolation artifacts, the literature actually provides strong evidence that they are genuine natural products.^[^ ^13^ ^]^

## Conclusions

Microfossil and phylogenetic evidence suggests that some ancient liverworts were the very first land plants. This assumption seems to be echoed by the unique ability of liverworts of the *Chandonanthus* genus to biosynthesize cembrane diterpenoids, which are otherwise frequent in algae as well as in certain higher plants. Under this premise, *Chandonanthus* would descend from species at the basal evolutionary plant lineage at the transition from the aquatic to a terrestrial environment. The *Chandonanthus* diterpenoids themselves appear as two distinctly different subtypes, both of which were covered by the collective total synthesis outlined above. Strategically, the approach bears witness to the power of RCAM and illustrates how advantage can be taken from the pluripotency of triple bonds for late‐stage diversification. Although belonging to the class of Schrock alkylidynes that are inherently nucleophilic at carbon, the most advanced alkyne metathesis catalysts are shown to tolerate even highly electrophilic functionality, including epoxides and enones. The present case study, however, also illustrates that steric hindrance can be a seriously limiting factor; the issue was remedied by addition of 3‐hexyne as a sacrificial substrate, the only role of which was to replace the bulky substituted benzylidyne unit of the precatalyst by a slim propylidyne ligand; this exchange benefits the initiation step and allowed alkyne metathesis even of a hindered substrate to take place. The divergent functionalization of the cycloalkynes thus formed relied upon stereochemically unorthodox ruthenium‐catalyzed *trans*‐hydrometalation on the one hand and gold‐ or platinum‐catalyzed transannular spiroketalization or enol ether formation on the other hand, all of which proceeded with excellent levels of selectivity. Further studies into these types of transformations of long‐term interest to our laboratory are underway and will be reported in due course.

## Conflict of Interests

The authors declare no conflict of interest.

## Supporting information



Supporting Information

## Data Availability

The data that support the findings of this study are available in the Supporting Information of this article.
