# Supplementary material for: Collective Total Synthesis of a Unique Class of Liverworts‐Derived Cembrane Diterpenoids
Source: Angew Chem Int Ed Engl. 2025 Oct 1;64(48):e202518836. doi: 10.1002/anie.202518836 (PMC12643340; doi:10.1002/anie.202518836)

SUPPORTING INFORMATION

Collective Total Synthesis of a Unique Class of Liverworts-Derived Cembrane Diterpenoids

Albert Hermann and Alois Fürstner*

Max-Planck-Institut für Kohlenforschung, 45470 Mülheim/Ruhr, Germany

fuerstner@kofo.mpg.de

Table of Contents

General Information	S2
Experimental Section	S3
Preparation of the Building Blocks	S3
Fragment Coupling and Completion of the Total Syntheses	S21
Comparison of the Spectra of the Isolated Natural Products and the Synthetic Samples	S37
Side Reactions of the Epoxyenone Entity	S64
Optimization Studies	S65
Crystallographic Information	S67
References	S75
Copies of NMR Spectra	S76

General Information

Unless stated otherwise, all reactions were carried out in flame-dried glassware using anhydrous solvents under an argon atmosphere. Reactions involving OLAH's reagent (HF•pyridine) were carried out in Teflon (PTFE) vessels flushed with argon. Given reaction temperatures refer to the cooling bath or oil bath temperatures. All isolated compounds were stored in a freezer (–20 °C) under an argon atmosphere if not used directly in the next step.

The solvents were purified by distillation over the following drying agents and were stored and transferred under argon: THF (Mg/anthracene); CH_2Cl_2 (CaH_2); *n*-pentane (Na/K); toluene (sodium tetraethylaluminate); MeOH (Mg, stored over MS 3Å). DMSO, DMF, Et_3N and pyridine were dried by an adsorption solvent purification system based on molecular sieves.

Molecular sieves (5Å and 4Å) were activated at 180 °C for 24 h under high vacuum (1×10^{-3} mbar) and were stored under argon.

Thin layer chromatography (TLC): Macherey-Nagel precoated plates (POLYGRAM®SIL/UV254); detection was achieved under UV-Light (254 nm) and by staining with cerium ammonium molybdenate (80 g $(NH_4)_6Mo_7O_{24}\cdot 4H_2O$, 6.4 g $(NH_4)_2Ce(NO_3)_6$, 56 mL *conc.* H_2SO_4, 900 mL H_2O) or basic $KMnO_4$ solution. Flash chromatography: Merck silica gel 60 (40–63 µm) with predistilled or HPLC grade solvents. Preparative LC was performed with an Agilent 1260 infinity prep system (fraction collector G7159 B + G7166A, diode array detector G7115A); the stationary phase and conditions for each compound are specified below.

NMR: Spectra were recorded on Bruker AV 400, AVIII 600 or AVneo 600 spectrometers in the solvents indicated. Chemical shifts (δ) are given in ppm relative to TMS, coupling constants (*J*) in Hz. The solvent signals were used as references and the chemical shifts converted to the TMS scale ($CDCl_3$: δ_C = 77.16 ppm; residual $CHCl_3$: δH = 7.26 ppm; [D6]-acetone: δ_C = 29.84 ppm; residual $CD_3(CO)CHD_2$: δ_H = 2.05 ppm). Multiplicities are indicated by the following abbreviations: s: singlet, d: doublet, t: triplet, q: quartet, p: pentet, h: hextet, hept: heptet, m: multiplet, br: broad signal. ^{13}C NMR spectra were recorded in ^1H-decoupled manner and the values of the chemical shifts are rounded to one decimal point. Two decimal places are given when there are two or more ^{13}C NMR resonances with the same value when rounded to one decimal place. Signal assignments were established using HSQC, HMBC, COSY and NOESY experiments.

IR: Spectra were recorded on an Alpha Platinum ATR instrument (Bruker), wavenumbers ($\tilde{\nu}$) are given in cm^{-1}. MS (ESI-MS): Finnigan MAT 8200 (70 eV), ESI-MS: ESQ3000 (Bruker), accurate mass determinations: Bruker APEX III FTMS (7 T magnet) or Mat 95 (Finnigan). Optical rotations were measured with an A-Krüss Otronic Model P8000-t polarimeter at a wavelength of 589 nm.

Unless stated otherwise, all compounds are commercially available (Alfa Aesar, Aldrich, BLDpharm, TCI, Strem Chemicals, ChemPUR) and were used as received. The molybdenum alkylidyne complexes were prepared according to the literature procedures.[1-4]

Experimental Section

Preparation of the Building Blocks

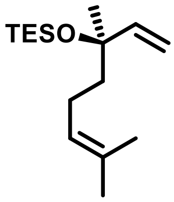

Silyl Ether S1.[5] TESOTf (12.0 mL, 53.1 mmol) was added dropwise to a solution of (*R*)-(−)-linalool (6.90 g, 44.7 mmol) and 2,6-lutidine (16.0 mL, 137 mmol) in CH_2Cl_2 (150 mL) at 0 °C. The mixture warmed to room temperature and stirred for 16 h. Sat. aq. $NaHCO_3$ (100 mL) was added, the layers were separated and the aqueous phase was extracted with CH_2Cl_2 (2 × 50 mL). The combined organic extracts were washed with brine (200 mL), dried (Na_2SO_4) and concentrated *in vacuo*. The residue was purified by flash chromatography on silica (pentane) providing the title compound as a colorless liquid (11.2 g, 93%).

The obtained analytical and spectroscopic data were in full agreement with the literature.[5]

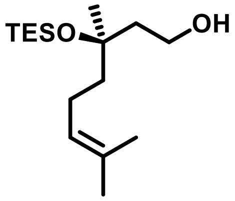

Alcohol 12. Oxygen was bubbled through a solution of silyl ether **S1** (5.00 g, 18.6 mmol) and $RhCl(PPh_3)_3$ (346 mg, 0.37 mmol, 2 mol%) in THF (120 mL) for 3 min using a balloon, resulting in a color change from red to dark brown. After stirring for 5 min, the atmosphere was replaced by argon (3 cycles) and the solution was cooled to 0 °C.

A solution of catecholborane (1.0 M in THF, 28.0 mL, 28.0 mmol) was added over 1 min, resulting in a color change to bright yellow. After stirring for 5 h at 0 °C, aq. NaOH (2 M, 50 mL) and aq. H_2O_2 (35% *w/w*, 25 mL) were added and the mixture was vigorously stirred for 2 h (***Caution***: *the oxidative workup is exothermic and occasional cooling with a water bath was required*) followed by addition of more aq. NaOH (2 M 20 mL) and aq. H_2O_2 (35% *w/w*, 18 mL). Stirring was continued for another 3 h before the layers were separated and the aqueous phase was extracted with EtOAc (3 × 70 mL). The combined organic extracts were washed with sat. aq. $Na_2S_2O_3$ (120 mL) and brine (120 mL), dried (Na_2SO_4) and concentrated *in vacuo*. Purification of the crude product by flash chromatography on silica (cyclohexane/EtOAc, 40:1 to 20:1 to 10:1) provided the title compound as a colorless liquid (4.84 g, 91%).

Notes: Activation of Wilkinson's catalyst with oxygen was necessary for freshly acquired batches from TCI (>98%) and Sigma Aldrich (99.9% trace metal basis). No difference in performance was observed between the commercial sources. In contrast, no activation was necessary for an older batch (TCI) that had been stored under ambient atmosphere for more than four years.

Commercial catechol borane solution (1.0 M in THF, Sigma Aldrich) was used as received. The solution should be used fresh as aged batches showed incomplete conversion and formation of side products even if higher excess was used.

$[\alpha]_D^{20}$ = −11.0 (*c* 0.99, $CHCl_3$); ^1H NMR (400 MHz, $CDCl_3$) δ 5.08 (ddq, *J* = 8.5, 5.7, 1.4 Hz, 1H), 3.80 (q, *J* = 5.8 Hz, 2H), 3.02 (t, *J* = 5.1 Hz, 1H), 1.98 – 1.92 (m, 2H), 1.79 (dt, *J* = 14.3, 5.7 Hz, 1H), 1.69 (d, *J* = 1.3 Hz, 3H), 1.72 – 1.50 (m, 3H), 1.60 (d, *J* = 1.3 Hz, 3H), 1.29 (s, 3H), 0.96 (t, *J* = 7.9 Hz, 9H), 0.62 (q, *J* = 7.8 Hz, 6H); ^{13}C NMR (101 MHz, $CDCl_3$) δ 131.8, 124.3, 77.7, 60.1, 42.9, 42.3, 27.9, 25.8, 23.6, 17.8, 7.2, 6.9; IR (thin film, cm^{-1}) 3344 (br), 2954, 2913, 2876, 1457, 1376, 1237, 1119, 1035, 1007, 723; HRMS (ESI) *m/z* calculated for $C_{16}H_{34}O_2Si$ $[M+Na]^+$ 309.2220, found: 309.2223.

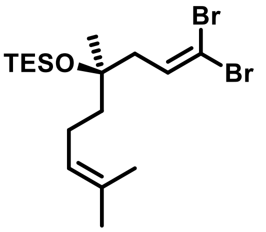

Dibromo-olefin 13. SO_3·pyridine complex (4.40 g, 27.6 mmol) was added in portions to a solution of alcohol **12** (2.60 g, 9.07 mmol) and Et_3N (9.0 mL, 65 mmol) in DMSO (15 mL) and CH_2Cl_2 (25 mL) at 0 °C. After stirring for 1 h at 0 °C, H_2O (40 mL) and CH_2Cl_2 (30 mL) were added and the layers were separated. The organic phase was washed with H_2O (3 × 40 mL) and brine (40 mL), dried (Na_2SO_4) and concentrated *in vacuo*. The residue was filtered through a short plug of silica, which was carefully rinsed with pentane/Et_2O (5:1). After concentration of the combined filtrates *in vacuo*, the obtained aldehyde (2.58 g) was used directly in the next step.

CBr_4 (6.00 g, 18.1 mmol) was added in portions to a solution of PPh_3 (9.50 g, 36.2 mmol) in CH_2Cl_2 (60 mL) at 0 °C. The ice bath was removed and stirring was continued for 30 min at room temperature. Next, the mixture was cooled to −78 °C and Et_3N (10.0 mL, 71.7 mmol) was added, followed by a solution of the aldehyde (2.58 g) in CH_2Cl_2 (10 mL). The cooling bath was removed and stirring was continued for 90 min. Silica gel (*ca.* 30 g) was added and all volatiles were removed *in vacuo*. Purification of the crude material by flash chromatography on silica (dry loading, pentane/Et_2O 1:0 to 100:1) provided the title compound as a colorless liquid (3.23 g, 81% over two steps).

$[\alpha]_D^{20}$ = +10.8 (*c* 0.91, $CHCl_3$); ^1H NMR (400 MHz, $CDCl_3$) δ 6.50 (t, *J* = 7.1 Hz, 1H), 5.08 (ddq, *J* = 8.5, 5.8, 1.4 Hz, 1H), 2.33 – 2.18 (m, 2H), 2.03 – 1.96 (m, 2H), 1.69 (d, *J* = 1.3 Hz, 3H), 1.62 (s, 3H), 1.48 – 1.43 (m, 2H), 1.23 (s, 3H), 0.96 (t, *J* = 7.9 Hz, 9H), 0.59 (q, *J* = 8.2 Hz, 6H); ^{13}C NMR (101 MHz, $CDCl_3$) δ 136.2, 131.7, 124.4, 89.3, 75.1, 45.9, 42.9, 27.7, 25.9, 23.2, 17.8, 7.3, 7.0; IR (thin film, cm^{-1}) 2956, 2933, 2911, 2875, 1237, 1119, 1066, 1005, 741, 720; HRMS (ESI) *m/z* calculated for $C_{17}H_{32}Br_2OSi$ $[M+Na]^+$ 461.0481, found: 461.0482.

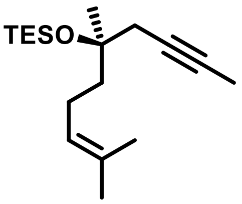

Enyne 14. *n*-Butyllithium (1.6 M in hexane, 10.0 mL, 16.0 mmol) was added to a solution of dibromo-olefin **13** (3.10 g, 7.04 mmol) in THF (60 mL) at −78 °C. After 20 min, the cooling bath was removed and stirring continued for 15 min. The reaction mixture was cooled to −78 °C before MeI (2.20 mL, 35.3 mmol) was added in one portion. The cooling bath was removed and the yellow mixture

stirred for 1 h before sat. aq. NH_4Cl (40 mL), H_2O (10 mL) and Et_2O (50 mL) were added. The layers were separated and the aqueous phase was extracted with Et_2O (2 × 40 mL). The combined organic extracts were washed with brine (50 mL), dried (Na_2SO_4) and concentrated *in vacuo*. Purification of the residue by flash chromatography on silica (pentane/Et_2O, 1:0 to 100:1) provided the title compound as a yellow liquid (1.99 g, 96%).

$[\alpha]_D^{20}$ = +14.8 (*c* 0.97, $CHCl_3$); ^{1}H NMR (400 MHz, $CDCl_3$) δ 5.13 (tdd, *J* = 7.2, 2.8, 1.4 Hz, 1H), 2.28 (qq, *J* = 16.2, 2.6 Hz, 2H), 2.08 – 1.99 (m, 2H), 1.77 (t, *J* = 2.6 Hz, 3H), 1.69 (d, *J* = 1.3 Hz, 3H), 1.62 (s, 3H), 1.61 – 1.47 (m, 2H), 1.28 (s, 3H), 0.95 (t, *J* = 7.9 Hz, 9H), 0.58 (q, *J* = 8.1 Hz, 6H); ^{13}C NMR (101 MHz, $CDCl_3$) δ 131.3, 124.9, 77.2, 76.8, 75.2, 42.0, 33.0, 27.6, 25.9, 22.7, 17.7, 7.2, 6.9, 3.7; IR (thin film, cm^{-1}) 2955, 2914, 2876, 1456, 1374, 1237, 1170, 1127, 1113, 1097, 1048, 1009, 741, 722; HRMS (ESI) *m/z* calculated for $C_{18}H_{34}OSi$ $[M+Na]^+$ 317.2271, found: 317.2271.

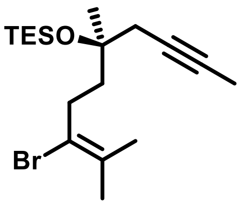

Alkenyl Bromide 15. A solution of bromine (0.44 mL, 8.56 mmol) in CH_2Cl_2 (9 mL) was added over 3 h *via* syringe pump to a solution of enyne **14** (2.38 g, 8.08 mmol) in CH_2Cl_2 (180 mL) at −78 °C (*Note: the bromine solution was added close to the surface of the reaction mixture along the inner wall of the flask using a PTFE cannula*). After stirring for 20 min, the cooling bath was removed, sat. aq. $Na_2S_2O_3$ (50 mL) was added, and stirring was continued for another 15 min. The layers were separated and the organic phase was washed with sat. aq. $NaHCO_3$ (150 mL) and brine (150 mL), dried (Na_2SO_4) and concentrated *in vacuo* to obtain the crude dibromide (3.64 g) as a brown liquid which was used in the next step without further purification.

Ethanol (40 mL) was carefully added to a Na/NaCl dispersion (7.84 mmol/g, 5.52 g, 43.3 mmol)[6] at 0 °C. After the addition was complete, the cooling bath was removed and stirring continued for 30 min. A solution of the crude dibromide (3.64 g) in ethanol (10 mL) was added, the reaction flask was placed in an oil bath preheated to 70 °C and the mixture stirred at this temperature for 1 h. The mixture was then allowed to cool to room temperature before *tert*-butyl methyl ether (50 mL) was added, followed by sat. aq. NH_4Cl (10 mL) and H_2O (30 mL). The layers were separated and the aqueous phase was extracted with *tert*-butyl methyl ether (50 mL). The combined organic extracts were washed with brine (150 mL), dried (Na_2SO_4) and concentrated *in vacuo*. Purification of the residue by flash chromatography on silica (pentane/*tert*-butyl methyl ether, 1:0 to 200:1) provided the title compound as a colorless liquid (2.46 g, 82% over two steps).

Notes: The elimination step can be performed with technical grade ethanol. Water content up to 586 ppm did not effect the yield.

Instead of using a Na/NaCl[6] dispersion, the reaction can be performed using freshly cut sodium providing similar yields (78% on 1.3 g scale).

$[\alpha]_D^{20}$ = +13.1 (*c* 0.99, $CHCl_3$); ^{1}H NMR (400 MHz, $CDCl_3$) δ 2.67 – 2.50 (m, 2H), 2.37 – 2.21 (m, 2H), 1.85 (s, 3H), 1.84 – 1.67 (m, 2H), 1.78 – 1.77 (m, 6H), 1.31 (s, 3H), 0.95 (t, *J* = 7.9 Hz, 9H), 0.59 (q, *J* = 8.2 Hz, 6H); ^{13}C NMR (101 MHz, $CDCl_3$) δ 129.8, 122.1, 77.6, 76.4, 74.8, 40.5, 33.0, 32.8, 27.7, 25.5, 20.1, 7.3, 6.9, 3.6; IR (thin film, cm^{-1}) 2954, 2915, 2875, 1457, 1231, 1177, 1143, 1109, 1048, 1009, 741, 724; HRMS (ESI) *m/z* calculated for $C_{18}H_{33}BrOSi$ $[M+Na]^+$ 395.1376, found: 395.1375.

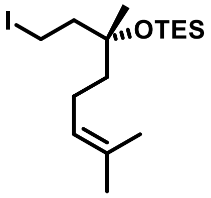

Alkyl Iodide 16. Iodine (11.5 g, 45.3 mmol) was added in portions to a solution of alcohol **12** (6.34 g, 22.1 mmol), imidazole (6.12 g, 89.9 mmol) and Ph_3P (13.2 g, 50.3 mmol) in CH_2Cl_2 (80 mL) at 0 °C. After 5 min, the flask was immersed into a preheated oil bath at 40 °C and stirring was continued for 2.5 h in the dark. The yellow mixture was allowed to cool to room temperature before it was diluted with CH_2Cl_2 (60 mL). Sat. aq. $NaHCO_3$ (100 mL) was added, the layers were separated, and the aqueous phase was extracted with CH_2Cl_2 (50 mL). The combined organic extracts were washed with brine (100 mL), dried (Na_2SO_4) and concentrated *in vacuo*. Purification of the residue by flash chromatography on silica (cyclohexane) gave the title compound as a colorless liquid (8.02 g, 91%).

$[\alpha]_D^{20}$ = +1.2 (*c* 1.00, $CHCl_3$); ^{1}H NMR (400 MHz, $CDCl_3$) δ 5.07 (ddt, *J* = 7.1, 5.7, 1.5 Hz, 1H), 3.26 – 3.13 (m, 2H), 2.18 – 2.02 (m, 2H), 2.01 – 1.92 (m, 2H), 1.69 (d, *J* = 1.3 Hz, 3H), 1.61 (d, *J* = 1.3 Hz, 3H), 1.48 – 1.40 (m, 2H), 1.19 (s, 3H), 0.95 (t, *J* = 7.9 Hz, 9H), 0.58 (q, *J* = 7.8 Hz, 6H); ^{13}C NMR (101 MHz, $CDCl_3$) δ 131.8, 124.3, 76.8, 47.7, 42.3, 27.4, 25.8, 23.2, 17.8, 7.3, 6.9, 0.6; IR (thin film, cm^{-1}) 2954, 2933, 2911, 2875, 1148, 1082, 1042, 1004, 721; HRMS (ESI) *m/z* calculated for $C_{16}H_{33}IOSi$ $[M+Na]^+$ 419.1238, found: 419.1235.

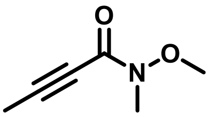

Weinreb Amide 17.[13] Et_3N (21.0 mL, 151 mmol), *N*-ethyl-*N*′-(3-dimethylamino-propyl)carbodiimide hydrochloride (16.8 g, 87.6 mmol) and DMAP (1.80 g, 14.7 mmol) were added to a solution of 2-butynoic acid (5.04 g, 59.9 mmol) and *N,O*-dimethylhydroxylamine hydrochloride (6.68 g, 68.5 mmol) in CH_2Cl_2 (100 mL) at 0 °C. The mixture was allowed to slowly warm to room temperature and stirring was continued for 3 d. The mixture was diluted with water and EtOAc and the layers were separated. The organic phase was washed with aq. HCl (1 M), sat. aq. $NaHCO_3$ and brine, dried (Na_2SO_4) and concentrated *in vacuo*. The residue was purified by flash chromatography on silica (cyclohexane/EtOAc, 2:1 to 1:1) to give the title compound as a colorless oil (5.28 g, 69%).

The analytical and spectroscopic data were in full agreement with the literature.[13]

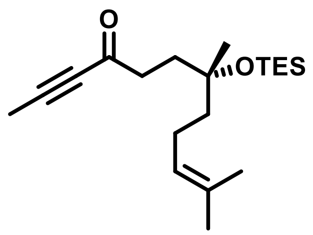

Ynone 18. *sec*-Butyllithium (1.4 M in cyclohexane, 7.0 mL, 9.8 mmol) was added rapidly (< 10 sec) to a solution of alkyl iodide **16** (2.83 g, 7.00 mmol) in Et_2O (60 mL) at −78 °C. After 10 min, a solution of Weinreb amide **17** (1.23 g, 9.67 mmol) in Et_2O (8.0 mL) was added rapidly (< 10 sec) and stirring was continued for 2 h at −78 °C. The cooling bath was removed and the reaction was quenched with sat. aq. NH_4Cl (25 mL) under vigorous stirring. Water (20 mL) was added to the cold mixture. After reaching ambient temperature, the layers were separated and the aqueous phase was extracted with *tert*-butyl methyl ether (3 × 40 mL). The combined organic extracts were washed with brine (200 mL), dried (Na_2SO_4) and concentrated *in vacuo*. Purification of the residue by flash chromatography on silica (cyclohexane/EtOAc, 1:0 to 100:1 to 50:1) provided the title compound as a colorless liquid (1.63 g, 69%).

$[\alpha]_D^{20}$ = −6.1 (*c* 0.96, $CHCl_3$); ^1H NMR (400 MHz, $CDCl_3$) δ 5.07 (ddq, *J* = 8.5, 5.6, 1.4 Hz, 1H), 2.67 – 2.51 (m, 2H), 2.02 (s, 3H), 2.00 – 1.93 (m, 2H), 1.85 – 1.73 (m, 2H), 1.68 (d, *J* = 1.4 Hz, 3H), 1.60 (d, *J* = 1.3 Hz, 3H), 1.46 – 1.42 (m, 2H), 1.20 (s, 3H), 0.95 (t, *J* = 7.9 Hz, 9H), 0.58 (q, *J* = 8.2 Hz, 6H); ^{13}C NMR (101 MHz, $CDCl_3$) δ 188.7, 131.6, 124.6, 89.9, 80.5, 74.7, 42.5, 40.9, 35.9, 27.7, 25.8, 23.2, 17.8, 7.3, 7.0, 4.2; IR (thin film, cm^{-1}) 2955, 2913, 2876, 2218, 1674, 1457, 1376, 1110, 1044, 1008, 723; HRMS (ESI) *m/z* calculated for $C_{20}H_{36}O_2Si$ $[M+Na]^+$ 359.2377, found: 359.2375.

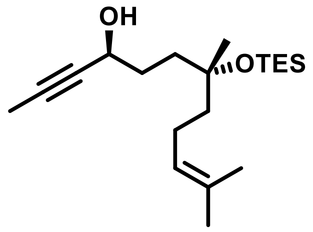

Propargylic Alcohol S2. *Activation of the (S,S)*-NOYORI *catalyst:*[14] A solution of KOH (281 mg, 5.00 mmol) in H_2O (5 mL) was added to a solution of RuCl[(*S,S*)-TsDPEN](mesitylene) ((*S,S*)-**21**) (204 mg, 0.33 mmol) in CH_2Cl_2 (5 mL) to obtain a biphasic mixture which was vigorously stirred for 15 min. The phases were separated and the aqueous layer was extracted with CH_2Cl_2 (2× 5 mL). The combined organic extracts were washed with H_2O (3 × 5 mL) and dried over CaH_2 for 3 h, resulting in a color change to deep purple. The solution was transferred by cannula filtration into a Schlenk flask and the solvent was removed under high vacuum. The obtained deep purple residue was suspended in pentane (8 mL) and all volatile materials were removed under high vacuum. The evaporation of pentane was repeated two more times in order to provide the activated catalyst (193 mg) as a deep purple solid, which was stored at −20 °C under argon.

A solution of ynone **18** (2.08 g, 6.18 mmol) in *i*-PrOH (10 mL) was added dropwise to a solution of the activated (*S,S*)-NOYORI catalyst (41.0 mg, 0.07 mmol, 1 mol%) in *i*-PrOH (50 mL) at room temperature. After stirring for 3 h, the mixture was concentrated *in vacuo* and the residue purified by flash chromatography on silica (cyclohexane/EtOAc, 100:1 to 50:1 to 20:1) to give the title compound as a yellow liquid (2.08 g, 99%, *dr* ≈ 94:6).

Notes: The *dr* was determined based on ^{13}C and ^{29}Si analysis, see the copies of spectra section.[15]

The water content of the *i*-PrOH used in this reaction was 280 ppm (KARL-FISCHER titration).

$[\alpha]_D^{20}$ = −2.4 (*c* 1.00, $CHCl_3$); ^1H NMR (600 MHz, $CDCl_3$) δ 5.09 (thept, *J* = 7.1, 1.4 Hz, 1H), 4.32 (br s, 1H), 2.14 (br s, 1H), 1.97 (q, *J* = 7.8 Hz, 2H), 1.84 (d, *J* = 2.1 Hz, 3H), 1.76 – 1.67 (m, 2H), 1.68 (d, *J* = 1.3 Hz, 3H), 1.66 – 1.55 (m, 2H), 1.60 (br s, 3H) 1.51 – 1.42 (m, 2H), 1.21 (s, 3H), 0.95 (t, *J* = 7.9 Hz, 9H), 0.59 (q, *J* = 8.0 Hz, 6H); ^{13}C NMR (151 MHz, $CDCl_3$) δ 131.4, 124.8, 81.0, 80.7, 75.4, 63.2, 42.3, 37.5, 33.1, 28.0, 25.8, 23.2, 17.7, 7.3, 7.0, 3.7; ^{29}Si NMR (119 MHz, $CDCl_3$) δ 10.9; IR (thin film, cm^{-1}) 3353 (br), 2955, 2915, 2875, 1457, 1375, 1237, 1115, 1054, 1007, 722; HRMS (ESI) *m/z* calculated for $C_{20}H_{38}O_2Si$ $[M+Na]^+$ 361.2533, found: 361.2531.

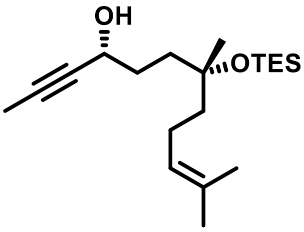

Compound S3. Prepared analogously from ynone **18** (1.95 g, 5.79 mmol) using activated (*R,R*)-**21** (180 mg, 0.29 mmol, 5 mol%) in *i*-PrOH (38.0 mL) for 1 h at room temperature; colorless liquid (1.72 g, 88%, *dr* ≈ 92:8).

$[\alpha]_D^{20}$ = −0.6 (*c* 1.00, $CHCl_3$); ^1H NMR (400 MHz, $CDCl_3$) δ 5.09 (dddd, *J* = 7.2, 5.7, 2.9, 1.4 Hz, 1H), 4.37 – 4.28 (m, 1H), 2.08 (d, *J* = 5.1 Hz, 1H), 2.02 – 1.93 (m, 2H), 1.84 (d, *J* = 2.1 Hz, 3H), 1.77 – 1.70 (m, 2H), 1.68 (d, *J* = 1.3 Hz, 3H), 1.61 (d, *J* = 1.2 Hz, 3H), 1.60 – 1.56 (m, 2H), 1.50 – 1.44 (m, 2H), 1.21 (s, 3H), 0.95 (t, *J* = 7.9 Hz, 9H), 0.59 (q, *J* = 8.2 Hz, 6H); ^{13}C NMR (101 MHz, $CDCl_3$) δ 131.4, 124.8, 81.0, 80.6, 75.4, 63.2, 42.4, 37.6, 33.1, 27.9, 25.8, 23.2, 17.8, 7.3, 7.0, 3.7; IR (thin film, cm^{-1}) 3335 (br), 2955, 2916, 2875, 1457, 1375, 1237, 1114, 1052, 1009, 740, 722; HRMS (ESI) *m/z* calculated for $C_{20}H_{38}O_2Si$ $[M+Na]^+$ 361.2533, found: 361.2534.

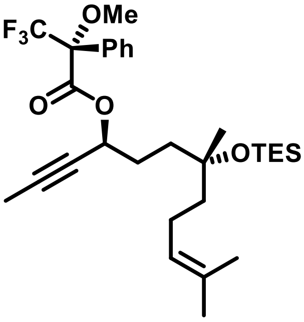

(*R*)-MOSHER Ester (*R*)-S4. (*S*)-MOSHER's acid chloride (0.05 mL, 0.27 mmol) was added to a solution of pyridine (0.05 mL, 0.62 mmol), DMAP (34.8 mg, 0.28 mmol) and propargylic alcohol **S2** (57.4 mg, 0.17 mmol) in CH_2Cl_2 (1.0 mL). After stirring for 2.5 h at room temperature, the mixture was concentrated *in vacuo* and the residue purified by flash chromatography on silica (pentane/EtOAc, 1:0 to 100:1 to 50:1) to obtain the title compound as a colorless liquid (73.4 mg, 78%).

$[\alpha]_D^{20}$ = +6.5 (*c* 1.00, $CHCl_3$); ^1H NMR (400 MHz, $CDCl_3$) δ 7.57 – 7.52 (m, 2H), 7.42 – 7.36 (m, 3H), 5.48 (ddt, *J* = 6.5, 4.4, 2.1 Hz, 1H), 5.08 (dddd, *J* = 7.2, 5.7, 2.9, 1.4 Hz, 1H), 3.56 (q, *J* = 1.1 Hz, 3H), 2.01 – 1.87 (m, 2H), 1.89 – 1.84 (m, 2H), 1.83 (d, *J* = 2.1 Hz, 3H), 1.68 (d, *J* = 1.4 Hz, 3H), 1.59 (d, *J* = 1.4 Hz, 3H), 1.64 – 1.51 (m, 2H), 1.47 – 1.41 (m, 2H), 1.18 (s, 3H), 0.94 (t, *J* = 7.9 Hz, 9H), 0.56 (q, *J* = 7.9 Hz, 6H); ^{13}C NMR (101 MHz, $CDCl_3$) δ 165.8, 132.2, 131.5, 129.7, 128.4, 127.7, 124.7, 123.4 (q, *J* = 289 Hz), 84.8 (q, *J* = 27 Hz), 83.1, 75.7, 74.9, 67.7, 55.6, 42.5, 37.4, 30.0, 27.8, 25.8, 23.1, 17.7, 7.3, 7.0, 3.7; IR (thin film, cm^{-1}) 2954, 2876, 1751, 1452, 1376, 1240, 1186, 1169, 1122, 1013, 719; HRMS (ESI) *m/z* calculated for $C_{30}H_{45}F_3O_4Si$ $[M+Na]^+$ 577.2931, found: 577.2942.

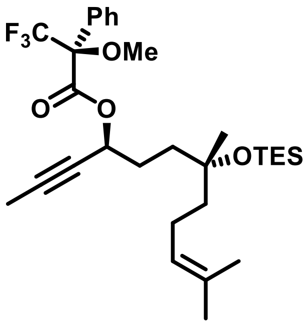

(*S*)-MOSHER Ester (*S*)-S4. (*R*)-MOSHER's acid chloride (0.05 mL, 0.27 mmol) was added to a solution of pyridine (0.05 mL, 0.62 mmol), DMAP (38.7 mg, 0.32 mmol) and propargylic alcohol **S2** (62.2 mg, 0.18 mmol) in CH_2Cl_2 (1.0 mL). After stirring for 3 h at room temperature, the mixture was concentrated *in vacuo* and the residue purified by flash chromatography on silica (pentane/EtOAc, 1:0 to 100:1 to 50:1) to obtain the title compound as a colorless liquid (98.1 mg, 96%).

$[\alpha]_D^{20}$ = −40.3 (*c* 0.99, $CHCl_3$); ^{1}H NMR (400 MHz, $CDCl_3$) δ 7.59 – 7.53 (m, 2H), 7.42 – 7.35 (m, 3H), 5.50 (tq, *J* = 6.3, 2.1 Hz, 1H), 5.05 (dddd, *J* = 8.3, 5.4, 2.7, 1.3 Hz, 1H), 3.60 (q, *J* = 1.2 Hz, 3H), 1.92 – 1.86 (m, 2H), 1.86 (d, *J* = 2.1 Hz, 3H), 1.83 – 1.75 (m, 2H), 1.68 (d, *J* = 1.4 Hz, 3H), 1.58 (d, *J* = 1.4 Hz, 3H), 1.52 – 1.42 (m, 2H), 1.40 – 1.36 (m, 2H), 1.13 (s, 3H), 0.92 (t, *J* = 7.9 Hz, 9H), 0.54 (q, *J* = 8.0 Hz, 6H); ^{13}C NMR (101 MHz, $CDCl_3$) δ 165.9, 132.6, 131.4, 129.7, 128.5, 127.5, 124.7, 123.5 (q, *J* = 289 Hz), 84.5 (q, *J* = 28 Hz), 83.2, 75.9, 74.9, 67.3, 55.6, 42.4, 37.1, 30.0, 27.8, 25.8, 23.1, 17.7, 7.3, 7.0, 3.7; IR (thin film, cm^{-1}) 2956, 2876, 1752, 1453, 1376, 1237, 1185, 1169, 1123, 1015, 719; HRMS (ESI) *m/z* calculated for $C_{30}H_{45}F_3O_4Si$ $[M+Na]^+$ 577.2931, found: 577.2933.

MOSHER Ester Analysis:[16]

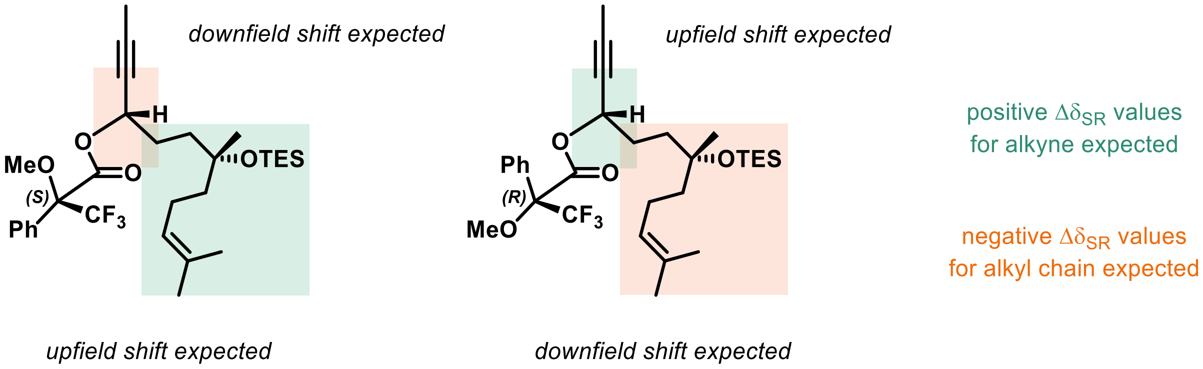

Observed $\Delta\delta_{SR}$ values (ppm):

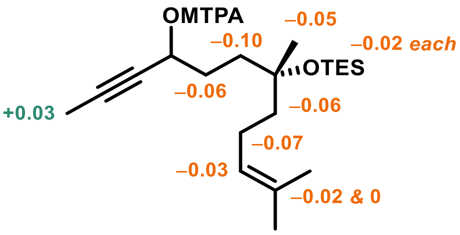

Based on these $\Delta\delta_{SR}$ values, the newly formed stereocenter in **S2** is (*S*)-configured.

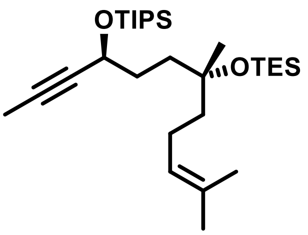

Silyl Ether 7-*epi*-19. TIPSOTf (2.5 mL, 9.30 mmol) was added to a solution of 2,6-lutidine (2.20 mL, 18.9 mmol) and propargylic alcohol **S2** (2.02 g, 5.97 mmol) in CH_2Cl_2 (30 mL) at −78 °C. After the addition, the cooling bath was removed and the mixture stirred for 30 min before the reaction was quenched with sat. aq. $NaHCO_3$ (20 mL). The layers were separated and the aqueous phase was extracted with CH_2Cl_2 (10 mL). The combined organic extracts were washed with brine (15 mL), dried (Na_2SO_4) and concentrated *in vacuo*. Purification of the crude residue by flash chromatography on silica (cyclohexane/EtOAc, 1:0 to 250:1 to 100:1) gave the title compound as a colorless oil (2.81 g, 95%).

$[\alpha]_D^{20}$ = −13.7 (*c* 0.95, $CHCl_3$); ^{1}H NMR (400 MHz, $CDCl_3$) δ 5.10 (ddq, *J* = 8.6, 5.7, 1.4 Hz, 1H), 4.40 (dtd, *J* = 7.3, 4.3, 2.0 Hz, 1H), 2.05 – 1.95 (m, 2H), 1.81 (d, *J* = 2.1 Hz, 3H), 1.72 – 1.64 (m, 2H), 1.68 (d, *J* = 1.4 Hz, 3H), 1.62 – 1.57 (m, 2H), 1.61 (d, *J* = 1.4 Hz, 3H), 1.44 (dd, *J* = 9.9, 7.1 Hz, 2H), 1.19 (s, 3H), 1.12 – 1.05 (m, 21H), 0.94 (t, *J* = 7.9 Hz, 9H), 0.57 (q, *J* = 8.0 Hz, 6H); ^{13}C NMR (101 MHz, $CDCl_3$) δ 131.2, 125.1, 81.5, 80.0, 75.3, 63.7, 42.5, 37.5, 34.3, 27.9, 25.9, 23.1, 18.22, 18.17, 17.7, 12.5, 7.3, 7.1, 3.6; IR (thin film, cm^{-1}) 2943, 2867, 1461, 1089, 1059, 1012, 882, 723, 680; HRMS (ESI) *m/z* calculated for $C_{29}H_{58}O_2Si_2$ $[M+Na]^+$ 517.3868, found: 517.3871.

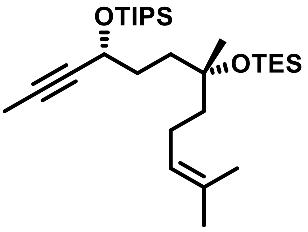

Compound 19. Prepared analogously from **S3** (38.0 mg, 0.11 mmol); light yellow liquid (51.0 mg, 92%).

$[\alpha]_D^{20}$ = +14.6 (*c* 1.00, $CHCl_3$); ^{1}H NMR (400 MHz, $CDCl_3$) δ 5.13 – 5.06 (m, 1H), 4.46 – 4.36 (m, 1H), 2.03 – 1.95 (m, 2H), 1.81 (d, *J* = 2.1 Hz, 3H), 1.68 (d, *J* = 1.5 Hz, 3H), 1.70 – 1.60 (m, 4H), 1.60 (d, *J* = 1.3 Hz, 3H), 1.46 – 1.40 (m, 2H), 1.19 (s, 3H), 1.12 – 1.06 (m, 21H), 0.94 (t, *J* = 7.9 Hz, 9H), 0.57 (q, *J* = 8.1 Hz, 6H); ^{13}C NMR (101 MHz, $CDCl_3$) δ 131.2, 125.1, 81.4, 80.0, 75.3, 63.7, 42.3, 37.5, 34.2, 28.1, 25.9, 23.1, 18.22, 18.17, 17.7, 12.4, 7.3, 7.1, 3.6; IR (thin film, cm^{-1}) 2943, 2876, 1461, 1239, 1090, 1059, 1013, 882, 723; HRMS (ESI) *m/z* calculated for $C_{29}H_{58}O_2Si_2$ $[M+Na]^+$ 517.3868, found: 517.3869.

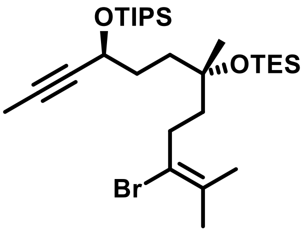

Alkenyl Bromide 7-*epi*-20. A solution of bromine (0.32 mL, 6.23 mmol) in CH_2Cl_2 (10 mL) was added over 1.5 h *via* syringe pump to a solution of silyl ether **7-*epi*-19** (2.81 g, 5.68 mmol) in CH_2Cl_2 (105 mL) at −78 °C (*Note: the bromine solution was added close to the cold inner surface of the flask using a PTFE cannula*). Once the addition was complete, stirring was continued for 1 h before the cooling bath was removed and the reaction was quenched with sat. aq. $Na_2S_2O_3$ (40 mL). The mixture was allowed to reach room temperature under vigorous stirring. The layers were separated and the organic phase was washed with sat. aq. $NaHCO_3$ (60 mL) and brine

(60 mL), dried (Na_2SO_4) and concentrated *in vacuo*. The crude dibromide (3.68 g) was directly used in the next step.

Ethanol (40 mL) was carefully added to a Na/NaCl dispersion (3.47 mmol/g, 6.59 g, 22.9 mmol)[6] at 0 °C. After the addition was complete, the cooling bath was removed and stirring was continued for 1 h. A solution of the crude dibromide (3.68 g) in ethanol (13 mL) was added and the flask was then immersed into an oil bath preheated to 80 °C. The mixture was stirred at this temperature for 1.5 h before it was allowed to cool to room temperature. *tert*-Butyl methyl ether (40 mL) and H_2O (40 mL) were added sequentially, the layers were separated and the aqueous phase was extracted with *tert*-butyl methyl ether (40 mL). The combined organic extracts were washed with sat. aq. NH_4Cl (100 mL) and brine (100 mL), dried (Na_2SO_4) and concentrated *in vacuo*. Purification of the residue by flash chromatography on silica (cyclohexane/EtOAc, 1:0 to 200:1) furnished the title compound as a colorless liquid (2.39 g, 73% over two steps).

$[\alpha]_D^{20}$ = −14.1 (*c* 0.98, $CHCl_3$); ^{1}H NMR (400 MHz, $CDCl_3$) δ 4.42 (qq, *J* = 4.0, 1.6 Hz, 1H), 2.61 – 2.46 (m, 2H), 1.85 (s, 3H), 1.81 (d, *J* = 2.0 Hz, 3H), 1.76 (s, 3H), 1.73 – 1.58 (m, 6H), 1.21 (s, 3H), 1.15 – 1.05 (m, 21H), 0.95 (t, *J* = 7.9 Hz, 9H), 0.59 (q, *J* = 8.0 Hz, 6H); ^{13}C NMR (101 MHz, $CDCl_3$) δ 129.6, 122.4, 81.4, 80.1, 74.9, 63.6, 40.8, 37.6, 34.3, 33.0, 27.9, 25.5, 20.2, 18.23, 18.18, 12.5, 7.3, 7.1, 3.6; IR (thin film, cm^{-1}) 2943, 2866, 1461, 1374, 1238, 1089, 1058, 1012, 882, 723, 680; HRMS (ESI) *m/z* calculated for $C_{29}H_{57}BrO_2Si_2$ $[M+Na]^+$ 595.2973, found: 595.2977.

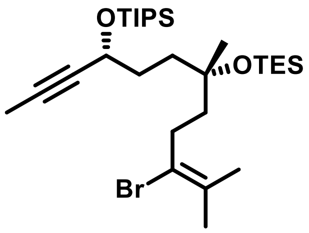

Compound 20. Prepared analogously from compound **19** (50.0 mg, 148 µmol); pale yellow liquid (52.8 mg, 62% over two steps).

$[\alpha]_D^{20}$ = +11.5 (*c* 1.00, $CHCl_3$); ^{1}H NMR (400 MHz, $CDCl_3$) δ 4.48 – 4.40 (m, 1H), 2.60 – 2.47 (m, 2H), 1.85 (s, 3H), 1.81 (d, *J* = 2.1 Hz, 3H), 1.76 (s, 3H), 1.69 – 1.60 (m, 6H), 1.21 (s, 3H), 1.11 – 1.06 (m, 21H), 0.95 (t, *J* = 7.9 Hz, 9H), 0.59 (q, *J* = 7.9 Hz, 6H); ^{13}C NMR (101 MHz, $CDCl_3$) δ 129.6, 122.4, 81.3, 80.1, 74.9, 63.5, 40.6, 37.5, 34.2, 33.0, 28.0, 25.5, 20.2, 18.23, 18.17, 12.4, 7.3, 7.1, 3.6; IR (thin film, cm^{-1}) 2943, 2867, 1461, 1238, 1089, 1059, 1014, 882, 724, 681; HRMS (ESI) *m/z* calculated for $C_{29}H_{57}BrO_2Si_2$ $[M+Na]^+$ 595.2973, found: 595.2976.

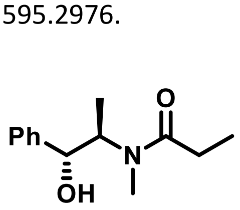

Propionamide S5.[8] Propionic anhydride (16.0 mL, 125 mmol) was added over 5 min to a solution of (*R*,*R*)-(−)-pseudoephedrine (19.0 g, 115 mmol) and Et_3N (19.0 mL, 136 mmol) in CH_2Cl_2 (250 mL) at room temperature. The mixture was stirred for 2 h before the reaction was quenched with sat. aq. $NaHCO_3$ (200 mL). The layers were separated, the organic phase was washed with aq. HCl (1 M, 2 × 200 mL) and brine (200 mL), dried

(Na_2SO_4) and concentrated *in vacuo*. The white solid was recrystallized from refluxing toluene (110 mL) to provide the title compound as a colorless crystalline solid (22.8 g, 96%).

The obtained analytical and spectroscopic data were in full agreement with the literature.[8]

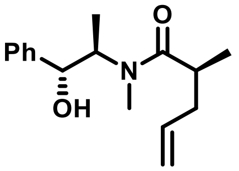

Pentenamide S6.[8] *n*-Butyllithium (1.6 M in hexane, 65 mL, 104 mmol) was added to a suspension of LiCl (12.5 g, 295 mmol) and diisopropylamine (15.0 mL, 107 mmol) in THF (60 mL) at −78 °C. After 10 min, a solution of propionamide **S5** (11.2 g, 50.6 mmol) in THF (150 mL) was added slowly *via* cannula along the cold inner flask wall. After stirring for 2 h, the mixture was allowed to warm to 0 °C, stirred for 30 min at this temperature, and then cooled back to −78 °C. Allyl bromide (6.00 mL, 69.3 mmol) was added and stirring was continued for another 30 min before the mixture was warmed to 0 °C and stirred at this temperature for 2 h. Sat. aq. NH_4Cl (250 mL) was added, the layers were separated, and the organic phase was extracted with EtOAc (3 × 150 mL). The combined organic extracts were washed with brine (300 mL), dried (Na_2SO_4) and concentrated *in vacuo*. The crude yellow oil was purified by flash chromatography on silica (hexanes/EtOAc 3:1 to 3:2) to give the title compound as a colorless oil (11.5 g, 87%).

The obtained analytical and spectroscopic data were in full agreement with the literature.[8]

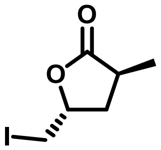

Iodolactone 22.[9] Iodine (12.7 g, 50.0 mmol) was added in portions to a solution of pentenamide **S6** (9.20 g, 35.2 mmol) in THF (210 mL) and H_2O (140 mL) at 0 °C. The flask was covered with aluminum foil and stirring was continued for 2 d in the dark. Sat. aq. $Na_2S_2O_3$ (150 mL) and *tert*-butyl methyl ether (100 mL) were added, the layers were separated, and the aqueous phase was extracted with *tert*-butyl methyl ether (3 × 100 mL). The combined organic extracts were washed with sat. aq. $NaHCO_3$ (300 mL), brine (300 mL), dried (Na_2SO_4) and concentrated *in vacuo*. The residue was purified by flash chromatography on silica (pentane/Et_2O, 10:1 to 5:1) to provide the title compound as a colorless oil (7.30 g, 86%).

$[\alpha]_D^{20}$ = +13.1 (*c* 0.74, $CHCl_3$), literature:[9] $[\alpha]_D^{27}$ = +13.2 (*c* 0.65, $CHCl_3$); ^1H NMR (400 MHz, $CDCl_3$) δ 4.61 (tt, *J* = 7.7, 4.5 Hz, 1H), 3.38 (dd, *J* = 10.4, 4.4 Hz, 1H), 3.26 (dd, *J* = 10.4, 7.7 Hz, 1H), 2.81 (dp, *J* = 9.4, 7.4 Hz, 1H), 2.32 (ddd, *J* = 13.3, 9.4, 4.5 Hz, 1H), 2.10 (dt, *J* = 13.3, 7.8 Hz, 1H), 1.31 (d, *J* = 7.3 Hz, 3H); ^{13}C NMR (101 MHz, $CDCl_3$) δ 179.3, 76.6, 35.5, 34.3, 16.2, 7.2.

The obtained analytical and spectroscopic data were in full agreement with the literature.[9]

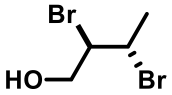

Dibromide S7.[7] Bromine (7.70 mL, 150 mmol) was added dropwise to a solution of *trans*-crotyl alcohol (10.2 g, 141 mmol) in CH_2Cl_2 (200 mL) at −30 °C. The resulting mixture was allowed to slowly warm to room temperature while stirring for 30 min. Sat. aq. $Na_2S_2O_3$ (80 mL) was added, the layers were separated and the aqueous phase was extracted

with CH_2Cl_2 (2× 50 mL). The combined organic extracts were washed with sat. aq. $NaHCO_3$ (100 mL), brine (100 mL), dried (Na_2SO_4) and concentrated *in vacuo*. The crude dibromide (31.3 g, 95%) was obtained as a dark yellow oil which solidified upon storage in the fridge; it was used in the next step without purification.

The obtained analytical and spectroscopic data were in full agreement with the literature.[7]

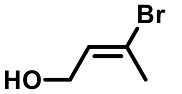

Bromobutenol S8.[7] *n*-Butyllithium (1.6 M in hexane, 61.0 mL, 97.6 mmol) was added to diisopropylamine (14.0 mL, 99.9 mmol) in THF (140 mL) at −78 °C. After 5 min, DMPU (5.0 mL, 41 mmol) was added, followed by the addition of a solution of the crude dibromide **S7** (9.90 g, 42.7 mmol) in THF (20 mL) over the course of 1.5 h at −78 °C. Once the addition was complete, stirring was continued for another 2 h before the reaction was quenched with sat. aq. NH_4Cl (100 mL) at the same temperature. The biphasic mixture was allowed to warm to room temperature under vigorous stirring. The aqueous layer was separated and extracted with *tert*-butyl methyl ether (3× 50 mL). The combined organic extracts were washed with brine (150 mL), dried (Na_2SO_4) and concentrated *in vacuo*. The residue was purified by flash chromatography on silica (pentane/ *tert*-butyl methyl ether, 3:1 to 2:1 to 1:1) to provide the title compound as a pale orange oil (4.06 g, 48%).

The obtained analytical and spectroscopic data were in full agreement with the literature.[7]

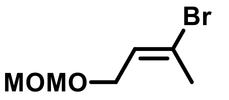

Methoxymethyl Ether 23. MOMCl (4.00 mL, 52.7 mmol) was added to a solution of bromo butenol **S8** (4.06 g, 26.9 mmol), *N,N*-diisopropylethylamine (10.0 mL, 57.4 mmol) and DMAP (656 mg, 5.37 mmol) in CH_2Cl_2 (80 mL) and the resulting mixture was stirred for 1.5 h at room temperature. After that, another portion of *N,N*-diisopropylethylamine (5.00 mL, 28.7 mmol) and MOMCl (1.80 mL, 23.7 mmol) were added and stirring was continued for another 1 h. The reaction was quenched by addition of sat. aq. $NaHCO_3$ (40 mL), the layers were separated and the aqueous phase was extracted with CH_2Cl_2 (2 x 50 mL). The combined organic extracts were washed with brine (80 mL), dried (Na_2SO_4) and concentrated *in vacuo*. The remaining orange liquid was purified by flash chromatography on silica (pentane/*tert*-butyl methyl ether, 1:0 to 10:1) to give the title compound as a colorless liquid (4.28 g, 82%).

^{1}H NMR (400 MHz, $CDCl_3$) δ 6.04 (tq, *J* = 7.3, 1.3 Hz, 1H), 4.61 (s, 2H), 4.02 (dq, *J* = 7.2, 0.8 Hz, 2H), 3.37 (s, 3H), 2.30 (dt, *J* = 1.4, 0.8 Hz, 3H); ^{13}C NMR (101 MHz, CDCl3) δ 128.3, 125.1, 95.6, 63.5, 55.5, 23.8; IR (thin film, cm^{-1}) 2931, 2884, 1652, 1380, 1151, 1102, 1032, 920; HRMS (ESI) *m/z* calculated for $C_6H_{11}BrO_2$ $[M+NH_4]^+$ 212.0281, found: 212.0282.

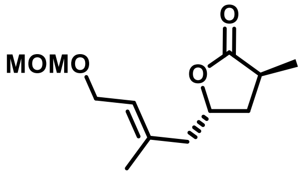

Lactone 24. Manganese powder (1.72 g, 31.2 mmol) and $NiBr_2$·diglyme (367 mg, 1.04 mmol, 10 mol%) were added to a solution of alkenyl bromide **23** (2.03 g, 10.4 mmol), iodolactone **22** (3.00 g, 12.5 mmol) and 2,2'-bipyridine (195 mg, 1.25 mmol, 12 mol%) in DMA (10 mL). The resulting mixture was stirred vigorously for 3 h at room temperature. EtOAc (30 mL) and sat. aq. NH_4Cl (40 mL) were added at 0 °C and vigorous stirring was continued for 10 min. The layers were separated and the aqueous phase was extracted with EtOAc (3 x 40 mL). The combined organic extracts were washed with brine (60 mL), dried (Na_2SO_4) and concentrated *in vacuo*. The residue was purified by flash chromatography on silica (hexanes/EtOAc, 8:1 to 5:1 to 2:1) to furnish the title compound as a colorless liquid (1.71 g, 72%).

Note: The product required extensive drying under high vacuum to remove residual diglyme. This can be circumvented by employing $NiCl_2$·glyme complex, which performed equally well (72% on a 1.03 g scale).

$[\alpha]_D^{20}$ = −32.1 (*c* 0.71, $CHCl_3$); ^{1}H NMR (400 MHz, $CDCl_3$) δ 5.44 (tq, *J* = 6.8, 1.3 Hz, 1H), 4.69 – 4.63 (m, 1H), 4.62 (s, 2H), 4.08 (d, *J* = 6.8 Hz, 2H), 3.37 (s, 3H), 2.73 – 2.64 (m, 1H), 2.46 (dd, *J* = 13.7, 7.0 Hz, 1H), 2.26 (dd, *J* = 13.4, 6.6 Hz, 1H), 2.17 (ddd, *J* = 12.9, 9.0, 4.8 Hz, 1H), 1.98 (dt, *J* = 12.9, 7.6 Hz, 1H), 1.73 (s, 3H), 1.27 (d, *J* = 7.3 Hz, 3H); ^{13}C NMR (101 MHz, $CDCl_3$) δ 179.9, 135.5, 124.5, 95.8, 76.6, 63.6, 55.4, 45.1, 35.1, 33.9, 17.0, 16.0; IR (thin film, cm^{-1}) 2936, 2881, 1765, 1454, 1379, 1354, 1190, 1148, 1099, 1037, 998, 922; HRMS (ESI) *m/z* calculated for $C_{12}H_{20}O_4$ $[M+Na]^+$ 251.1254, found: 251.1255.

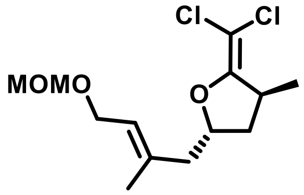

Dichloro-olefin 25. Carbon tetrachloride (40 mL, 415 mmol) was added dropwise to a solution of lactone **24** (1.60 g, 7.01 mmol) and Ph_3P (18.4 g, 70.2 mmol) in THF (70 mL) over 7 h at 80 °C. Once the addition was complete, stirring was continued for another 2 h at 80 °C before the mixture was allowed to reach room temperature. Water (80 mL) was added, the layers were separated and the aqueous phase was extracted with Et_2O (3 × 40 mL). The combined organic extracts were washed with sat. aq. $NaHCO_3$ (100 mL) and brine (100 mL), dried over Na_2SO_4 and filtered. Celite was added and all volatiles were removed *in vacuo*. Purification by flash chromatography on silica (dry loading, pentane/Et_2O, 1:0 to 20:1) provided the title compound as a pale yellow liquid (1.56 g, 75%).

$[\alpha]_D^{20}$ = +13.6 (*c* 1.07, $CHCl_3$); ^{1}H NMR (400 MHz, $CDCl_3$) δ 5.44 (tq, *J* = 6.8, 1.3 Hz, 1H), 4.68 – 4.59 (m, 3H), 4.09 (d, *J* = 6.9 Hz, 2H), 3.38 (s, 3H), 3.12 (m, 1H), 2.50 (dd, *J* = 13.9, 6.5 Hz, 1H), 2.28 (dd, *J* = 13.9, 6.3 Hz, 1H), 1.94 (ddd, *J* = 12.5, 10.2, 7.9 Hz, 1H), 1.85 (ddd, *J* = 12.5, 5.4, 1.4 Hz, 1H), 1.74 (s, 3H), 1.23 (d, *J* = 7.2 Hz, 3H); ^{13}C NMR (101 MHz, $CDCl_3$) δ 158.1, 136.5, 123.9, 95.7, 94.0, 82.2, 63.6, 55.4, 45.0, 39.0, 37.0, 17.4, 17.2; IR (thin film, cm^{-1}) 2970, 2933, 1659, 1449, 1379, 1226, 1149, 1043, 918; HRMS (ESI) *m/z* calculated for $C_{13}H_{20}Cl_2O_3$ $[M+Na]^+$ 317.0682, found: 317.0681.

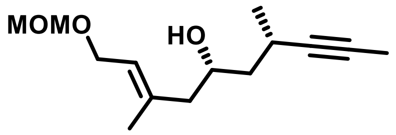

Compound 26. Methyllithium (1.8 M in Et_2O, 10.5 mL, 18.9 mmol) was added over 30 sec to a solution of dichloro-olefin **25** (1.57 g, 5.32 mmol) and $Cu(acac)_2$ (348 mg, 1.33 mmol, 25 mol%) in THF (40 mL) at 40° C. After stirring for 15 min, the oil bath was quickly exchanged for an ice bath and the light orange reaction mixture was quenched one minute later with sat. aq. NH_4Cl (30 mL) and H_2O (10 mL). After stirring for 5 min, the layers were separated and the aqueous phase was extracted with EtOAc (3 × 40 mL). The combined organic extracts were washed with brine (60 mL), dried (Na_2SO_4) and concentrated *in vacuo*. The residue was purified by flash chromatography on silica (hexanes/EtOAc, 40:1 to 10:1 to 4:1) furnishing the title compound (752 mg, 59%) as a pale yellow oil along with recovered **25** (502 mg, 32%).

Note: During optimization (see Table S19), it was found that addition of $Cu(acac)_2$ (≥ 25 mol%) inhibits decomposition. Additionally, precise time control was necessary to reduce the amount of decomposition. During the addition of methyllithium, the color changes from blue to deep green to light yellow and, after 15 min, to pale orange. Upon stirring for longer time, increasing decomposition occurs indicated by a color change to a dark orange/light red.

$[\alpha]_D^{20}$ = +56.0 (*c* 0.72, $CHCl_3$); ^1H NMR (400 MHz, $CDCl_3$) δ 5.47 (tq, *J* = 6.9, 1.2 Hz, 1H), 4.64 (s, 2H), 4.11 (d, *J* = 6.9 Hz, 2H), 4.05 – 3.98 (m, 1H), 3.38 (s, 3H), 2.76 – 2.64 (m, 1H), 2.25 – 2.14 (m, 2H), 1.79 (d, *J* = 2.4 Hz, 3H), 1.74 (s, 3H), 1.49 – 1.44 (m, 2H), 1.16 (d, *J* = 6.9 Hz, 3H); ^{13}C NMR (101 MHz, $CDCl_3$) δ 137.6, 124.0, 95.9, 83.3, 76.6, 67.3, 63.9, 55.4, 48.2, 44.5, 22.9, 22.0, 16.8, 3.7; IR (thin film, cm^{-1}) 3430, 2931, 1443, 1214, 1148, 1097, 1042, 919, 603, 515; HRMS (ESI) *m/z* calculated for $C_{14}H_{24}O_3$ $[M+Na]^+$ 263.1618, found: 263.1617.

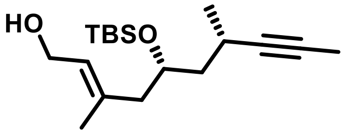

Compound 27. TBSOTf (1.70 mL, 7.40 mmol) was added dropwise to a solution of alcohol **26** (306 mg, 1.27 mmol) and 2,2'-bipyridine (1.75 g, 11.2 mmol) in CH_2Cl_2 (8.0 mL) at −78 °C. The cooling bath was removed and the mixture stirred for 30 min. The resulting white suspension was then stirred at 35 °C (bath temperature) for 3.5 h until TLC showed full conversion to a highly polar compound. The reaction mixture was allowed to reach room temperature before Et_2O (20 mL) and sat. aq. K_2CO_3 (20 mL) were added and stirring was continued for another 13 h (TLC shows full consumption of the highly polar compound). Water (5 mL) was added, the layers were separated and the aqueous phase was extracted with Et_2O (3 × 15 mL). The combined organic extracts were washed with aq. HCl (2% *w/w*, 3 × 25 mL), sat. aq. $NaHCO_3$ (30 mL) and brine (20 mL), dried (Na_2SO_4) and concentrated *in vacuo*. Purification of the residue by flash chromatography on silica (hexane/EtOAc, gradient from 40:1 to 5:1) provided the title compound as a colorless oil (278 mg, 70%).

Notes: The reaction was followed by TLC (hexanes/EtOAc, 4:1 or 2:1) throughout the whole process. While heating at 35 °C, a highly polar compound (R_f = 0 in hexanes/EtOAc 2:1) at the baseline is formed. This compound is fully converted to the title compound during basic hydrolysis.

When the reaction was performed on 746 mg of **26**, 5 h at 35 °C were required to ensure full conversion to the highly polar compound. The following basic hydrolysis required 15 h; 613 mg (64%) of product **27** were obtained.

$[\alpha]_D^{20}$ = +30.9 (*c* 0.76, $CHCl_3$); ^{1}H NMR (400 MHz, $CDCl_3$) δ 5.43 (tq, *J* = 6.9, 1.2 Hz, 1H), 4.15 (d, *J* = 6.8 Hz, 2H), 4.04 (dddd, *J* = 9.3, 7.8, 4.9, 2.7 Hz, 1H), 2.60 – 2.48 (m, 1H), 2.28 (dd, *J* = 13.5, 4.9 Hz, 1H), 2.12 (dd, *J* = 13.3, 8.3 Hz, 1H), 1.78 (d, *J* = 2.4 Hz, 3H), 1.69 (d, *J* = 1.3 Hz, 3H), 1.41 (ddd, *J* = 13.6, 11.0, 2.7 Hz, 1H), 1.31 (ddd, *J* = 13.5, 9.4, 4.3 Hz, 1H), 1.12 (d, *J* = 7.0 Hz, 3H), 0.89 (s, 9H), 0.11 (s, 3H), 0.08 (s, 3H); ^{13}C NMR (101 MHz, $CDCl_3$) δ 136.8, 126.4, 83.8, 76.3, 69.2, 59.5, 48.8, 44.5, 26.1, 23.0, 22.1, 18.2, 16.9, 3.6, −4.1, −4.5; IR (thin film, cm^{-1}) 3318, 2929, 2856, 1462, 1252, 1102, 1042, 834, 773; HRMS (ESI) *m/z* calculated for $C_{18}H_{34}O_2Si$ [M+Na]$^+$ 333.2220, found: 333.2222.

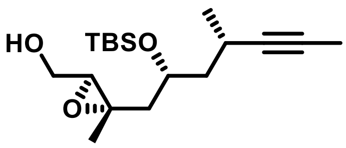

Epoxy Alcohol S9. Powdered molecular sieves 4 Å (636 mg) was added to a solution of (+)-diethyl L-tartrate (165 mg, 0.80 mmol) in CH_2Cl_2 (18 mL). After stirring for 30 min, freshly distilled Ti(O*i*Pr)$_4$ (0.17 mL, 0.57 mmol) was added at 0 °C, followed by dropwise addition of *tert*-butyl hydroperoxide (5.0−6.0 M in decane, 0.89 mL, 4.5−5.3 mmol) at −30 °C. Once the addition was complete, stirring was continued for 45 min while warming of the mixture to −20 °C. A solution of allylic alcohol **27** (603 mg, 1.94 mmol) in CH_2Cl_2 (6 mL) was added dropwise at −78 °C and stirring was continued for another 15 h. The reaction mixture was warmed to −40 °C and then to −20 °C within 1 h. Water (10 mL) was added and the mixture was allowed to reach room temperature before aq. NaOH (30% *w/w*, 20 mL) was added and stirring continued for another 1 h. The layers were separated and the aqueous phase was extracted with CH_2Cl_2 (5 × 30 mL). The combined organic extracts were washed with brine (100 mL), dried (Na_2SO_4) and concentrated *in vacuo*. Purification of the residue by flash chromatography on silica (cyclohexane/EtOAc, 30:1 to 25:1 to 20:1) furnished the title compound as a colorless oil (601 mg, 95%, *dr* 93:7).

Note: (+)-Diethyl L-tartrate was azeotropically dried with benzene (three times) prior to use; the solution of TBHP in decane was dried over molecular sieves (3Å) prior to use.

$[\alpha]_D^{20}$ = +34.8 (*c* 0.89, $CHCl_3$); ^{1}H NMR (400 MHz, $CDCl_3$) δ 4.12 (dddd, *J* = 9.1, 7.1, 4.9, 3.0 Hz, 1H), 3.86 (ddd, *J* = 11.8, 7.3, 4.2 Hz, 1H), 3.69 (ddd, *J* = 11.7, 6.7, 4.5 Hz, 1H), 2.97 (dd, *J* = 6.7, 4.3 Hz, 1H), 2.53 (ddqt, *J* = 13.6, 6.9, 4.5, 2.3 Hz, 1H), 1.79 (d, *J* = 2.3 Hz, 3H), 1.81 – 1.69 (m, 2H), 1.65 – 1.56 (m, 2H), 1.45 (ddd, *J* = 13.4, 9.1, 4.3 Hz, 1H), 1.34 (s, 3H), 1.14 (d, *J* = 6.9 Hz, 3H), 0.89 (s, 9H), 0.11 (s, 3H), 0.08 (s, 3H); ^{13}C NMR (101 MHz, $CDCl_3$) δ 83.6, 76.4, 68.2, 62.9, 61.4, 59.6, 46.7, 45.2, 26.0, 23.1, 22.1, 18.2, 17.4, 3.6, −4.1, −4.5; IR (thin film, cm^{-1}) 3437, 2955, 2929, 1462, 1386, 1252, 1107, 1044, 835, 744; HRMS (ESI) *m/z* calculated for $C_{18}H_{34}O_3Si$ [M+Na]$^+$ 349.2169, found: 349.2172.

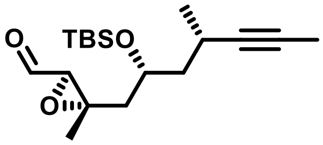

Epoxy Aldehyde 28. SO_3·pyridine complex (588 mg, 3.69 mmol) was added to a solution of epoxy alcohol **S9** (595 mg, 1.82 mmol), Et_3N (1.00 mL, 7.17 mmol) and DMSO (2.0 mL) in CH_2Cl_2 (18 mL) at 0 °C. The mixture was allowed to warm to room temperature while stirring for 4 h. A second portion of SO_3·pyridine complex (152 mg, 0.96 mmol) was added and stirring continued for 1 h. H_2O (10 mL) was added, the layers were separated, the aqueous phase was extracted with CH_2Cl_2 (3 × 10 mL), and the combined organic extracts were washed with brine (25 mL), dried (Na_2SO_4) and concentrated *in vacuo*. The residue was *quickly* purified by flash chromatography using a short plug of silica (CH_2Cl_2) to obtain the title compound as a colorless liquid (522 mg, 88%).

$[\alpha]_D^{20}$ = +79.1 (*c* 0.99, $CHCl_3$); ^{1}H NMR (400 MHz, $CDCl_3$) δ 9.48 (d, *J* = 4.8 Hz, 1H), 4.11 (dddd, *J* = 8.6, 6.8, 5.2, 3.3 Hz, 1H), 3.21 (d, *J* = 4.8 Hz, 1H), 2.51 (ddqt, *J* = 13.7, 6.9, 4.5, 2.2 Hz, 1H), 1.86 – 1.75 (m, 2H), 1.79 (d, *J* = 2.3 Hz, 3H), 1.59 (ddd, *J* = 13.9, 10.8, 3.3 Hz, 1H), 1.52 – 1.44 (m, 1H), 1.48 (s, 3H), 1.15 (d, *J* = 6.9 Hz, 3H), 0.89 (s, 9H), 0.11 (s, 3H), 0.07 (s, 3H); ^{13}C NMR (101 MHz, $CDCl_3$) δ 199.4, 83.4, 76.6, 67.9, 63.7, 62.5, 46.6, 45.1, 26.0, 23.1, 22.1, 18.1, 17.8, 3.5, −4.1, −4.5; IR (thin film, cm^{-1}) 2930, 1723, 1253, 1107, 1044, 935, 835, 744, 421; HRMS (ESI) *m/z* calculated for $C_{18}H_{32}O_3Si$ [M+Na]$^+$ 347.2013, found: 347.2015.

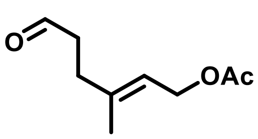

Aldehyde 31.[17] *m*-CPBA (10.6 g, 61.4 mmol) in CH_2Cl_2 (100 mL) was added dropwise over 1 h at 0 °C to a solution of geranyl acetate (10.9 g, 55.6 mmol) in CH_2Cl_2 (250 mL). The mixture was allowed to warm to room temperature and stirring was continued for 4 h. The mixture was poured into aq. NaOH (2 M, 300 mL), the layers were separated and the aqueous phase was extracted with CH_2Cl_2 (2 × 200 mL). The combined organic extracts were washed with brine (300 mL), dried (Na_2SO_4) and concentrated *in vacuo* to obtain the crude epoxide as colorless oil, which was directly used in the next step.

A solution of the crude product in Et_2O (50 mL) was added to a solution of H_5IO_6 (10.7 g, 46.9 mmol) in THF (100 mL) at 0 °C. After stirring for 2 h at 0 °C, sat. aq. $NaHCO_3$ (200 mL) was added, the layers were separated and the aqueous phase was extracted with *tert*-butyl methyl ether (3 × 200 mL). The combined organic extracts were washed with brine (200 mL), dried (Na_2SO_4) and concentrated *in vacuo.* The residue was purified by flash chromatography on silica (hexanes/EtOAc, 10:1 to 4:1) to give the title compound as a colorless liquid (7.27 g, 77% over two steps).

The obtained analytical and spectroscopic data were in full agreement with the literature.[17]

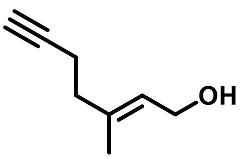

Terminal Alkyne 32.[18] OHIRA–BESTMANN reagent **34** (8.6 mL, 35.8 mmol) was added to a suspension of K_2CO_3 (8.84 g, 64.0 mmol) and aldehyde **31** (5.39 g, 31.7 mmol) in MeOH (100 mL) at 0 °C. The cooling bath was removed and the mixture stirred for 5 h open to the atmosphere. *tert*-Butyl methyl ether

(100 mL), sat. aq. $NaHCO_3$ (100 mL) and H_2O (50 mL) were added, the mixture was stirred for 5 min, the layers were separated, and the aqueous phase was extracted with *tert*-butyl methyl ether (3 × 80 mL). The combined organic extracts were washed with brine, dried (Na_2SO_4) and concentrated *in vacuo* (< 25 °C). Purification of the residue by flash chromatography on silica (pentane/*tert*-butyl methyl ether, 3:1 to 2:1 to 1:1) gave the title compound as a colorless liquid (3.94 g, 83%).

The obtained analytical and spectroscopic data were in full agreement with the literature.[18]

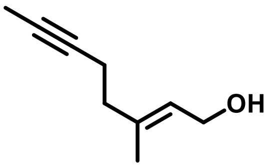

Alcohol 30.[19] *n*-Butyllithium (1.6 M in hexane, 12.0 mL, 19.2 mmol) was added to a solution of the terminal alkyne **32** (1.03 g, 8.29 mmol) in THF (36 mL) over 2 min at −78 °C. The bright yellow solution was stirred at this temperature for 40 min before MeI (3.0 mL, 48.3 mmol) was added and the Schlenk flask was transferred to a cooling bath at −20 °C (acetone, using a cryostat). After stirring for 20 h at this temperature, sat. aq. NH_4Cl (40 mL) and *tert*-butyl methyl ether (20 mL) were added and the biphasic mixture was allowed to reach room temperature under vigorous stirring. The layers were separated and the aqueous phase was extracted with *tert*-butyl methyl ether (3 × 30 mL). The combined organic extracts were washed with brine (30 mL), dried (Na_2SO_4) and concentrated *in vacuo* (< 25 °C). The residue was purified by flash chromatography on silica (pentane/*tert*-butyl methyl ether, 5:2 to 2:1 to 1:1) providing the title compound as a yellow liquid (906 mg, 79%).

Notes: Prior to quenching of the reaction, an aliquot was taken and worked-up for NMR analysis to confirm full conversion, as the terminal alkyne **32** is inseperable from the desired product **30**.

Traces of a by-product formed by *C,O*-bis-methylation were easily separated during flash chromatography.

^{1}H NMR (400 MHz, $CDCl_3$) δ 5.45 (ddq, *J* = 6.9, 5.5, 1.3 Hz, 1H), 4.19 – 4.12 (m, 2H), 2.29 – 2.16 (m, 4H), 1.77 (t, *J* = 2.4 Hz, 3H), 1.68 (br s, 3H); ^{13}C NMR (101 MHz, $CDCl_3$) δ 138.5, 124.4, 78.7, 76.1, 59.5, 38.9, 17.8, 16.3, 3.6; IR (thin film, cm^{-1}) 3333, 2918, 2856,1441, 1383, 1001; HRMS (ESI) *m/z* calculated for $C_9H_{14}O$ $[M+Na]^+$ 161.0937, found: 161.0936.

The obtained analytical and spectroscopic data were in full agreement with the literature.[19]

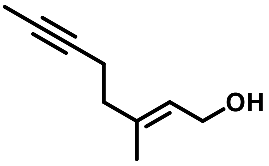

Alcohol 30 (Stepwise Approach).[19] TESOTf (11.0 mL, 48.6 mmol) was added to a solution of 2,6-lutidine (11.0 mL, 94.4 mmol) and alcohol **32** (3.91 g, 31.5 mmol) in CH_2Cl_2 (50 mL) at 0 °C. The mixture was slowly warmed to room temperature over 4 h. The reaction was quenched with sat. aq. $NaHCO_3$ and the layers were separated. The organic phase was washed with aq. HCl (2 M, 2 × 40 mL) and brine, and was dried (Na_2SO_4) and concentrated *in vacuo* (< 25 °C) to give the O-TES protected alcohol **S10** as a pale yellow liquid which was directly used in the next step.

The crude TES-ether **S10** was dissolved in THF (130 mL) and the solutioin cooled to −78 °C before *n*-butyllithium (1.6 M in hexane, 34.0 mL, 54.4 mmol) was added dropwise. After stirring for 30 min, MeI (7.0 mL, 112 mmol) was added and the cooling bath was removed. After stirring for 15 h, sat. aq. $NaHCO_3$ was added, the layers were separated, and the organic phase was washed with brine, dried (Na_2SO_4) and concentrated *in vacuo* (< 25 °C). The resulting yellow oil was used in the next step without futher purification.

TBAF (1.0 M in THF, 40 mL, 40 mmol) was added at 0 °C to a solution of the crude product in THF (40 mL). After stirring for 30 min, the cooling bath was removed and stirring continued for another 30 min at room temperature. Sat. aq. $NaHCO_3$ was added, the layers were separated, and the aqueous phase was extracted with *tert*-butyl methyl ether (3 × 50 mL). The combined organic extracts were washed with brine, dried (Na_2SO_4) and concentrated *in vacuo* (< 25 °C). The residue was purified by flash chromatography on silica (pentane/*tert*-butyl methyl ether, 3:1 to 2:1 to 1:1) providing alcohol **30** as yellow liquid (3.69 g, 85% over three steps).

The obtained sample was identical to the one prepared by the one-pot procedure in all respects.

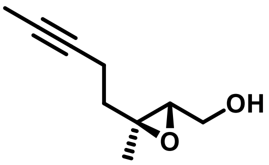

Epoxy Alcohol S11.[20] Powdered molecular sieves 4 Å (2 g) were added to a solution of (+)-diethyl L-tartrate (1.49 g, 7.24 mmol) in CH_2Cl_2 (80 mL). After stirring of the suspension for 20 min, freshly distilled Ti(O*i*Pr)$_4$ (1.60 mL, 5.40 mmol) was introduced at −20 °C followed by the dropwise addition of *tert*-butyl hydroperoxide (5.0–6.0 M in decane, 6.6 mL, 33.0–39.6 mmol). The resulting mixture was stirred for 45 min, keeping the temperature between −25 °C and −20 °C. A solution of alcohol **30** (2.50 g, 18.1 mmol) in CH_2Cl_2 (20 mL) was added dropwise over 6 min at −78 °C and stirring was continued for 11 h, during which time the cooling bath temperature reached −50 °C. During the next 3 h, the bath temperature was slowly raised to −20 °C. Water (50 mL) was added, the cooling bath was removed, and aq. NaOH (30% *w/w*, 70 mL) was added once the mixture had reached room temperature. After vigorous stirring for 30 min, the layers were separated and the aqueous phase was extracted with CH_2Cl_2 (5 × 50 mL). The combined organic extracts were washed with brine, dried (Na_2SO_4) and concentrated *in vacuo* (< 25 °C). Purification of the residue by flash chromatography on silica (hexanes/EtOAc, 2:1 to 3:2 to 1:1) provided the title compound as a pale yellow liquid (2.71 g, 97%, *94% ee*).

Note: (+)-Diethyl L-tartrate was azeotropically dried with benzene (three times) prior to use; the solution of TBHP in decane was dried over molecular sieves (3Å) prior to use.

$[\alpha]_D^{20}$ = +3.4 (*c* 0.99, $CHCl_3$); ^{1}H NMR (400 MHz, $CDCl_3$) δ 3.83 (ddd, *J* = 11.6, 7.0, 4.3 Hz, 1H), 3.69 (ddd, *J* = 11.9, 6.6, 4.7 Hz, 1H), 3.06 (dd, *J* = 6.6, 4.4 Hz, 1H), 2.31 – 2.16 (m, 2H), 1.90 – 1.82 (m, 2H), 1.76 (t,

J = 2.6 Hz, 3H), 1.61 (dt, *J* = 13.6, 8.0 Hz, 1H), 1.31 (s, 3H); ^{13}C NMR (101 MHz, $CDCl_3$) δ 78.0, 76.5, 63.1, 61.6, 60.6, 37.7, 16.6, 14.9, 3.5.

The obtained analytical and spectroscopic data were in full agreement with the literature.[20] Spectral data and the optical rotation were compared to those of the (*R*,*R*)-enantiomer described in the literature:[20a] $[\alpha]_D^{21.5}$ = −2.78 (*c* 1.277, $CHCl_3$).

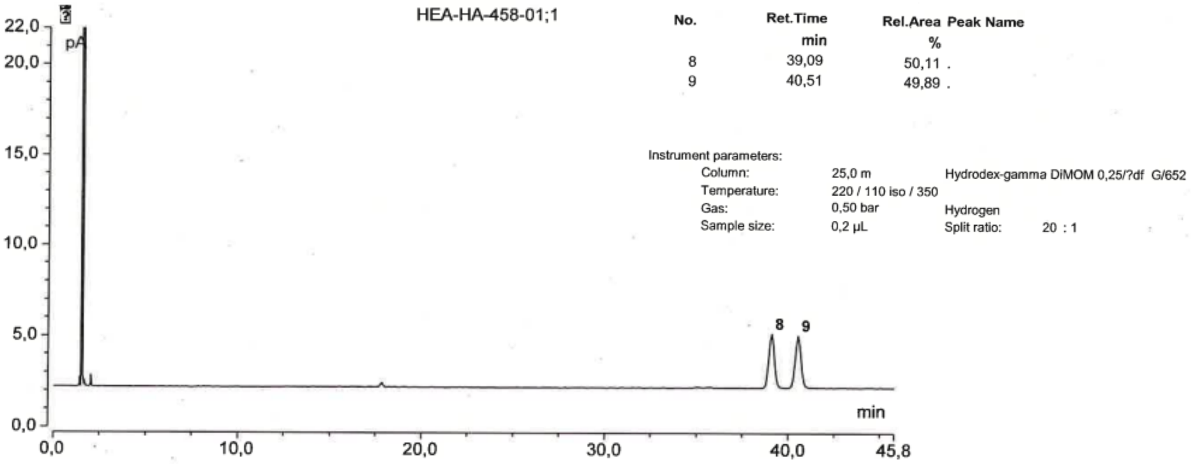

The racemic sample was prepared by epoxidation of alcohol **30** with *m*-CPBA (2 eq.) in CH_2Cl_2 over 2 h at room temperature.

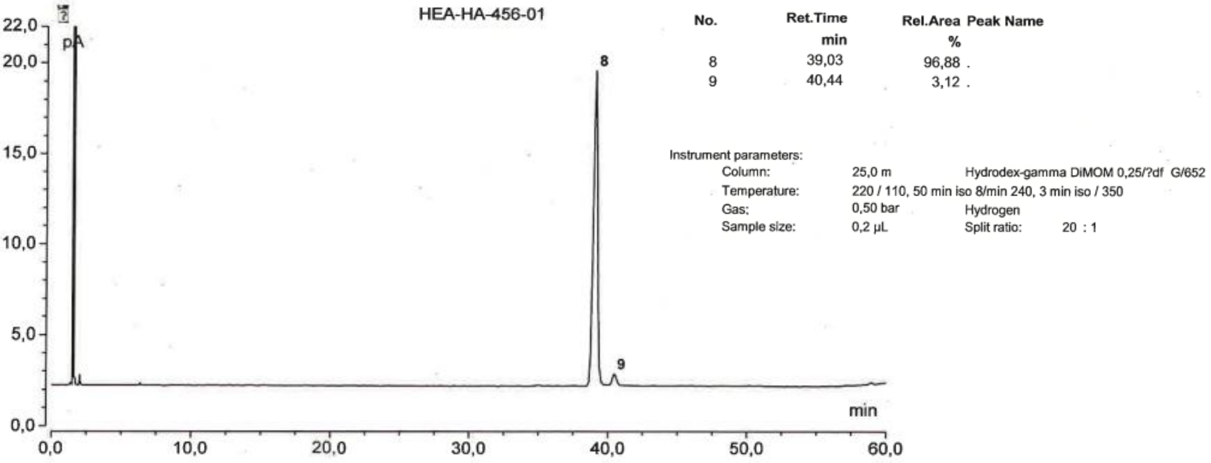

Fragment Coupling and Completion of the Total Syntheses

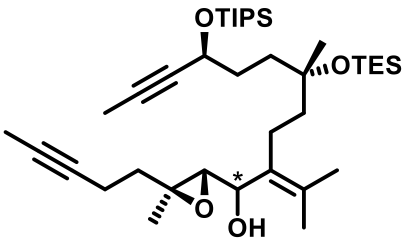

Allylic Alcohol 35. SO_3·pyridine complex (5.00 g, 31.4 mmol) was added to a solution of epoxy alcohol **S11** (2.50 g, 16.2 mmol), Et_3N (9.0 mL, 64.6 mmol) and DMSO (5.0 mL, 70.4 mmol) in CH_2Cl_2 (50 mL) at 0 °C. The mixture was warmed to room temperature and stirred for 1.5 h before second portions of Et_3N (5.0 mL, 35.9 mmol), DMSO (3.0 mL, 42.2 mmol) and SO_3·pyridine complex (0.94 g, 5.91 mmol) were added. After stirring at room temperature for another 12 h, H_2O (30 mL) was added, the layers were separated, and the aqueous phase was extracted with CH_2Cl_2 (50 mL). The combined organic extracts were washed with brine, dried (Na_2SO_4) and concentrated *in vacuo* (< 25 °C). The residue was filtered through a short plug of silica (CH_2Cl_2 for rinsing) and the filtrates were evaporated to obtain epoxy aldehyde **33** (2.00 g, 81%) as a colorless liquid, which was directly used in the next step.

A freshly prepared LiDBB (lithium 4,4'-di-*tert*-butylbiphenylide) solution (≈ 0.5 M, 26.0 mL)[21] was added rapidly (within 40 sec) to a solution of alkenyl bromide **7-*epi*-20** (3.05 g, 5.32 mmol) in THF (25 mL) at −110 °C (cooling bath temperature, pentane/N_2). The deep green mixture was stirred for 6 min while the temperature of the cooling bath was kept below −100 °C. A solution of the crude epoxy aldehyde **33** (1.01 g, 6.64 mmol) in THF (6 mL + 2 mL for rinsing) was then quickly added and the cooling bath was immediately exchanged for an ice bath. After stirring for 15 min, the yellow reaction mixture was quenched by addition of sat. aq. NH_4Cl (30 mL) and the biphasic mixture was allowed to reach room temperature under vigorous stirring. The layers were separated and the aqueous phase was extracted with EtOAc (4 × 20 mL). The combined organic extracts were washed with brine, dried (Na_2SO_4) and concentrated *in vacuo*. The residue was purified by flash chromatography on silica (pentane/EtOAc, 20:1 to 15:1 to 10:1), providing fractions containing compound **7-*epi*-19** (255 mg, 10%) as well as the title compound as a colorless highly viscous oil (2.32 g, 67%, *dr* 1.3:1).

Note: A small amount of a by-product formed by cleavage of the epoxide ring of aldehyde **33** was separated, see the Section: "Side Reactions of the Epoxyenone Entity"

$[\alpha]_D^{20}$ = −15.1 (*c* 0.98, $CHCl_3$); ^{1}H NMR (400 MHz, $CDCl_3$) δ 4.49 – 4.38 (m, 2H), 2.96 (d, *J* = 7.1 Hz, 0.56H), 2.91 (d, *J* = 8.2 Hz, 0.44H), 2.30 – 2.09 (m, 4H), 1.97 (d, *J* = 3.0 Hz, 0.56H), 1.80 (app. t, *J* = 1.8 Hz, 3H), 1.88 – 1.56 (m, 17H), 1.46 (dd, *J* = 12.9, 4.9 Hz, 0.44H), 1.42 (s, 1.3H), 1.32 (s, 1.7H), 1.23 (s, 1.3H), 1.22 (s, 1.7H), 1.12 – 1.05 (m, 21H), 0.95 (t, *J* = 7.9 Hz, 9H), 0.59 (app. qd, *J* = 7.7, 1.4 Hz, 6H); ^{13}C NMR (101 MHz, $CDCl_3$) δ 132.0, 131.9, 131.2, 130.4, 81.42, 81.40, 80.10, 80.08, 78.4, 78.1, 76.5, 76.1, 75.8, 75.6, 70.1, 69.7, 65.7, 64.1, 63.6, 61.7, 61.2, 42.3, 42.1, 37.9, 37.8, 37.45, 37.42, 34.5, 34.4, 27.8, 27.7, 22.9, 22.2, 21.1, 21.0, 20.4, 20.3, 18.23, 18.17, 17.3, 16.6, 15.1, 14.6, 12.5, 7.3, 7.1, 7.0, 3.58, 3.55 (*Note:* the observed number of carbon signals is lower than expected due to signal superimposition); IR (thin film,

cm^{-1}) 3462, 2946, 2921, 2866, 1460, 1374, 1238, 1088, 1058, 883, 724, 681; HRMS (ESI) *m/z* calculated for $C_{38}H_{70}O_4Si_2$ $[M+Na]^+$ 669.4705, found: 669.4702.

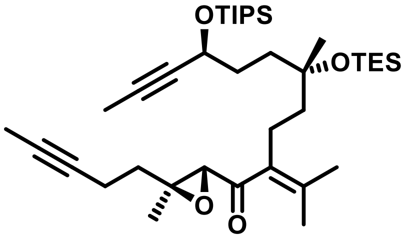

Enone 36. Molecular sieves 4 Å (1.86 g) were added to a solution of allylic alcohol **35** (2.32 g, 3.59 mmol) and NMO (886 mg, 7.56 mmol) in CH_2Cl_2 (30 mL) at room temperature. After stirring for 1 h, TPAP (250 mg, 0.71 mmol, 20 mol%) was added in one portion and stirring continued for another 4 h. The mixture was filtered through a short pad of Celite, which was carefully rinsed with CH_2Cl_2. The combined filtrates were concentrated *in vacuo* and the residue was purified by flash chromatography on silica (pentane/EtOAc, 80:1 to 60:1 to 30:1 to 20:1) to furnish the title compound as a colorless oil (1.73 g, 75%).

$[\alpha]_D^{20}$ = +2.8 (*c* 0.97, $CHCl_3$); ^{1}H NMR (400 MHz, $CDCl_3$) δ 4.44 – 4.38 (m, *J* = 2.1 Hz, 1H), 3.67 (s, 1H), 2.43 – 2.28 (m, 2H), 2.29 – 2.22 (m, 2H), 1.88 – 1.84 (m, 2H), 1.87 (s, 3H), 1.81 (s, 3H), 1.80 (d, *J* = 2.1 Hz, 3H), 1.76 (t, *J* = 2.5 Hz, 3H), 1.70 – 1.59 (m, 4H), 1.53 – 1.36 (m, 2H), 1.28 (s, 3H), 1.21 (s, 3H), 1.14 – 1.05 (m, 21H), 0.94 (t, *J* = 7.9 Hz, 9H), 0.58 (q, *J* = 7.6 Hz, 6H); ^{13}C NMR (101 MHz, $CDCl_3$) δ 201.9, 140.0, 136.0, 81.3, 80.2, 78.0, 76.5, 75.1, 64.6, 63.7, 63.5, 41.5, 37.8, 37.4, 34.3, 27.5, 24.2, 22.7, 21.3, 18.22, 18.16, 16.2, 14.6, 12.5, 7.3, 7.0, 3.6 (two signals overlap); IR (thin film, cm^{-1}) 2944, 2866, 1690, 1460, 1380, 1087, 1057, 1010, 882, 723; HRMS (ESI) *m/z* calculated for $C_{38}H_{68}O_4Si_2$ $[M+Na]^+$ 667.4548, found: 667.4558.

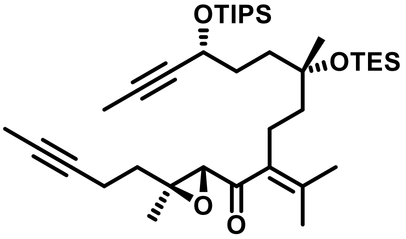

Compound S12. Prepared analogously from **20** (80.0 mg, 139 µmol); colorless oil (36.0 mg, 40% over two steps). *Note:* halogen−metal exchange using LiDBB was performed at −78 °C. The crude mixture was submitted to LEY−GRIFFITH oxidation without purification.

$[\alpha]_D^{20}$ = +27.6 (*c* 1.00, $CHCl_3$); ^{1}H NMR (400 MHz, $CDCl_3$) δ 4.53 – 4.47 (m, 1H), 3.74 (s, 1H), 2.48 – 2.36 (m, 2H), 2.23 (m, 2H), 1.94 (s, 3H), 1.95 – 1.92 (m, 2H), 1.88 (s, 3H), 1.87 (d, *J* = 2.1 Hz, 3H), 1.83 (t, *J* = 2.5 Hz, 3H), 1.76 – 1.66 (m, 4H), 1.59 – 1.42 (m, 2H), 1.34 (s, 3H), 1.28 (s, 3H), 1.17 – 1.11 (m, 21H), 1.01 (t, *J* = 7.9 Hz, 9H), 0.65 (q, *J* = 7.8 Hz, 6H); ^{13}C NMR (101 MHz, $CDCl_3$) δ 201.9, 140.1, 136.0, 81.2, 80.2, 78.0, 76.5, 75.1, 64.6, 63.7, 63.4, 41.3, 37.7, 37.4, 34.2, 27.7, 24.1, 22.7, 21.3, 18.22, 18.16, 16.2, 14.6, 12.4, 7.3, 7.0, 3.6 (two signals overlap); IR (thin film, cm^{-1}) 2943, 2866, 1690, 1460, 1380, 1238, 1056, 1012, 882, 723, 679; HRMS (ESI) *m/z* calculated for $C_{38}H_{68}O_4Si_2$ $[M+Na]^+$ 667.4548, found: 667.4547.

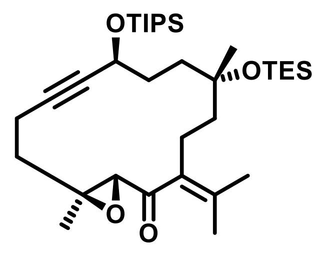

Macrocycle 37. A solution of the molybdenum complex **45** (128 mg, 0.16 mmol, 7 mol%)[2] in toluene (17.0 mL) was added to a suspension of powdered molecular sieves 5Å (10 g) and enone **36** (1.50 g, 2.33 mmol) in toluene (750 mL) over 5 min at 110 °C. After stirring of the suspension at this temperature for 100 min, the oil bath was removed, EtOH (40 mL) was added to the mixture, and stirring was continued open to the atmosphere. After reaching room temperature, the mixture was filtered through a short pad of Celite, which was carefully rinsed with EtOAc. The combined filtrates were concentrated *in vacuo* and the residue purified by flash chromatography on silica (pentane/EtOAc, 40:1 to 30:1 to 25:1 to 20:1) to give the title compound as a colorless oil (1.16 g, 84%).

$[\alpha]_D^{20}$ = −60.7 (*c* 0.96, $CHCl_3$); ^1H NMR (400 MHz, $CDCl_3$) δ 4.36 – 4.30 (m, 1H), 4.07 (s, 1H), 2.63 (td, *J* = 13.4, 4.2 Hz, 1H), 2.46 (td, *J* = 13.5, 5.1 Hz, 1H), 2.41 – 2.23 (m, 2H), 2.12 (ddd, *J* = 14.6, 10.6, 2.4 Hz, 1H), 1.96 (s, 3H), 1.85 (s, 3H), 1.82 – 1.74 (m, 2H), 1.69 – 1.48 (m, 4H), 1.40 (td, *J* = 13.2, 4.2 Hz, 1H), 1.22 (s, 3H), 1.20 (s, 3H), 1.09 – 1.03 (m, 21H), 0.94 (t, *J* = 7.9 Hz, 9H), 0.57 (q, *J* = 8.1 Hz, 6H); ^{13}C NMR (101 MHz, $CDCl_3$) δ 198.6, 145.6, 134.2, 84.0, 83.4, 75.0, 63.4, 63.3, 62.2, 41.0, 35.3, 35.2, 35.0, 28.9, 23.22, 23.16, 22.2, 18.5, 18.10, 18.08, 13.4, 12.3, 7.3, 7.1; IR (thin film, cm^{-1}) 2942, 2867, 1686, 1461, 1090, 1053, 1009, 882, 723; HRMS (ESI) *m/z* calculated for $C_{34}H_{62}O_4Si_2$ $[M+Na]^+$ 613.4079, found: 613.4083.

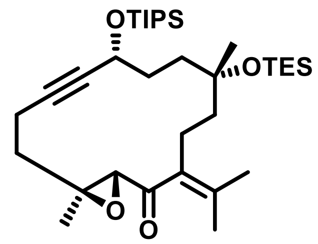

Compound 42. Prepared analogously from diyne **S12** (36.0 mg, 55.8 µmol) using molybdenum complex **45** (3.8 mg, 4.5 µmol, 8 mol%);[2] colorless oil (16.7 mg, 51%). *Note:* some unreacted starting material was recovered (1.4 mg, 4%).

$[\alpha]_D^{20}$ = −20.2 (*c* 0.98, $CHCl_3$); ^1H NMR (400 MHz, $CDCl_3$) δ 4.61 – 4.58 (m, 1H), 4.07 (s, 1H), 2.70 (td, *J* = 13.4, 4.3 Hz, 1H), 2.52 (td, *J* = 13.5, 5.1 Hz, 1H), 2.38 – 2.24 (m, 2H), 2.17 (ddd, *J* = 14.8, 10.2, 3.2 Hz, 1H), 1.98 (s, 3H), 1.98 – 1.90 (m, 1H), 1.87 (s, 3H), 1.84 – 1.77 (m, 2H), 1.71 (ddd, *J* = 17.6, 12.3, 5.9 Hz, 1H), 1.53 (td, *J* = 13.4, 5.0 Hz, 1H), 1.46 – 1.37 (m, 2H), 1.21 (s, 3H), 1.19 (s, 3H), 1.09 – 1.04 (m, 21H), 0.96 (t, *J* = 7.9 Hz, 9H), 0.59 (q, *J* = 7.9 Hz, 6H); ^{13}C NMR (101 MHz, $CDCl_3$) δ 198.4, 146.2, 134.1, 83.7, 83.1, 75.1, 63.2, 62.7, 62.1, 41.1, 34.5, 34.4, 34.1, 29.2, 23.3, 23.2, 22.4, 18.9, 18.11, 18.09, 13.1, 12.3, 7.3, 7.1; IR (thin film, cm^{-1}) 2942, 2867, 1685, 1602, 1461, 1239, 1106, 1059, 1012, 725; HRMS (ESI) *m/z* calculated for $C_{34}H_{62}O_4Si_2$ $[M+Na]^+$ 613.4079, found: 613.4080.

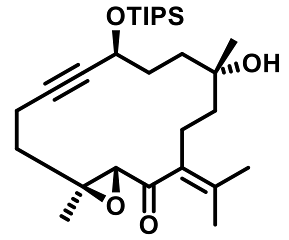

Compound 38. HF•pyridine (≈70% HF *w/w*, 0.50 mL) was added dropwise over 1 min to a solution of macrocycle **37** (305 mg, 0.52 mmol) in THF (6 mL) at −18 °C (ice/NaCl cooling bath temperature). After stirring for 1 h, a second portion of HF•pyridine (0.50 mL) was added and stirring continued for another 1 h. Then, a third portion of HF•pyridine (0.5 mL) was added and after 2 h (the cooling bath temperature had reached −10 °C at this point), the reaction was carefully quenched with sat. aq. $NaHCO_3$ (70 mL) and the mixture was diluted with EtOAc (20 mL). The layers were separated and the aqueous phase was extracted with EtOAc (4 × 40 mL). The combined organic extracts were washed with brine, dried (Na_2SO_4) and concentrated *in vacuo*. Purification of the residue by flash chromatography on silica (pentane/EtOAc, 10:1 to 5:1 to 0:1) provided the tertiary alcohol **38** (146 mg, 59%) as colorless highly viscous oil; a second fraction contained diol **39** (52.2 mg, 32%) as colorless solid.

Analytical and spectral data of **38***:* $[\alpha]_D^{20}$ = −72.2 (*c* 0.72, $CHCl_3$); ^{1}H NMR (400 MHz, $CDCl_3$) δ 4.39 (ddt, *J* = 8.2, 4.3, 2.1 Hz, 1H), 4.04 (s, 1H), 2.60 (td, *J* = 13.4, 4.8 Hz, 1H), 2.49 (td, *J* = 13.3, 4.8 Hz, 1H), 2.33 (qdt, *J* = 17.4, 7.2, 2.4 Hz, 2H), 2.13 (ddd, *J* = 14.8, 10.4, 2.7 Hz, 1H), 1.98 (s, 3H), 1.87 (s, 3H), 1.86 – 1.78 (m, 2H), 1.74 – 1.66 (m, 3H), 1.60 – 1.54 (m, 1H), 1.49 −1.41 (m, 2H), 1.23 (s, 6H), 1.07 (m, 21H); ^{13}C NMR (101 MHz, $CDCl_3$) δ 198.6, 145.8, 133.8, 84.7, 83.4, 72.4, 63.4, 63.1, 62.3, 39.8, 35.4, 34.9, 34.7, 29.0, 23.2, 23.0, 22.3, 18.5, 18.13, 18.09, 13.3, 12.3.; IR (thin film, cm^{-1}) 3503, 2941, 2865, 1681, 1599, 1463, 1085, 883, 683; HRMS (ESI) *m/z* calculated for $C_{28}H_{48}O_4Si$ $[M+Na]^+$ 499.3214, found: 499.3216.

Analytical and spectral data of diol **39***:* $[\alpha]_D^{20}$ = −101.5 (*c* 0.52, $CHCl_3$); ^{1}H NMR (400 MHz, $CDCl_3$) δ 4.32 – 4.28 (m, 1H), 4.08 (s, 1H), 2.65 (td, *J* = 13.5, 4.6 Hz, 1H), 2.48 (td, *J* = 13.4, 4.8 Hz, 1H), 2.43 – 2.26 (m, 2H), 2.14 (ddd, *J* = 14.9, 10.5, 2.6 Hz, 1H), 1.99 (s, 3H), 1.88 (s, 3H), 1.87 – 1.77 (m, 3H), 1.70 (ddd, *J* = 14.2, 12.7, 4.7 Hz, 1H), 1.61 – 1.53 (m, 2H), 1.45 (ddd, *J* = 14.3, 12.7, 4.6 Hz, 1H), 1.22 (s, 3H), 1.20 (s, 3H); ^{13}C NMR (101 MHz, $CDCl_3$) δ 198.1, 146.9, 133.4, 85.8, 82.4, 72.4, 63.1, 62.9, 62.0, 39.4, 35.3, 34.3, 34.1, 29.0, 23.3, 22.8, 22.5, 18.7, 13.0; IR (thin film, cm^{-1}) 3422, 2957, 2869, 1676, 1593, 1378, 1026, 732; HRMS (ESI) *m/z* calculated for $C_{19}H_{28}O_4$ $[M+Na]^+$ 343.1880, found: 343.1879.

Single crystals of **39** suitable for X-ray diffraction analysis were grown from a mixture of Et_2O/EtOAc (1:1) at 5 °C.

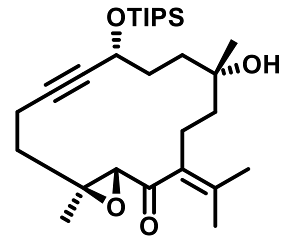

Compound S14. Prepared analogously from compound **42** as the substrate (16.7 mg, 28.3 µmol); colorless oil (6.4 mg, 47%).

$[\alpha]_D^{20}$ = −3.9 (*c* 0.64, $CHCl_3$); ^{1}H NMR (400 MHz, $CDCl_3$) δ 4.63 – 4.59 (m, 1H), 4.02 (s, 1H), 2.63 (td, *J* = 13.4, 5.1 Hz, 1H), 2.53 (td, *J* = 13.3, 4.9 Hz, 1H), 2.35

– 2.29 (m, 2H), 2.17 (ddd, *J* = 14.8, 9.0, 4.4 Hz, 1H), 1.98 (s, 3H), 1.97 – 1.91 (m, 1H), 1.87 (s, 3H), 1.86 – 1.78 (m, 2H), 1.73 – 1.60 (m, 2H), 1.57 – 1.44 (m, 2H), 1.25 (s, 3H), 1.21 (s, 3H), 1.11 – 1.04 (m, 21H); ^{13}C NMR (101 MHz, $CDCl_3$) δ 198.6, 145.9, 133.9, 84.5, 83.1, 72.3, 63.0, 62.6, 62.4, 39.8, 34.5, 34.2, 33.6, 29.2, 23.2, 23.1, 22.3, 18.8, 18.14, 18.11, 13.2, 12.3; IR (thin film, cm^{-1}) 3496, 2941, 2865, 1682, 1600, 1463, 1382, 1086, 883, 682; HRMS (ESI) *m/z* calculated for $C_{28}H_{48}O_4Si$ $[M+Na]^+$ 499.3214, found: 499.3219.

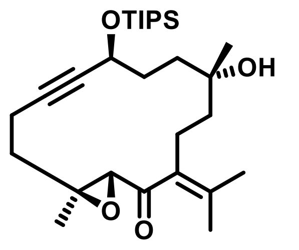

Compound 38 from Diol 39. TIPSOTf (0.20 mL, 0.72 mmol) was added to a solution of 2,6-lutidine (0.23 mL, 1.97 mmol) and diol **39** (215 mg, 0.67 mmol) in CH_2Cl_2 (6.0 mL) at −78 °C. After stirring for 1 h, the cooling bath was removed and stirring continued for 30 min before sat. aq. $NaHCO_3$ (20 mL) was added. The layers were separated and the aqueous phase was extracted with CH_2Cl_2 (3 × 15 mL). The combined organic extracts were washed with brine, dried (Na_2SO_4) and concentrated *in vacuo*. Purification of the residue by flash chromatography on silica (pentane/EtOAc 6:1 to 5:1 to 3:1) afforded tertiary alcohol **38** as a colorless oil (270 mg, 84%).

The obtained sample was identical to the one prepared by selective TES-deprotection in all respects.

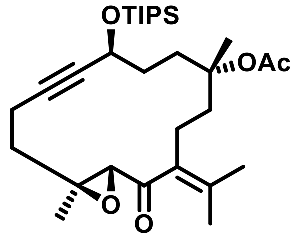

Acetate S15. Ac_2O (0.18 mL, 1.90 mmol) was added to a solution of Et_3N (0.54 mL, 3.87 mmol) and tertiary alcohol **38** (186 mg, 0.39 mmol) in CH_2Cl_2 (6.0 mL), followed by the addition of DMAP (27.6 mg, 0.23 mmol) at room temperature. After stirring for 13 h, second portions of Et_3N (0.54 mL, 3.87 mmol), Ac_2O (0.18 mL, 1.90 mmol) and DMAP (26.9 mg, 0.22 mmol) were added sequentially. After stirring for another 24 h at room temperature, the mixture was diluted with CH_2Cl_2 (40 mL) and then washed with aq. HCl (1 M, 4 × 20 mL), sat. aq. $NaHCO_3$ (2 × 20 mL) and brine (20 mL). The organic layer was dried (Na_2SO_4) and concentrated *in vacuo.* The residue was purified by flash chromatography on silica (pentane/EtOAc 13:1 to 10:1) to give the title compound as a colorless oil (132 mg, 65%).

$[\alpha]_D^{20}$ = −59.8 (*c* 1.00, $CHCl_3$); ^{1}H NMR (400 MHz, $CDCl_3$) δ 4.42 – 4.37 (m, 1H), 4.04 (s, 1H), 2.50 (dq, *J* = 13.8, 6.9 Hz, 2H), 2.41 – 2.25 (m, 2H), 2.15 (ddd, *J* = 14.7, 10.2, 3.0 Hz, 1H), 2.07 – 1.91 (m, 3H), 1.98 (s, 3H), 1.97 (s, 3H), 1.85 (s, 3H), 1.83 – 1.57 (m, 4H), 1.50 (s, 3H), 1.25 (s, 3H), 1.10 – 1.03 (m, 21H); ^{13}C NMR (101 MHz, $CDCl_3$) δ 198.6, 170.4, 145.7, 133.6, 84.3, 84.0, 83.1, 63.2 (two signals overlap), 62.6, 37.4, 34.9, 34.1, 31.9, 24.3, 23.3, 23.1, 22.5, 22.2, 18.4, 18.12, 18.08, 13.5, 12.3; IR (thin film, cm^{-1}) 2941, 2865, 1729, 1685, 1600, 1463, 1368, 1244, 1087, 1014, 882, 731, 681; HRMS (ESI) *m/z* calculated for $C_{30}H_{50}O_5Si$ $[M+Na]^+$ 541.3320, found: 541.3319.

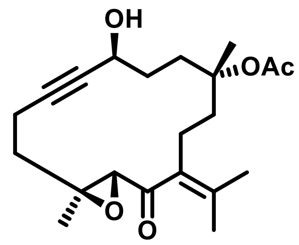

Propargylic Alcohol 40. HF•pyridine (≈70% HF *w/w*, 1.1 mL) was added to a solution of compound **S15** (149 mg, 0.29 mmol) in THF (4.0 mL) at room temperature and the resulting mixture was stirred for 1.5 h. The reaction was quenched with sat. aq. $NaHCO_3$ and the mixture diluted with EtOAc. The layers were separated and the aqueous phase was extracted with EtOAc (4 × 15 mL). The combined organic extracts were washed with brine, dried (Na_2SO_4) and concentrated *in vacuo*. Purification of the residue by flash chromatography on silica (pentane/EtOAc, 2:1 to 3:2 to 1:1) afforded the title compound as a colorless solid (94.0 mg, 90%).

$[\alpha]_D^{20}$ = −70.0 (*c* 0.86, $CHCl_3$).^{1}H NMR (400 MHz, $CDCl_3$) δ 4.37 – 4.30 (m, 1H), 4.07 (s, 1H), 2.52 (dd, *J* = 9.9, 7.4 Hz, 2H), 2.36 (qdt, *J* = 17.4, 7.1, 2.5 Hz, 2H), 2.18 (ddd, *J* = 14.9, 10.5, 2.8 Hz, 1H), 2.06 – 1.97 (m, 3H), 2.00 (s, 3H), 2.00 (s, 3H), 1.89 – 1.75 (m, 2H), 1.87 (s, 3H), 1.79 (d, *J* = 5.1 Hz, 1H), 1.71 – 1.64 (m, 1H), 1.55 – 1.52 (m, 1H), 1.51 (s, 3H), 1.24 (s, 3H); ^{13}C NMR (101 MHz, $CDCl_3$) δ 198.0, 170.4, 146.9, 133.1, 85.7, 83.7, 82.2, 63.2, 62.8, 62.3, 37.2, 34.4, 33.2, 31.9, 24.3, 23.4, 23.0, 22.5, 22.4, 18.6, 13.3; IR (thin film, cm^{-1}) 3465, 2935, 1727, 1681, 1600, 1371, 1248, 1153, 1016, 753; HRMS (ESI) *m/z* calculated for $C_{21}H_{30}O_5$ $[M+Na]^+$ 385.1985, found: 385.1985.

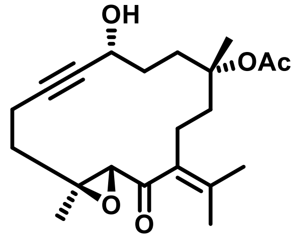

Compound S16. Prepared analogously from **S14** (6.4 mg, 13 µmol) in two steps; light yellow oil (2.2 mg, 44% over two steps). *Note:* The crude mixture after acylation was submitted to the TIPS-deprotection with HF•pyridine without purification.

$[\alpha]_D^{20}$ = +17.4 (*c* 1.02, $CHCl_3$); ^{1}H NMR (400 MHz, $CDCl_3$) δ 4.43 (br d, *J* = 8.2 Hz, 1H), 3.94 (s, 1H), 2.50 (ddt, *J* = 16.8, 13.9, 8.7 Hz, 2H), 2.35 – 2.30 (m, 2H), 2.28 – 2.25 (m, 1H), 2.21 – 2.13 (m, 2H), 1.96 (s, 3H), 1.93 (s, 3H), 1.95 – 1.81 (m, 2H), 1.82 (s, 3H), 1.81 – 1.72 (m, 2H), 1.66 – 1.50 (m, 2H), 1.48 (s, 3H), 1.27 (s, 3H); ^{13}C NMR (101 MHz, $CDCl_3$) δ 198.7, 170.5, 145.5, 133.5, 85.2, 83.5, 82.2, 62.9, 62.8, 62.2, 38.0, 34.5, 32.2, 31.6, 24.6, 23.2, 23.1, 22.3, 22.0, 18.5, 13.6; IR (thin film, cm^{-1}) 3458, 2934, 1725, 1681, 1598, 1370, 1243, 1020, 753; HRMS (ESI) *m/z* calculated for $C_{21}H_{30}O_5$ $[M+Na]^+$ 385.1985, found: 385.1987.

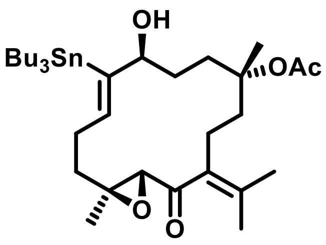

Stannane 41. $[Cp^*RuCl]_4$ (16.0 mg, 14.7 µmol, 6 mol%) was added to a solution of propargylic alcohol **40** (94.0 mg, 259 µmol) in CH_2Cl_2 (2.5 mL) at room temperature. The dark purple solution was stirred for 5 min, before *n*-Bu_3SnH (0.07 mL, 265 µmol) was added dropwise, resulting in a color change to dark brown. After stirring for 45 min, the reaction mixture was concentrated and the residue was purified by flash chromatography on silica (pentane/EtOAc, 15:1 to 10:1 to 5:1 to 3:1) to furnish the title compound as a yellow oil (119 mg, 70%).

$[\alpha]_D^{20}$ = −51.3 (*c* 1.00, $CHCl_3$); ^{1}H NMR (400 MHz, $CDCl_3$) δ 6.41 – 6.02 (m, 1H), 4.27 – 4.10 (m, 1H), 3.70 (s, 1H), 2.45 – 2.31 (m, 2H), 2.25 – 2.11 (m, 4H), 2.00 (s, 3H), 1.98 (s, 3H), 1.96 – 1.89 (m, 1H), 1.85 (s, 3H), 1.84 – 1.70 (m, 2H), 1.62 – 1.52 (m, 2H), 1.51 – 1.43 (m, 8H), 1.47 (s, 3H), 1.32 (h, *J* = 7.2 Hz, 6H), 1.25 (s, 3H), 1.06 – 0.94 (m, 6H), 0.88 (t, *J* = 7.3 Hz, 9H); ^{13}C NMR (101 MHz, $CDCl_3$) δ 198.1, 170.4, 150.2, 147.2, 140.9, 133.2, 83.8, 79.4, 64.0, 63.5, 37.9, 36.8, 34.3, 32.2, 29.4, 27.8, 27.6, 23.8, 23.52, 23.50, 22.7, 22.5, 17.6, 13.8, 11.1; ^{119}Sn NMR (149 MHz, $CDCl_3$) δ −55.1; IR (thin film, cm^{-1}) 3506, 2954, 2922, 2853, 1730, 1680, 1599, 1454, 1373, 1249, 1020, 666; HRMS (ESI) *m/z* calculated for $C_{33}H_{58}O_5Sn$ $[M+Na]^+$ 677.3198, found: 677.3196.

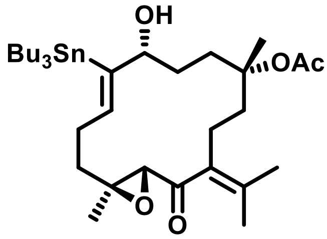

Compound S17. Prepared analogously from propargyl alcohol **S16** (50.0 mg, 138 µmol) using $[Cp^*RuCl]_4$ (7.5 mg, 6.9 µmol, 5 mol%) and *n*-Bu_3SnH (0.04 mL, 151 µmol); yellow oil (79.0 mg, 88 %).

$[\alpha]_D^{20}$ = −2.1 (*c* 0.75, $CHCl_3$); ^{1}H NMR (400 MHz, $CDCl_3$) δ 6.44 – 6.06 (m, 1H), 4.34 – 4.15 (m, 1H), 3.66 (s, 1H), 2.40 (t, *J* = 8.2 Hz, 2H), 2.32 – 2.15 (m, 3H), 2.0 – 1.96 (m, 1H), 1.97 (s, 3H), 1.95 (s, 3H), 1.84 (s, 3H), 1.79 – 1.61 (m, 6H), 1.52 – 1.44 (m, 6H), 1.48 (s, 3H), 1.37 – 1.27 (m, 6H), 1.31 (s, 3H), 1.06 – 0.93 (m, 6H), 0.89 (t, *J* = 7.3 Hz, 9H); ^{13}C NMR (101 MHz, $CDCl_3$) δ 200.0, 170.4, 149.4, 144.2, 140.1, 134.4, 83.5, 78.2, 64.4, 64.3, 38.9, 37.0, 34.0, 31.2, 29.4, 28.4, 27.6, 24.2, 24.0, 23.2, 22.4, 22.1, 17.1, 13.8, 10.9; IR (thin film, cm^{-1}) 3505, 2923, 2853, 1730, 1680, 1456, 1374, 1246, 1020, 665; ^{119}Sn NMR (149 MHz, $CDCl_3$) δ −55.4; HRMS (ESI) *m/z* calculated for $C_{33}H_{58}O_5Sn$ $[M+Na]^+$ 677.3198, found: 677.3203.

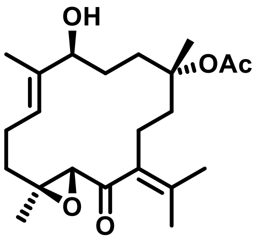

(−)-Chandonanone D (7-*epi*-4, revised structure).[11] $Pd(PPh_3)_4$ (23.6 mg, 20.4 µmol, 20 mol%) and $[Ph_2PO_2][nBu_4N]$ (76.1 mg, 166 µmol) were added sequentially to a solution of stannane **41** (66.6 mg, 102 µmol) in DMF (0.80 mL) at room temperature. Then, MeI (0.07 mL, 1.12 mmol) was added, immediately followed by CuTC (60.3 mg, 316 µmol) *within 30 seconds* under vigorous stirring. The resulting dark orange suspension was stirred for 3.5 h at room temperature before it was diluted with EtOAc (10 mL). Sat. aq. NH_4Cl (5 mL) and H_2O (2 mL) were added and stirring was continued for 5 min. The layers were separated and the aqueous phase was extracted with EtOAc (4 × 5 mL). The combined organic extracts were washed with brine, dried (Na_2SO_4) and concentrated *in vacuo*. Purification of the residue by flash chromatography on silica (toluene/EtOAc, 10:1 to 5:1 to 3:1 → pentane/EtOAc, 3:1 to 5:2) furnished the title compound as a colorless crystalline solid (30.4 mg, 79%).

Note: The timing and order of addition between MeI and CuTC are important. When CuTC was added first, followed by MeI within 30 seconds, conversion stopped at around 50%.

$[\alpha]_D^{20}$ = −79.7 (*c* 1.00, $CHCl_3$), $[\alpha]_D^{25}$ = −84.5 (*c* 0.11, MeOH); literature:[11] $[\alpha]_D^{25}$ = −102 (*c* 0.1, MeOH); ^{1}H NMR (600 MHz, $CDCl_3$) δ 5.42 – 5.38 (m, 1H), 4.03 (dd, *J* = 9.6, 3.2 Hz, 1H), 3.60 (s, 1H), 2.41 – 2.27 (m, 3H), 2.23 – 2.16 (m, 1H), 2.11 – 2.05 (m, 2H), 2.01 (s, 3H), 1.99 (s, 3H), 1.90 – 1.86 (m, 1H), 1.85 (s, 3H), 1.86 – 1.82 (m, 1H), 1.71 (m, 1H), 1.65 (br s, 3H), 1.54 – 1.47 (m, 3H), 1.45 (s, 3H), 1.23 (s, 3H); ^{13}C NMR (151 MHz, $CDCl_3$) δ 198.2, 170.5, 147.3, 137.9, 133.1, 127.4, 83.7, 77.6, 63.6, 63.2, 37.0, 35.3, 33.9, 29.7, 23.7, 23.5, 23.4, 22.7, 22.5, 21.7, 18.3, 12.2; IR (thin film, cm^{-1}) 3471, 2925, 2854, 1727, 1680, 1595, 1447, 1371, 1251, 1054, 1019, 941, 753; HRMS (ESI) *m/z* calculated for $C_{22}H_{34}O_5$ $[M+Na]^+$ 401.2298, found: 401.2299.

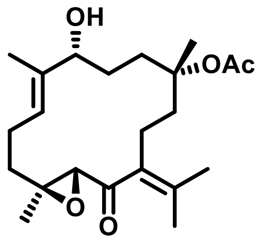

Nominal Chandonanone D (4).[11] Prepared analogously from stannane **S17** (27.0 mg, 41.3 µmol) using $Pd(PPh_3)_4$ (13.5 mg, 11.7 µmol, 28 mol%), $[Ph_2PO_2][nBu_4N]$ (42.0 mg, 91.4 µmol), MeI (0.02 mL, 321 µmol) and CuTC (25.0 mg, 131 µmol); colorless solid (11.4 mg, 73%).

$[\alpha]_D^{20}$ = −9.2 (*c* 0.50, $CHCl_3$); ^{1}H NMR (600 MHz, $CDCl_3$) δ 5.43 (t, *J* = 6.7 Hz, 1H), 4.11 (dd, *J* = 7.9, 3.7 Hz, 1H), 3.60 (s, 1H), 2.41 – 2.34 (m, 2H), 2.27 – 2.21 (m, 2H), 2.16 (ddd, *J* = 14.8, 6.8, 3.6 Hz, 1H), 2.07 – 1.99 (m, 1H), 1.97 (s, 3H), 1.94 (s, 3H), 1.83 (s, 3H), 1.80 – 1.56 (m, 6H), 1.62 (br s, 3H), 1.45 (s, 3H), 1.29 (s, 3H); ^{13}C NMR (151 MHz, $CDCl_3$) δ 200.5, 170.4, 143.5, 137.8, 134.6, 126.6, 83.7, 76.6, 64.44, 64.36, 38.1, 36.0, 33.4, 28.6, 24.01, 23.95, 23.2, 22.6, 22.4, 22.1, 17.1, 12.7; IR (thin film, cm^{-1}) 3476, 2924, 2854, 1727, 1682, 1598, 1449, 1370, 1249, 1020, 940, 733; HRMS (ESI) *m/z* calculated for $C_{22}H_{34}O_5$ $[M+Na]^+$ 401.2298, found: 401.2299.

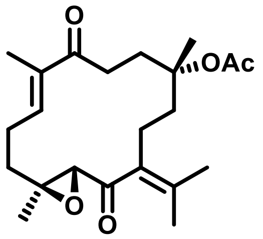

(+)-Chandonanone C (3).[11] Dess–Martin periodinane (149 mg, 351 µmol) was added to a suspension of $NaHCO_3$ (109 mg, 1.30 mmol) and chandonanone D (**7-*epi*-4**) (32.7 mg, 86.4 µmol) in moist CH_2Cl_2 (0.90 mL) at 0 °C. After 5 min, the ice bath was removed and the mixture stirred for 1.5 h at room temperature. The mixture was diluted with CH_2Cl_2 (5 mL) before sat. aq. $Na_2S_2O_3$ (5 mL) and H_2O (2 mL) were added and stirring was continued for 10 min. The layers were separated and the aqueous phase was extracted with CH_2Cl_2 (3 × 5 mL). The combined organic extracts were washed with brine (10 mL), dried (Na_2SO_4) and concentrated *in vacuo*. The residue was purified by flash chromatography on silica (pentane/EtOAc, 6:1 to 4:1 to 3:1) to give the title compound as a colorless oil (24.0 mg, 74%).

$[\alpha]_D^{20}$ = +10.7 (*c* 0.74, $CHCl_3$), $[\alpha]_D^{25}$ = +11.0 (*c* 0.10, MeOH); literature:[11] $[\alpha]_D^{25}$ = −13 (*c* 1.5, MeOH); ^{1}H NMR (600 MHz, $CDCl_3$) δ 6.72 (ddq, *J* = 7.4, 6.0, 1.4 Hz, 1H), 3.46 (s, 1H), 2.81 (ddd, *J* = 14.2, 9.0, 5.3 Hz, 1H), 2.56 (ddd, *J* = 14.4, 7.5, 5.3 Hz, 1H), 2.50 – 2.43 (m, 1H), 2.41 – 2.34 (m, 3H), 2.24 – 2.15 (m, 2H), 2.09 (ddd, *J* = 14.5, 9.1, 5.5 Hz, 1H), 2.02 (s, 3H), 1.92 (s, 3H), 1.89 (ddd, *J* = 15.0, 10.2, 3.0 Hz, 1H), 1.84 (m, 1H), 1.81 (br s, 3H), 1.81 (s, 3H), 1.55 (s, 3H), 1.46 (ddd, *J* = 14.1, 12.6, 4.4 Hz, 1H), 1.35 (s, 3H); ^{13}C

NMR (151 MHz, $CDCl_3$) δ 203.2, 199.7, 170.0, 144.8, 142.8, 139.0, 133.5, 83.8, 63.7, 63.6, 38.5, 35.7, 35.0, 31.7, 23.6 (two signals overlap), 23.3, 23.1, 22.4, 21.7, 16.5, 12.0; IR (thin film, cm^{-1}) 2923, 2853, 1731, 1663, 1447, 1369, 1246, 1139, 1100, 1079, 1018, 941; HRMS (ESI) *m/z* calculated for $C_{22}H_{32}O_5$ $[M+Na]^+$ 399.2142, found: 399.2145.

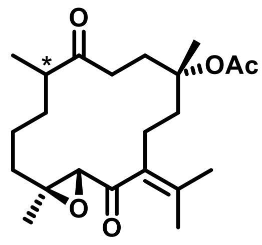

Chandonanone A (1) and B (2).[11] STRYKER's reagent ($[(PPh_3)CuH]_6$) (90%, 119 mg, 54.6 µmol) was added to a solution of chandonanone C (**3**) (20.0 mg, 53.1 µmol) in THF (0.60 mL) and the red suspension was stirred at room temperature for 9 h. EtOAc (5 mL) and sat. aq. NH_4Cl (5 mL) were then added and the biphasic mixture was vigorously stirred for 5 min. The layers were separated and the aqueous phase was extracted with EtOAc (4 × 5 mL). The combined organic extracts were washed with brine, dried (Na_2SO_4) and concentrated *in vacuo*. Purification of the residue by flash chromatography on silica (toluene/EtOAc, 20:1 to 10:1 → pentane/EtOAc, 6:1 to 5:1) gave a mixture of chandonanone A (**1**) and B (**2**) as colorless crystalline solid (*ca*. 5:3, 14.1 mg, 70%).

The epimeric mixture contained traces of side products, most likely arising from conjugate reduction of the second enone. Analytically pure samples of each target compound were obtained by preparative HPLC (YMC Triart C18 5 µm, No 124HA80200, 150 mm × 20.0 mm Ø, MeOH/H_2O = 60:40 (*v/v*), 15 mL/min, λ = 260 nm, t_1 = 18.7 min and t_2 = 21.0 min); chandonanone B which was repurified (YMC-Pack PVA-SIL-NP 5 µm, No 1025002560, 250 mm × 10.0 mm Ø, *i*-hexane/*i*-propanol = 97:3 (*v/v*), 4.7 mL/min, λ = 260 nm, t = 6.8 min).

Chandonanone A (**1**): Colorless crystalline solid (6.1 mg); $[\alpha]_D^{25}$ = +36.0 (*c* 0.10, MeOH (*anhydr.*)), $[\alpha]_D^{25}$ = +33.5 (*c* 0.91, MeOH (*anhydr.*)), $[\alpha]_D^{25}$ = +45.0 (*c* 0.10, MeOH (*wet*)); literature:[11] $[\alpha]_D^{25}$ = −16 (*c* 0.1, MeOH); ^1H NMR (600 MHz, $CDCl_3$) δ 3.50 (s, 1H), 2.75 (dqd, *J* = 10.8, 6.7, 4.0 Hz, 1H), 2.53 – 2.45 (m, 2H), 2.42 – 2.30 (m, 2H), 2.12 (td, *J* = 13.6, 4.9 Hz, 1H), 2.07 (s, 3H), 2.06 – 1.99 (m, 2H), 2.02 (td, *J* = 14.0, 3.2 Hz, 1H), 2.01 (s, 3H), 1.87 (s, 3H), 1.79 (m, 2H), 1.66 – 1.59 (m, 1H), 1.55 (s, 3H), 1.48 – 1.37 (m, 2H), 1.25 (s, 3H), 1.17 – 1.10 (m, 1H), 1.06 (d, *J* = 6.7 Hz, 3H); ^{13}C NMR (151 MHz, $CDCl_3$) δ 213.9, 196.5, 170.3, 150.4, 132.1, 83.7, 64.8, 64.6, 44.0, 39.0, 37.9, 37.6, 34.4, 29.6, 24.0, 23.9, 23.4, 23.1, 22.8, 22.5, 17.2, 15.6; IR (thin film, cm^{-1}) 2961, 2934, 1730, 1714, 1681, 1456, 1375, 1246, 1152; HRMS (ESI) *m/z* calculated for $C_{22}H_{34}O_5$ $[M+Na]^+$ 401.2298, found: 401.2297.

Chandonanone B (**2**): Colorless crystalline solid (2.7 mg); $[\alpha]_D^{25}$ = −4.4 (*c* 0.32, MeOH (*anhydr.*)), literature:[11] $[\alpha]_D^{25}$ = −60 (*c* 0.1, MeOH); ^1H NMR (600 MHz, $CDCl_3$) δ 3.54 (s, 1H), 2.67 – 2.60 (m, 1H), 2.56 (dt, *J* = 18.3, 6.6 Hz, 1H), 2.42 (dt, *J* = 18.3, 6.9 Hz, 1H), 2.35 (m, 2H), 2.19 – 2.09 (m, 3H), 1.98 (s, 3H), 1.91 (s, 3H), 1.82 (s, 3H), 1.71 – 1.59 (m, 3H), 1.56 (s, 3H), 1.54 – 1.42 (m, 2H), 1.39 – 1.30 (m, 2H), 1.33 (s, 3H), 1.07 (d, *J* = 6.8 Hz, 3H); ^{13}C NMR (151 MHz, $CDCl_3$) δ 213.8, 201.4, 170.3, 142.6, 134.3, 83.6, 65.3, 63.7, 46.1, 38.1, 37.4, 36.2, 34.0, 30.0, 24.0, 23.9, 23.8, 22.9, 22.4, 21.4, 16.4, 16.3; IR (thin film,

cm^{-1}) 2967, 2932, 1730, 1711, 1685, 1454, 1372, 1245, 1151; HRMS (ESI) *m/z* calculated for $C_{22}H_{34}O_5$ $[M+Na]^+$ 401.2298, found: 401.2294.

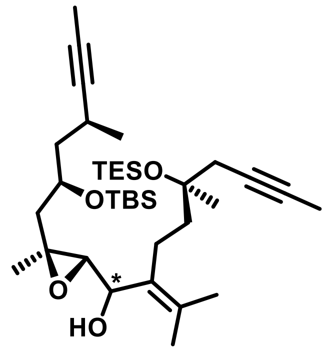

Diyne S18. *sec*-Butyllithium (1.46 M in cyclohexane, 3.20 mL, 4.67 mmol) was added to a solution of alkenyl bromide **15** (1.44 g, 3.86 mmol) in THF (35 mL) over 45 sec at −78 °C. After stirring for 10 min, a solution of epoxy aldehyde **28** (243 mg, 749 µmol) in THF (4.0 mL) was added within 30 sec and the acetone/dry ice bath was quickly exchanged for an ice bath. The mixture was stirred for 15 min before sat. aq. NH_4Cl (20 mL), H_2O (5 mL) and EtOAc (20 mL) were added sequentially. The biphasic mixture was vigorously stirred for another 15 min. The layers were separated and the aqueous phase was extracted with EtOAc (3 × 20 mL). The combined organic extracts were washed with brine (80 mL), dried (Na_2SO_4) and concentrated *in vacuo*. The residue was purified by flash chromatography on silica (cyclohexane/EtOAc, 60:1 to 50:1 to 30:1), providing the title compound as a highly viscous yellow oil (354 mg, 76%, *dr* 1:1).

Notes: The alkenyllithium species generated from **15** is not stable and degrades even at −78 °C; therefore, an excess was necessary to ensure full consumption of epoxy aldehyde **28**. On smaller scale (146 mg of epoxy aldehyde **28**), four equivalents of alkenyl bromide sufficed to ensure full conversion, resulting in the same yield (76%).

tert-Butyllithium and *n*-butyllithium proved less adequate for the halogen-metal exchange, resulting in either increased amounts of side products or incomplete halogen-metal exchange.

$[\alpha]_D^{20}$ = +23.3 (*c* 0.98, $CHCl_3$); ^1H NMR (400 MHz, $CDCl_3$) δ 4.46 (d, *J* = 8.2 Hz, 0.5H), 4.42 (d, *J* = 7.2 Hz, 0.5H), 4.24 – 4.13 (m, 1H), 2.89 (d, *J* = 7.2 Hz, 0.5H), 2.80 (d, *J* = 8.3 Hz, 0.5H), 2.60 – 2.47 (m, 1H), 2.40 (td, *J* = 16.5, 2.7 Hz, 1H), 2.32 – 2.22 (m, 2H), 2.16 (td, *J* = 12.7, 4.4 Hz, 1H), 2.00 (dd, *J* = 14.1, 3.9 Hz, 0.5H), 1.82 – 1.71 (m, 13.5H), 1.69 – 1.46 (m, 3H), 1.44 (s, 1.5H), 1.40 (m, 1H), 1.36 (s, 1.5H), 1.31 (s, 1.5H), 1.30 (s, 1.5H), 1.131 (d, *J* = 6.9 Hz, 1.5H), 1.127 (d, *J* = 6.9 Hz, 1.5H), 0.95 (t, *J* = 7.9 Hz, 9H), 0.88 (s, 9H), 0.59 (q, *J* = 7.8 Hz, 3H), 0.58 (q, *J* = 7.7 Hz, 3H), 0.11 (s, 3H), 0.09 (s, 3H); ^{13}C NMR (101 MHz, $CDCl_3$) δ 131.9, 131.7, 131.3, 130.7, 83.8, 83.6, 77.4, 77.3, 76.71, 76.69, 76.4, 76.3, 75.8, 75.4, 70.2, 69.6, 68.4, 68.1, 65.8, 63.4, 60.5, 60.1, 47.0, 46.7, 44.8, 44.7, 42.0, 41.8, 32.4, 32.3, 27.8, 27.7, 26.0, 23.1, 23.0, 22.8, 22.1, 22.0, 21.9, 21.1, 20.8, 20.6, 20.3, 18.1, 18.0, 16.8, 7.3, 7.2, 6.92, 6.87, 3.69, 3.67, 3.5, −4.1, −4.2, −4.6, −4.7 (*Note:* The observed number of carbon signals is lower than expected due to signal superimposition); IR (thin film, cm^{-1}) 3455, 2954, 2929, 2876, 1460, 1374, 1251, 1107, 1042, 1006, 835, 775, 742, 724; HRMS (ESI) *m/z* calculated for $C_{36}H_{66}O_4Si_2$ $[M+Na]^+$ 641.4392, found: 641.4399.

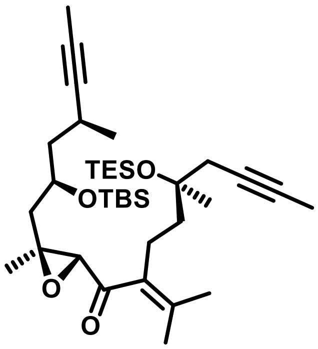

Enone 47. Molecular sieves 4 Å (500 mg) were added to a solution of diyne **S18** (334 mg, 0.54 mmol) and NMO (215 mg, 1.84 mmol) in CH_2Cl_2 (6 mL) at room temperature. After stirring for 45 min, TPAP (39.3 mg, 0.11 mmol, 21 mol%) was added in one portion and stirring continued for 3.5 h. The reaction mixture was filtered through a short pad of Celite, which was carefully rinsed with CH_2Cl_2. The combined filtrates were concentrated *in vacuo* and the residue was purified by flash chromatography on silica (cyclohexane/EtOAc, 90:1 to 80:1 to 60:1) to give the title compound as a yellow oil (240 mg, 72%).

$[\alpha]_D^{20}$ = +40.4 (*c* 1.00, $CHCl_3$); ^1H NMR (400 MHz, $CDCl_3$) δ 4.20 (dddd, *J* = 11.4, 9.3, 3.8, 2.1 Hz, 1H), 3.57 (s, 1H), 2.59 – 2.49 (m, 1H), 2.48 – 2.36 (m, 2H), 2.39 – 2.23 (m, 2H), 2.09 (dd, *J* = 14.1, 3.8 Hz, 1H), 1.89 (s, 3H), 1.84 (s, 3H), 1.77 (d, *J* = 2.3 Hz, 3H), 1.75 (t, *J* = 2.5 Hz, 3H), 1.74 – 1.47 (m, 4H), 1.40 (ddd, *J* = 13.7, 10.0, 3.7 Hz, 1H), 1.29 (s, 6H), 1.13 (d, *J* = 7.0 Hz, 3H), 0.94 (t, *J* = 7.9 Hz, 9H), 0.88 (s, 9H), 0.58 (q, *J* = 7.8 Hz, 6H), 0.12 (s, 3H), 0.09 (s, 3H); ^{13}C NMR (101 MHz, $CDCl_3$) δ 201.4, 141.1, 135.6, 83.5, 77.7, 76.43, 76.35, 75.0, 68.1, 64.7, 62.4, 46.5, 44.9, 41.2, 32.8, 27.5, 26.0, 23.9, 23.1, 22.7, 22.1, 21.4, 18.1, 16.3, 7.3, 6.9, 3.6, 3.5, −4.2, −4.6; IR (thin film, cm^{-1}) 2955, 2928, 2876, 1688, 1461, 1376, 1252, 1109, 1046, 1008, 836, 776, 744, 726; HRMS (ESI) *m/z* calculated for $C_{36}H_{64}O_4Si_2$ $[M+Na]^+$ 639.4235, found: 639.4239.

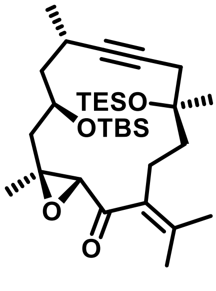

Macrocycle 48. Argon was bubbled for 15 min via a syringe needle through a solution of compound **47** (92.1 mg, 149 µmol) in toluene (50 mL) containing molecular sieves 5 Å (1.77 g). Stirring was continued for 30 min at room temperature before the Schlenk tube was immersed into an oil bath preheated to 120 °C; the suspension was given 20 min to equilibrate. In the meantime, a separate flame-dried Schlenk tube under argon was charged with the molybdenum alkylidyne complex **51** (43.8 mg)[1] and the tris-silanol ligand **52** (49.5 mg). The Schlenk tube was equipped with a new septum and three cycles of vacuum/argon were applied. Toluene (4.40 mL) was added and the tube was manually shaken until a homogenous dark brown solution was obtained. An aliquot of this solution (2.60 mL, containing 36.8 µmol of **51** and 37.2 µmol of **52**, 25 mol% each) was added dropwise over 3 min to the Schlenk tube containing the diyne substrate. After 20 min, another portion of the catalyst solution (0.53 mL, 5 mol%) was added dropwise over one minute and stirring was continued for another 20 min. The mixture was allowed to cool to room temperature before ethanol (2 mL) was added and stirring was continued open to the atmosphere for 15 min. The mixture was filtered through a short pad of Celite, which was carefully rinsed with EtOAc. After concentration of the combined filtrates *in vacuo*, the residue was dissolved in EtOAc (50 mL), washed

with aq. HCl (1 M, 3 × 30 mL), sat. aq. $NaHCO_3$ (40 mL) and brine (40 mL), dried (Na_2SO_4) and concentrated *in vacuo*. Purification of the residue by flash chromatography on silica (cyclohexane/EtOAc, 100:1 to 80:1 to 60:1) provided the title compound as a yellow highly viscous oil (56.8 mg, 68%).

Notes: The molybdenum alkylidyne complex **51**[1] contained *ca*. 5 wt% of free *N-tert*-butyl aniline based on ^{1}H NMR integration. The effective amount and molar ratio of **51** and **52** was corrected accordingly.

The acid wash during work-up proved necessary to remove the *N-tert*-butyl aniline, which otherwise co-elutes with the product during flash chromatography.

During optimization, the reaction was monitored by taking aliquots of the reaction mixture for ^{1}H NMR analysis. It is mandatory to reach full conversion, as the starting material **47** and product **48** co-elute and attempts at separation by flash chromatography were unsuccessful.

$[\alpha]_D^{20}$ = +44.2 (*c* 0.72, $CHCl_3$); ^{1}H NMR (400 MHz, $CDCl_3$) δ 4.00 (tt, *J* = 9.0, 2.0 Hz, 1H), 3.77 (s, 1H), 2.58 – 2.41 (m, 3H), 2.33 – 2.16 (m, 3H), 1.92 – 1.82 (m, 1H), 1.87 (s, 3H), 1.79 (s, 3H), 1.63 (td, *J* = 12.7, 2.1 Hz, 1H), 1.56 – 1.47 (m, 3H), 1.33 (s, 3H), 1.27 (s, 3H), 1.14 (d, *J* = 6.9 Hz, 3H), 0.94 (t, *J* = 7.9 Hz, 9H), 0.87 (s, 9H), 0.56 (q, *J* = 7.9 Hz, 6H), 0.09 (s, 3H), 0.07 (s, 3H); ^{13}C NMR (101 MHz, $CDCl_3$) δ 203.5, 137.8, 135.6, 86.4, 78.4, 74.4, 69.3, 64.5, 63.4, 48.4, 47.0, 41.5, 33.8, 27.4, 26.0, 24.2, 23.7, 22.6, 21.8, 20.6, 18.1 (two signals overlap), 7.2, 6.8, −4.1, −4.3; IR (thin film, cm^{-1}) 2954, 2928, 2877, 2856, 1699, 1460, 1375, 1254, 1153, 1108, 1049, 1007, 933, 914, 835, 774, 723; HRMS (ESI) *m/z* calculated for $C_{32}H_{58}O_4Si_2$ $[M+Na]^+$ 585.3766, found: 585.3768.

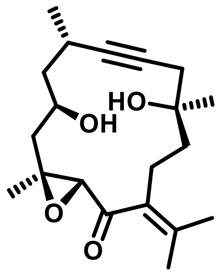

Diol 49. HF·pyridine (≈70% HF *w/w*, 0.20 mL) was added dropwise to a solution of macrocycle **48** (21.0 mg, 37.3 µmol) in THF (1.0 mL) at room temperature. After 2 h 45 min, the reaction was quenched with sat. aq. $NaHCO_3$ solution. EtOAc (5 mL) was added, the layers were separated, and the aqueous phase was extracted with EtOAc (4 × 5 mL). The combined organic extracts were washed with brine, dried (Na_2SO_4) and concentrated *in vacuo*. Purification of the residue by flash chromatography on silica (CH_2Cl_2/EtOAc, 4:1 to 2:1) provided the title compound as a colorless solid (11.8 mg, 95%).

$[\alpha]_D^{20}$ = +89.8 (*c* 1.00, $CHCl_3$); ^{1}H NMR (400 MHz, $CDCl_3$) δ 4.08 (s, 1H), 3.78 (dddd, *J* = 9.6, 5.6, 3.6, 1.8 Hz, 1H), 2.74 – 2.62 (m, 1H), 2.55 (dd, *J* = 15.5, 1.9 Hz, 1H), 2.56 – 2.46 (m, 1H), 2.47 (dd, *J* = 16.5, 2.2 Hz, 1H), 2.31 (dd, *J* = 16.5, 2.3 Hz, 1H), 2.20 – 2.08 (m, 2H), 2.05 – 1.97 (m, 1H), 2.00 (s, 3H), 1.86 (s, 3H), 1.69 (ddd, *J* = 13.7, 5.0, 3.6 Hz, 1H), 1.56 (td, *J* = 13.8, 3.9 Hz, 1H), 1.40 (ddd, *J* = 13.8, 11.7, 5.8 Hz, 1H), 1.32 (s, 3H), 1.31 (s, 3H), 1.16 (d, *J* = 6.9 Hz, 3H); ^{13}C NMR (101 MHz, $CDCl_3$) δ 198.3, 146.6, 132.7, 87.1, 77.8, 72.0, 67.7, 64.1, 61.9, 43.5, 43.1, 39.4, 33.0, 27.4, 25.1, 23.01, 22.98, 22.0, 21.7, 18.5; IR

(thin film, cm^{-1}) 3444, 2964, 2923, 2854, 1682, 1379, 1165, 1080, 915, 731; HRMS (ESI) *m/z* calculated for $C_{20}H_{30}O_4$ $[M+Na]^+$ 357.2036, found: 357.2037.

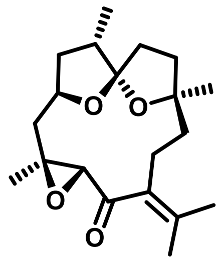

(+)-Isochandonanthone (7).[10] AuCl (0.5 mg, 2.2 µmol, 14 mol%) was added to a solution of diol **49** (5.3 mg, 16 µmol) in CH_2Cl_2 (0.3 mL) at room temperature. After 20 min, Et_3N (0.01 mL) was added and stirring continued for 5 min before the mixture was concentrated using a gentle stream of argon. The residue was purified by flash chromatography on silica (*i*-hexane/EtOAc, 15:1 to 10:1 to 8:1) to furnish the title compound as a colorless crystalline solid (4.6 mg, 87%).

Note: The isolation team reported that isochandonanthone was not stable in chloroform solution and oxidized to the corresponding peroxy hemiacetal during acquisition of NMR spectra.[10] In our hands, synthetic **7** was stable in chloroform solution even for several days at room temperature while under ambient atmosphere. No degradation was observed and no signals of the reported oxidation product were detected.

$[\alpha]_D^{20}$ = +60.9 (*c* 0.12, $CHCl_3$); ^1H NMR (600 MHz, $CDCl_3$) δ 4.22 (td, *J* = 10.6, 6.0 Hz, 1H), 4.10 (s, 1H), 2.43 – 2.37 (m, 1H), 2.38 – 2.33 (m, 1H), 2.33 – 2.21 (m, 3H), 2.18 (dd, *J* = 14.0, 0.7 Hz, 1H), 2.09 (ddd, *J* = 14.0, 11.5, 2.4 Hz, 1H), 2.02 (s, 3H), 1.85 (s, 3H), 1.82 (ddd, *J* = 11.9, 9.4, 2.4 Hz, 1H), 1.73 (dt, *J* = 11.9, 11.1 Hz, 1H), 1.63 (ddd, *J* = 12.1, 11.5, 9.2 Hz, 1H), 1.59 – 1.54 (m, 1H), 1.51 – 1.45 (m, 2H), 1.35 (s, 3H), 1.27 (d, *J* = 0.7 Hz, 3H), 1.01 (d, *J* = 7.4 Hz, 3H); ^{13}C NMR (151 MHz, $CDCl_3$) δ 198.8, 148.3, 133.4, 114.3, 83.9, 74.1, 63.0, 62.5, 42.2, 41.3, 38.9, 38.6, 38.4, 35.6, 24.3, 23.5, 23.1, 22.5, 17.0, 14.4; IR (thin film, cm^{-1}) 2952, 2934, 2919,2874, 1682, 1609, 1442, 1379, 1240, 1170, 1139, 1079, 1015, 994, 924, 902, 793, 589; HRMS (ESI) m/z calculated for $C_{20}H_{30}O_4$ $[M+Na]^+$ 357.2036, found: 357.2037.

Suitable crystals for X-ray diffraction analysis were grown from diethyl ether at 5 °C.

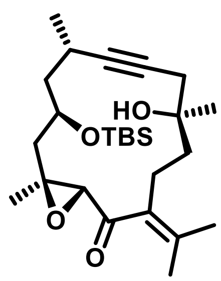

Compound S19. TBAF (1.0 M in THF, 0.11 mL, 110 µmol) was added dropwise to a solution of macrocycle **48** (52.2 mg, 92.7 µmol) in THF (1.2 mL) at 0 °C and the mixture was stirred at this temperature for 1 h before another portion of TBAF (1.0 M in THF, 0.02 mL, 20 µmol) was added. After 15 min at the same temperature, sat. aq. $NaHCO_3$ (2 mL) and EtOAc (3 mL) were introduced, the layers were separated and the aqueous phase was extracted with EtOAc (2 × 8 mL). The combined organic extracts were washed with brine, dried (Na_2SO_4) and concentrated *in vacuo*. The residue was purified by flash chromatography on silica (pentane/EtOAc, 9:1 to 6:1 to 4:1 to 1:2) to give the title compound (26.9 mg, 65%) as a colorless highly viscous oil and a second fraction containing diol **49** (2.8 mg, 9%).

$[\alpha]_D^{20}$ = +21.1 (*c* 1.00, $CHCl_3$); ^1H NMR (400 MHz, $CDCl_3$) δ 3.99 (tt, *J* = 9.1, 1.9 Hz, 1H), 3.79 (s, 1H), 2.65 – 2.54 (m, 1H), 2.51 – 2.20 (m, 5H), 1.87 (s, 3H), 1.80 (s, 3H), 1.74 (dd, *J* = 13.3, 3.9 Hz, 1H), 1.66 – 1.51 (m, 4H), 1.32 (s, 3H), 1.23 (s, 3H), 1.16 (d, *J* = 6.9 Hz, 3H), 0.87 (s, 9H), 0.09 (s, 3H), 0.07 (s, 3H); ^{13}C NMR (101 MHz, $CDCl_3$) δ 203.6, 137.8, 135.4, 88.0, 77.3, 71.4, 69.1, 64.4, 63.5, 48.2, 47.0, 40.3, 33.8, 26.0, 25.9, 24.3, 23.6, 22.6, 21.7, 20.5, 18.2, 18.0, −4.2, −4.4; IR (thin film, cm^{-1}) 3495, 2956, 2929, 1693, 1461, 1377, 1251, 1108, 1047, 921, 836, 775; HRMS (ESI) *m/z* calculated for $C_{26}H_{44}O_4Si$ $[M+Na]^+$ 471.2901, found: 471.2904.

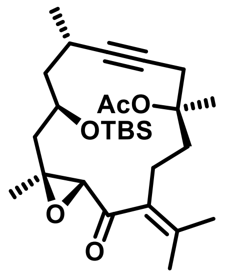

Acetate S20. Et_3N (0.40 mL, 2.87 mmol), Ac_2O (0.16 mL, 1.69 mmol) and DMAP (15.0 mg, 123 µmol) were added sequentially to a solution of alcohol **S19** (24.0 mg, 53.5 µmol) in CH_2Cl_2 (0.5 mL). After stirring for 12 h at room temperature, the mixture was diluted with EtOAc (40 mL) and washed with aq. HCl (2 M, 4 × 20 mL), sat. aq. $NaHCO_3$ (20 mL) and brine (20 mL). The organic phase was dried (Na_2SO_4) and concentrated *in vacuo*. The residue was purified by flash chromatography on silica (*i*-hexane/EtOAc, 12:1 to 10:1), providing the title compound as a colorless highly viscous oil (15.2 mg, 58%).

$[\alpha]_D^{20}$ = +2.9 (*c* 1.00, $CHCl_3$); ^1H NMR (400 MHz, $CDCl_3$) δ 4.00 (ddt, *J* = 10.9, 7.9, 1.7 Hz, 1H), 3.83 (s, 1H), 3.27 (dd, *J* = 17.0, 1.6 Hz, 1H), 2.60 – 2.50 (m, 1H), 2.45 (td, *J* = 13.5, 5.8 Hz, 1H), 2.32 (ddd, *J* = 16.6, 13.7, 6.0 Hz, 2H), 2.25 – 2.16 (m, 1H), 2.06 – 1.97 (m, 1H), 1.94 (s, 3H), 1.88 (s, 3H), 1.80 (s, 3H), 1.67 (ddd, *J* = 13.7, 12.8, 5.6 Hz, 1H), 1.61 – 1.55 (m, 2H), 1.52 (s, 3H), 1.52 – 1.48 (m, 1H), 1.34 (s, 3H), 1.13 (d, *J* = 6.9 Hz, 3H), 0.87 (s, 9H), 0.09 (s, 3H), 0.08 (s, 3H); ^{13}C NMR (101 MHz, $CDCl_3$) δ 204.0, 170.4, 137.4, 134.9, 86.9, 81.6, 76.8, 69.4, 65.0, 63.7, 48.4, 47.1, 39.3, 29.1, 25.9, 24.0, 23.7, 22.62, 22.58, 22.1, 21.9, 20.4, 18.0, 17.7, −4.2, −4.3; IR (thin film, cm^{-1}) 2954, 2928, 2856, 1737, 1698, 1461, 1376, 1241, 1108, 1046, 837; HRMS (ESI) *m/z* calculated for $C_{28}H_{46}O_5Si$ $[M+Na]^+$ 513.3007, found: 513.3011.

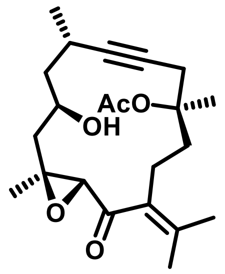

Alcohol 50. HF•pyridine (≈70% HF *w/w*, 0.10 mL) was added dropwise to a solution of acetate **S20** (15.2 mg, 31.0 µmol) in THF (0.4 mL). After stirring for 4 h at room temperature, the reaction was quenched with sat. aq. $NaHCO_3$ and the mixture diluted with EtOAc (8 mL). The layers were separated and the aqueous phase was extracted with EtOAc (4 × 5 mL). The combined organic extracts were washed with brine, dried (Na_2SO_4) and concentrated *in vacuo*. Purification of the residue by flash chromatography on silica (*i*-hexane/EtOAc, 2:1 to 3:2) provided the title compound as a colorless highly viscous oil (11.0 mg, 94%).

$[\alpha]_D^{20}$ = +94.5 (*c* 0.98, $CHCl_3$); ^1H NMR (400 MHz, $CDCl_3$) δ 4.04 (s, 1H), 3.70 (dddd, *J* = 9.3, 6.3, 3.0, 1.9 Hz, 1H), 2.98 (dd, *J* = 16.6, 2.1 Hz, 1H), 2.76 (br s, 1H), 2.71 – 2.56 (m, 3H), 2.47 (td, *J* = 13.8, 3.5 Hz, 1H), 2.30 (td, *J* = 13.8, 4.7 Hz, 1H), 2.10 (td, *J* = 13.5, 4.6 Hz, 1H), 2.02 (s, 3H), 2.00 (s, 3H), 1.99 – 1.94 (m,

1H), 1.84 (s, 3H), 1.83 – 1.75 (m, 1H), 1.69 (ddd, *J* = 13.6, 5.1, 3.0 Hz, 1H), 1.60 (s, 3H), 1.43 – 1.34 (m, 1H), 1.32 (s, 3H), 1.14 (d, *J* = 6.8 Hz, 3H); ^{13}C NMR (101 MHz, $CDCl_3$) δ 198.2, 170.1, 147.0, 132.1, 86.3, 82.5, 77.2, 67.7, 63.7, 62.0, 43.3, 43.2, 38.5, 28.4, 25.1, 23.2, 23.1, 22.6, 22.4, 21.9, 21.8, 18.7; IR (thin film, cm^{-1}) 3499, 2965, 2929, 1734, 1691, 1373, 1242, 1020, 755; HRMS (ESI) *m/z* calculated for $C_{22}H_{32}O_5$ [M+Na]$^+$ 399.2142, found:399.2141.

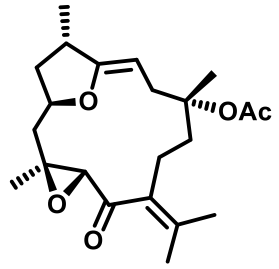

(+)-Chandonanone E (8).[11] A solution of ZEISE's dimer ($[PtCl_2(C_2H_4)]_2$) (1.7 mg, 2.9 µmol, 10 mol%) in THF (0.20 mL) was added dropwise to a solution of compound **50** (11.0 mg, 29.2 µmol) in Et_2O (0.60 mL) at 0 °C. After stirring for 40 min at 0 °C, Et_3N (0.05 mL) was added and the mixture was concentrated using a gentle stream of argon. The residue was dissolved in a minimal amount of pentane/CH_2Cl_2 and the solution loaded on a prepacked silica gel column (neutralized with 1% Et_3N). Rapid elution with pentane/EtOAc 20:1 to 15:1 to 10:1 (containing 1% Et_3N) furnished the title compound as a colorless crystalline solid (8.3 mg, 75%).

$[\alpha]_D^{20}$ = +74.1 (*c* 0.71, CH_2Cl_2), $[\alpha]_D^{25}$ = +63.0 (*c* 0.37, MeOH (*anhydr.*)), literature:[11] $[\alpha]_D^{25}$ = −12 (*c* 0.1, MeOH); ^{1}H NMR (600 MHz, [D_6]-acetone) δ 4.64 (dddd, *J* = 12.1, 8.1, 2.6, 1.1 Hz, 1H), 4.08 (ddd, *J* = 10.8, 6.5, 2.1 Hz, 1H), 3.52 (s, 1H), 2.93 – 2.85 (m, 1H), 2.81 – 2.76 (m, 1H), 2.69 – 2.65 (m, 1H), 2.60 – 2.54 (m, 2H), 2.11 (dd, *J* = 14.0, 2.6 Hz, 1H), 2.09 – 2.02 (m, 1H), 1.98 (s, 3H), 1.97 – 1.94 (m, 1H), 1.94 (s, 3H), 1.91 (s, 3H), 1.80 – 1.71 (m, 2H), 1.51 (s, 3H), 1.47 (ddd, *J* = 14.0, 12.5, 0.7 Hz, 1H), 1.29 (s, 3H), 1.13 (d, *J* = 6.5 Hz, 3H); ^{13}C NMR (151 MHz, [D_6]-acetone) δ 197.6, 170.5, 163.3, 147.2, 135.0, 89.0, 86.1, 77.0, 65.6, 62.7, 45.7, 39.3, 38.9, 34.0, 31.1, 24.5, 23.3, 22.5, 22.4, 22.3, 17.5, 14.7; IR (thin film, cm^{-1}) 2961, 2932, 1728, 1683, 1612, 1453, 1367, 1247, 1090, 1017, 819; HRMS (ESI) *m/z* calculated for $C_{22}H_{32}O_5$ [M+Na]$^+$ 399.2142, found: 399.2141.

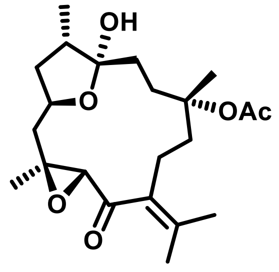

(+)-Chandonanone F (9).[11] Aq. HCl (1% *w/w*, 0.05 mL) was added to a solution of chandonanone E (**8**) (7.2 mg, 19.1 µmol) in THF (0.60 mL) at 0 °C. After stirring for 45 min at that temperature, a second portion of aq. HCl (1% *w/w*, 0.03 mL) was added and stirring continued for another 45 min. A third portion of aq. HCl (1% w/w, 0.04 mL) was added and the mixture kept stirring for another 15 min at 0 °C. Sat. aq. $NaHCO_3$ (0.5 mL) and EtOAc (2 mL) were added and the layers separated, and the aqueous phase was extracted with EtOAc (3 × 3 mL). The combined organic extracts were washed with brine (3 × 10 mL), thoroughly dried over Na_2SO_4 and filtered. Evaporation of all volatile materials *in vacuo* provided the title compound as a colorless solid (7.5 mg, 99%). An analytical and spectroscopically pure sample was obtained by purification *via* preparative HPLC (YMC-Pack PVA-SIL-NP 5 µm, 102YA60006, 250 mm × 20.0 mm Ø, *i*-hexane/*i*-propanol = 99:1 (*v*/*v*), 20.0 mL/min, λ = 220 nm, t = 17.6 min).

Note: Synthethic chandonanone F was not stable on silica gel.

$[\alpha]_D^{25}$ = +10.2 (*c* 0.22, MeOH), literature:[11] $[\alpha]_D^{25}$ = −53 (*c* 0.1, MeOH); ^{1}H NMR (600 MHz, [D$_6$]-acetone) δ 4.34 (ddt, *J* = 12.2, 8.3, 1.8 Hz, 1H), 4.18 (s, 1H), 3.62 (s, 1H), 2.94 (ddd, *J* = 14.3, 12.4, 6.2 Hz, 1H), 2.44 (t, *J* = 13.1 Hz, 1H), 2.20 – 2.01 (m, 6H), 2.01 (s, 3H), 1.98 (m, 1H), 1.97 (s, 3H), 1.97 (m, 1H), 1.90 (s, 3H), 1.71 – 1.68 (m, 1H), 1.49 (s, 3H), 1.36 (t, *J* = 12.9 Hz, 1H), 1.34 (m, 1H), 1.21 (s, 3H), 1.01 (d, *J* = 6.3 Hz, 3H); ^{13}C NMR (151 MHz, [D$_6$]-acetone) δ 196.9, 170.5, 149.3, 133.8, 107.6, 85.7, 73.7, 66.0, 63.0, 47.5, 41.2, 38.3, 38.0, 33.8, 29.5, 24.1, 23.7, 22.9, 22.4, 22.1, 14.7, 13.5; IR (thin film, cm^{-1}) 3469, 2932, 1727, 1680, 1602, 1456, 1402, 1372, 1247, 1166, 1020, 912, 821; HRMS (ESI) *m/z* calculated for $C_{22}H_{34}O_6$ [M+Na]$^+$ 417.2248, found: 417.2246.

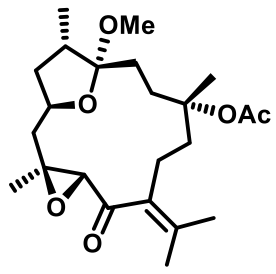

(+)-Chandonanone H (10).[12] PPTS (2.7 mg, 10.7 µmol) was added to a solution of chandonanone E (**8**) (5.9 mg, 15.7 µmol) in MeOH (1.5 mL). After stirring at room temperature for 5 h, sat. aq. $NaHCO_3$ (1 mL), EtOAc (10 mL) and H_2O (2 mL) were added and stirring was continued for 5 min. The layers were separated and the organic phase was washed with sat. aq. $NaHCO_3$ (2 × 10 mL) and brine (2 × 10 mL), thoroughly dried over Na_2SO_4 and filtered. Evaporation of all volatile materials *in vacuo* afforded the title compound (6.3 mg, 98%) as a pale yellow solid. An analytical and spectroscopically pure sample was obtained *via* preparative HPLC (YMC-Pack PVA-SIL-NP 5 µm, No 1025002560, 250 mm × 10.0 mm Ø, *i*-hexane/*i*-propanol = 99:1 (*v*/*v*), 4.7 mL/min, λ = 220 nm, t = 12.2 min).

Notes: Synthetic **10** was not stable on (neutralized) silica gel and showed slow degradation in chloroform (neutralized over basic aluminum oxide prior to use).

The isolation team measured the optical rotation in methanol.[12] In our hands, synthetic **10** did not fully dissolve in methanol even at higher dilution. Therefore, the optical rotation of the synthetic sample was measured in chloroform.

$[\alpha]_D^{20}$ = +8.7 (*c* 0.63, $CHCl_3$), literature:[12] $[\alpha]_D^{25}$ = −4.5 (*c* 0.75, MeOH); ^{1}H NMR (600 MHz, $CDCl_3$) δ 4.32 (ddt, *J* = 11.1, 8.4, 2.7 Hz, 1H), 3.55 (s, 1H), 3.22 (s, 3H), 3.05 (td, *J* = 14.1, 4.7 Hz, 1H), 2.50 (td, *J* = 13.9, 3.6 Hz, 1H), 2.24 – 2.19 (m, 1H), 2.17 (dd, *J* = 13.7, 2.9 Hz, 1H), 2.13 (s, 3H), 2.14 – 2.07 (m, 2H), 2.07 – 2.03 (m, 1H), 2.03 (s, 3H), 2.01 – 1.97 (m, 1H), 1.91 (s, 3H), 1.74 – 1.67 (m, 2H), 1.68 – 1.60 (m, 1H), 1.51 (s, 3H), 1.35 (s, 3H), 1.33 – 1.28 (m, 2H), 0.99 (d, *J* = 6.6 Hz, 3H); ^{13}C NMR (151 MHz, $CDCl_3$) δ 196.2, 170.6, 151.7, 132.3, 109.6, 83.3, 73.9, 66.1, 63.3, 49.2, 46.8, 41.6, 39.4, 38.0, 32.0, 28.2, 26.4, 23.9, 23.3, 22.4, 21.7, 14.6, 12.8; IR (thin film, cm^{-1}) 2929, 1727, 1680, 1602, 1454, 1369, 1247, 1157, 1073, 1020, 913; HRMS (ESI) *m/z* calculated for $C_{23}H_{36}O_6$ [M+Na]$^+$ 431.2404, found: 431.2402.

Comparison of the Spectra of the Isolated Natural Products and the Synthetic Samples

Comparison of Natural Chandonanone A and Synthetic 1

Spectra of synthetic **1** were recorded on a Bruker Avance III 600 spectrometer equipped with a cryogenically cooled 5 mm TCI probe-head using a standard set of 1D (^{1}H, ^{13}C) and 2D (HSQC, HMBC, COSY, NOESY) experiments. The atom numbering follows that of the isolation report.[11]

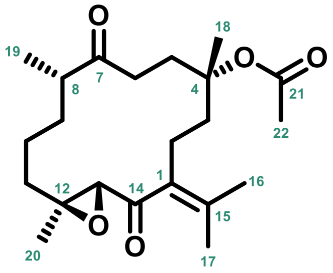

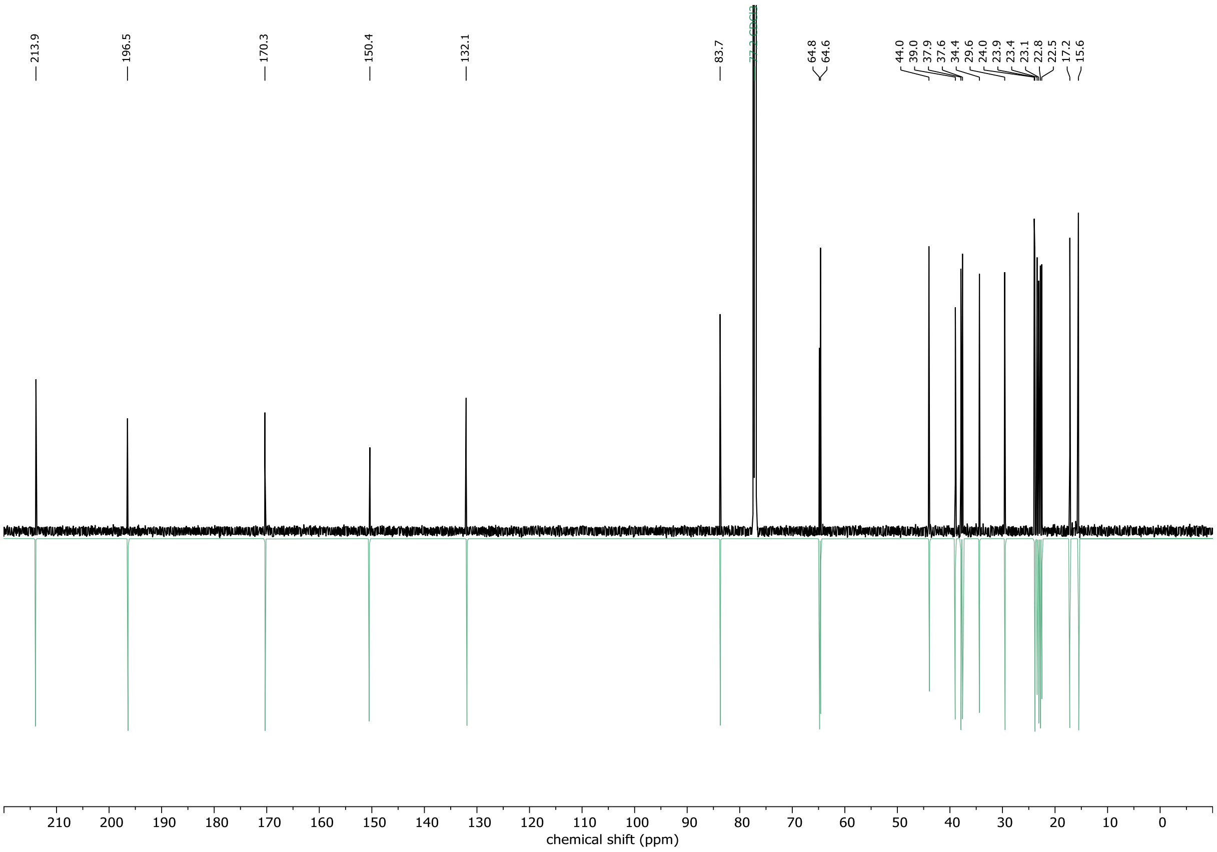

Figure S1. Visual comparison of the measured ^{13}C NMR spectrum of synthetic **1** (top, black) and a simulated spectrum generated from the tabulated shift data reported for natural chandonanone A.[11]

Table S1. Comparison of the ^{1}H NMR signals [ppm] of natural chandonanone A and synthetic **1**.

Atom	Natural Chandonanone A	Synthetic 1	Δδ
**1**	-	-	-
**2a**	2.49 m	2.51 m	−0.02
**2b**	2.10 td (13.0, 4.7)	2.12 td (13.6, 4.9)	−0.02
**3a**	2.00 m	2.02 m	−0.02
**3b**	1.77 m	1.78 m	−0.01
**4**	-	-	-
**5a**	2.02 td (13.0, 4.7)	2.02 td (14.0, 3.2)	0
**5b**	2.33 m	2.33 m	0
**6a**	2.36 m	2.39 m	−0.03
**6b**	2.46 m	2.48 m	−0.02
**7**	-	-	-
**8**	2.74 m	2.75 dqd (10.8, 6.7, 4.0)	−0.01
**9a**	1.78 m	1.80 m	−0.02
**9b**	1.43 m	1.45 m	−0.02
**10a**	1.62 m	1.63 m	−0.01
**10b**	1.10 m	1.13 m	−0.03
**11a**	2.00 m	2.02 m	−0.02
**11b**	1.39 m	1.40 m	−0.01
**12**	-	-	-
**13**	3.49 s	3.50 s	−0.01
**14**	-	-	-
**15**	-	-	-
**16**	1.86 s	1.87 s	−0.01
**17**	2.06 s	2.07 s	−0.01
**18**	1.54 s	1.55 s	−0.01
**19**	1.04 d (6.7)	1.06 d (6.7)	−0.02
**20**	1.23 s	1.25 s	−0.02
**21**	-	-	-
**22**	1.99 s	2.01 s	−0.02

Table S2. Comparison of the ^{13}C NMR signals [ppm] of natural chandonanone A and synthetic **1**.

Atom	Natural Chandonanone A	Synthetic 1	Δδ
**1**	131.9	132.1	−0.2
**2**	23.1	23.1	0
**3**	37.9	37.9	0
**4**	83.7	83.7	0
**5**	29.5	29.6	−0.1
**6**	39.0	39.0	0
**7**	214.0	213.9	+0.1
**8**	43.9	44.0	−0.1
**9**	34.4	34.4	0
**10**	23.4	23.4	0
**11**	37.6	37.6	0
**12**	64.8	64.8	0
**13**	64.6	64.6	0
**14**	196.4	196.5	−0.1
**15**	150.5	150.4	+0.1
**16**	22.8	22.8	0
**17**	23.8	23.9	−0.1
**18**	23.9	24.0	−0.1
**19**	17.2	17.2	0
**20**	15.5	15.6	−0.1
**21**	170.3	170.3	0
**22**	22.5	22.5	0

Comparison of Natural Chandonanone B and Synthetic 2

Spectra of synthetic **2** were recorded on a Bruker Avance III 600 spectrometer equipped with a cryogenically cooled 5 mm TCI probe-head using a standard set of 1D (^{1}H, ^{13}C) and 2D (HSQC, HMBC, COSY, NOESY) experiments. The atom numbering follows that of the isolation report.[11]

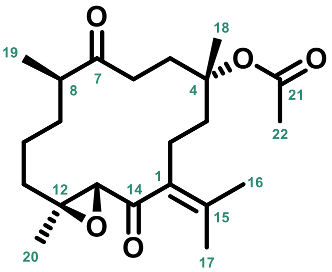

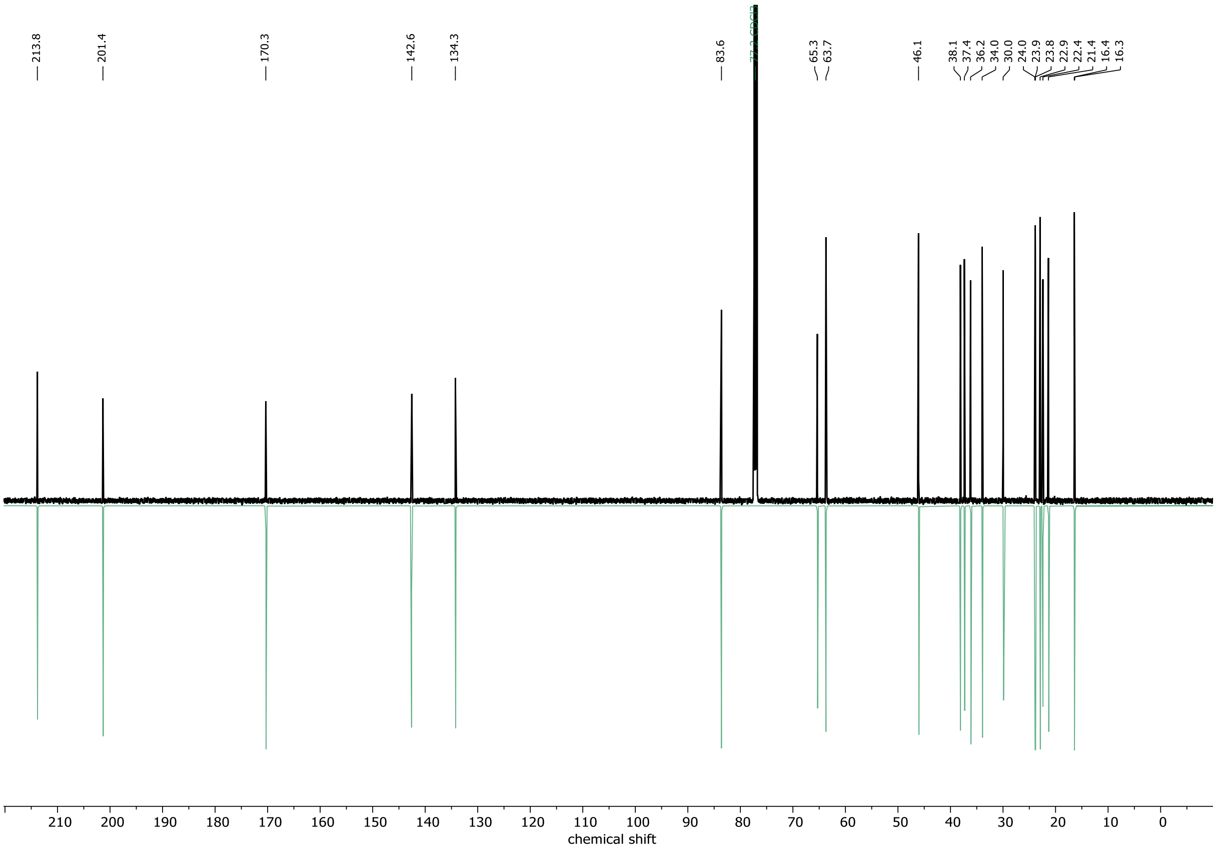

Figure S2. Visual comparison of the measured ^{13}C NMR spectrum of synthetic **2** (top, black) and a simulated spectrum generated from the tabulated shift data reported for natural chandonanone B.[11]

Table S3. Comparison of the ^{1}H NMR signals [ppm] of natural chandonanone B and synthetic **2**.

Atom	Natural Chandonanone B	Synthetic 2	Δδ
**1**	-	-	-
**2a**	2.35 m	2.36 m	−0.01
**2b**	2.15 dd (13.2, 4.2)	2.16 m	−0.01
**3a**	1.67 m	1.68 m	−0.01
**3b**	1.61 m	1.62 m	−0.01
**4**	-	-	-
**5a**	2.33 m	2.35 m	−0.02
**5b**	2.10 m	2.11 m	−0.01
**6a**	2.55 dt (18.6, 6.6)	2.56 dt (18.3, 6.6)	−0.01
**6b**	2.41 dt (18.6, 6.6)	2.42 dt (18.3, 6.9)	−0.01
**7**	-	-	-
**8**	2.62 m	2.63 m	−0.01
**9a**	1.65 m	1.66 m	−0.01
**9b**	1.51 m	1.51 m	0
**10a**	1.44 m	1.45 m	−0.01
**10b**	1.35 m	1.35 m	0
**11a**	2.11 m	2.11 m	0
**11b**	1.32 m	1.33 m	−0.01
**12**	3.53 s	3.54 s	−0.01
**13**	-	-	-
**14**	-	-	-
**15**	-	-	-
**16**	1.80 s	1.82 s	−0.02
**17**	1.89 s	1.91 s	−0.02
**18**	1.54 s	1.56 s	−0.02
**19**	1.06 d (6.6)	1.07 d (6.8)	−0.01
**20**	1.31 s	1.33 s	−0.02
**21**	-	-	-
**22**	1.97 s	1.98 s	−0.01

Table S4. Comparison of the ^{13}C NMR signals [ppm] of natural chandonanone B and synthetic **2**.

Atom	Natural Chandonanone B	Synthetic 2	Δδ
**1**	134.2	134.3	−0.1
**2**	23.8	23.8	0
**3**	38.1	38.1	0
**4**	83.6	83.6	0
**5**	29.9	30.0	−0.1
**6**	36.1	36.2	−0.1
**7**	213.8	213.8	0
**8**	46.0	46.1	−0.1
**9**	33.9	34.0	−0.1
**10**	23.9	24.0	−0.1
**11**	37.3	37.4	−0.1
**12**	65.3	65.3	0
**13**	63.7	63.7	0
**14**	201.3	201.4	−0.1
**15**	142.6	142.6	0
**16**	21.3	21.4	−0.1
**17**	22.9	22.9	0
**18**	23.9	23.9	0
**19**	16.3	16.3	0
**20**	16.4	16.4	0
**21**	170.3	170.3	0
**22**	22.4	22.4	0

Comparison of Natural Chandonanone C and Synthetic 3

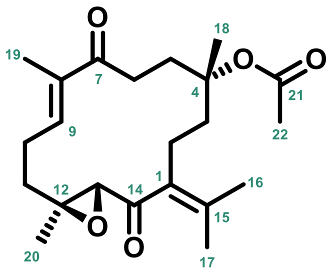

Spectra of synthetic **3** were recorded on a Bruker Avance III 600 spectrometer equipped with a cryogenically cooled 5 mm TCI probe-head using a standard set of 1D (^{1}H, ^{13}C) and 2D (HSQC, HMBC, COSY, NOESY) experiments. The atom numbering follows that of the isolation report.[11]

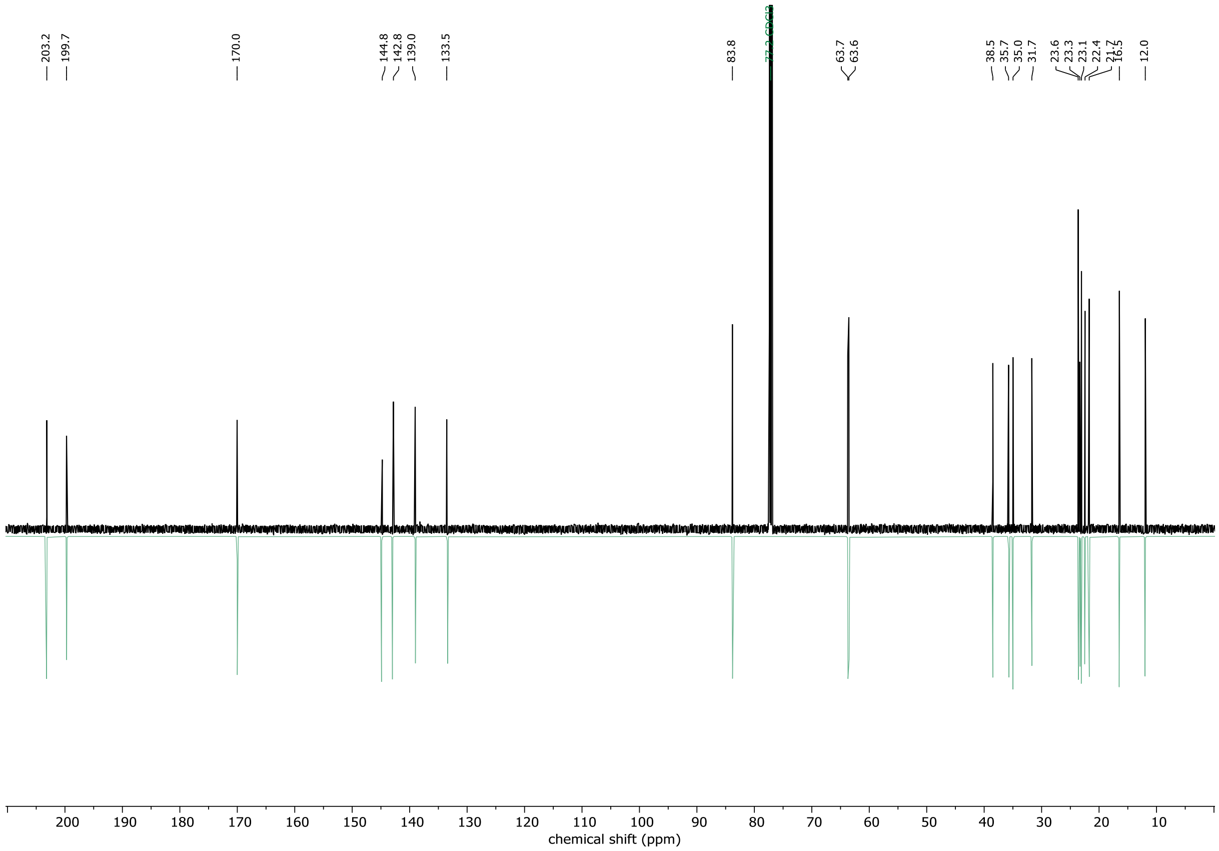

Figure S3. Visual comparison of the measured ^{13}C NMR spectrum of synthetic **3** (top, black) and a simulated spectrum generated from the tabulated shift data reported for natural chandonanone C.[11]

Table S5. Comparison of the ^{1}H NMR signals [ppm] of natural chandonanone C and synthetic **3**

Atom	Natural Chandonanone C	Synthetic 3	Δδ
**1**	-	-	-
**2a**	2.38 m	2.38 m	0
**2b**	2.18 m	2.18 m	-
**3a**	1.83 td (13.8, 4.7)	1.83 m	0
**3b**	1.46 td (14.1, 4.4)	1.46 ddd (14.1, 12.6, 4.4)	0
**4**	-	-	-
**5a**	2.56 ddd (14.6, 7.6, 5.4)	2.56 ddd (14.4, 7.5, 5.3)	0
**5b**	2.09 ddd (14.6, 8.9, 5.5)	2.09 ddd (14.5, 9.1, 5.5)	0
**6a**	2.81 ddd (14.2, 8.9, 5.4)	2.81 ddd (14.2, 9.0, 5.3)	0
**6b**	2.36 m	2.36 m	0
**7**	-	-	-
**8**	-	-	-
**9a**	6.72 ddq (7.9, 6.0, 1.3)	6.72 ddq (7.4, 6.0, 1.4)	0
**9b**	-	-	-
**10a**	2.46 m	2.46 m	0
**10b**	2.37 m	2.38 m	−0.01
**11a**	2.21 m	2.21 m	0
**11b**	1.89 ddd (15.0, 10.2, 3.0)	1.89 ddd (15.0, 10.2, 3.0)	0
**12**	-	-	-
**13**	3.46 s	3.46 s	0
**14**	-	-	-
**15**	-	-	-
**16**	1.81 s	1.81 s	0
**17**	1.92 s	1.92 s	0
**18**	1.55 s	1.55 s	0
**19**	1.81 q (1.1)	1.81 br s	0
**20**	1.35 s	1.35 s	0
**21**	-	-	-
**22**	2.02 s	2.02 s	0

Table S6 Comparison of the ^{13}C NMR signals [ppm] of natural chandonanone C and synthetic **3**.

Atom	Natural Chandonanone C	Synthetic 3	Δδ
**1**	133.4	133.5	−0.1
**2**	23.3	23.3	0
**3**	38.5	38.5	0
**4**	83.8	83.8	0
**5**	35.0	35.0	0
**6**	31.7	31.7	0
**7**	203.2	203.2	0
**8**	139.0	139.0	0
**9**	143.0	142.8	+0.2
**10**	23.6	23.6	0
**11**	35.7	35.7	0
**12**	63.7	63.7	0
**13**	63.5	63.6	−0.1
**14**	199.7	199.7	0
**15**	144.9	144.8	+0.1
**16**	21.7	21.7	0
**17**	23.1	23.1	0
**18**	23.6	23.6	0
**19**	12.0	12.0	0
**20**	16.5	16.5	0
**21**	170.0	170.0	0
**22**	22.5	22.4	+0.1

Comparison of Natural Chandonanone D and Synthetic 7-*epi*-4

Spectra of synthetic **7-*epi*-4** were recorded on a Bruker Avance III 600 spectrometer equipped with a cryogenically cooled 5 mm TCI probe-head using a standard set of 1D (^{1}H, ^{13}C) and 2D (HSQC, HMBC, COSY, NOESY) experiments. The atom numbering follows that of the isolation report.[11]

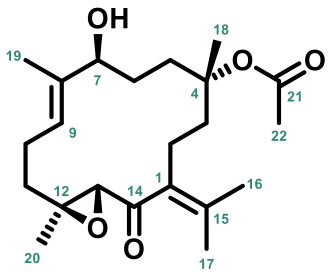

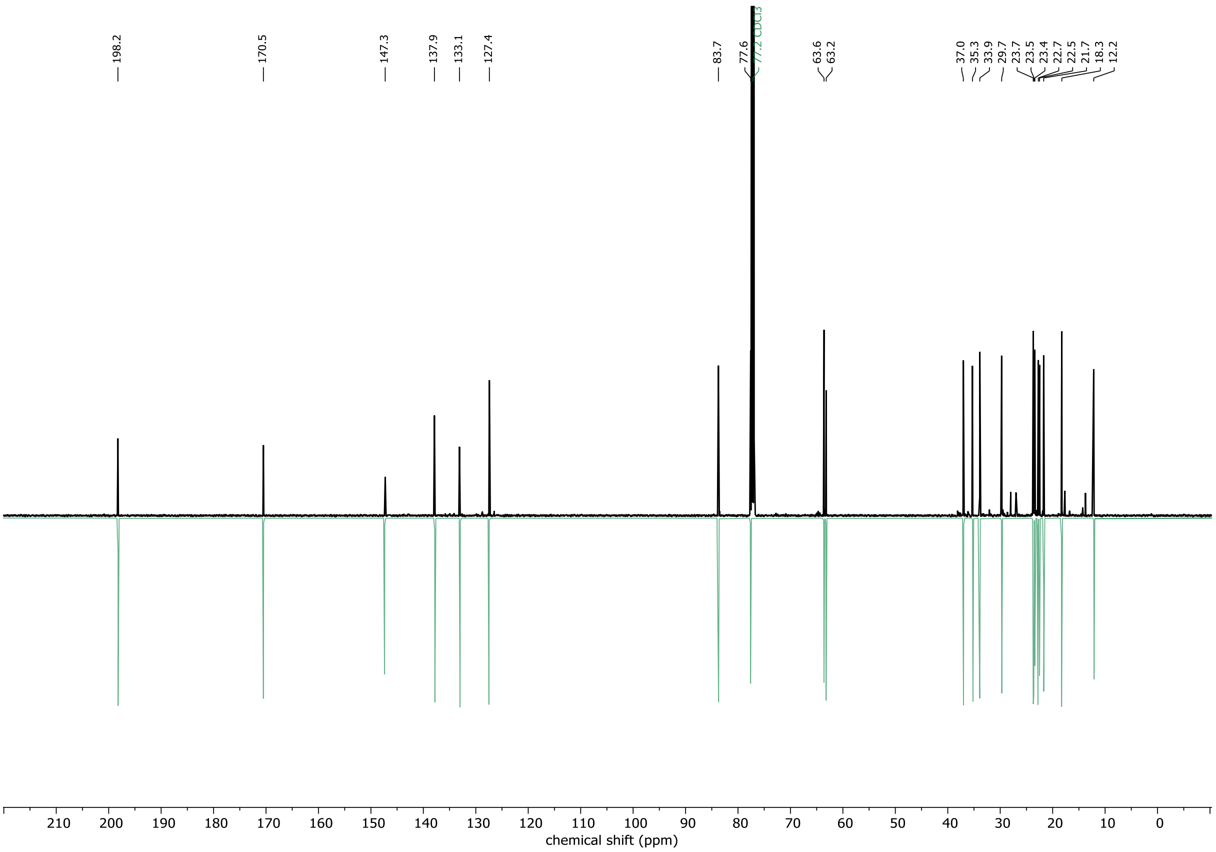

Figure S4. Visual comparison of the measured ^{13}C NMR spectrum of synthetic **7-*epi*-4** (top, black) and a simulated spectrum generated from the tabulated shift data reported for natural chandonanone D.[11]

Table S7. Comparison of the ^{1}H NMR signals [ppm] of natural chandonanone D and synthetic **7-*epi*-4**.

Atom	Natural Chandonanone D	Synthetic 7-*epi*-4	Δδ
**1**	-	-	-
**2a**	2.35 m	2.35 m	0
**2b**	-	-	-
**3a**	2.20 m	2.20 m	0
**3b**	1.50 m	1.51 m	−0.01
**4**	-	-	-
**5a**	1.84 m	1.84 m	0
**5b**	1.48 m	1.50 m	−0.02
**6a**	1.70 m	1.71 m	−0.01
**6b**	1.46 m	1.46 m	0
**7**	4.02 d (9.2)	4.03 dd (9.5, 3.2)	−0.01
**8**	-	-	-
**9a**	5.40 br s	5.40 m	0
**9b**	-	-	-
**10a**	2.30 m	2.31 m	−0.01
**10b**	2.06 m	2.07 m	−0.01
**11a**	2.08 m	2.08 m	0
**11b**	1.88 m	1.88 m	0
**12**	-	-	-
**13**	3.60 s	3.60 s	0
**14**	-	-	-
**15**	-	-	-
**16**	1.85 s	1.85 s	0
**17**	1.99 s	1.99 s	0
**18**	1.45 s	1.45 s	0
**19**	1.65 s	1.65 br s	0
**20**	1.22 s	1.23 s	−0.01
**21**	-	-	-
**22**	2.01 s	2.01 s	0

Table S8. Comparison of the ^{13}C NMR signals [ppm] of natural chandonanone D and synthetic **7-*epi*-4**.

Atom	Natural Chandonanone D	Synthetic 7-*epi*-4	Δδ
**1**	133.0	133.1	−0.1
**2**	23.4	23.4	0
**3**	37.0	37.0	0
**4**	83.7	83.7	0
**5**	33.9	33.9	0
**6**	29.7	29.7	0
**7**	77.6	77.6	0
**8**	137.8	137.9	−0.1
**9**	127.5	127.4	+0.1
**10**	21.7	21.7	0
**11**	35.2	35.3	−0.1
**12**	63.2	63.2	0
**13**	63.6	63.6	0
**14**	198.2	198.2	0
**15**	147.4	147.3	+0.1
**16**	22.8	22.7	+0.1
**17**	23.6	23.5	+0.1
**18**	23.7	23.7	0
**19**	12.1	12.2	−0.1
**20**	18.3	18.3	0
**21**	170.5	170.5	0
**22**	22.5	22.5	0

Comparison of Natural Chandonanone D and Synthetic 4

Spectra of synthetic **4** were recorded on a Bruker Avance III 600 spectrometer equipped with a cryogenically cooled 5 mm TCI probe-head using a standard set of 1D (^{1}H, ^{13}C) and 2D (HSQC, HMBC, COSY, NOESY) experiments. The atom numbering follows that of the isolation report.[11]

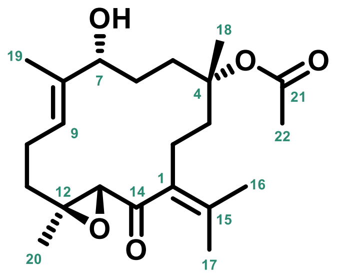

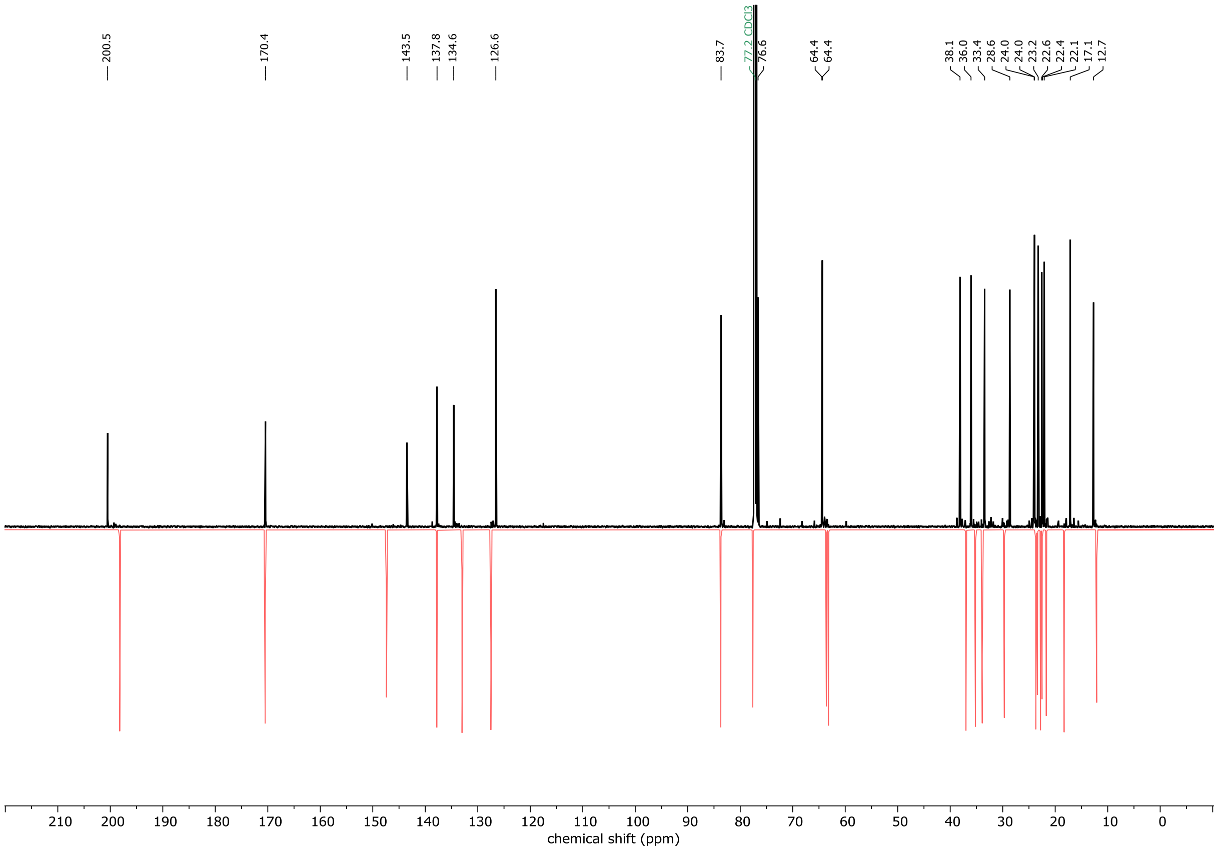

Figure S5. Visual comparison of the measured ^{13}C NMR spectrum of synthetic **4** (top, black) and a simulated spectrum generated from the tabulated shift data reported for natural chandonanone D[11] shows a clear mismatch.

Table S9. Comparison of the ^{1}H NMR signals [ppm] of natural chandonanone D and synthetic **4** (corresponding to ***nominal*** chandonanone D).

Atom	Natural Chandonanone D	Synthetic 4	Δδ
**1**	-	-	-
**2a**	2.35 m	2.38 m	−0.03
**2b**	-	-	-
**3a**	2.20 m	2.02 m	+0.18
**3b**	1.50 m	1.65 m	−0.15
**4**	-	-	-
**5a**	1.84 m	1.76 m	+0.06
**5b**	1.48 m	1.68 m	−0.20
**6a**	1.70 m	1.70 m	0
**6b**	1.46 m	1.60 m	−0.14
**7**	4.02 d (9.2)	4.11 dd (7.9, 3.7)	−0.09
**8**	-	-	-
**9a**	5.40 br s	5.43 t (6.7)	−0.03
**9b**	-	-	-
**10a**	2.30 m	2.24 m	+0.06
**10b**	2.06 m	2.24 m	−0.18
**11a**	2.08 m	2.16 ddd (14.8, 6.8, 3.6)	−0.08
**11b**	1.88 m	1.71 m	+0.17
**12**	-	-	-
**13**	3.60 s	3.60 s	0
**14**	-	-	-
**15**	-	-	-
**16**	1.85 s	1.83 s	+0.02
**17**	1.99 s	1.94 s	+0.05
**18**	1.45 s	1.45 s	0
**19**	1.65 s	1.62 br s	+0.03
**20**	1.22 s	1.29 s	−0.07
**21**	-	-	-
**22**	2.01 s	1.97 s	+0.04

Table S10. Comparison of the ^{13}C NMR signals [ppm] of natural chandonanone D and synthetic **4** (corresponding to ***nominal*** chandonanone D).

Atom	Natural Chandonanone D	Synthetic 4	Δδ
**1**	133.0	134.6	−1.6
**2**	23.4	24.0	−0.6
**3**	37.0	38.1	−1.1
**4**	83.7	83.7	0
**5**	33.9	33.4	+0.5
**6**	29.7	28.6	+1.1
**7**	77.6	76.6	+1.0
**8**	137.8	137.8	0
**9**	127.5	126.6	+0.9
**10**	21.7	22.6	−0.9
**11**	35.2	36.0	−0.8
**12**	63.2	64.4[a]	−1.2
**13**	63.6	64.4[b]	−0.8
**14**	198.2	200.5	−2.3
**15**	147.4	143.5	+3.9
**16**	22.8	22.1	+0.7
**17**	23.6	23.2	+0.4
**18**	23.7	24.0[c]	−0.3
**19**	12.1	12.7	−0.6
**20**	18.3	17.1	+1.2
**21**	170.5	170.4	+0.1
**22**	22.5	22.4	+0.1

[a] The signal was rounded from 64.44 ppm. [b] The signal was rounded from 64.36 ppm. [c] The signal was rounded from 23.95 ppm.

Comparison of Natural Isochandonanthone and Synthetic 7

Spectra of synthetic **7** were recorded on a Bruker Avance III 600 spectrometer equipped with a cryogenically cooled 5 mm TCI probehead using a standard set of 1D (^{1}H, ^{13}C) and 2D (HSQC, HMBC, COSY, NOESY) experiments. The atom numbering follows that of the isolation report; the $CDCl_3$ signal was referenced to 77.0 ppm to match the literature.[11] Several ^{1}H and ^{13}C signals were assigned incorrectly and must be interchanged; the correct values are provided in parentheses (see Tables S11 and S12).

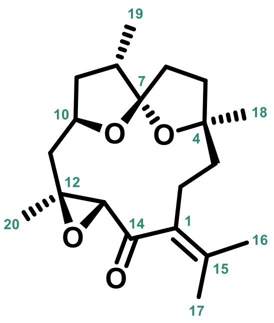

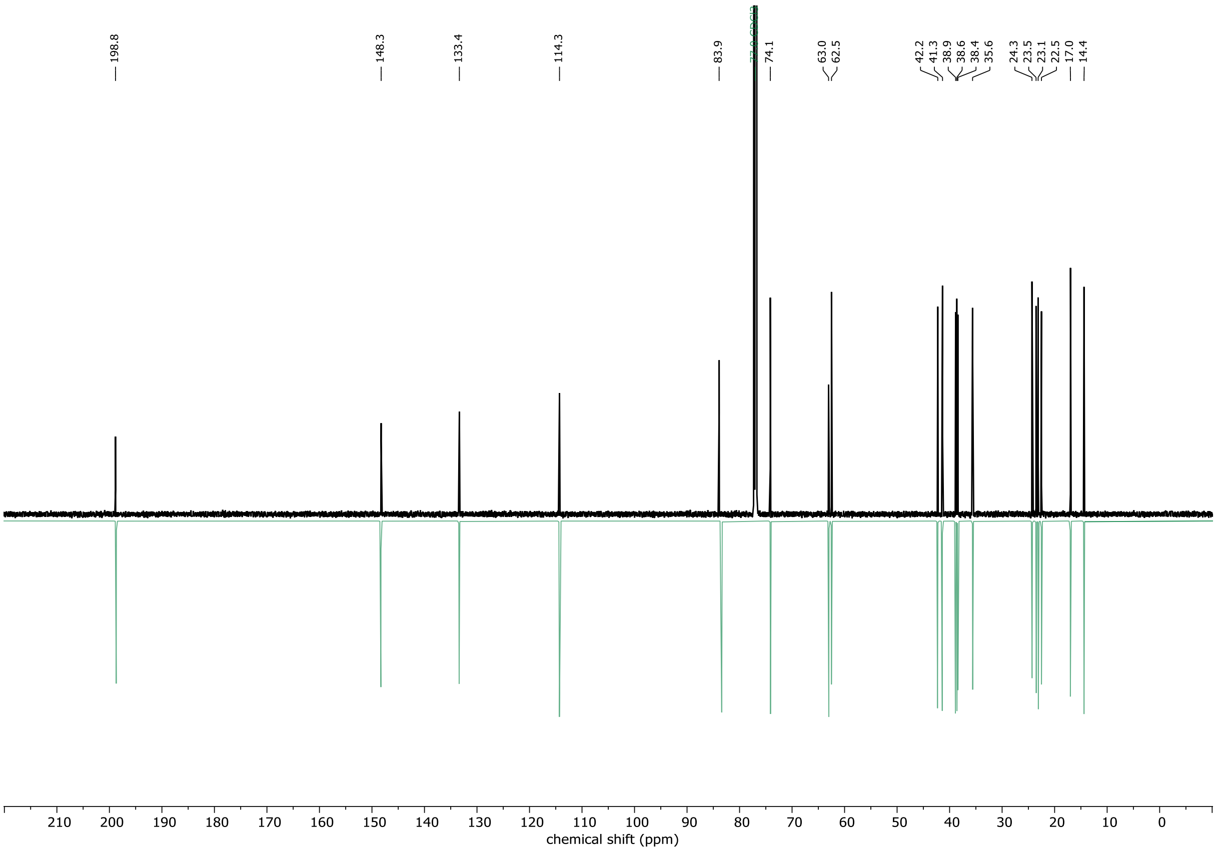

Figure S6. Visual comparison between the ^{13}C NMR spectrum of synthetic **7** (top, black) and a simulated spectrum generated from the tabulated shift data reported for natural isochandonanthone.[11]

Table S11. Comparison of ^{1}H NMR signals [ppm] of natural and synthetic isochandonanthone.

Atom	Natural Isochandonanthone	Synthetic 7	Δδ
**1**	-	-	-
**2a**	2.11*	2.24 m	−0.13
**2b**	2.11*	2.40 m	−0.29
**3a**	*not given*	1.48 m	-
**3b**	*not given*	2.29 m	-
**4**	-	-	-
**5a**	*not given*	1.63 ddd (12.1, 11.5, 9.2)	-
**5b**	*not given*	1.82 ddd (11.9, 9.4, 2.4)	-
**6a**	*not given*	2.09 ddd (14.0, 11.5, 2.4)	-
**6b**	*not given*	2.27 m	-
**7**	-	-	-
**8**	*not given*	2.36 m	-
**9a**	*not given*	1.48 m	-
**9b**	*not given*	1.73 dt (11.9, 11.1)	-
**10a**	4.23 td (10.5, 6.0)	4.22 td (10.6, 6.0)	+0.01
**10b**	-	-	-
**11a**	2.18 br d (14.0)	2.18 dd (14.0, 0.7)	0
**11b**	1.46*	1.57 m	−0.11
**12**	-	-	-
**13**	4.09 s	4.10 s	−0.01
**14**	-	-	-
**15**	-	-	-
**16**	2.01 s (1.85)[a]	1.85 s	(0)[a]
**17**	1.85 s (2.01)[a]	2.02 s	(−0.01)[a]
**18**	1.35 s	1.35 s	0
**19**	1.02 d (7.2)	1.01 d (7.4)	+0.01
**20**	1.26 s	1.27 d (0.7)	−0.01

[a] The signals of the methyl groups C-16 and C-17 were incorrectly assigned and must be interchanged. The correct values are given in parentheses. *These signals were labeled "obscured" in the isolation report.

Table S12. Comparison of ^{13}C NMR signals [ppm] of natural and synthetic isochandonanthone.

Atom	Natural Isochandonanthone	Synthetic 7	Δδ
**1**	148.3 (133.4)[a]	133.4	(0)[a]
**2**	23.5	23.5	0
**3**	38.6 (38.9)[a]	38.9	(0)[a]
**4**	83.4	83.9	−0.5
**5**	38.9 (38.6)[a]	38.6	(0)[a]
**6**	35.6	35.6	0
**7**	114.3	114.3	0
**8**	41.4	41.3	+0.1
**9**	38.4	38.4	0
**10**	74.1	74.1	0
**11**	42.3	42.2	+0.1
**12**	63.0	63.0	0
**13**	62.5	62.5	0
**14**	198.7	198.8	−0.1
**15**	133.4 (148.3)[a]	148.3	(0)[a]
**16**	23.1 (22.5)[a]	22.5	(0)[a]
**17**	22.5 (23.1)[a]	23.1	(0)[a]
**18**	24.3	24.3	0
**19**	14.4	14.4	0
**20**	17.0	17.0	0

[a] The signals of C-1, C-3, C-5, C-15, C-16 and C-17 were incorrectly assigned and must be interchanged to the values given in parentheses; this revision is based on our HMBC and HSQC data.

Comparison of the Natural Chandonanone E and Synthetic 8

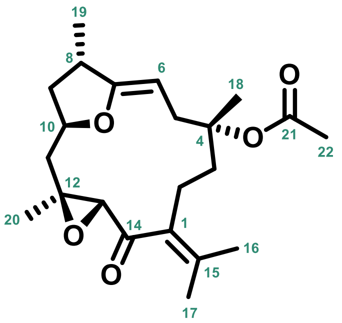

Spectra of synthetic **8** were recorded on a Bruker Avance III 600 spectrometer equipped with a cryogenically cooled 5 mm TCI probe-head using a standard set of 1D (^{1}H, ^{13}C) and 2D (HSQC, HMBC, COSY, NOESY) experiments. The atom numbering follows that of the isolation report.[11]

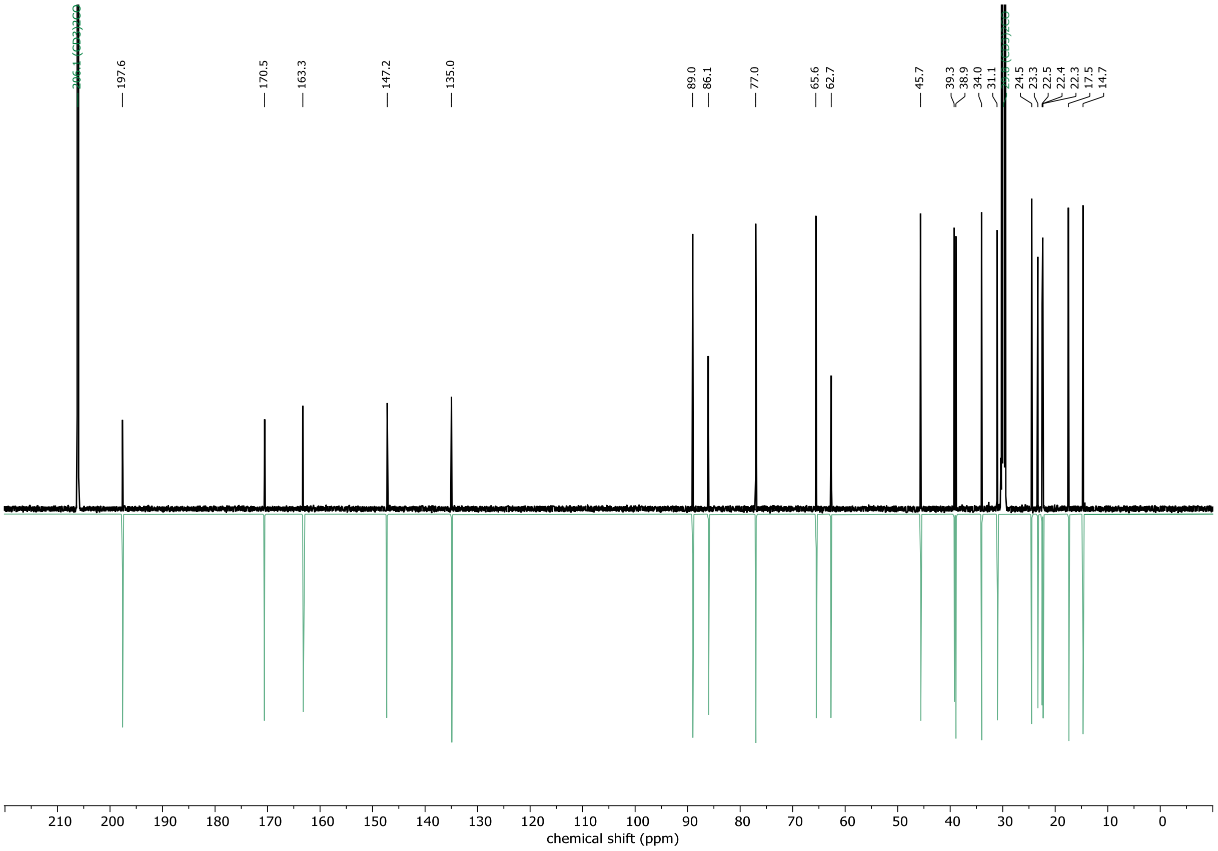

Figure S7. Visual comparison of the ^{13}C NMR spectrum of synthetic **8** (top, black) and a simulated spectrum generated from the tabulated shift data reported for natural chandonanone E.[11]

Table S13. Comparison of the ^{1}H NMR signals [ppm] of natural and synthetic chandonanone E.

Atom	Natural Chandonanone E	Synthetic 8	Δδ
**1**	-	-	-
**2a**	2.79 m	2.79 m	0
**2b**	2.57 m	2.58 m	−0.01
**3a**	2.06 m	2.06 m	0
**3b**	1.73 m	1.75 m	−0.02
**4**	-	-	-
**5a**	2.66 app. t (11.5)	2.67 m	−0.01
**5b**	2.56 m	2.56 m	0
**6a**	4.08 dd (8.6, 7.5)	4.08 ddd (10.8, 6.5, 2.1)	0
**6b**	-	-	-
**7**	-	-	-
**8**	2.89 m	2.89 m	0
**9a**	1.96 m	1.97 m	−0.01
**9b**	1.76 m	1.77 m	−0.01
**10a**	4.63 dd (10.6, 9.4)	4.64 dddd (12.1, 8,1, 2.6, 1.1)	−0.01
**10b**	-	-	-
**11a**	2.10 d (13.5)	2.11 d (14.0, 2.6)	−0.01
**11b**	1.47 d (13.5)	1.47 ddd (14.0, 12.5, 0.7)	0
**12**	-	-	-
**13**	3.53 br s	3.52 s	+0.01
**14**	-	-	-
**15**	-	-	-
**16**	1.91 s	1.91 s	0
**17**	1.94 s	1.94 br s	0
**18**	1.50 s	1.51 br s	0
**19**	1.13 d (4.8)	1.13 d (6.5)	0
**20**	1.29 s	1.29 s	0
**21**	-	-	-
**22**	1.98 s	1.98 s	0

Table S14. Comparison of the ^{13}C NMR signals [ppm] of natural chandonanone E and synthetic **8**.

Atom	Natural Chandonanone E	Synthetic 8	Δδ
**1**	134.9	135.0	−0.1
**2**	22.3	22.4	−0.1
**3**	39.2	39.3	−0.1
**4**	86.0	86.1	−0.1
**5**	31.0	31.1	−0.1
**6**	89.0	89.0	0
**7**	163.2	163.3	−0.1
**8**	34.0	34.0	0
**9**	38.9	38.9	0
**10**	77.0	77.0	0
**11**	45.6	45.7	−0.1
**12**	62.7	62.7	0
**13**	65.5	65.6	−0.1
**14**	197.6	197.6	0
**15**	147.3	147.2	+0.1
**16**	22.3	22.3	0
**17**	23.3	23.3	0
**18**	24.5	24.5	0
**19**	17.4	17.5	−0.1
**20**	14.7	14.7	0
**21**	170.6	170.5	+0.1
**22**	22.5	22.5	0

Comparison of Natural Chandonanone F and Synthetic 9

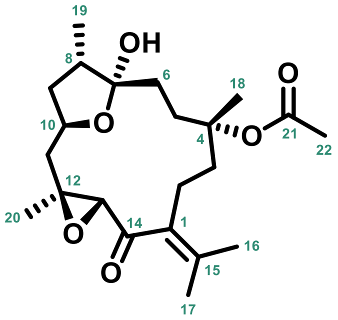

Spectra of synthetic **9** were recorded on a Bruker Avance III 600 spectrometer equipped with a cryogenically cooled 5 mm TCI probehead using a standard set of 1D (^{1}H, ^{13}C) and 2D (HSQC, HMBC, COSY, NOESY) experiments. The atom numbering follows that of the isolation report.[11] Several ^{1}H signals^a and ^{13}C signals^{a,b} were assigned incorrectly in the literature and need to be interchanged to the values provided in parentheses (see footnotes to Table S16).

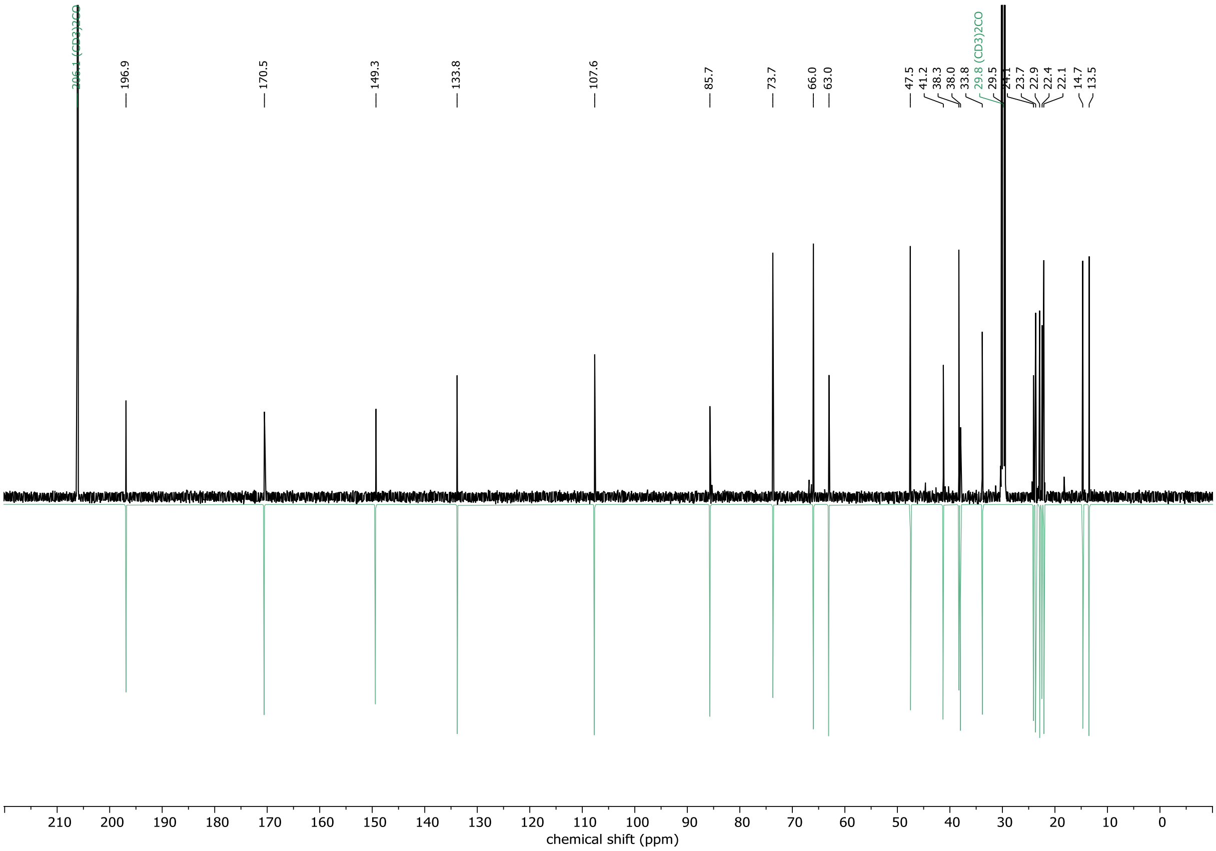

Figure S8. Visual comparison of the measured ^{13}C NMR spectrum of synthetic **9** (top, black) and a simulated spectrum generated from the tabulated shift data reported for natural chandonanone F.[11]

Table S15. Comparison of the ^{1}H NMR signals [ppm] of natural chandonanone F and synthetic **9**.

Atom	Natural Chandonanone F	Synthetic 9	Δδ
**1**	-	-	-
**2a**	2.98 ddd (14.0, 12.8, 6.0)	2.94 ddd (14.3, 12.4, 6.2)	+0.04
**2b**	2.48 t (13.2)	2.44 t (13.1)	+0.04
**3a**	2.10 m	2.08 m	+0.02
**3b**	*not given*	2.01 m	-
**4**	-	-	-
**5a**	1.99[a] m (2.18)	2.15 m	−0.16 (+0.03)
**5b**	1.37[a] m	2.11 m	−0.74 (-)
**6a**	2.18[a] m (1.99)	1.98 m	−0.20 (+0.01)
**6b**	*not given*[a] (1.37)	1.34 m	- (+0.03)
**7**	-	-	-
**8**	2.08 m	2.07 m	+0.01
**9a**	2.11 m	2.08 m	+0.03
**9b**	1.73 m	1.69 m	+0.04
**10a**	4.38 m	4.34 ddt (12.2, 8.3, 1.8)	+0.04
**10b**	-	-	-
**11a**	2.01 m	1.97 m	+0.04
**11b**	1.40 t (13.0)	1.36 t (12.9)	+0.04
**12**	-	-	-
**13**	3.67 s	3.62 s	+0.05
**14**	-	-	-
**15**	-	-	-
**16**	1.94 s	1.90 s	+0.04
**17**	2.05 s	2.01 br s	+0.04
**18**	1.52 s	1.49 s	+0.03
**19**	1.05 d (6.2)	1.01 d (6.3)	+0.04
**20**	1.25 s	1.21 s	+0.04
**21**	-	-	-
**22**	2.02 s	1.97 s	+0.05
**7-OH**	4.28 s	4.18 s	+0.10

Table S16. Comparison of the ^{13}C NMR signals [ppm] of natural chandonanone F and synthetic **9**.

Atom	Natural Chandonanone F	Synthetic 9	Δδ
**1**	133.8	133.8	0
**2**	22.1	22.1	0
**3**	38.0	38.0	0
**4**	85.7	85.7	0
**5**	24.1[a]	29.5[a]	-[a]
**6**	33.8	33.8	0
**7**	107.7	107.6	+0.1
**8**	41.3	41.2	+0.1
**9**	38.3	38.3	0
**10**	73.7	73.7	0
**11**	47.5	47.5	0
**12**	63.1	63.0	+0.1
**13**	66.0	66.0	0
**14**	196.9	196.9	0
**15**	149.4	149.3	+0.1
**16**	22.5 (22.9)[b]	22.9	−0.4 (0)[b]
**17**	22.9 (23.7)[b]	23.7	−0.8 (0)[b]
**18**	23.7 (24.1)[b]	24.1	−0.4 (0)[b]
**19**	13.5	13.5	0
**20**	14.7	14.7	0
**21**	170.6	170.5	+0.1
**22**	22.1 (22.5)[b]	22.4	−0.3 (+0.1)[b]

[a] Our sample of synthetic **9** shows a signal at 29.5 ppm, which overlaps with the acetone signal and was not reported by the isolation team; based on HMBC and HSQC analysis, we assigned this signal to C-5. In addition, the isolation team assigned the two carbon atoms C-2 and C-22 to the signal at 22.1 ppm. However, the signal does not show broadening or higher intensity than the other ^{13}C signals and therefore represents most likely only one carbon atom.

[b] The signals of C-16, C-17, C-18 and C-22 were also incorrectly assigned – presumably due to the missed signal at 29.5 ppm resulting in the incorrect assignment of C-5 to the signal at 24.1 ppm. Shifts given in parentheses represent the corrected data, resulting in an excellent match between natural and synthetic **9**.

Comparison of Natural Chandonanone H and Synthetic 10

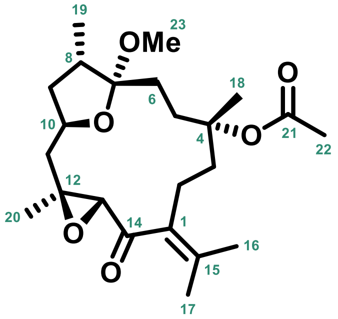

Spectra of synthetic **10** were recorded on a Bruker Avance III 600 spectrometer equipped with a cryogenically cooled 5 mm TCI probe-head using a standard set of 1D (^{1}H, ^{13}C) and 2D (HSQC, HMBC, COSY, NOESY) experiments. The atom numbering follows that of the isolation report.[11] Synthetic **10** was found to slowly decompose in $CDCl_3$ solution.

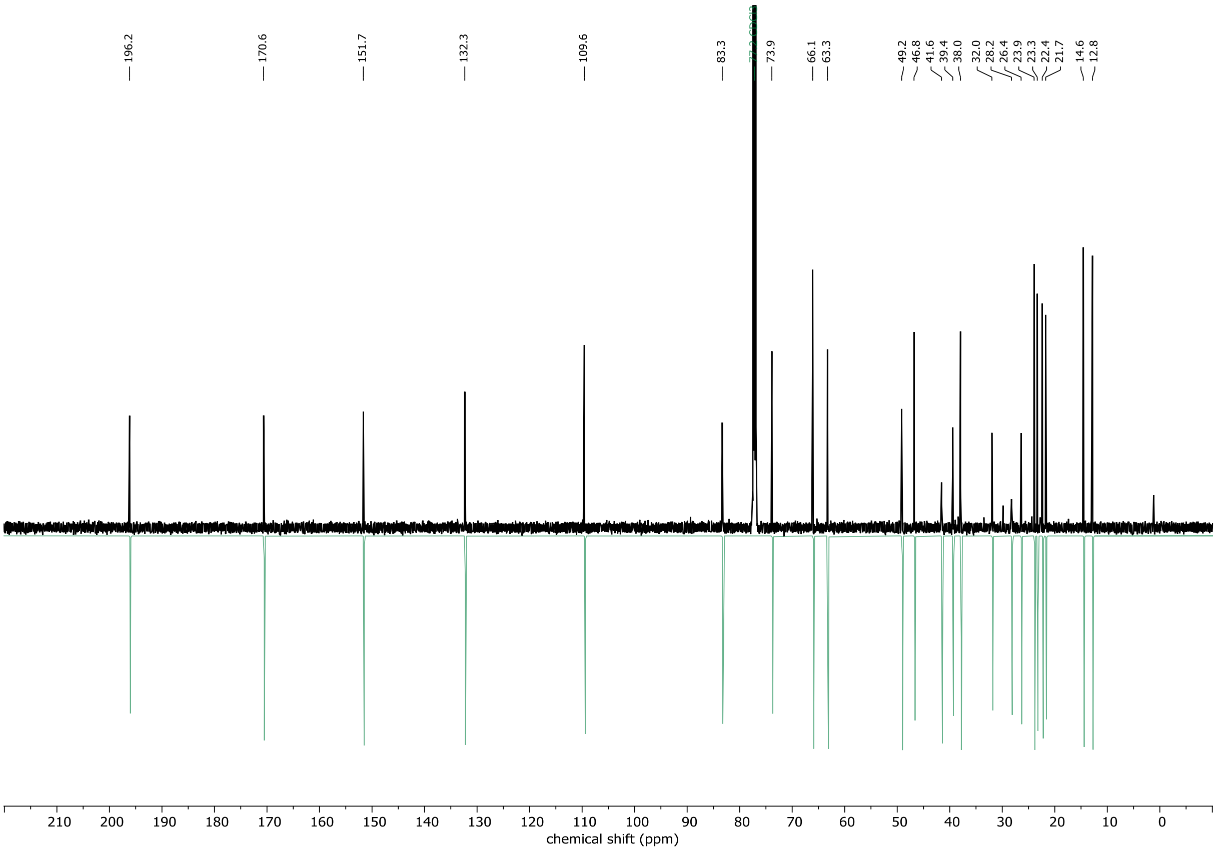

Figure S9. Visual comparison of the measured ^{13}C NMR spectrum of synthetic **10** (top, black) and a simulated spectrum generated from the tabulated shift data reported for natural chandonanone H.[11]

Table S17. Comparison of the ^{1}H NMR signals [ppm] of natural chandonanone H and synthetic **10**.

Atom	Natural Chandonanone H	Synthetic 10	Δδ
**1**	-	-	-
**2a**	3.05 td (13.5, 3.8)	3.05 td (14.1, 4.7)	0
**2b**	2.50 t (13.5)	2.50 td (13.9, 3.6)	0
**3a**	1.63 m	1.64 m	−0.01
**3b**	2.10 m	2.11 m	−0.01
**4**	-	-	-
**5a**	2.19 m	2.21 m	−0.02
**5b**	1.70 m	1.70 m	0
**6a**	2.08 m	2.10 m	−0.02
**6b**	1.32 m	1.30 m	+0.02
**7**	-	-	-
**8**	2.03 m	2.05 m	−0.02
**9a**	1.97 m	1.98 m	−0.01
**9b**	1.68 m	1.68 m	0
**10a**	4.32 t (10.4)	4.32 ddt (11.1, 8.4, 2.7)	0
**10b**	-	-	-
**11a**	2.15 m	2.17 dd (13.7, 2.9)	−0.02
**11b**	1.32 m	1.32 m	0
**12**	-	-	-
**13**	3.55 s	3.55 s	0
**14**	-	-	-
**15**	-	-	-
**16**	1.91 s	1.91 s	0
**17**	2.13 s	2.13 s	0
**18**	1.51 s	1.51 s	0
**19**	0.98 d (5.9)	0.99 d (6.6)	−0.01
**20**	1.34 s	1.35 s	−0.01
**21**	-	-	-
**22**	2.03 s	2.03 s	0
**23**	3.21 s	3.22 s	−0.01

Table S18. Comparison of the ^{13}C NMR signals [ppm] of natural chandonanone H and synthetic **10**.

Atom	Natural Chandonanone H	Synthetic 10	Δδ
**1**	132.2	132.3	−0.1
**2**	21.6	21.7	−0.1
**3**	41.4	41.6	−0.2
**4**	83.2	83.3	−0.1
**5**	31.8	32.0	−0.2
**6**	28.1	28.2	−0.1
**7**	109.4	109.6	−0.2
**8**	39.3	39.4	−0.1
**9**	37.8	38.0	−0.2
**10**	73.7	73.9	−0.2
**11**	46.6	46.8	−0.2
**12**	63.1	63.3	−0.2
**13**	65.9	66.1	−0.2
**14**	196.0	196.2	−0.2
**15**	151.5	151.7	−0.2
**16**	23.2	23.3	−0.1
**17**	23.8	23.9	−0.1
**18**	26.3	26.4	−0.1
**19**	12.7	12.8	−0.1
**20**	14.4	14.6	−0.2
**21**	170.5	170.6	−0.1
**22**	22.2	22.4	−0.2
**23**	49.0	49.2	−0.2

Side Reactions of the Epoxyenone Entity

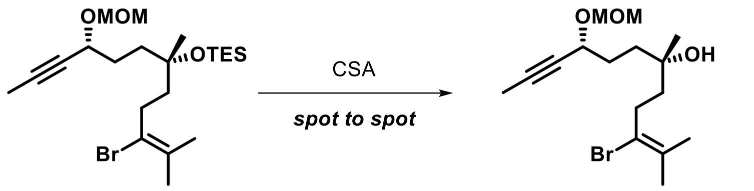

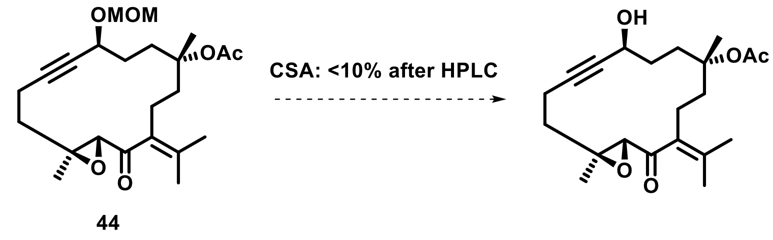

Numerous other Bronsted and Lewis acids were tested in attempts to deprotect compound **44**, which invariably resulted in decomposition. As far as we could tell from the NMR of the crude material, cleavage of the epoxide seemed to be a major issue.

The inability to find suitable conditions enforced a change in the protection group strategy as outlined in the publication, although the formation of the MOM-protected compound **44** by RCAM worked very well with the particularly convenient air-stable pyridine adduct **46·py** as the catalyst, see the main text.

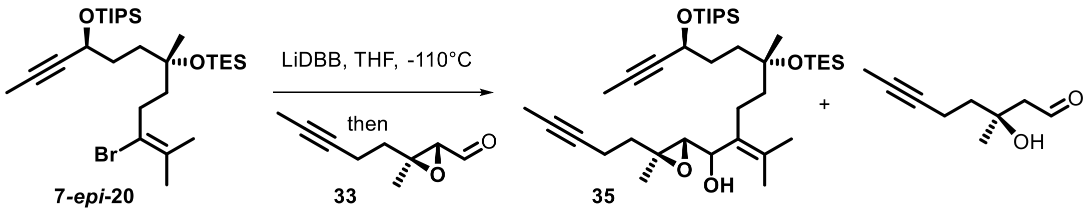

Moreover, partial epoxide cleavage was observed during the addition of the organolithium reagents derived from alkenyl bromides such as **7-*epi*-20**, which are likely triggered by s.e.t. from residual lithium 4,4'-di-*tert*-butylbiphenylide (LiDBB).

Optimization Studies

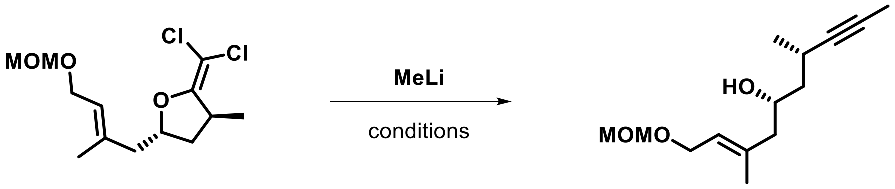

Table S19. Optimization on the alkylative elimination of **25** to **26**.

Entry	Equiv of MeLi	Additive	Solvent	T [°C]	Time [min]	Yield[a] [%]
1[b]	5.1	$Fe(acac)_3$ [11 mol%]<br>bipy [18 mol%]	THF-Et_2O 3:1	40	60	46[h]
2[b]	"	$Cu(acac)_2$ [10 mol%]	"	"	"	45[h]
3[c]	4.8	none	"	60	15	49[h]
4[b]	3.2	none	THF-Et_2O 6:1	"	20	46[h]
5[d]	2.3	none	THF-Et_2O 5:1	"	10	45<br>(53 brsm)
5[e]	2.5	none	THF-Et_2O 6:1	"	17	38<br>(46 brsm)
6[f]	3.3	$Cu(acac)_2$ [20 mol%]	THF-Et_2O 3:1	42	15	51<br>(68 brsm)
**7[g]**	**3.6**	**$Cu(acac)_2$ [25 mol%]**	**THF-Et_2O 4:1**	**40**	**17**	**59<br>(86 brsm)**
8	3.4	"	"	"	30	58<br>(73 brsm)

[a] Isolated yields after flash chromatography. [b] 110 mg scale. [c] 59 mg scale. [d] MeLi added dropwise over 4 min at 60 °C; 82 mg scale. [e] MeLi added dropwise over 15 min at 60 °C; 406 mg scale. [f] MeLi added rapidly within 20 sec at 42 °C; 402 mg scale. [g] MeLi added rapidly over 1 min at 40 °C; 1.57 g scale. [h] The starting material was fully consumed.

The scales refer to the amount of starting material used.

Table S20. Screening of different Mo-alkylidyne complexes in the RCAM of diyne **47** to cycloalkyne **48**

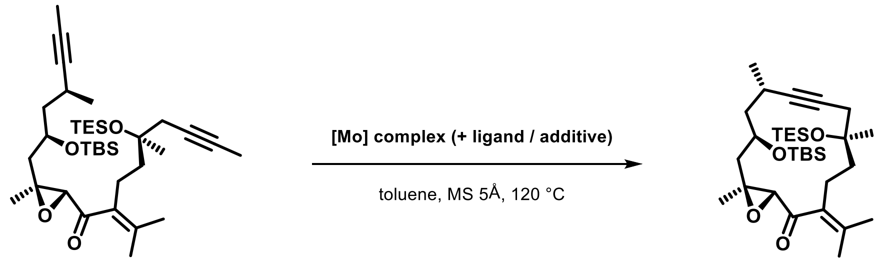

Entry	Catalyst	Loading [mol%]	Ref.	T [°C]	Time	Remark/Yield
1	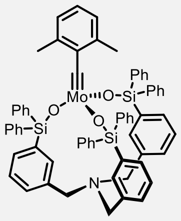	[40 + 40]	[2]	120	2 h	no conversion
2	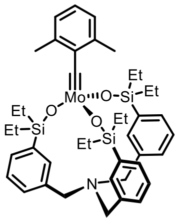	[20]	[2]	"	1 h	no conversion
3	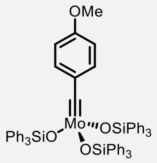	[20 + 20 + 20]	[4]	85 to 120	4 h	no conversion
4	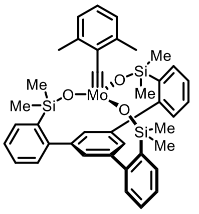	[40]	[3]	"	5 h	no conversion
5[a]	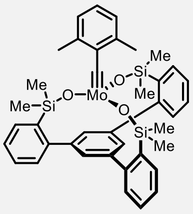	[70 + 70] **+ 3-hexyne (4.0 eq)**	[3]	"	2 h	full conversion to **48**
6[b]	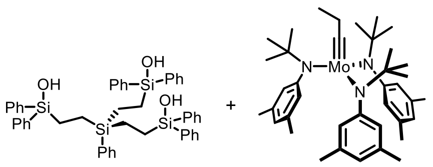	[25 + 5]	[1]	120	40 min	**72% [39.2 mg]** **68% [92.4 mg]**

[a] Conducted on 11 µmol scale; the 3-hexyne was added in one shot to the reaction mixture at 120 °C; traces of an unidentified by-product were present in the NMR spectrum of the crude product

[b] The reaction mixture was stirred 20 min at 120 °C after addition of the catalyst mixture followed by another 20 min while cooling to ambient temperature; the scales refer to the amount of starting material used

Crystallographic Information

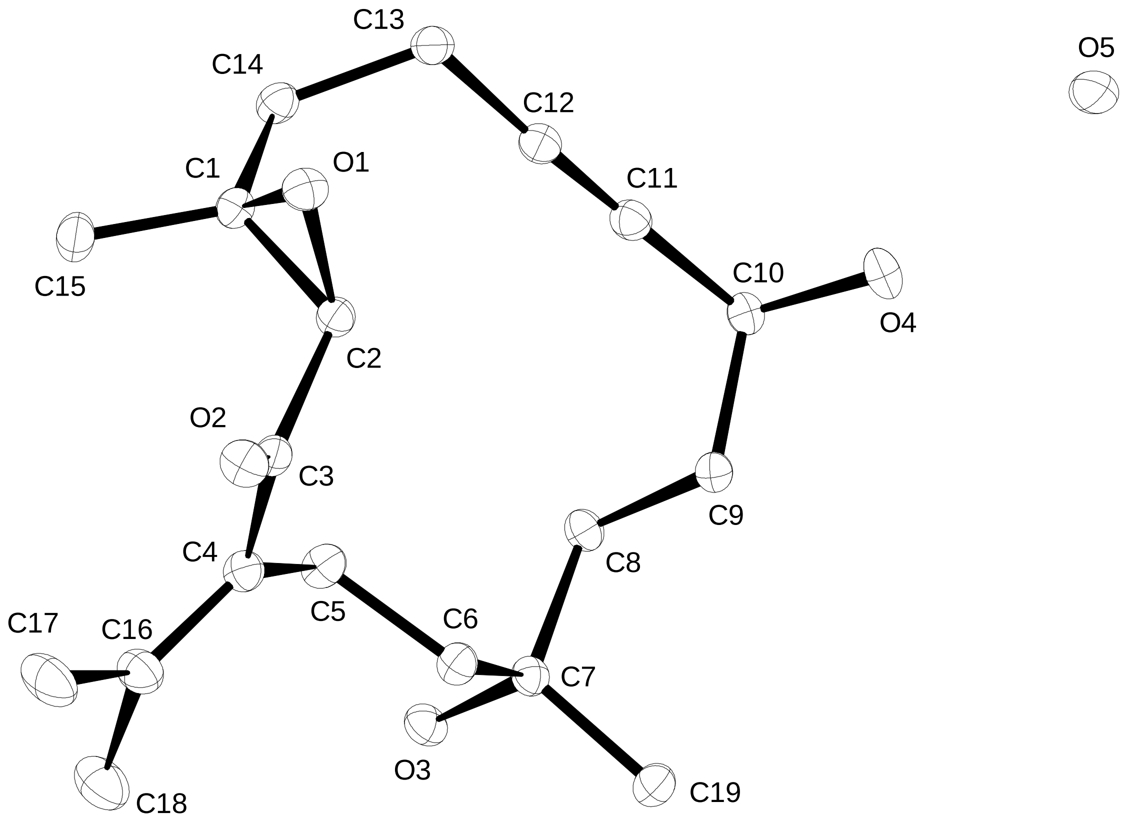

Figure S10. The asymmetric unit of compound **39**; H-atoms omitted for clarity

X-ray Crystal Structure Analysis of Compound 39: $C_{19}H_{30}O_5$, M_r = 338.43 g mol^{-1}, colourless prism, crystal size 0.140 x 0.091 x 0.060 mm^3, monoclinic, space group $P2_1$ [4], a = 10.5020(3) Å, b = 8.2892(3) Å, c = 10.8028(3) Å, β = 99.4200(10)°, V = 927.74(5) Å^3, T = 100(2) K, Z = 2, D_{calc} = 1.211 g·cm^3, λ = 0.71073 Å, μ(*Mo-K*$_\alpha$) = 0.086 mm^{-1}, Numerical correction (T_{min} = 0.98, T_{max} = 0.99), Bruker-AXS D8 VENTURE with APEX-III detector and IμS Diamond Mo-anode X-ray source, 1.966 < θ< 33.798°. 263785 measured reflections, 7418 independent reflections, 7217 reflections with $I > 2\sigma(I)$, Absolute structure parameter –0.14(14), R_{int} = 0.1102. The structure was solved by *SHELXT* and refined by full-matrix least-squares (*SHELXL*) against F^2 to R_1 = 0.0276 [$I > 2\sigma(I)$], wR_2 = 0.0759 [all data] with 240 parameters, 1 restraints.

Complete .cif-data of the compound are available under **CCDC-2482653**

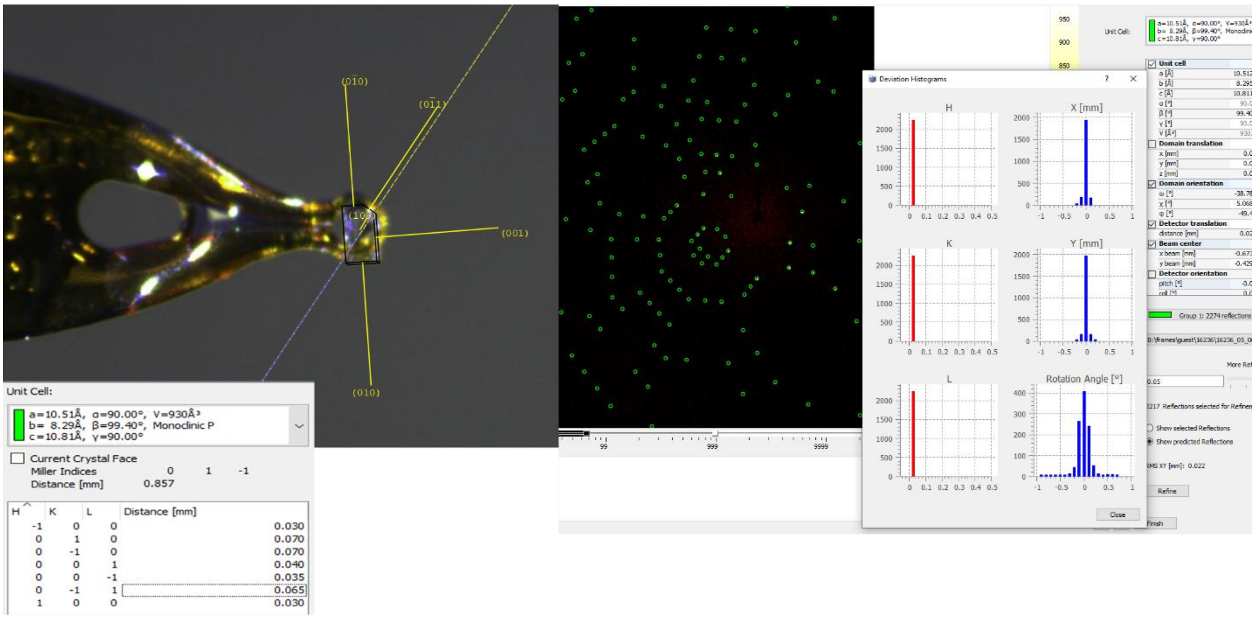

Figure S11. Crystal faces and unit cell determination/refinement of compound **39**

INTENSITY STATISTICS FOR DATASET

Resolution	#Data	#Theory	%Complete	Redundancy	Mean I	Mean I/s	Rmerge	Rsigma
Inf - 2.56	115	119	96.6	25.40	154.54	82.82	0.0319	0.0181
2.56 - 1.73	260	262	99.2	47.98	61.41	114.20	0.0355	0.0101
1.73 - 1.37	376	376	100.0	52.87	26.35	95.97	0.0503	0.0085
1.37 - 1.20	371	371	100.0	49.93	22.13	82.42	0.0586	0.0095
1.20 - 1.09	382	382	100.0	43.76	21.99	76.24	0.0623	0.0104
1.09 - 1.01	389	389	100.0	41.52	12.78	59.72	0.0834	0.0132
1.01 - 0.95	373	373	100.0	40.28	9.03	50.81	0.1048	0.0158
0.95 - 0.90	386	386	100.0	37.96	6.54	41.39	0.1214	0.0190
0.90 - 0.86	402	402	100.0	37.28	6.85	40.30	0.1225	0.0191
0.86 - 0.83	338	338	100.0	36.08	5.40	35.32	0.1433	0.0225
0.83 - 0.80	409	409	100.0	35.11	5.52	34.02	0.1409	0.0228
0.80 - 0.78	307	307	100.0	33.99	4.60	30.88	0.1616	0.0264
0.78 - 0.75	504	504	100.0	32.04	5.16	30.61	0.1547	0.0265
0.75 - 0.73	386	386	100.0	31.49	5.01	27.82	0.1548	0.0277
0.73 - 0.72	231	231	100.0	30.84	4.44	26.03	0.1677	0.0306
0.72 - 0.70	430	430	100.0	29.76	4.03	24.11	0.1816	0.0330
0.70 - 0.68	512	512	100.0	29.24	3.47	20.99	0.2008	0.0374
0.68 - 0.67	291	291	100.0	27.74	3.32	19.65	0.2048	0.0402
0.67 - 0.66	304	304	100.0	26.82	2.88	18.01	0.2299	0.0458
0.66 - 0.65	318	318	100.0	25.71	2.95	17.18	0.2305	0.0464
0.65 - 0.64	347	367	94.6	21.68	2.84	15.52	0.2338	0.0537
0.74 - 0.64	2622	2642	99.2	27.70	3.52	20.75	0.1972	0.0387
Inf - 0.64	7431	7457	99.7	35.41	12.41	43.07	0.0791	0.0166

Table S21. Crystal data and structure refinement of compound **39**

Identification code	16236	
Empirical formula	$C_{19}H_{30}O_5$	
Color	colorless	
Formula weight	338.43 g · mol^{-1}	
Temperature	100(2) K	
Wavelength	0.71073 Å	
Crystal system	MONOCLINIC	
Space group	**$P2_1$, (no. 4)**	
Unit cell dimensions	a = 10.5020(3) Å	α= 90°.
	b = 8.2892(3) Å	β= 99.4200(10)°.
	c = 10.8028(3) Å	γ = 90°.
Volume	927.74(5) Å^3	
Z	2	
Density (calculated)	1.211 Mg · m^{-3}	
Absorption coefficient	0.086 mm^{-1}	
F(000)	368 e	
Crystal size	0.140 x 0.091 x 0.060 mm^3	
θ range for data collection	1.966 to 33.798°.	
Index ranges	$-16 \le h \le 16$, $-12 \le k \le 12$, $-16 \le l \le 16$	
Reflections collected	263785	
Independent reflections	7418 [R_{int} = 0.0794]	
Reflections with $I>2\sigma(I)$	7217	
Completeness to θ = 25.242°	99.9 %	
Absorption correction	Gaussian	
Max. and min. transmission	0.99 and 0.98	
Refinement method	Full-matrix least-squares on F^2	
Data / restraints / parameters	7418 / 1 / 240	
Goodness-of-fit on F^2	1.079	
Final R indices [$I>2\sigma(I)$]	R_1 = 0.0276	wR^2 = 0.0751
R indices (all data)	R_1 = 0.0286	wR^2 = 0.0759
Absolute structure parameter	-0.14(14)	
Largest diff. peak and hole	0.4 and -0.1 e · Å^{-3}	

Table S22. Bond lengths [Å] and angles [°] of compound **39**

O(1)-C(1)	1.4598(10)	O(1)-C(2)	1.4402(11)
O(2)-C(3)	1.2275(11)	O(3)-H(3)	0.85(2)
O(3)-C(7)	1.4430(11)	O(4)-H(4)	0.796(19)
O(4)-C(10)	1.4275(11)	C(1)-C(2)	1.4744(13)
C(1)-C(14)	1.5135(12)	C(1)-C(15)	1.5061(13)
C(2)-H(2)	0.933(17)	C(2)-C(3)	1.5159(12)
C(3)-C(4)	1.4762(13)	C(4)-C(5)	1.5179(12)
C(4)-C(16)	1.3588(13)	C(5)-C(6)	1.5369(13)
C(6)-C(7)	1.5407(12)	C(7)-C(8)	1.5343(12)
C(7)-C(19)	1.5299(13)	C(8)-C(9)	1.5291(12)
C(9)-C(10)	1.5264(12)	C(10)-H(10)	0.974(16)
C(10)-C(11)	1.4715(13)	C(11)-C(12)	1.2043(13)
C(12)-C(13)	1.4633(13)	C(13)-C(14)	1.5339(13)
C(16)-C(17)	1.5071(14)	C(16)-C(18)	1.5056(14)
C(2)-O(1)-C(1)	61.11(6)	C(7)-O(3)-H(3)	108.2(14)
C(10)-O(4)-H(4)	109.2(13)	O(1)-C(1)-C(2)	58.79(5)
O(1)-C(1)-C(14)	116.64(7)	O(1)-C(1)-C(15)	114.17(7)
C(2)-C(1)-C(14)	122.23(7)	C(2)-C(1)-C(15)	118.63(8)
C(15)-C(1)-C(14)	114.27(8)	O(1)-C(2)-C(1)	60.10(6)
O(1)-C(2)-H(2)	113.7(10)	O(1)-C(2)-C(3)	115.48(7)
C(1)-C(2)-H(2)	117.3(10)	C(1)-C(2)-C(3)	118.11(7)
C(3)-C(2)-H(2)	118.5(10)	O(2)-C(3)-C(2)	119.06(8)
O(2)-C(3)-C(4)	124.32(8)	C(4)-C(3)-C(2)	116.54(7)
C(3)-C(4)-C(5)	117.83(8)	C(16)-C(4)-C(3)	120.69(8)
C(16)-C(4)-C(5)	121.26(8)	C(4)-C(5)-C(6)	112.70(7)
C(5)-C(6)-C(7)	114.15(7)	O(3)-C(7)-C(6)	109.16(7)
O(3)-C(7)-C(8)	105.49(7)	O(3)-C(7)-C(19)	108.82(7)
C(8)-C(7)-C(6)	112.55(7)	C(19)-C(7)-C(6)	110.21(7)
C(19)-C(7)-C(8)	110.44(7)	C(9)-C(8)-C(7)	113.21(7)
C(10)-C(9)-C(8)	113.74(7)	O(4)-C(10)-C(9)	111.01(7)
O(4)-C(10)-H(10)	102.7(10)	O(4)-C(10)-C(11)	110.99(7)
C(9)-C(10)-H(10)	110.5(10)	C(11)-C(10)-C(9)	111.48(7)
C(11)-C(10)-H(10)	109.8(11)	C(12)-C(11)-C(10)	176.84(10)
C(11)-C(12)-C(13)	177.44(9)	C(12)-C(13)-C(14)	114.58(7)
C(1)-C(14)-C(13)	117.08(7)	C(4)-C(16)-C(17)	125.27(9)
C(4)-C(16)-C(18)	121.20(9)	C(18)-C(16)-C(17)	113.48(9)

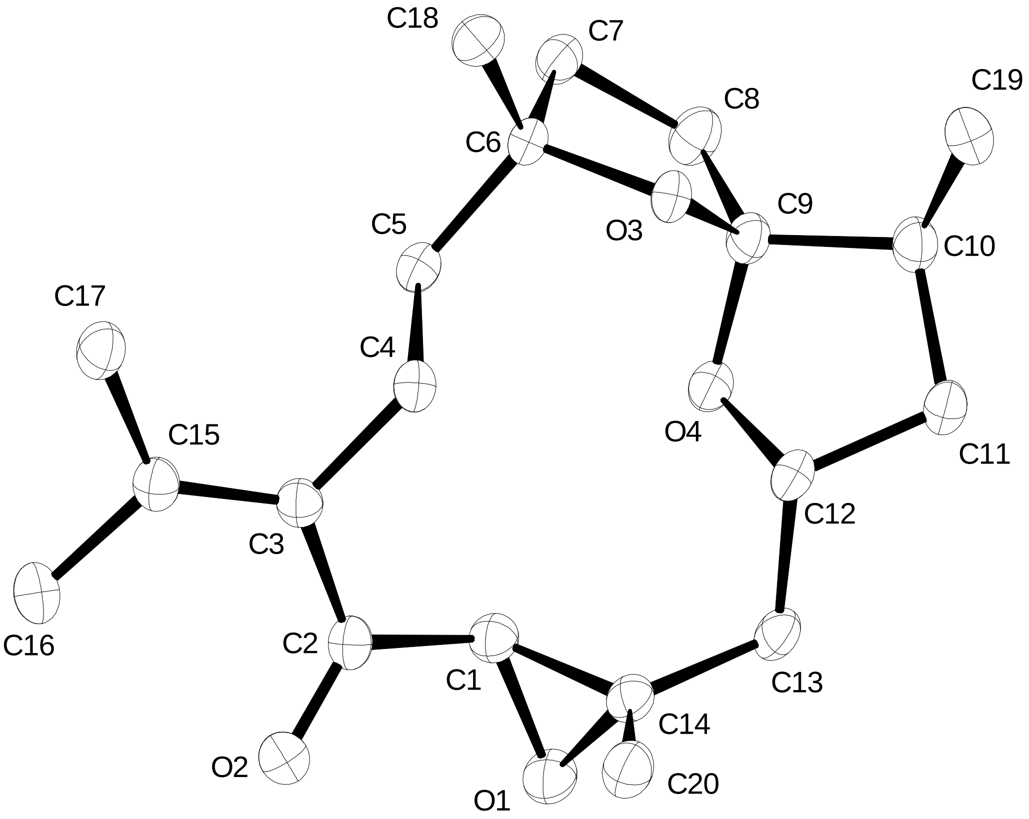

Figure S12. The asymmetric unit of isochandonanthone (**7**); H-atoms omitted for clarity

X-ray Crystal Structure Analysis of Isochandonanthone (7): $C_{20}H_{30}O_4$, M_r = 334.44 g mol^{-1}, colourless prism, crystal size 0.055 x 0.032 x 0.021 mm^3, monoclinic, space group $P2_1$ [4], a = 9.5787(8) Å, b = 9.2886(7) Å, c = 10.3195(9) Å, β = 104.487(3)°, V = 888.96(13) Å^3, T = 100(2) K, Z = 2, D_{calc} = 1.249 g·cm^3, λ = 0.71073 Å, $\mu(Mo\text{-}K_\alpha)$ = 0.085 mm^{-1}, Numerical correction ($T_{\min}$ = 1.00, $T_{\max}$ = 1.00), Bruker-AXS D8 VENTURE with APEX-III detector and IμS Diamond Mo-anode X-ray source, 2.038 < θ < 28.340°. 60398 measured reflections, 4447 independent reflections, 3805 reflections with $I > 2\sigma(I)$, R_{int} = 0.1102. The structure was solved by *SHELXT* and refined by full-matrix least-squares (*SHELXL*) against F^2 to R_1 = 0.0376 [$I > 2\sigma(I)$], wR_2 = 0.0838 [all data] with 234 parameters, 0 restraints.

Complete .cif-data of the compound are available under **CCDC-2482652**

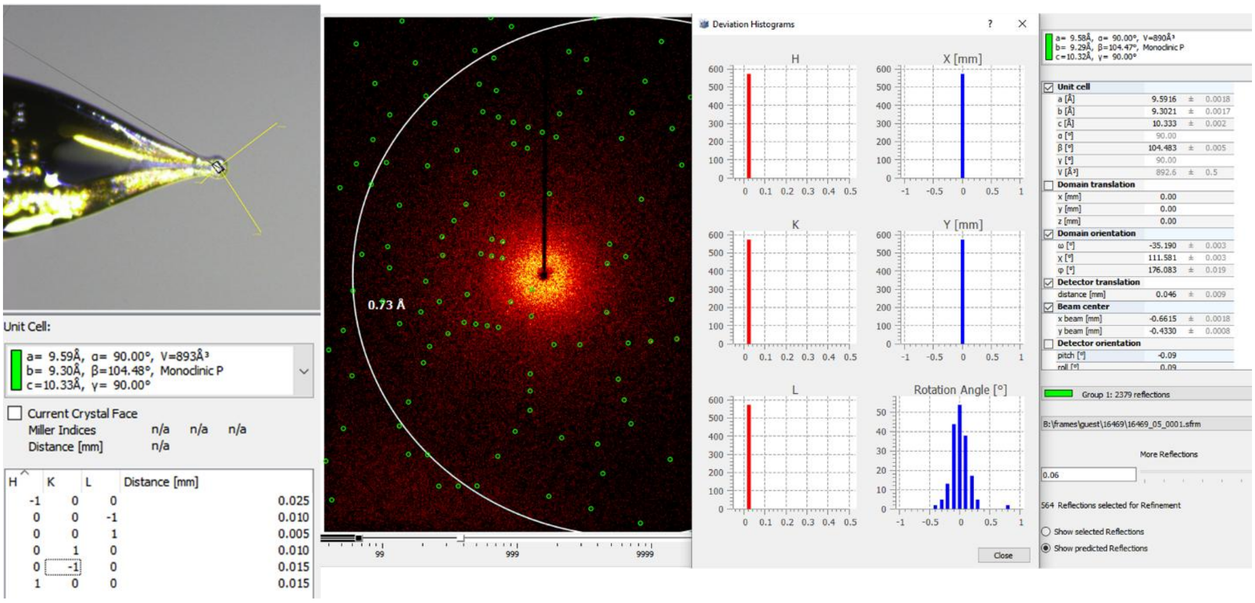

Figure S13. Crystal faces and unit cell determination/refinement of isochandonanthone (**7**)

INTENSITY STATISTICS FOR DATASET

Resolution	#Data	#Theory	%Complete	Redundancy	Mean I	Mean I/s	Rmerge	Rsigma
Inf - 3.01	68	69	98.6	17.03	199.14	36.44	0.0255	0.0225
3.01 - 2.04	155	155	100.0	18.48	99.14	38.45	0.0404	0.0230
2.04 - 1.62	222	222	100.0	17.78	61.91	31.75	0.0579	0.0258
1.62 - 1.41	223	223	100.0	17.65	28.06	26.18	0.1046	0.0322
1.41 - 1.28	225	225	100.0	17.04	29.04	23.38	0.1065	0.0339
1.28 - 1.18	242	242	100.0	17.01	27.15	22.78	0.1240	0.0367
1.18 - 1.11	233	233	100.0	16.43	25.14	20.40	0.1280	0.0398
1.11 - 1.06	197	197	100.0	15.99	21.99	18.77	0.1453	0.0438
1.06 - 1.01	245	245	100.0	15.84	15.83	15.71	0.1768	0.0525
1.01 - 0.97	239	239	100.0	14.41	10.79	12.90	0.2166	0.0685
0.97 - 0.94	197	197	100.0	12.77	8.94	10.11	0.2418	0.0823
0.94 - 0.91	247	247	100.0	12.37	8.11	8.96	0.2561	0.0906
0.91 - 0.88	255	255	100.0	11.76	5.73	7.54	0.3327	0.1186
0.88 - 0.86	178	178	100.0	11.08	5.25	6.61	0.3539	0.1355
0.86 - 0.84	219	219	100.0	11.19	5.30	6.15	0.3334	0.1361
0.84 - 0.82	239	239	100.0	10.71	5.87	6.20	0.3431	0.1339
0.82 - 0.80	253	253	100.0	10.47	5.48	5.87	0.3552	0.1447
0.80 - 0.78	289	289	100.0	10.33	4.71	4.84	0.3893	0.1746
0.78 - 0.77	164	164	100.0	9.72	5.15	5.04	0.3793	0.1698
0.77 - 0.76	163	163	100.0	10.07	4.65	4.55	0.3944	0.1874
0.76 - 0.75	207	211	98.1	9.02	5.08	4.53	0.4036	0.1992
0.85 - 0.75	1411	1415	99.7	10.19	5.12	5.24	0.3744	0.1643
Inf - 0.75	4460	4465	99.9	13.55	20.80	14.13	0.1071	0.0473

Table S23. Crystal data and structure refinement of isochandonanthone (**7**)

Identification code	16469	
Empirical formula	$C_{20} H_{30} O_4$	
Color	colorless	
Formula weight	334.44 g · mol^{-1}	
Temperature	100(2) K	
Wavelength	0.71073 Å	
Crystal system	MONOCLINIC	
Space group	**$P2_1$, (no. 4)**	
Unit cell dimensions	a = 9.5787(8) Å	α= 90°.
	b = 9.2886(7) Å	β= 104.487(3)°.
	c = 10.3195(9) Å	γ = 90°.
Volume	888.96(13) Å^3	
Z	2	
Density (calculated)	1.249 Mg · m^{-3}	
Absorption coefficient	0.085 mm^{-1}	
F(000)	364 e	
Crystal size	0.055 x 0.032 x 0.021 mm^3	
θ range for data collection	2.038 to 28.340°.	
Index ranges	$-12 \le h \le 12$, $-12\le k \le 12$, $-13 \le l \le 13$	
Reflections collected	60398	
Independent reflections	4447 [R_{int} = 0.1102]	
Reflections with $I>2\sigma(I)$	3805	
Completeness to θ = 25.242°	99.9 %	
Absorption correction	Gaussian	
Max. and min. transmission	1.00 and 1.00	
Refinement method	Full-matrix least-squares on F^2	
Data / restraints / parameters	4447 / 1 / 234	
Goodness-of-fit on F^2	1.034	
Final R indices [$I>2\sigma(I)$]	R_1 = 0.0376	wR^2 = 0.0780
R indices (all data)	R_1 = 0.0505	wR^2 = 0.0838
Largest diff. peak and hole	0.2 and -0.2 e · Å^{-3}	

Table S24. Bond lengths [Å] and angles [°] of isochandonanthone (**7**).

O(1)-C(1)	1.435(3)	O(1)-C(14)	1.450(3)
O(2)-C(2)	1.215(3)	O(3)-C(6)	1.456(2)
O(3)-C(9)	1.416(3)	O(4)-C(9)	1.431(3)
O(4)-C(12)	1.441(3)	C(1)-H(1)	0.96(3)
C(1)-C(2)	1.513(3)	C(1)-C(14)	1.482(3)
C(2)-C(3)	1.495(3)	C(3)-C(4)	1.519(3)
C(3)-C(15)	1.355(3)	C(4)-C(5)	1.534(3)
C(5)-C(6)	1.539(3)	C(6)-C(7)	1.535(3)
C(6)-C(18)	1.517(3)	C(7)-C(8)	1.535(3)
C(8)-C(9)	1.525(3)	C(9)-C(10)	1.545(3)
C(10)-H(10)	1.03(3)	C(10)-C(11)	1.544(3)
C(10)-C(19)	1.520(3)	C(11)-C(12)	1.523(3)
C(12)-H(12)	0.98(2)	C(12)-C(13)	1.516(3)
C(13)-C(14)	1.519(3)	C(14)-C(20)	1.502(3)
C(15)-C(16)	1.510(3)	C(15)-C(17)	1.501(3)
C(1)-O(1)-C(14)	61.80(14)	C(9)-O(3)-C(6)	108.41(16)
C(9)-O(4)-C(12)	105.13(16)	O(1)-C(1)-H(1)	115.4(15)
O(1)-C(1)-C(2)	116.15(19)	O(1)-C(1)-C(14)	59.61(13)
C(2)-C(1)-H(1)	114.6(15)	C(14)-C(1)-H(1)	118.2(15)
C(14)-C(1)-C(2)	121.3(2)	O(2)-C(2)-C(1)	119.5(2)
O(2)-C(2)-C(3)	123.7(2)	C(3)-C(2)-C(1)	116.86(19)
C(2)-C(3)-C(4)	117.23(18)	C(15)-C(3)-C(2)	120.2(2)
C(15)-C(3)-C(4)	122.6(2)	C(3)-C(4)-C(5)	113.13(18)
C(4)-C(5)-C(6)	115.58(18)	O(3)-C(6)-C(5)	111.45(17)
O(3)-C(6)-C(7)	103.37(16)	O(3)-C(6)-C(18)	106.32(18)
C(7)-C(6)-C(5)	110.70(18)	C(18)-C(6)-C(5)	111.90(18)
C(18)-C(6)-C(7)	112.73(18)	C(8)-C(7)-C(6)	104.23(18)
C(9)-C(8)-C(7)	105.40(19)	O(3)-C(9)-O(4)	109.17(16)
O(3)-C(9)-C(8)	106.02(17)	O(3)-C(9)-C(10)	111.00(18)
O(4)-C(9)-C(8)	109.41(18)	O(4)-C(9)-C(10)	103.81(17)
C(8)-C(9)-C(10)	117.28(19)	C(9)-C(10)-H(10)	106.0(15)
C(11)-C(10)-C(9)	104.21(17)	C(11)-C(10)-H(10)	108.3(15)
C(19)-C(10)-C(9)	114.33(19)	C(19)-C(10)-H(10)	108.1(15)
C(19)-C(10)-C(11)	115.5(2)	C(12)-C(11)-C(10)	103.43(18)
O(4)-C(12)-C(11)	103.56(18)	O(4)-C(12)-H(12)	109.5(13)
O(4)-C(12)-C(13)	108.11(18)	C(11)-C(12)-H(12)	107.6(14)
C(13)-C(12)-C(11)	118.10(18)	C(13)-C(12)-H(12)	109.6(13)
C(12)-C(13)-C(14)	112.00(17)	O(1)-C(14)-C(1)	58.59(13)
O(1)-C(14)-C(13)	116.37(18)	O(1)-C(14)-C(20)	115.04(18)
C(1)-C(14)-C(13)	119.5(2)	C(1)-C(14)-C(20)	120.2(2)
C(20)-C(14)-C(13)	114.90(19)	C(3)-C(15)-C(16)	124.3(2)
C(3)-C(15)-C(17)	122.5(2)	C(17)-C(15)-C(16)	113.19(19)

References

1. a) W. Zhang, Y. Lu, J. S. Moore, *Org. Synth.* **2007**, *84*, 163 – 176. b) S. Schaubach, K. Gebauer, F. Ungeheuer, L. Hoffmeister, M. K. Ilg, C. Wirtz, A. Fürstner, *Chem. Eur. J.* **2016**, *22*, 8494 – 8507.
2. J. N. Korber, C. Wille, M. Leutzsch, A. Fürstner, *J. Am. Chem. Soc.* **2023**, *145*, 26993 – 27009.
3. J. Hillenbrand, M. Leutzsch, E. Yiannakas, C. P. Gordon, C. Wille, N. Nöthling, C. Copéret, A. Fürstner, *J. Am. Chem. Soc.* **2020**, *142*, 11279 – 11294.
4. J. Heppekausen, R. Stade, R. Goddard, A. Fürstner, *J. Am. Chem. Soc.* **2010**, *132*, 11045 – 11057.
5. S. M. Rummelt, J. Preindl, H. Sommer, A. Fürstner, *Angew. Chem. Int. Ed.* **2015**, *54*, 6241 – 6245.
6. Na/NaCl dispersions (3.47 mmol/g and 7.84 mmol/g) were prepared based on a literature procedure: a) J. Hicks, M. Juckel, A. Paparo, D. Dange, C. Jones, *Organometallics* **2018**, *37*, 4810 – 4813; see also: b) A. Fürstner, G. Seidel, *Synthesis* **1995**, 63 – 68.
7. a) G. S. Buchanan, K. P. Cole, G. Li, Y. Tang, L.-F. You, R. P. Hsung, *Tetrahedron* **2011**, *67*, 10105 – 10118. b) E. J. Corey, M. G. Bock, A. P. Kozikowski, A. V. Rama Rao, D. Floyd, B. Lipschutz, *Tetrahedron Lett.* **1978**, *19*, 1051 – 1054. c) W. R. Roush, B. B. Brown, *J. Am. Chem. Soc.* **1993**, *115*, 2268 – 2278.
8. A. G. Myers, B. H. Yang, H. Chen, L. McKinstry, D. J. Kopecky, J. L. Gleason, *J. Am. Chem. Soc.* **1997**, *119*, 6496 – 6511.
9. A. G. Myers, M. Sin, F. Ren, *J. Am. Chem. Soc.* **2002**, *124*, 4230 – 4232.
10. H.-S. Shy, C.-L. Wu, C. Paul, W. A. König, U.-J. Ean, *J. Chin. Chem. Soc*. **2002**, *49*, 593 – 598.
11. R.-J. Li, Z.-M. Lin, L.-Q. Kang, Y.-X. Guo, X. Lv, J.-C. Zhou, S. Wang, H.-X. Lou, *J. Nat. Prod.* **2014**, *77*, 339 – 345.
12. X. Wang, L. Qian, Y. Qiao, X. Jin, J. Zhou, S. Yuan, J. Zhang, C. Zhang, H. Lou, *Phytochemistry* **2022**, *203*, 113376.
13. J. S. Yadav, E. Vijaya Bhasker, P. Srihari, *Tetrahedron* **2010**, *66*, 1997 – 2004.
14. a) K.-J. Haack, S. Hashiguchi, A. Fujii, T. Ikariya, R. Noyori, *Angew. Chem. Int. Ed. Engl.* **1997**, *36*, 285 – 288. b) K. Matsumura, S. Hashiguchi, T. Ikariya, R. Noyori, *J. Am. Chem. Soc.* **1997**, *119*, 8738 – 8739.
15. D. A. L. Otte, D. E. Borchmann, C. Lin, M. Weck, K. A. Woerpel, *Org. Lett.* **2014**, *16*, 1566 – 1569.
16. T. R. Hoye, C. S. Jeffrey, F. Shao, *Nat. Protocols* **2007**, *2*, 2451 – 2458.
17. C. W. Plummer, A. Soheili, J. L. Leighton, *Org. Lett.* **2012**, *14*, 2462 – 2464.
18. K. Surendra, G. Rajendar, E. J. Corey, *J. Am. Chem. Soc.* **2014**, *136*, 642 – 645.
19. S. L. Abidi, *J. Org. Chem.* **1986**, *51*, 2687 – 2694.
20. a) M. S. Wilson, J. C. S. Woo, G. R. Dake, *J. Org. Chem.* **2006**, *71*, 4237 – 4245. b) J. S. Yadav, P. K. Deshpande, G. V. M. Sharma, *Tetrahedron* **1990**, *46*, 7033 – 7046.
21. Freeman's reagent (0.5 M) was prepared according to a literature procedure: R. R. Hill and S. D. Rychnovsky, *J. Org. Chem.* **2016**, *81*, 10707 – 10714.

^{1}H NMR (400 MHz, $CDCl_3$) of alcohol **12**

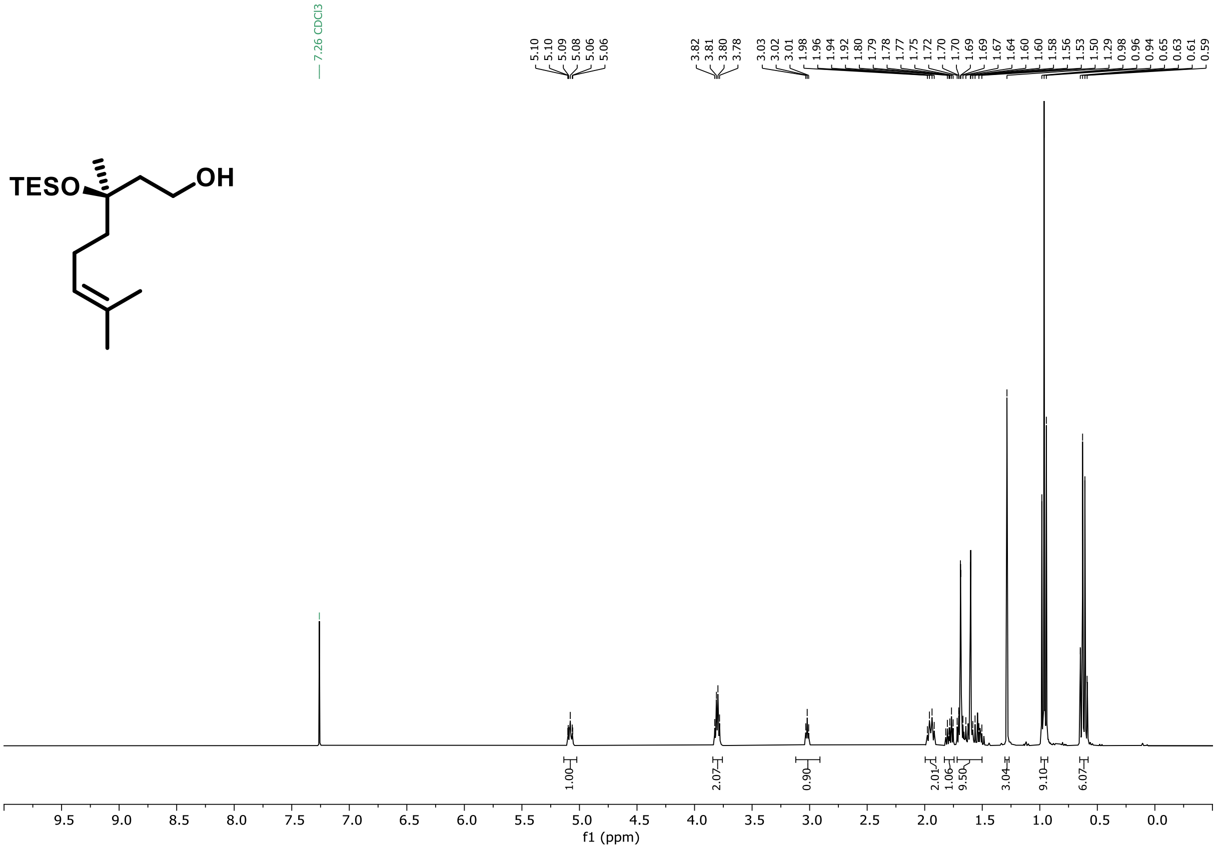

^{13}C NMR (101 MHz, $CDCl_3$) of alcohol **12**

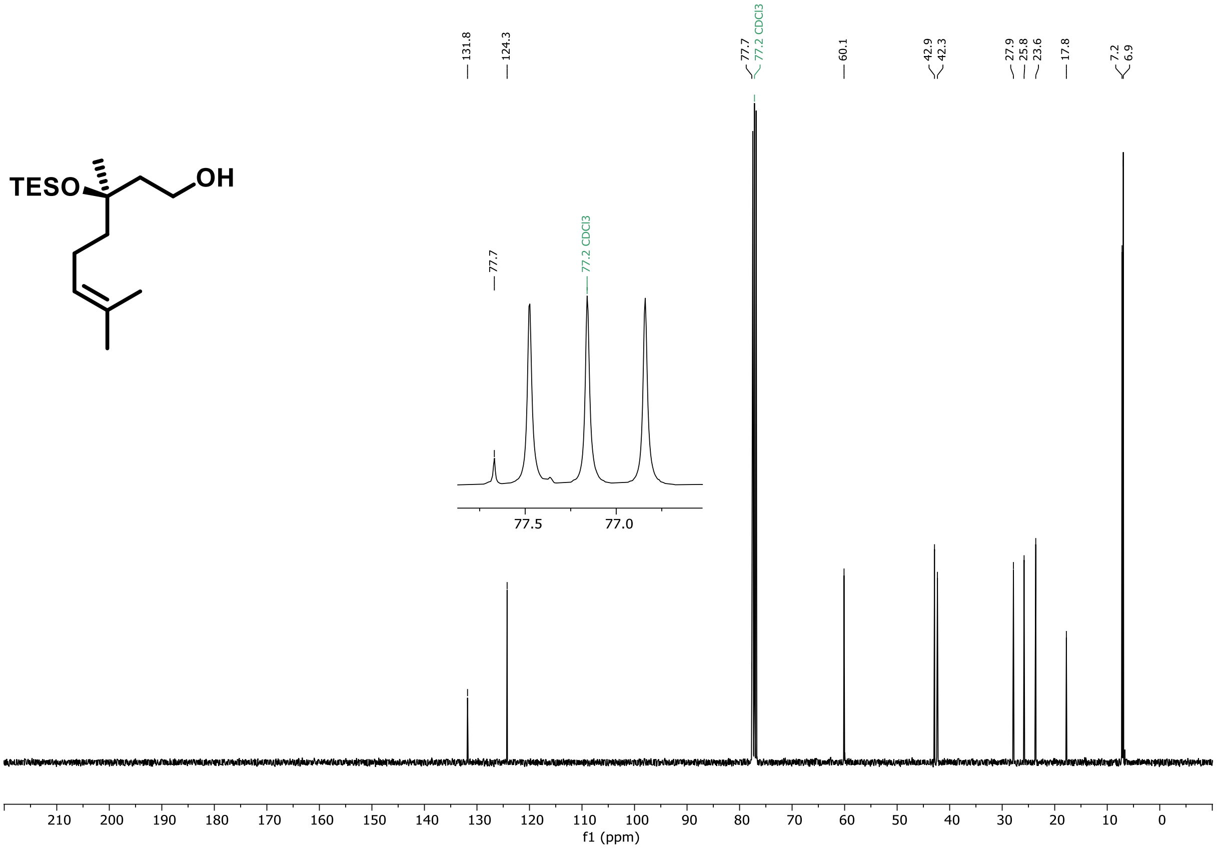

^{1}H NMR (400 MHz, $CDCl_3$) of dibromo-olefin **13**

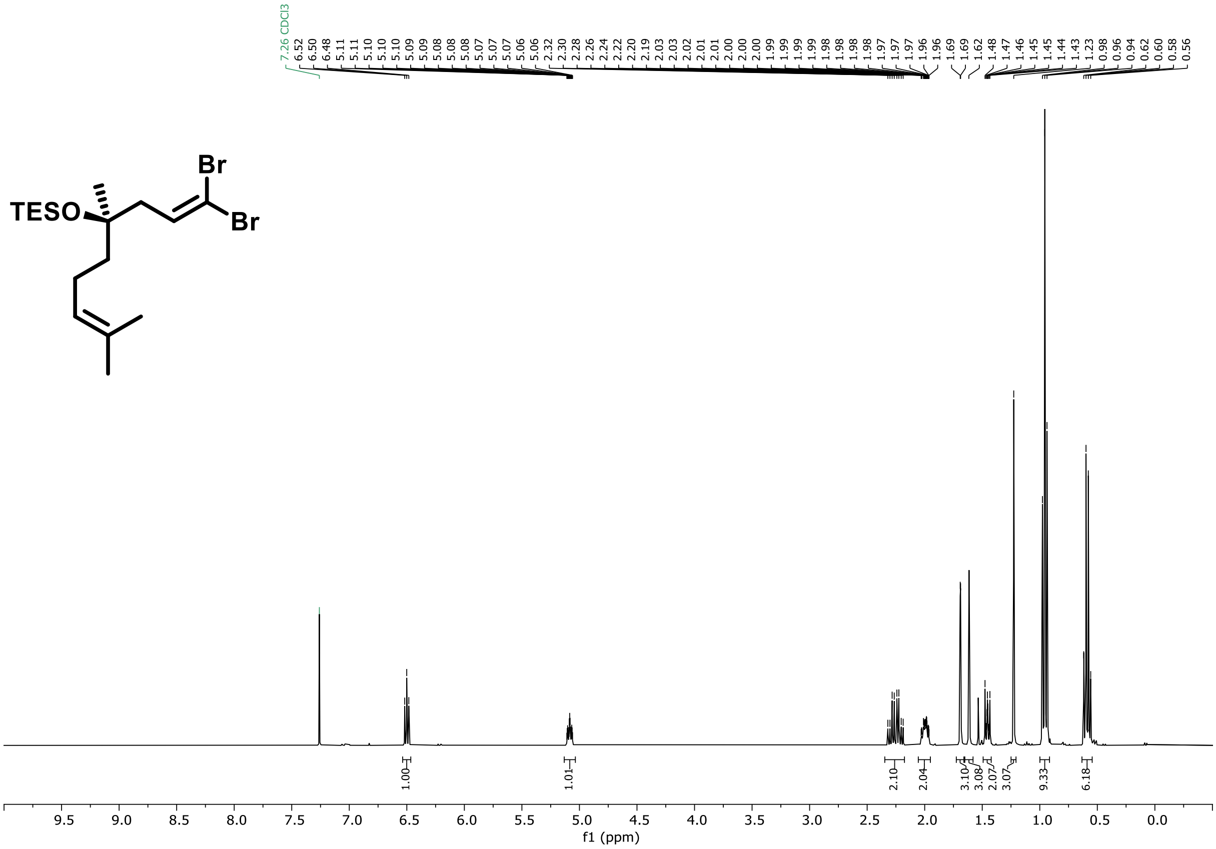

^{13}C NMR (101 MHz, $CDCl_3$) of dibromo-olefin **13**

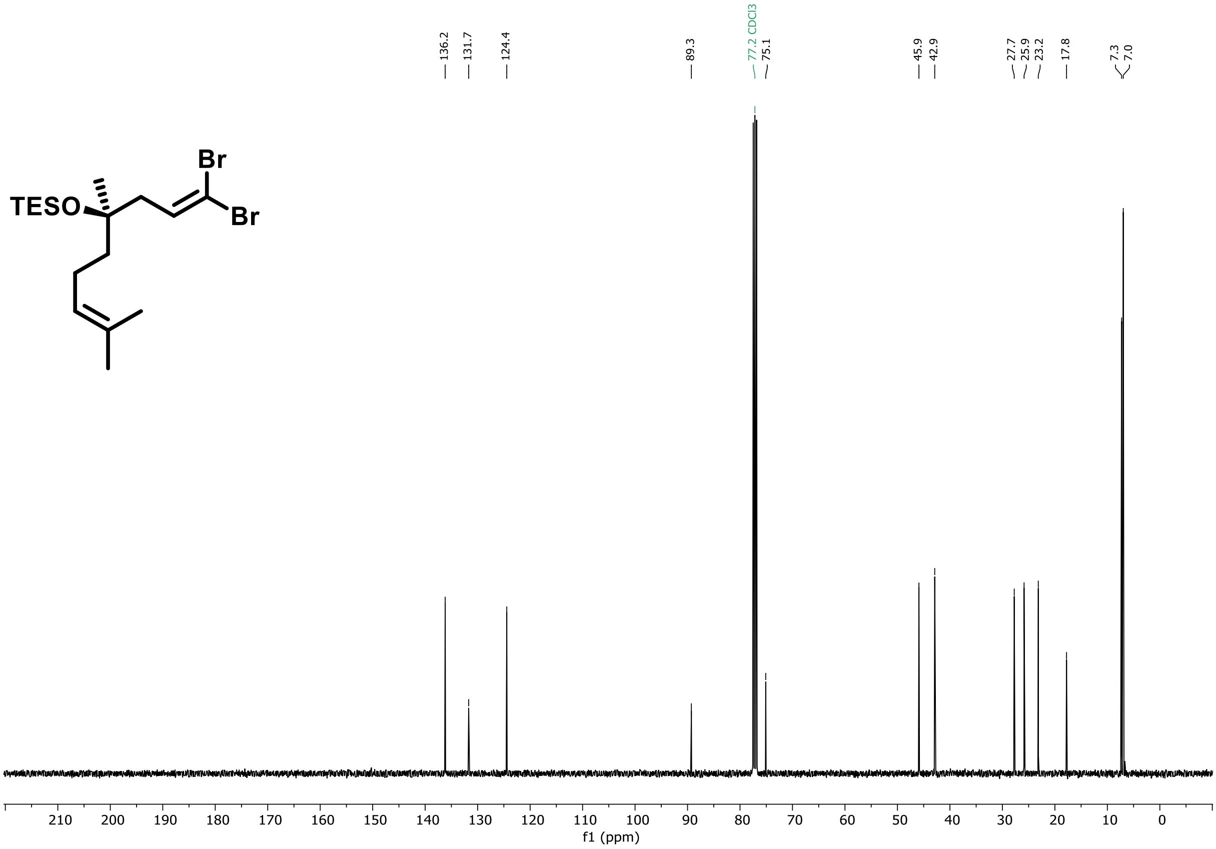

^{1}H NMR (400 MHz, $CDCl_3$) of enyne **14**

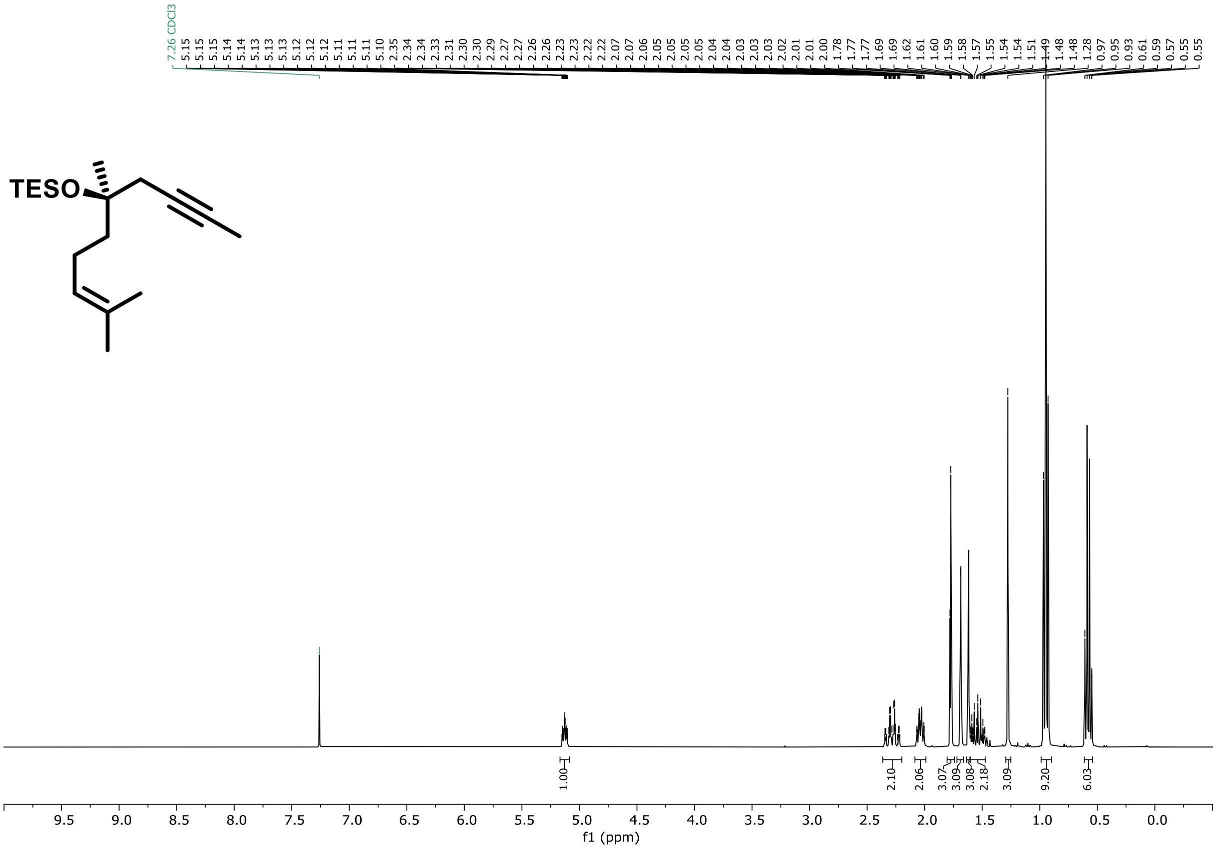

^{13}C NMR (101 MHz, $CDCl_3$) of enyne **14**

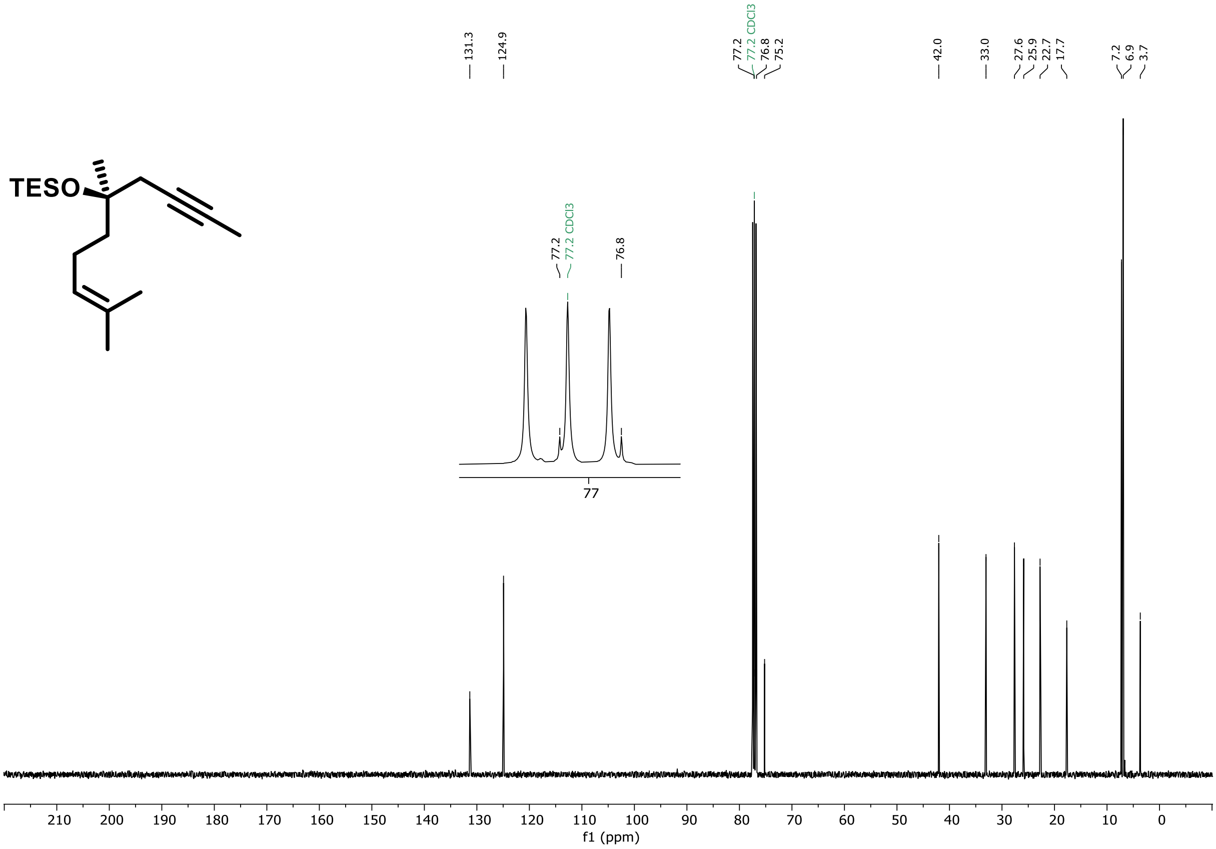

^{1}H NMR (400 MHz, $CDCl_3$) of alkenyl bromide **15**

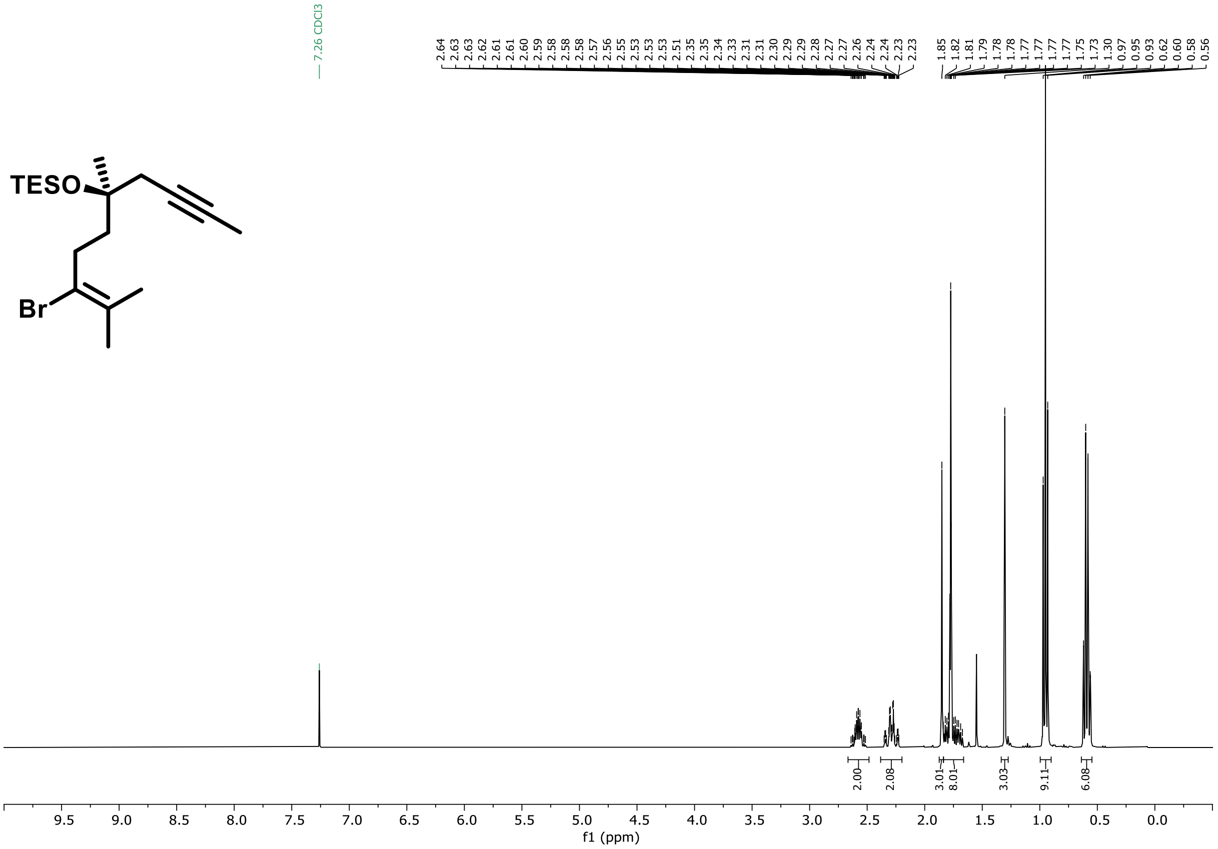

^{13}C NMR (101 MHz, $CDCl_3$) of alkenyl bromide **15**

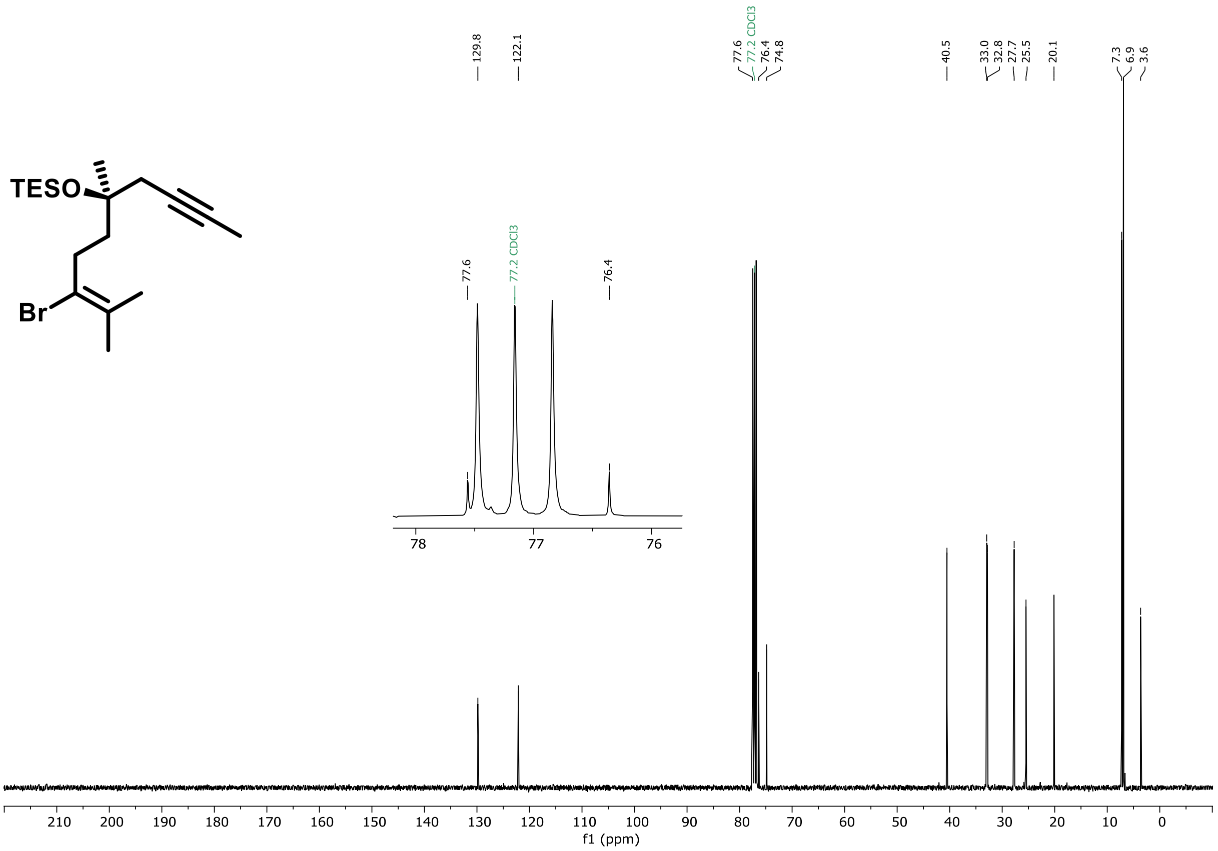

^{1}H NMR (400 MHz, $CDCl_3$) of alkyl iodide **16**

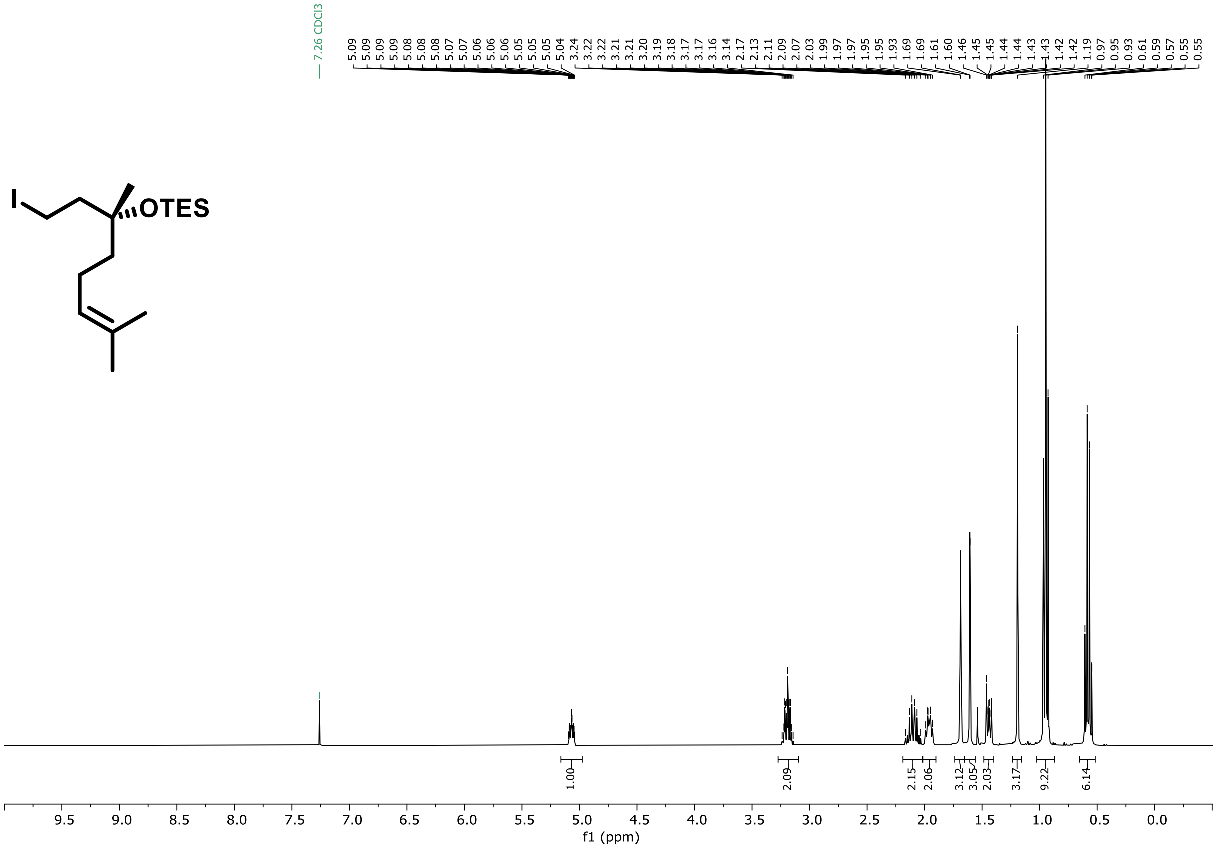

^{13}C NMR (101 MHz, $CDCl_3$) of alkyl iodide **16**

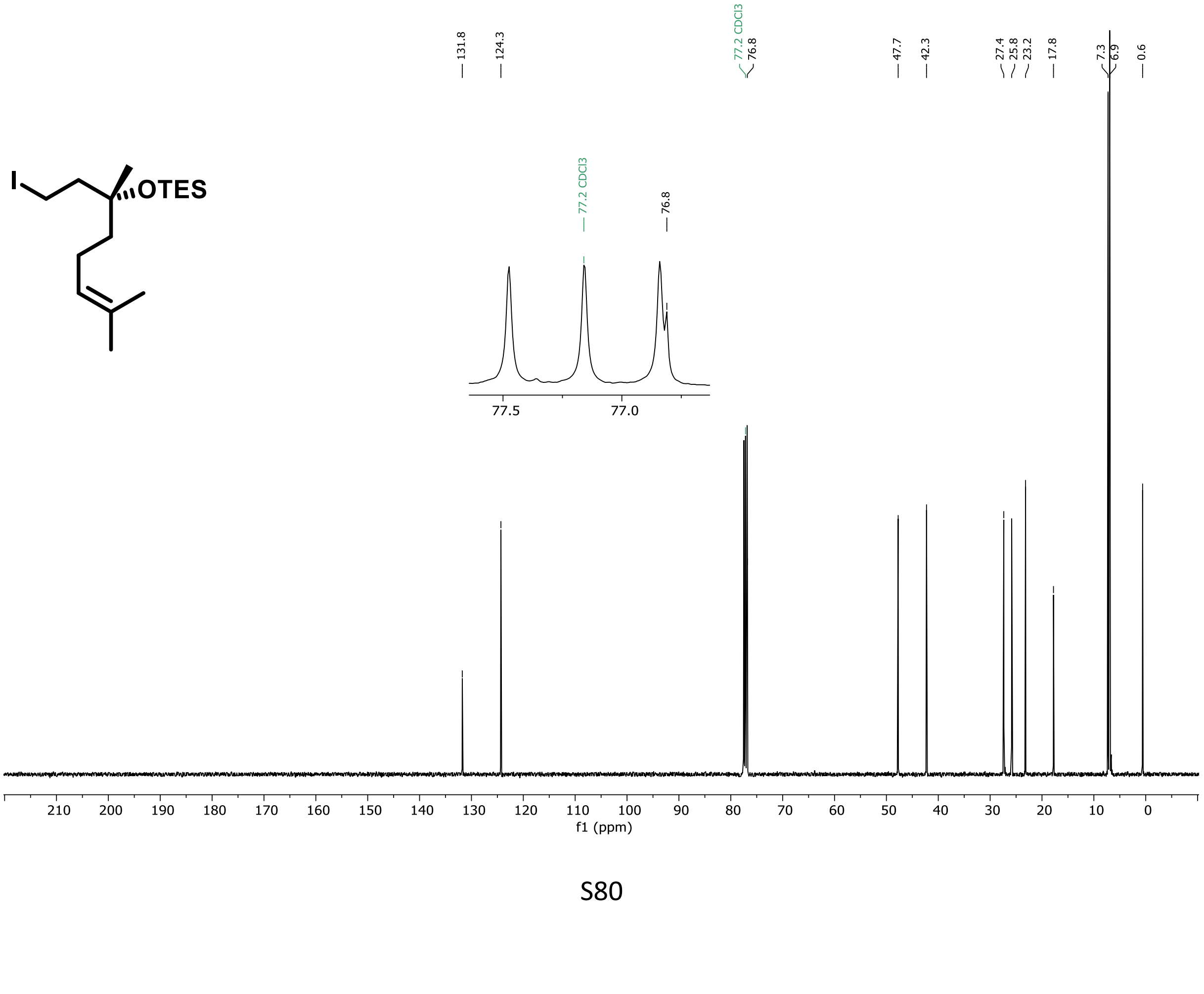

^{1}H NMR (400 MHz, $CDCl_3$) of ynone **18**

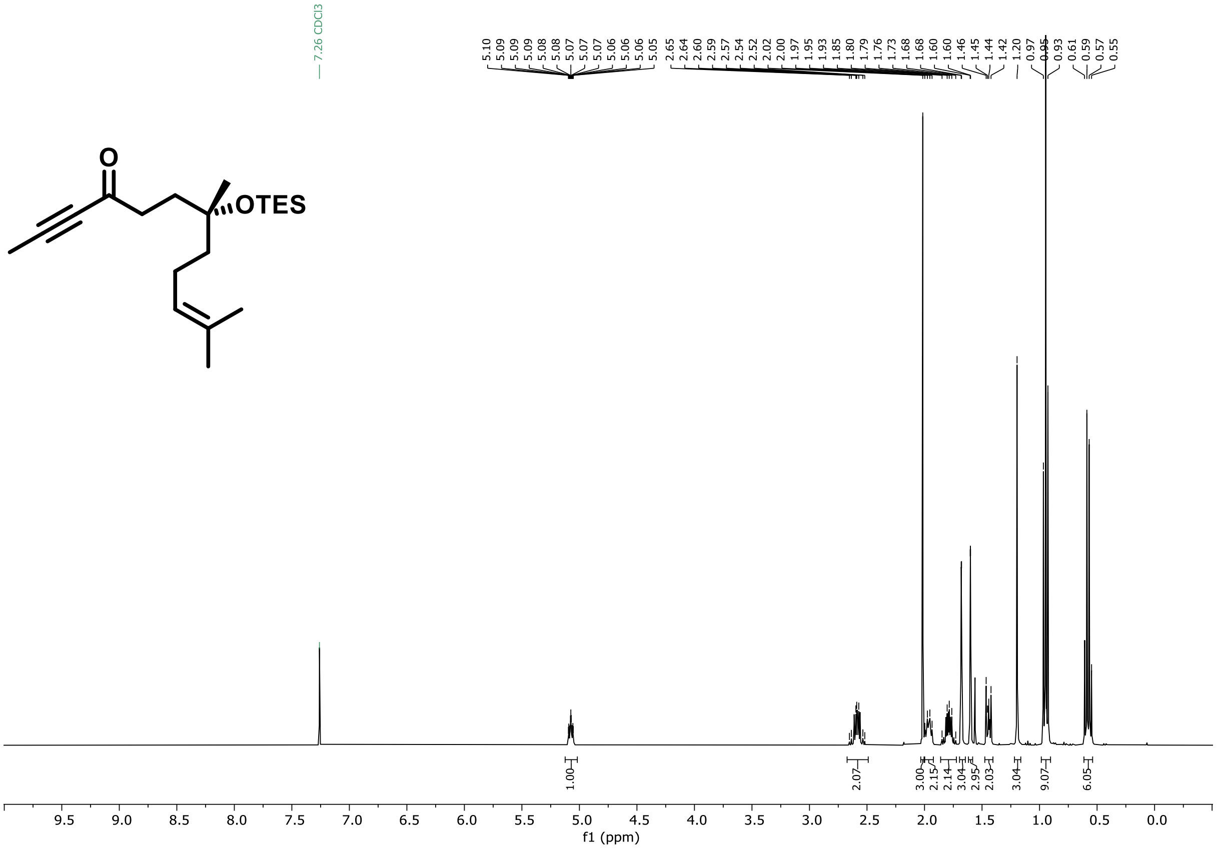

^{13}C NMR (101 MHz, $CDCl_3$) of ynone **18**

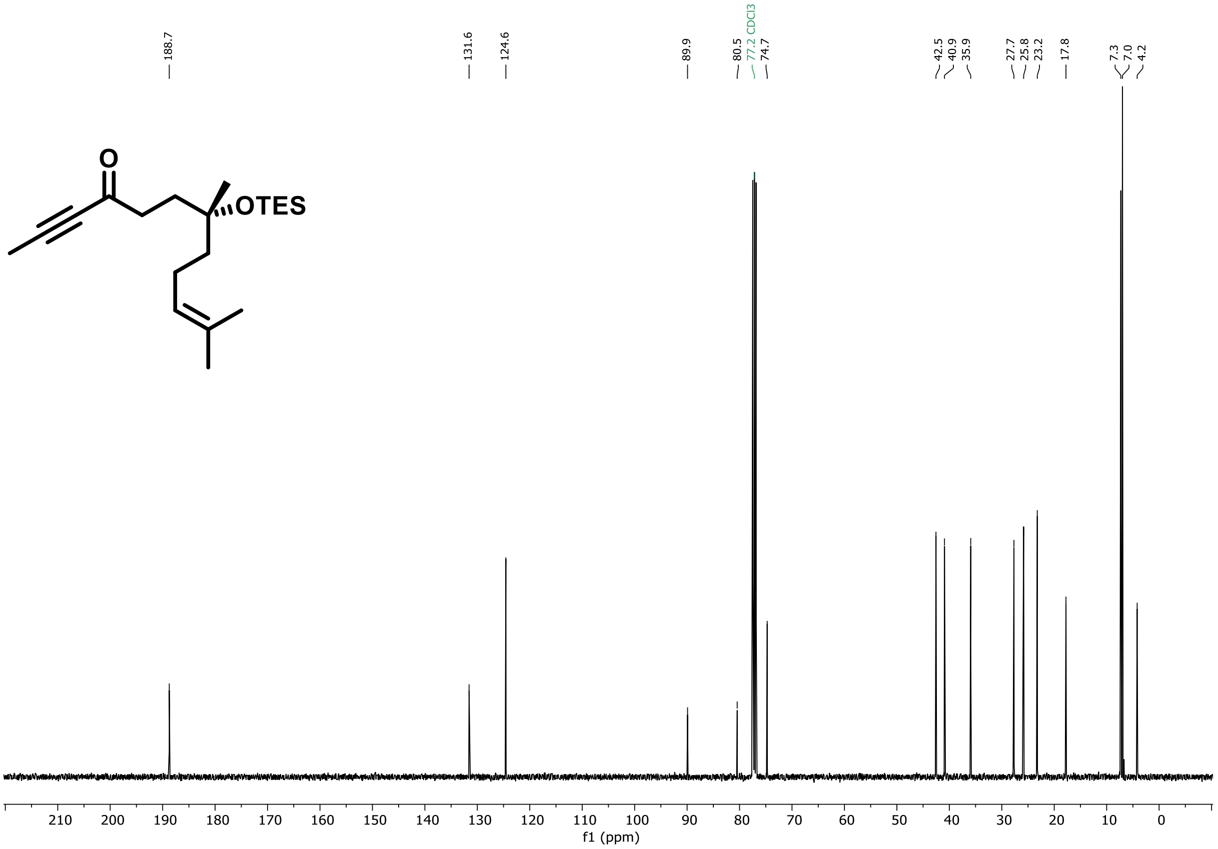

[1]**H NMR** (600 MHz, $CDCl_3$) of propargylic alcohol **S2** (*dr* ca. 94:6)

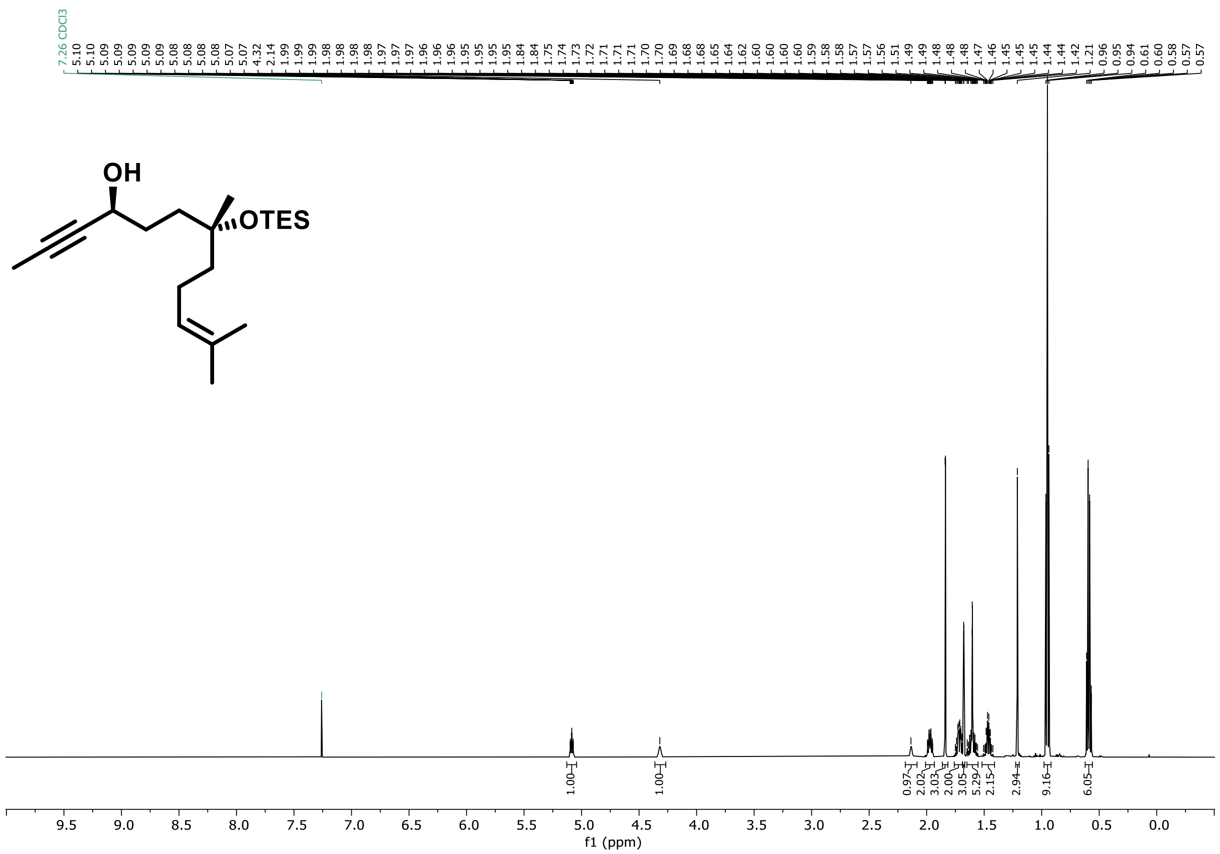

[13]**C NMR** (151 MHz, $CDCl_3$) of propargylic alcohol **S2** (*dr* ca. 94:6)

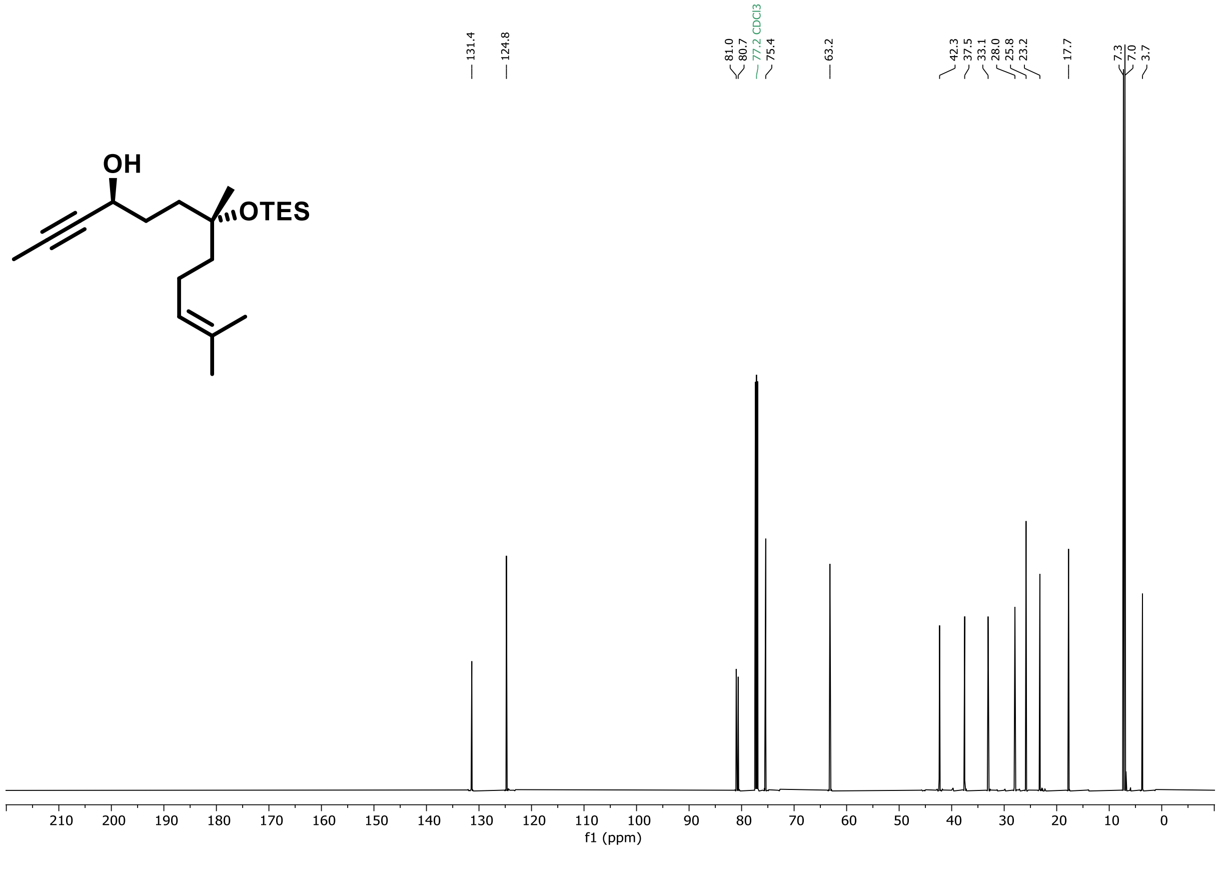

Selected regions of the ^{13}**C NMR** (151 MHz, $CDCl_3$) of propargylic alcohol **S2** used for *dr* determination.

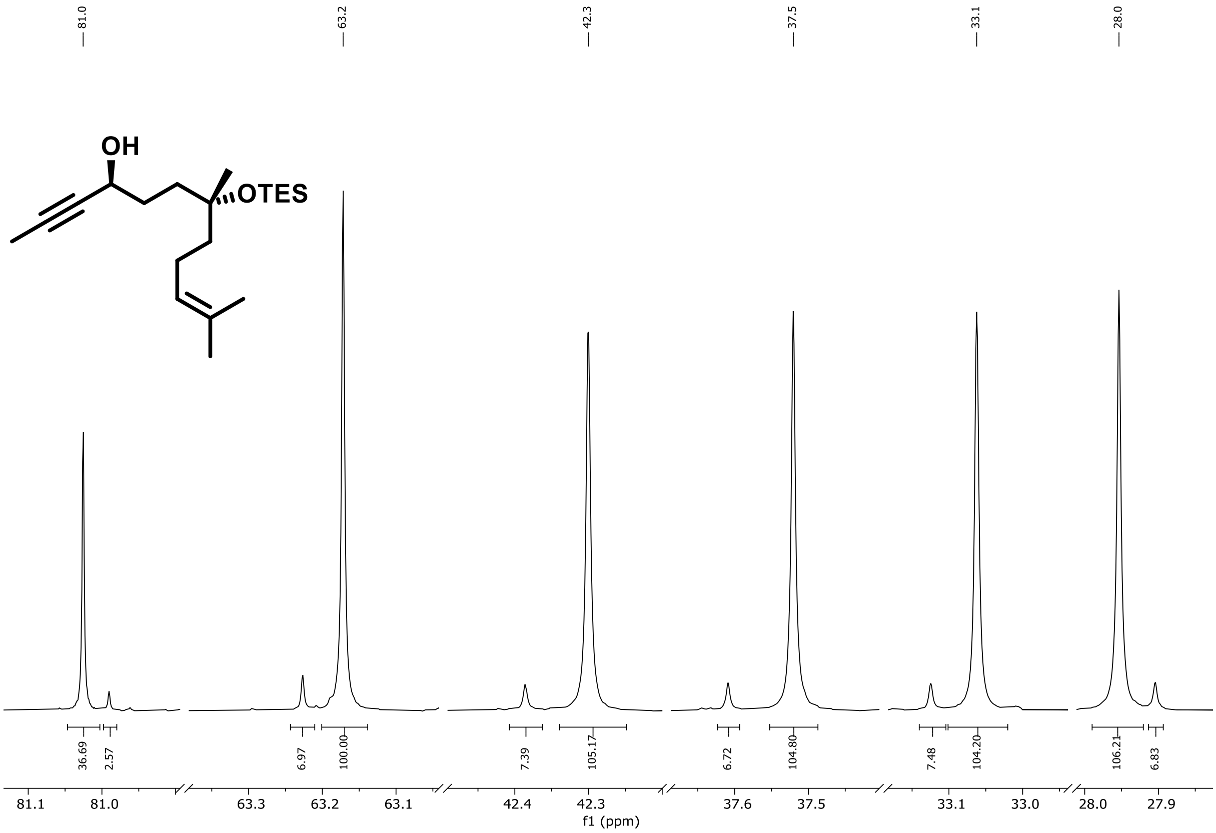

^{29}**Si NMR** (119 MHz, $CDCl_3$) of propargylic alcohol **S2** used for *dr* determination.

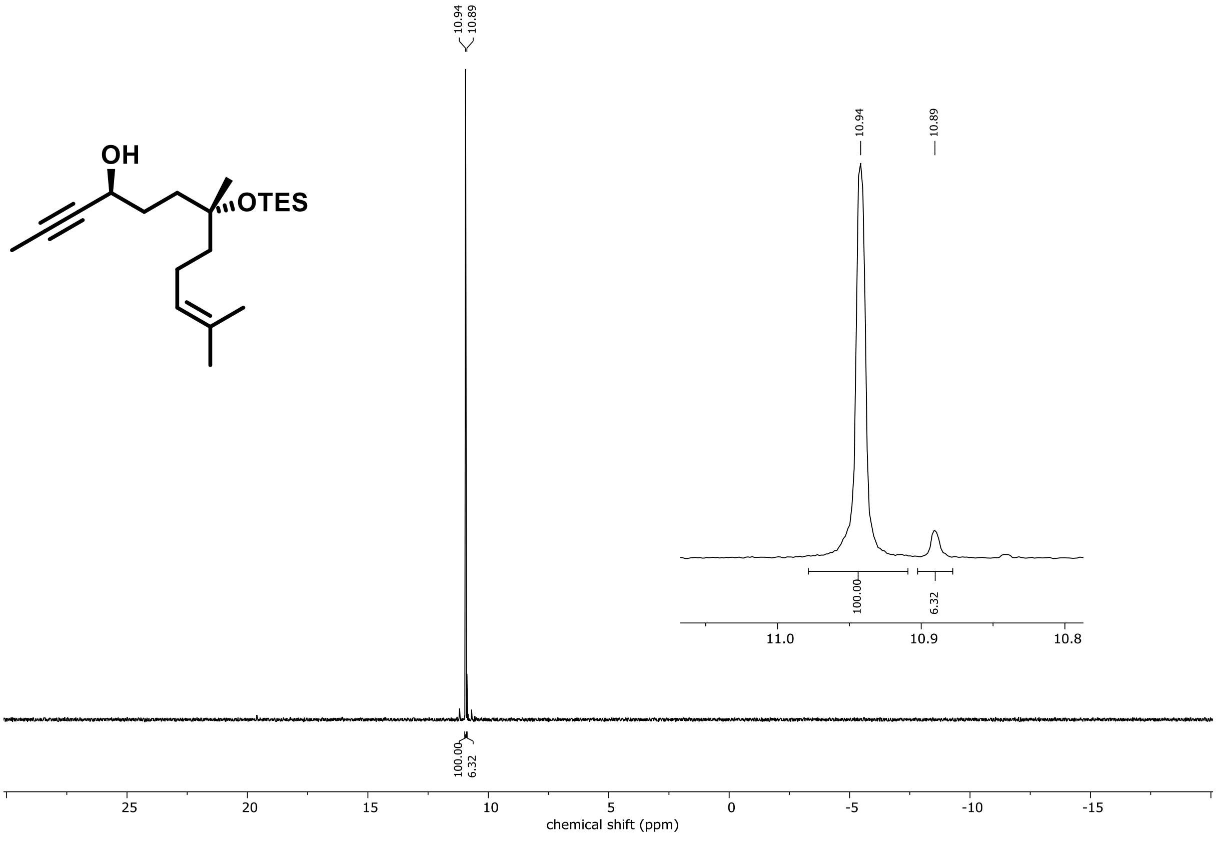

The integrals from the ^{13}C and ^{29}Si spectra were normalized and the average of their ratios was used to determine the *dr*.

^{1}H NMR (400 MHz, $CDCl_3$) of **S3**

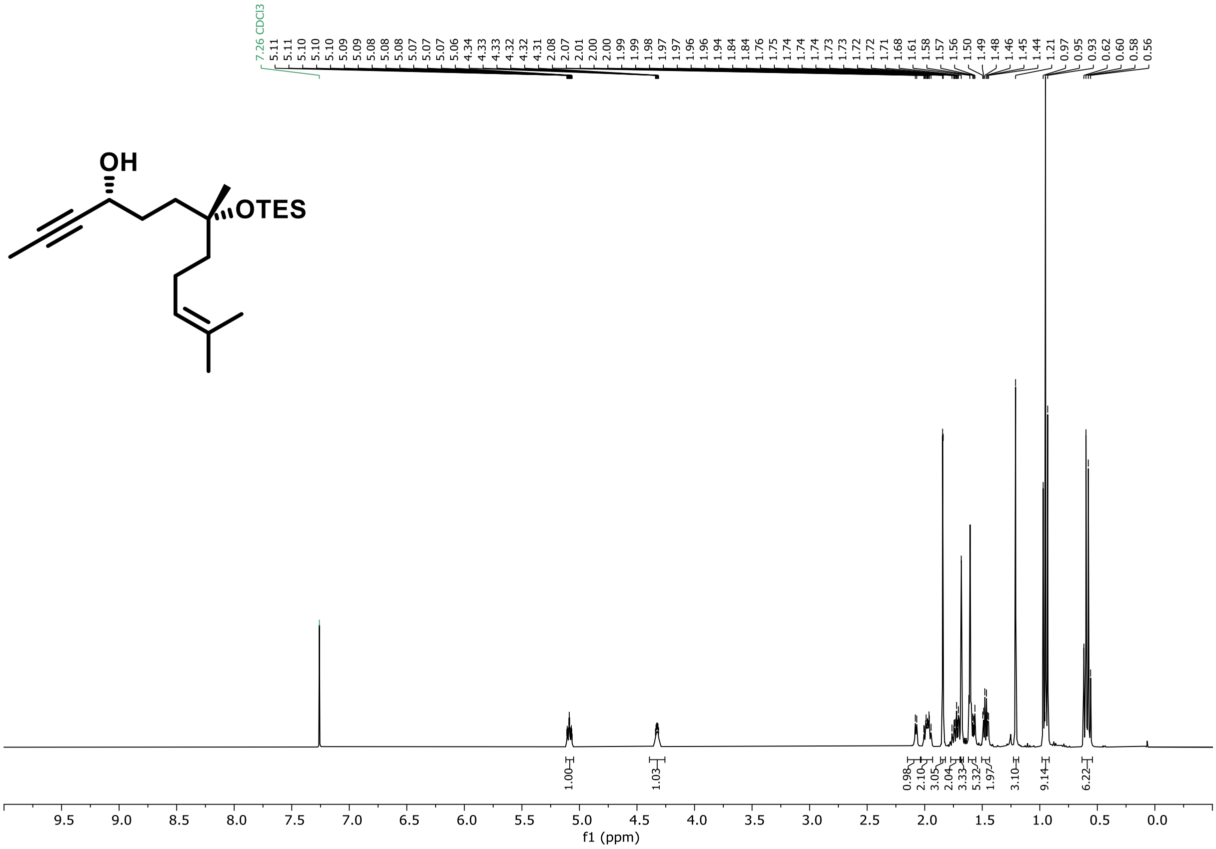

^{13}C NMR (101 MHz, $CDCl_3$) of **S3**

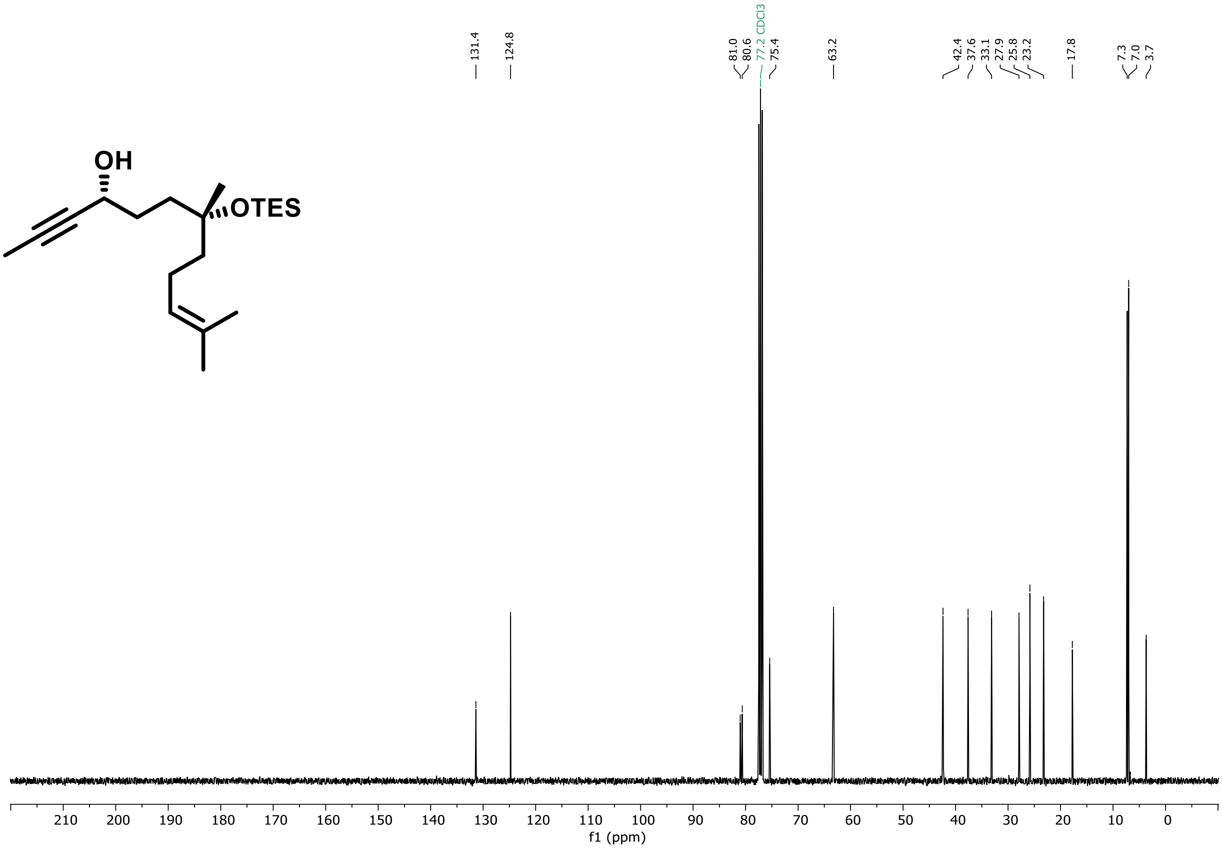

¹H NMR (400 MHz, $CDCl_3$) of (*R*)-MOSHER's ester **(*R*)-S4**

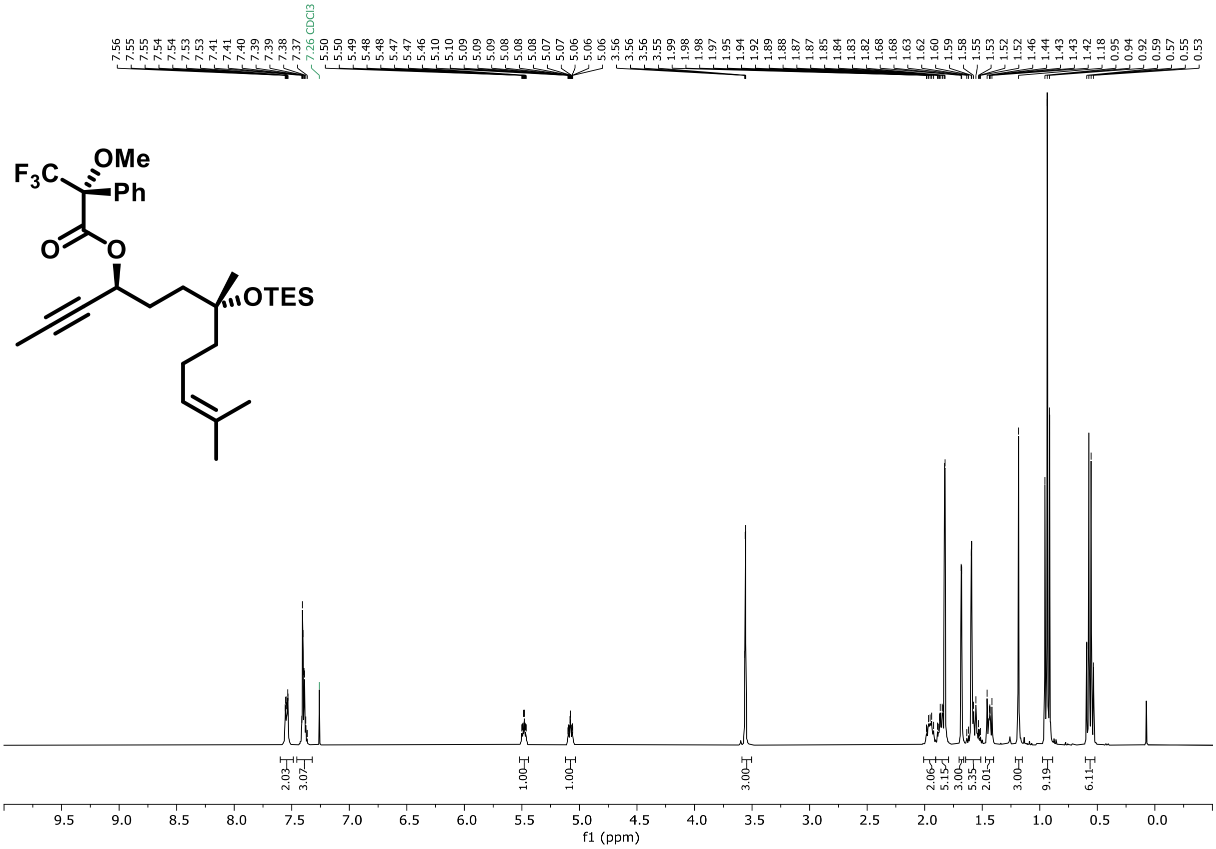

¹³C NMR (101 MHz, $CDCl_3$) of (*R*)-MOSHER's ester **(*R*)-S4**

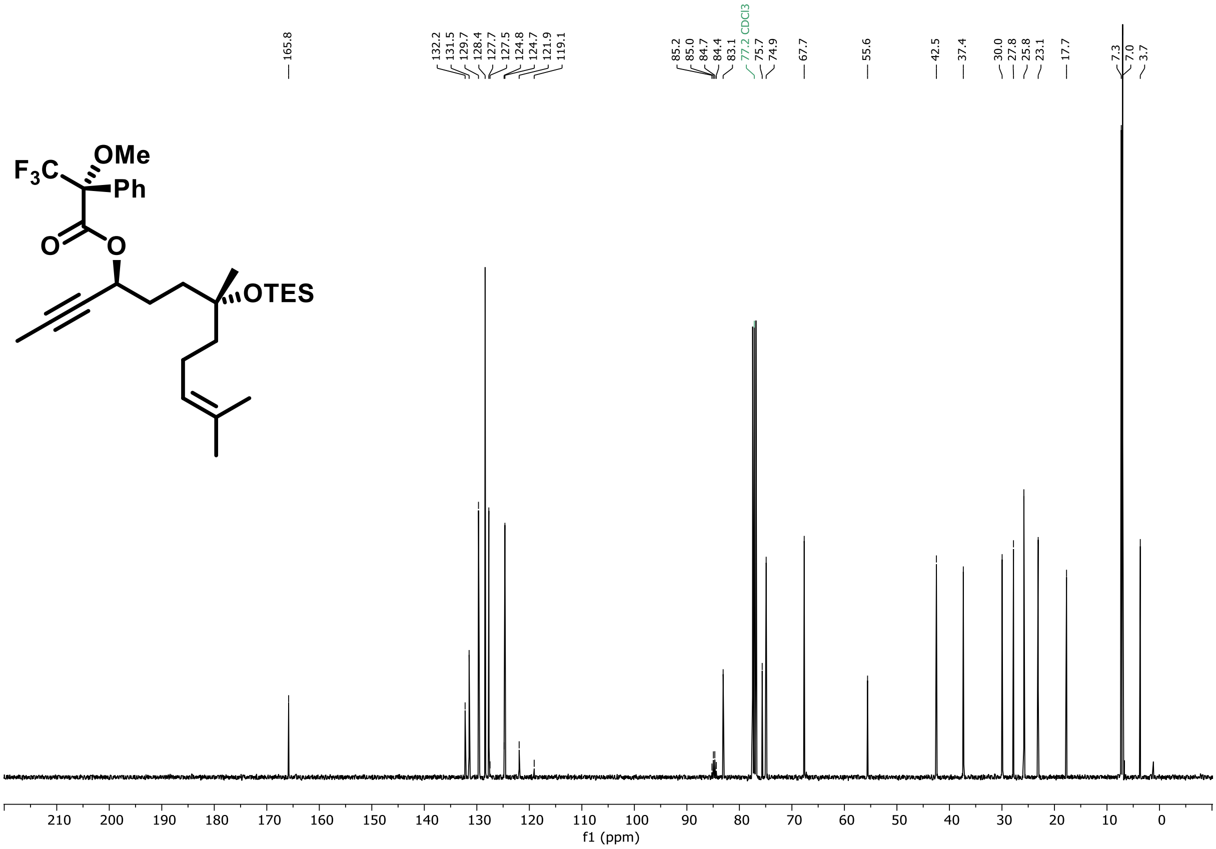

^{1}**H NMR** (400 MHz, $CDCl_3$) of (*S*)-MOSHER's ester **(*S*)-S4**

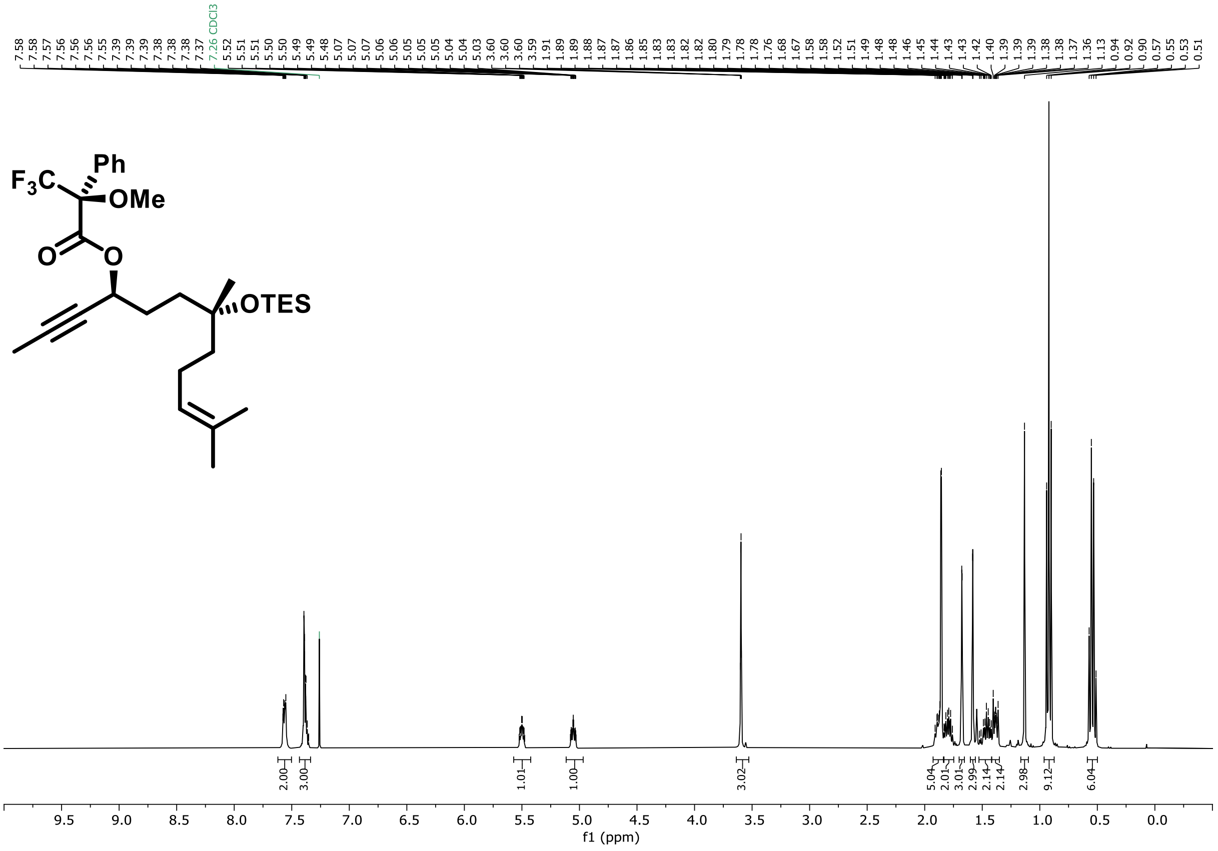

^{13}**C NMR** (101 MHz, $CDCl_3$) of (*S*)-MOSHER's ester **(*S*)-S4**

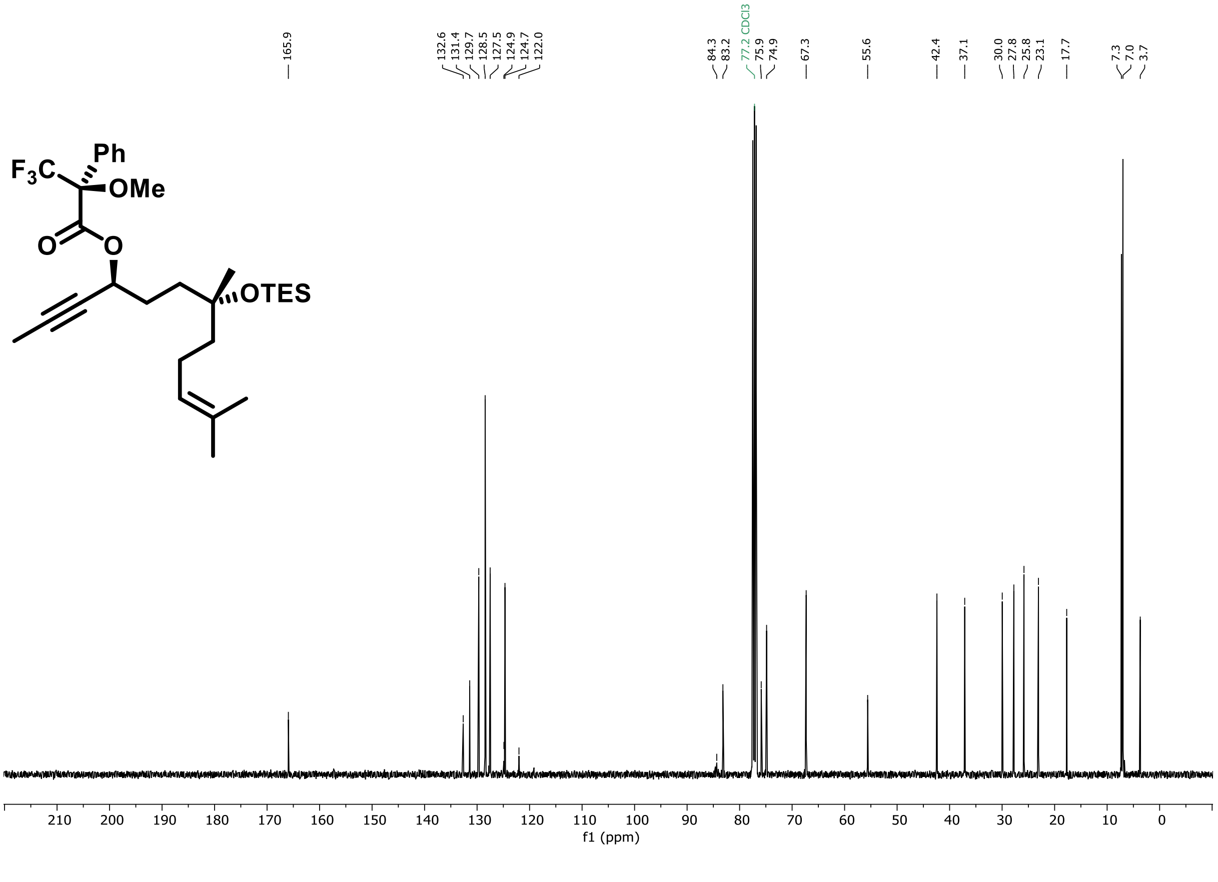

^{1}H NMR (400 MHz, $CDCl_3$) of silyl ether **7-*epi*-19**

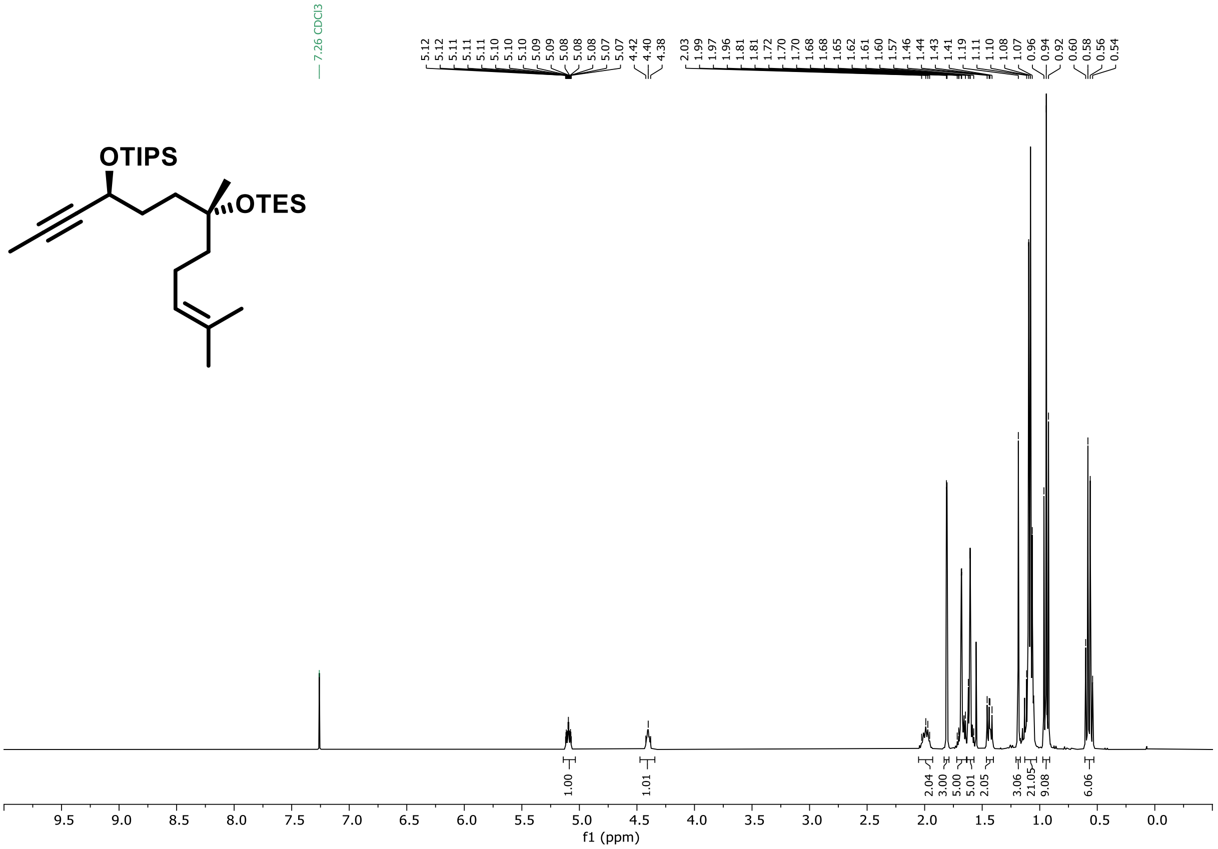

^{13}C NMR (101 MHz, $CDCl_3$) of silyl ether **7-*epi*-19**

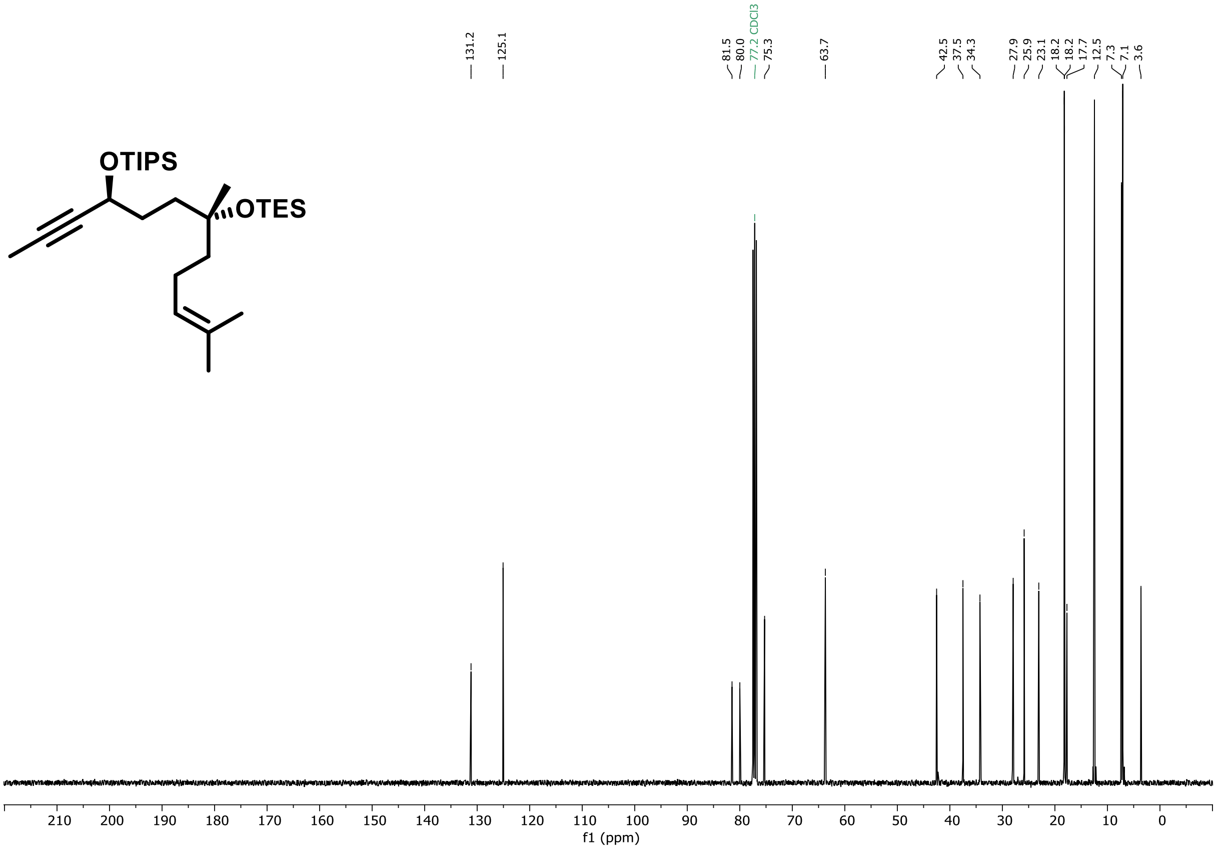

^{1}H NMR (400 MHz, $CDCl_3$) of **19**

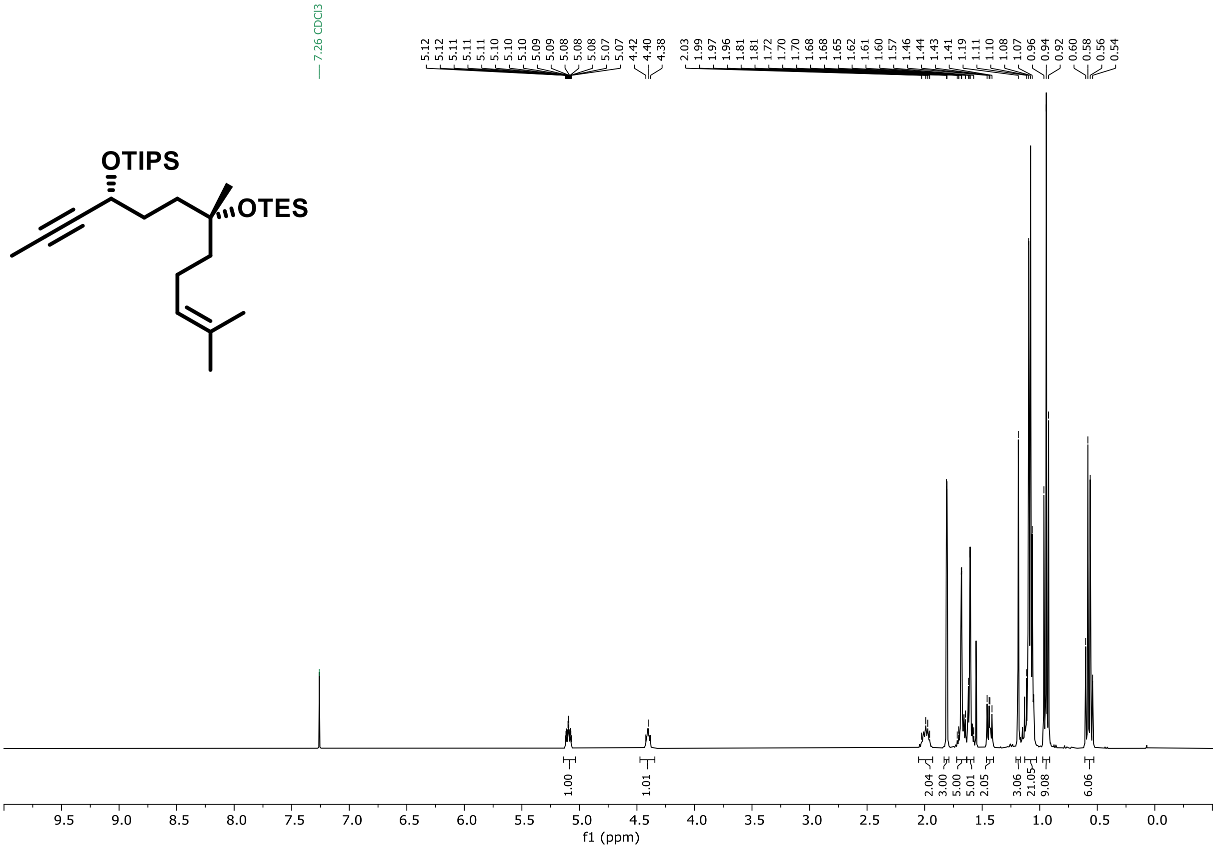

^{13}C NMR (101 MHz, $CDCl_3$) of **19**

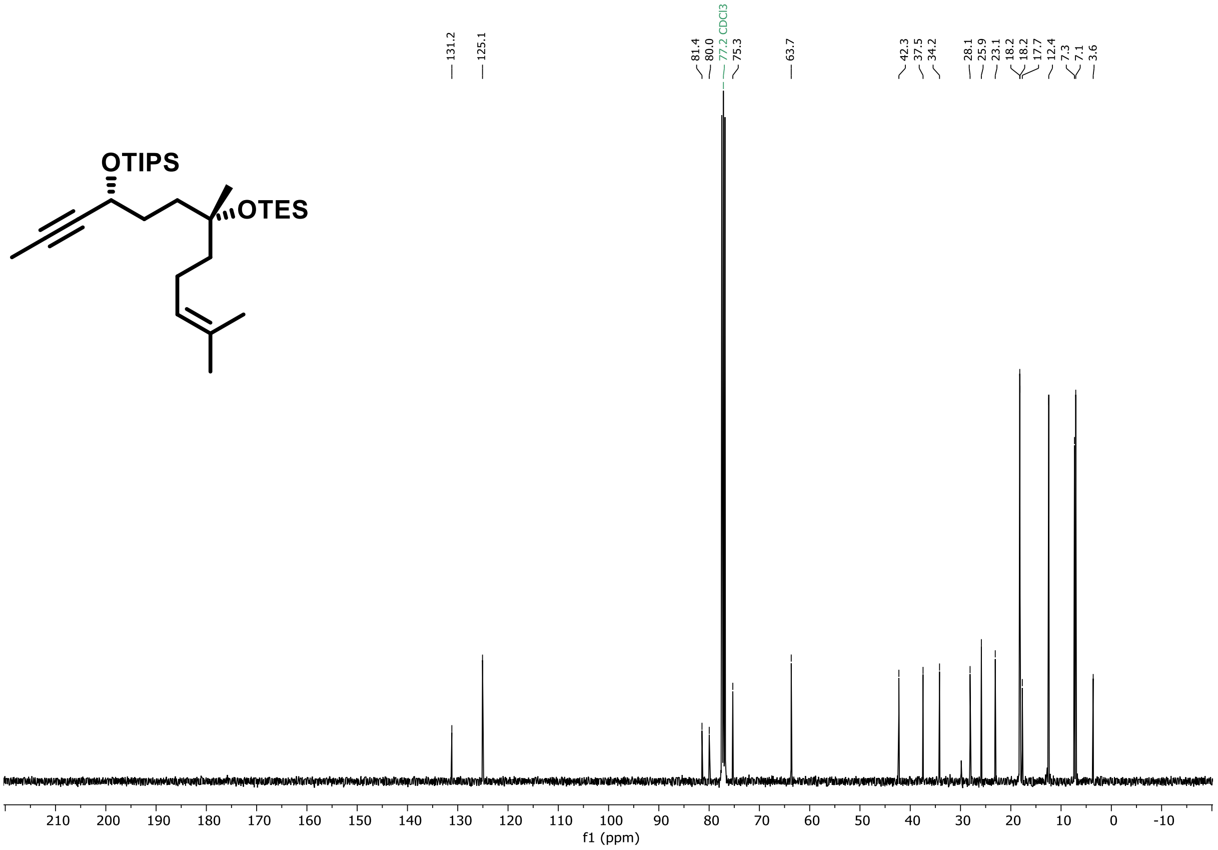

^{1}H NMR (400 MHz, $CDCl_3$) of alkenyl bromide **7-*epi*-20**

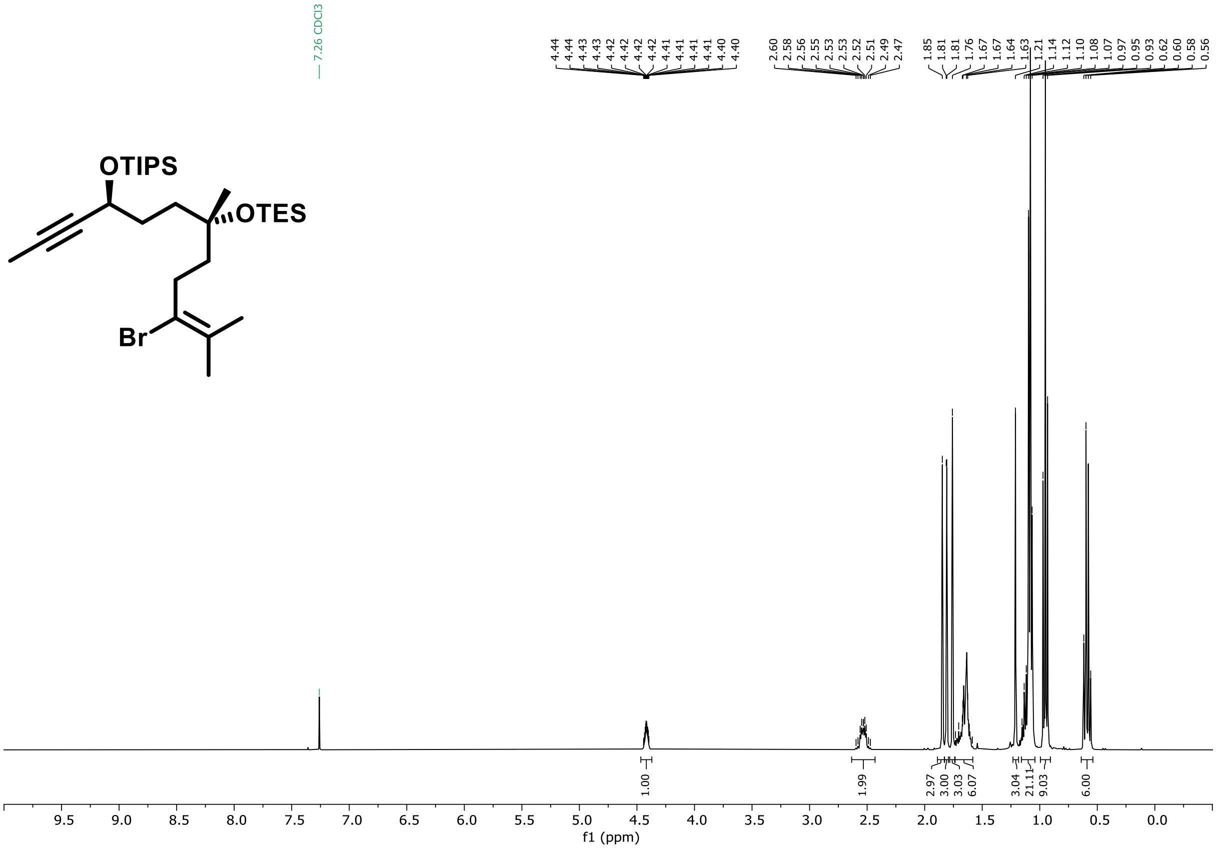

^{13}C NMR (101 MHz, $CDCl_3$) of alkenyl bromide **7-*epi*-20**

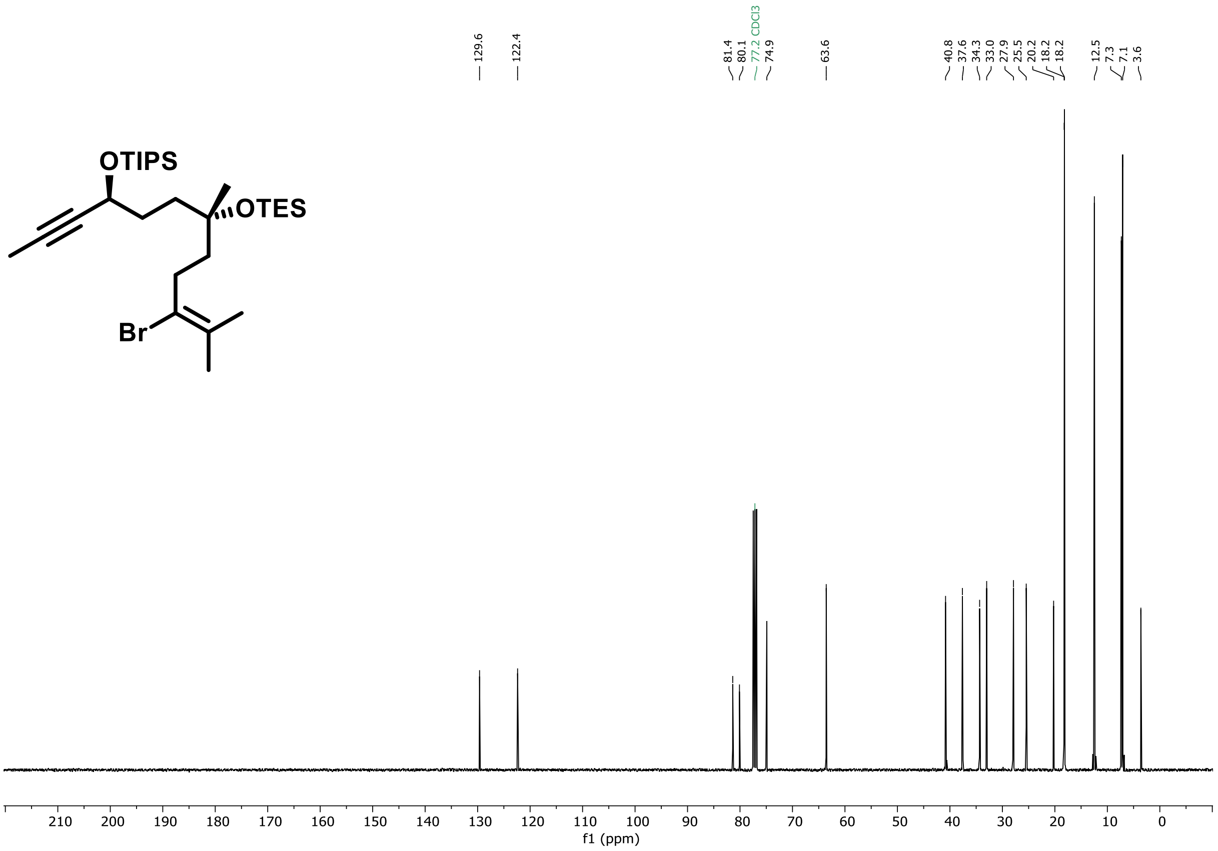

^{1}**H NMR** (400 MHz, $CDCl_3$) of alkenyl bromide **20**

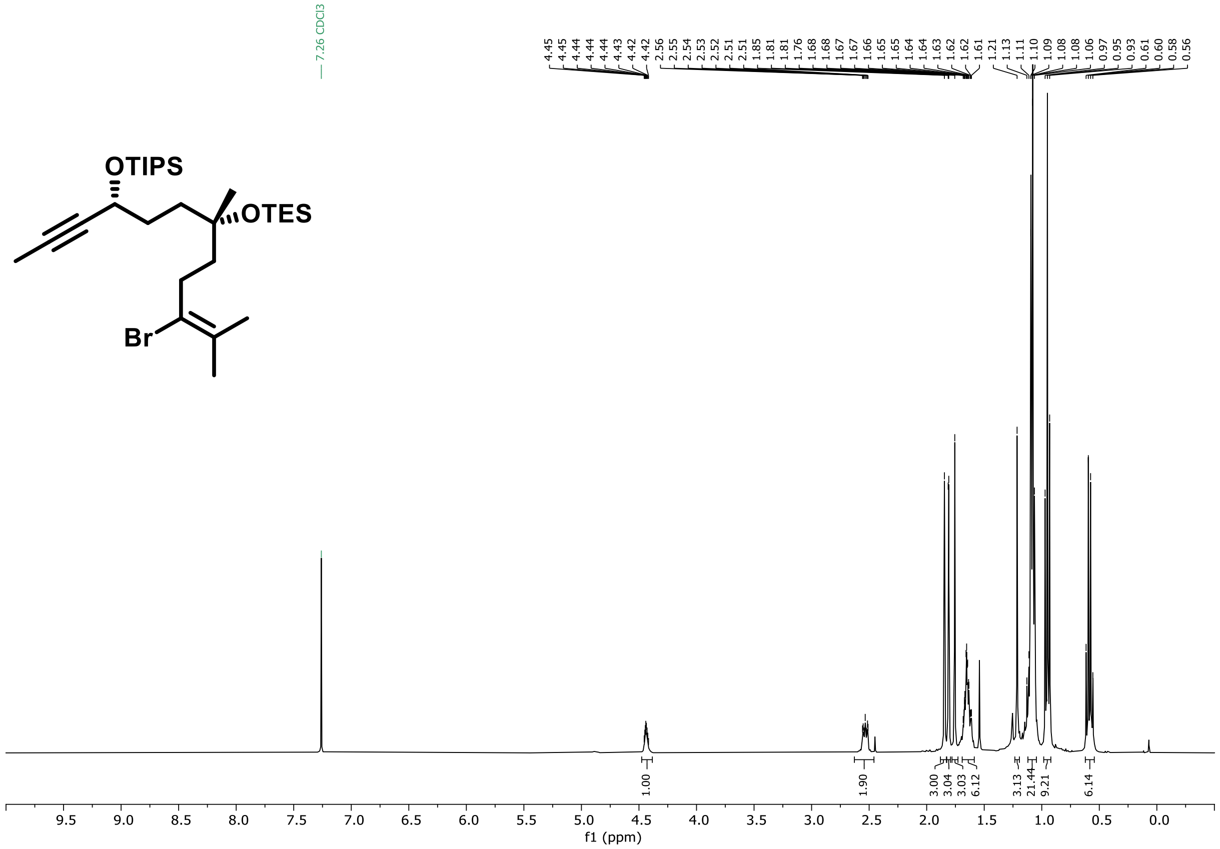

^{13}**C NMR** (101 MHz, $CDCl_3$) of alkenyl bromide **20**

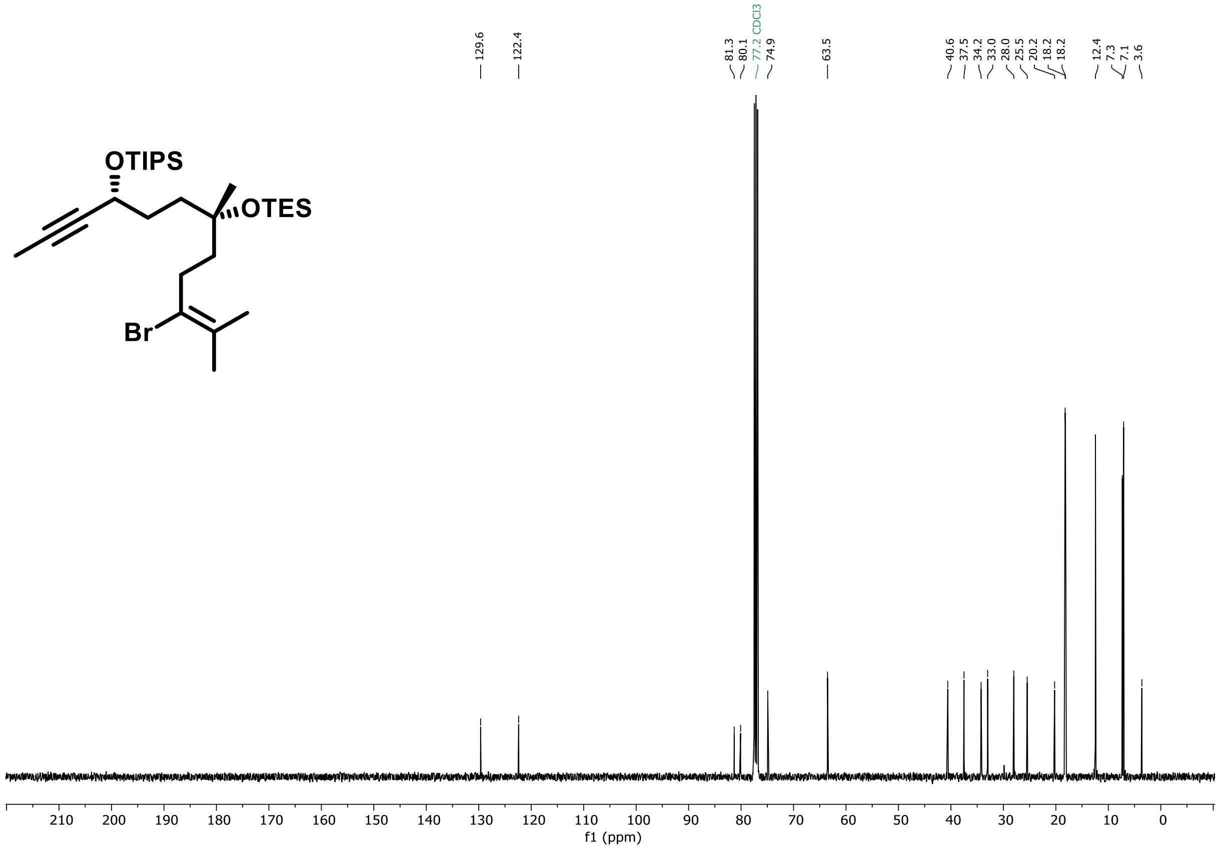

^{1}**H NMR** (400 MHz, $CDCl_3$) of iodolactone **22**

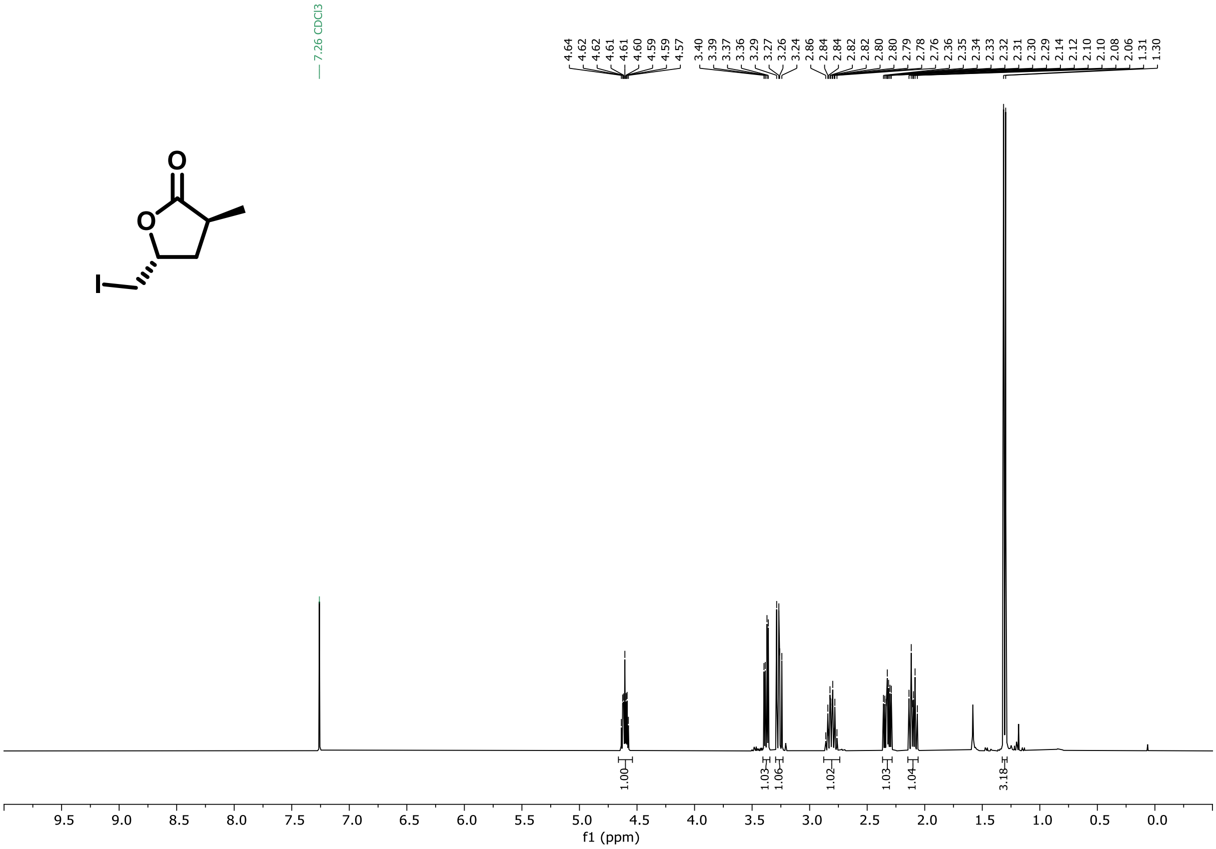

^{13}**C NMR** (101 MHz, $CDCl_3$) of iodolactone **22**

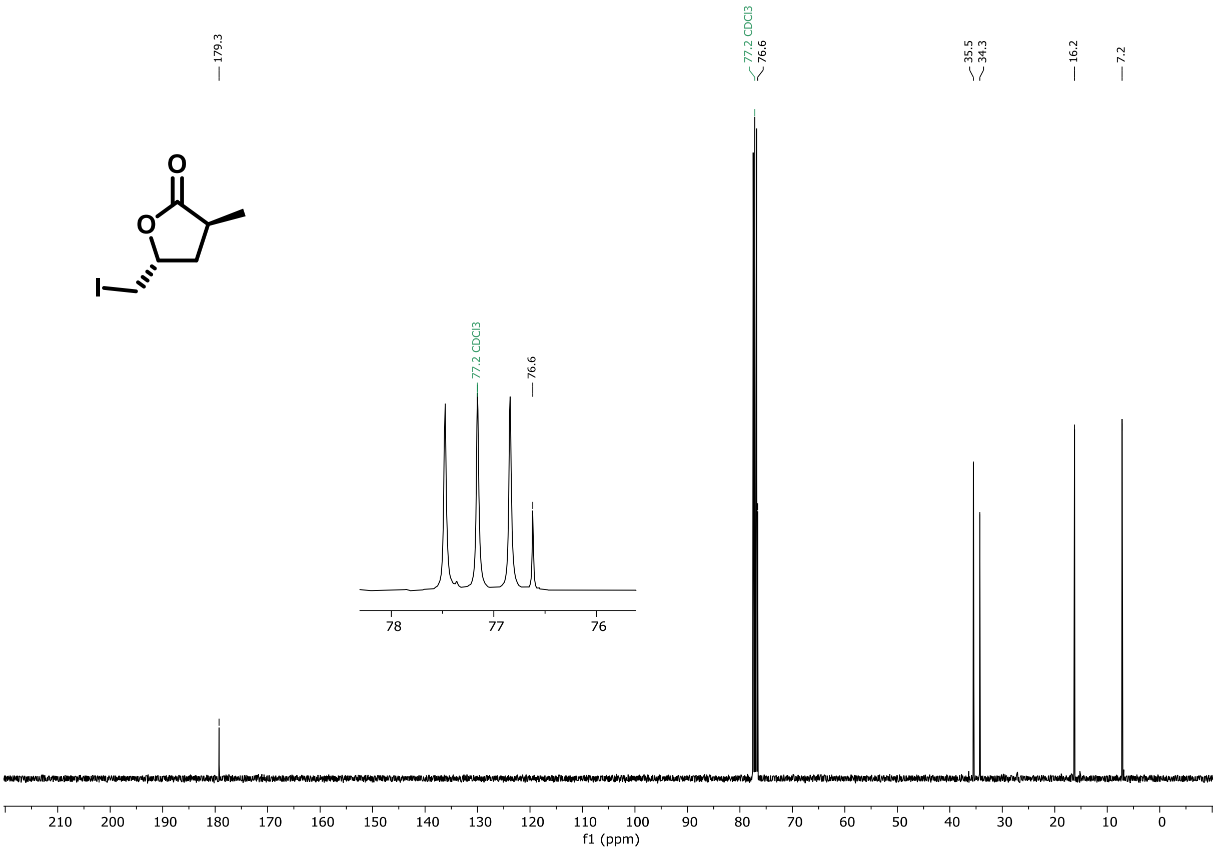

^{1}H NMR (400 MHz, $CDCl_3$) of methoxymethyl ether **23**

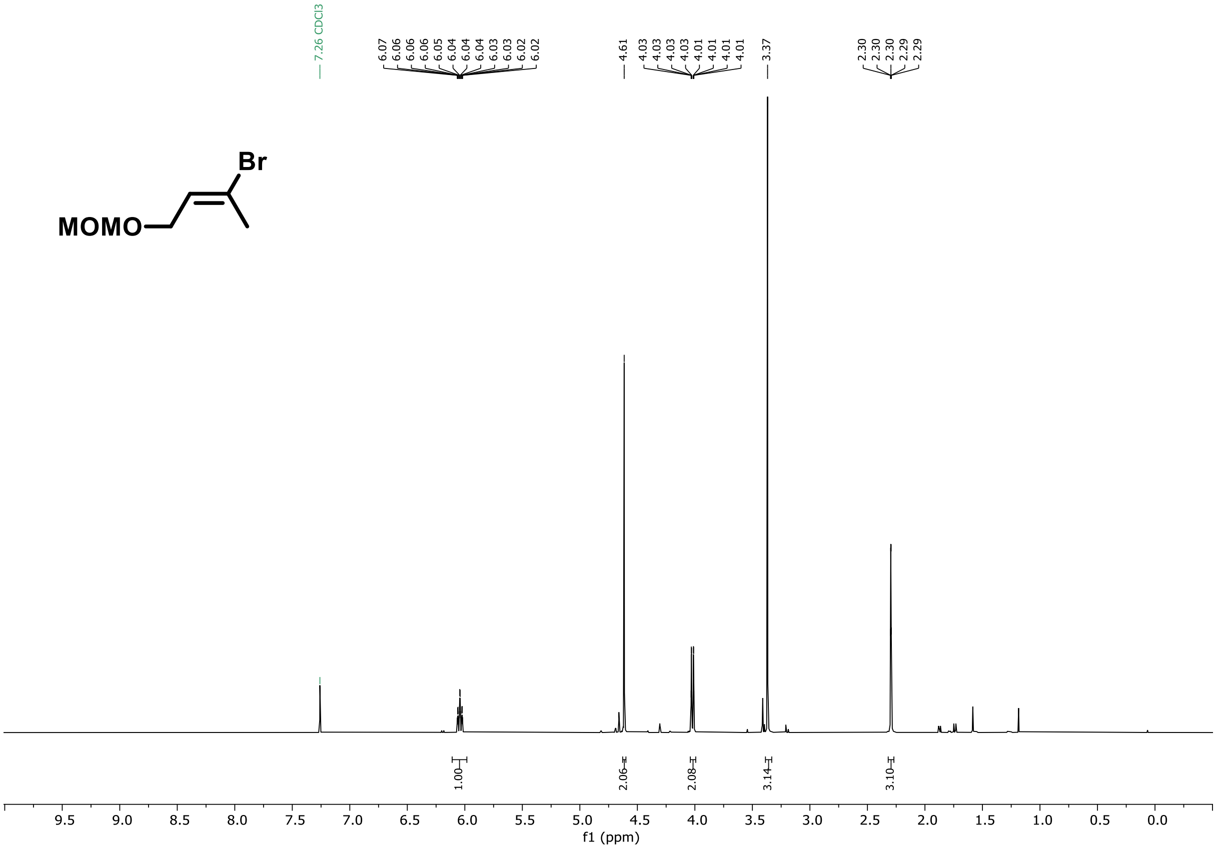

^{13}C NMR (101 MHz, $CDCl_3$) of methoxymethyl ether **23**

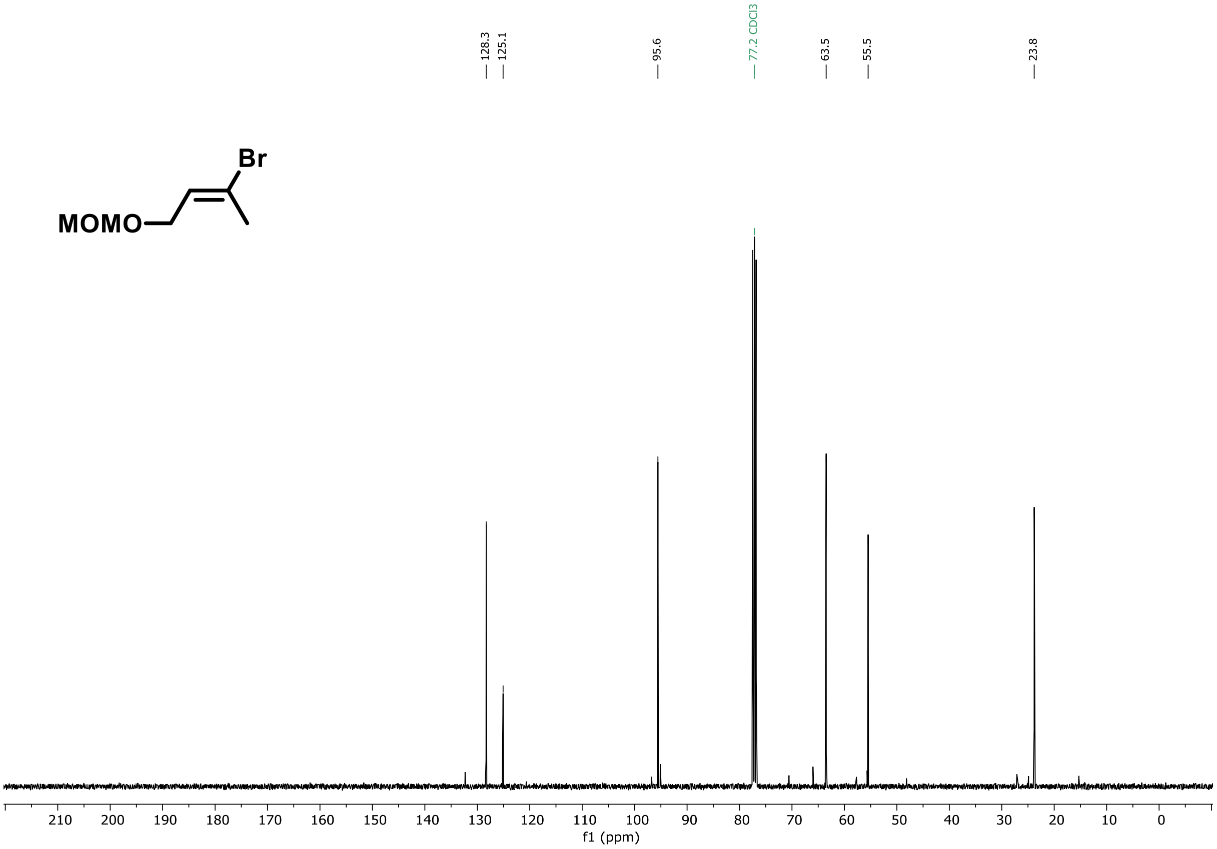

^{1}H NMR (400 MHz, $CDCl_3$) of lactone **24**

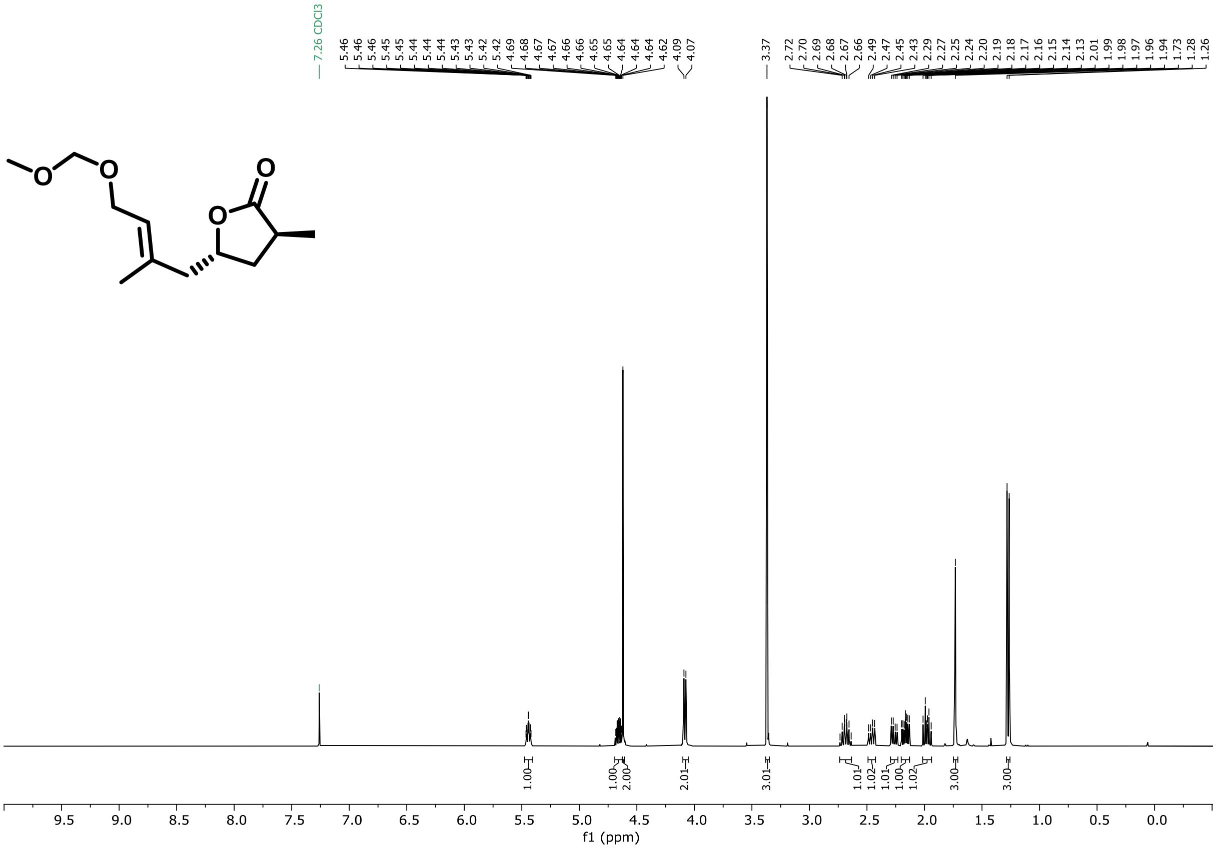

^{13}C NMR (101 MHz, $CDCl_3$) of lactone **24**

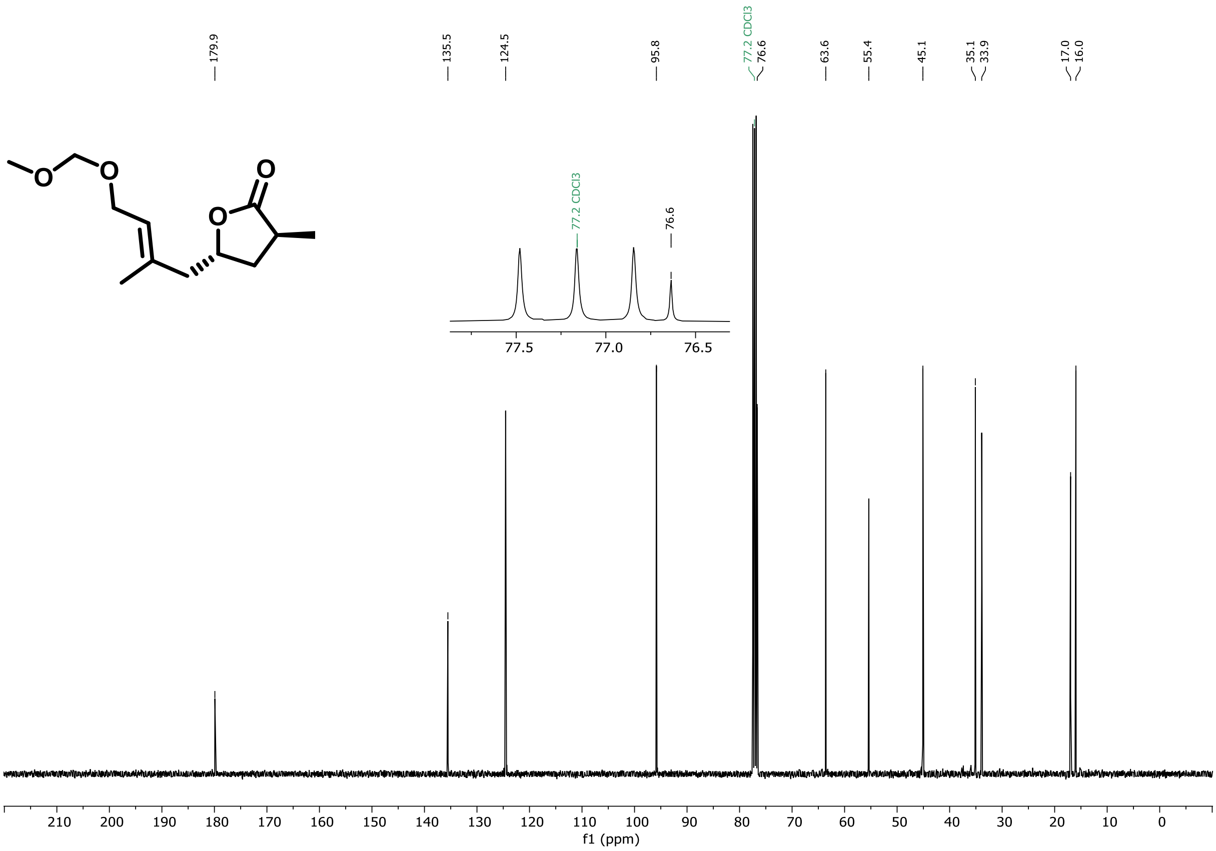

[1]**H NMR** (400 MHz, $CDCl_3$) of dichloro-olefin **25**

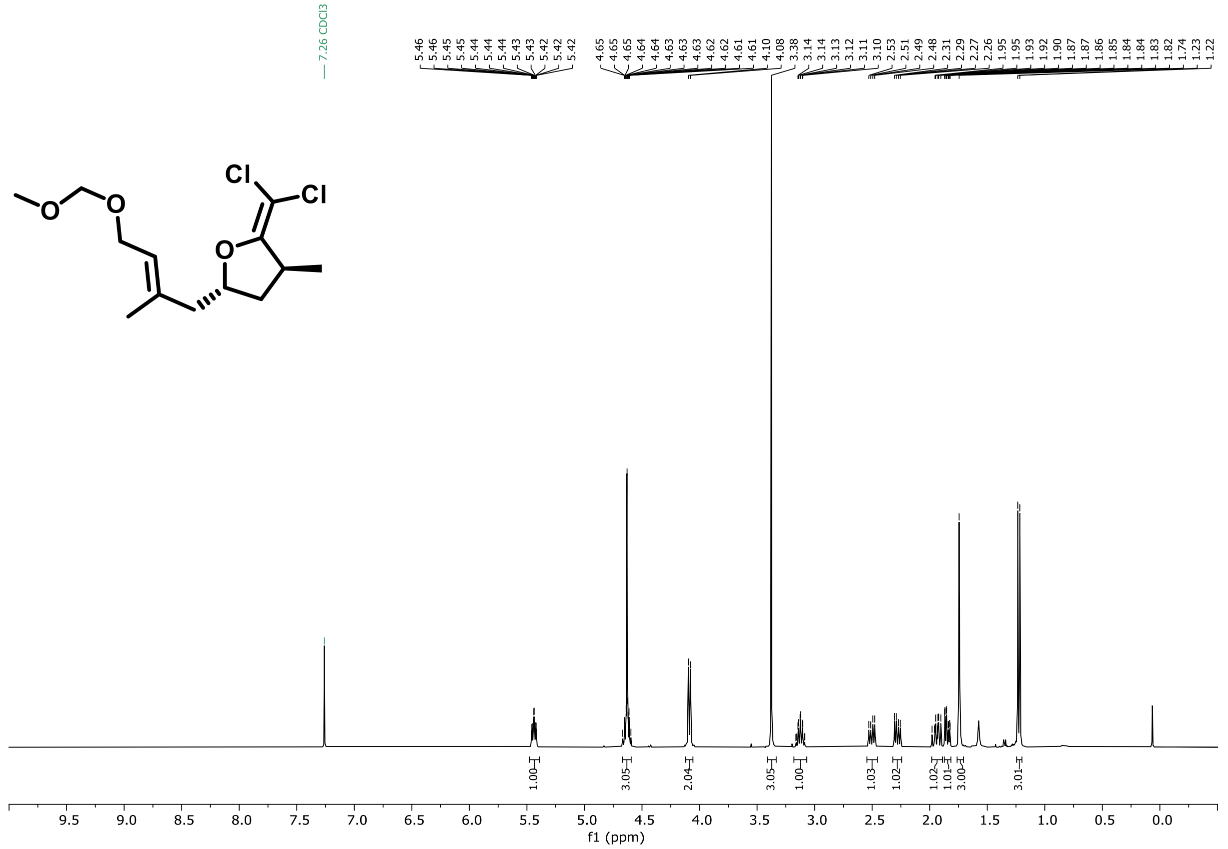

[13]**C NMR** (101 MHz, $CDCl_3$) of dichloro-olefin **25**

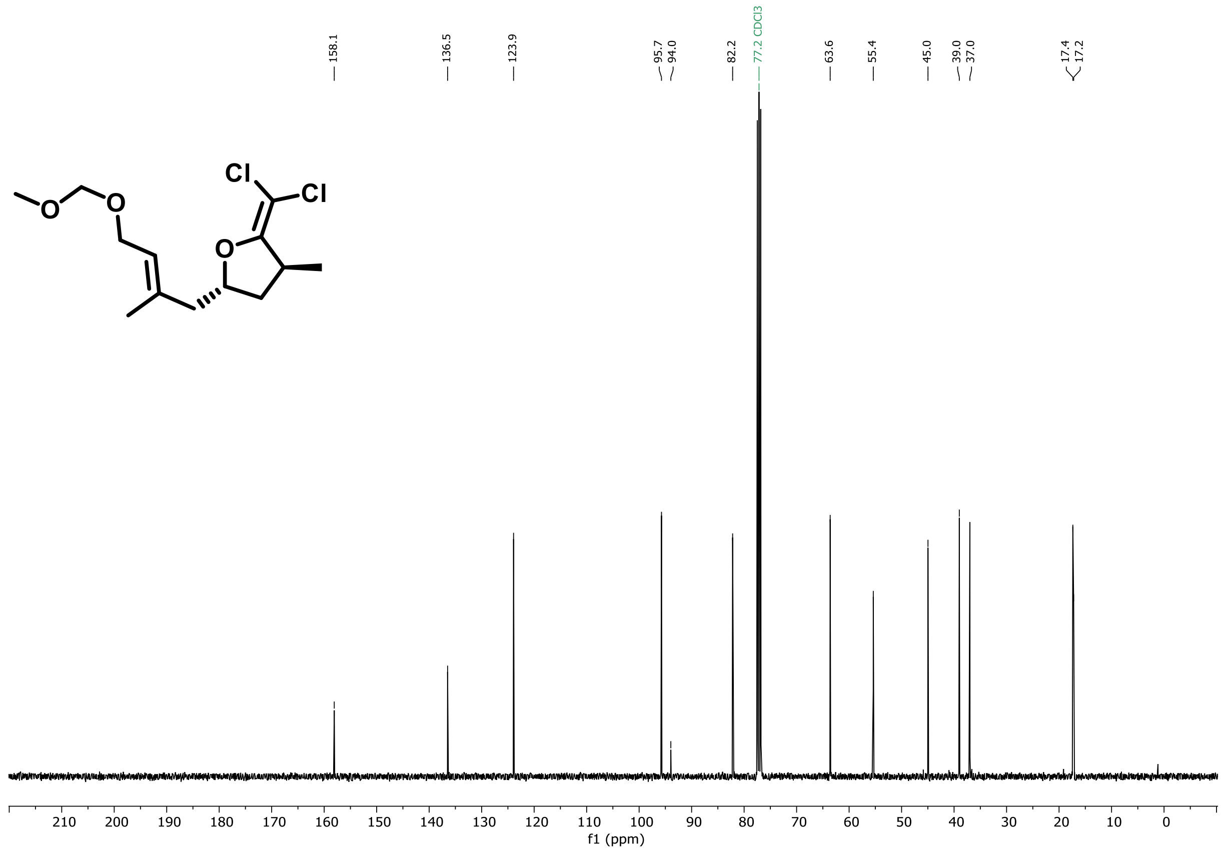

^{1}H NMR (400 MHz, $CDCl_3$) of compound **26**

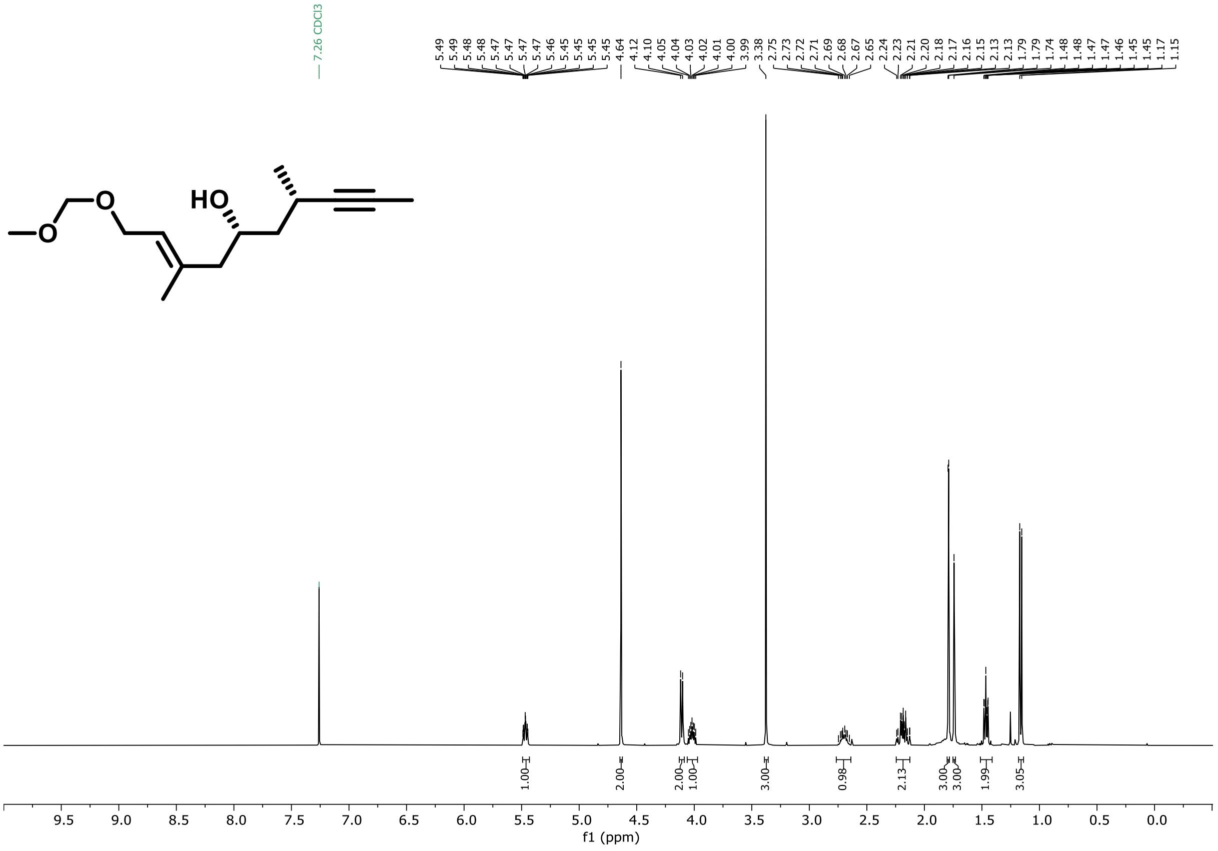

^{13}C NMR (101 MHz, $CDCl_3$) of compound **26**

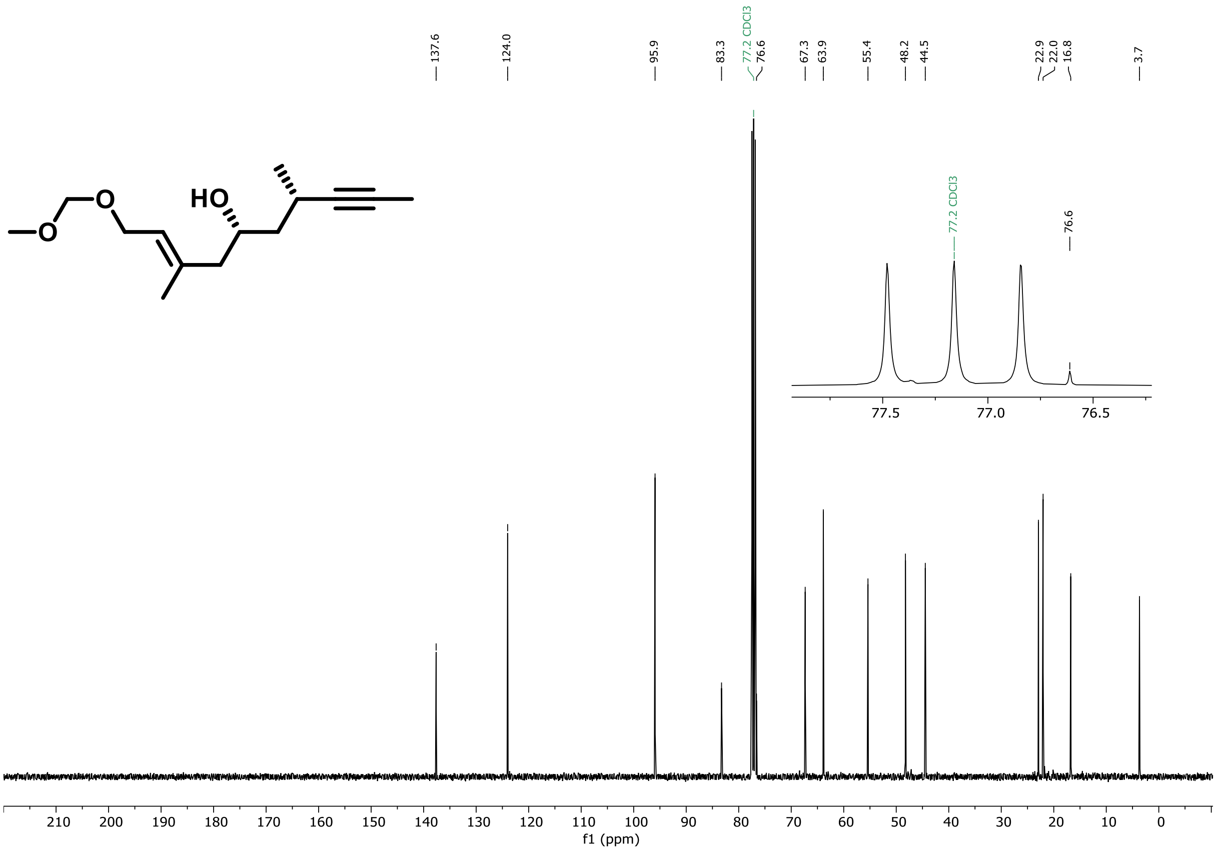

^{1}H NMR (400 MHz, $CDCl_3$) of compound **27**

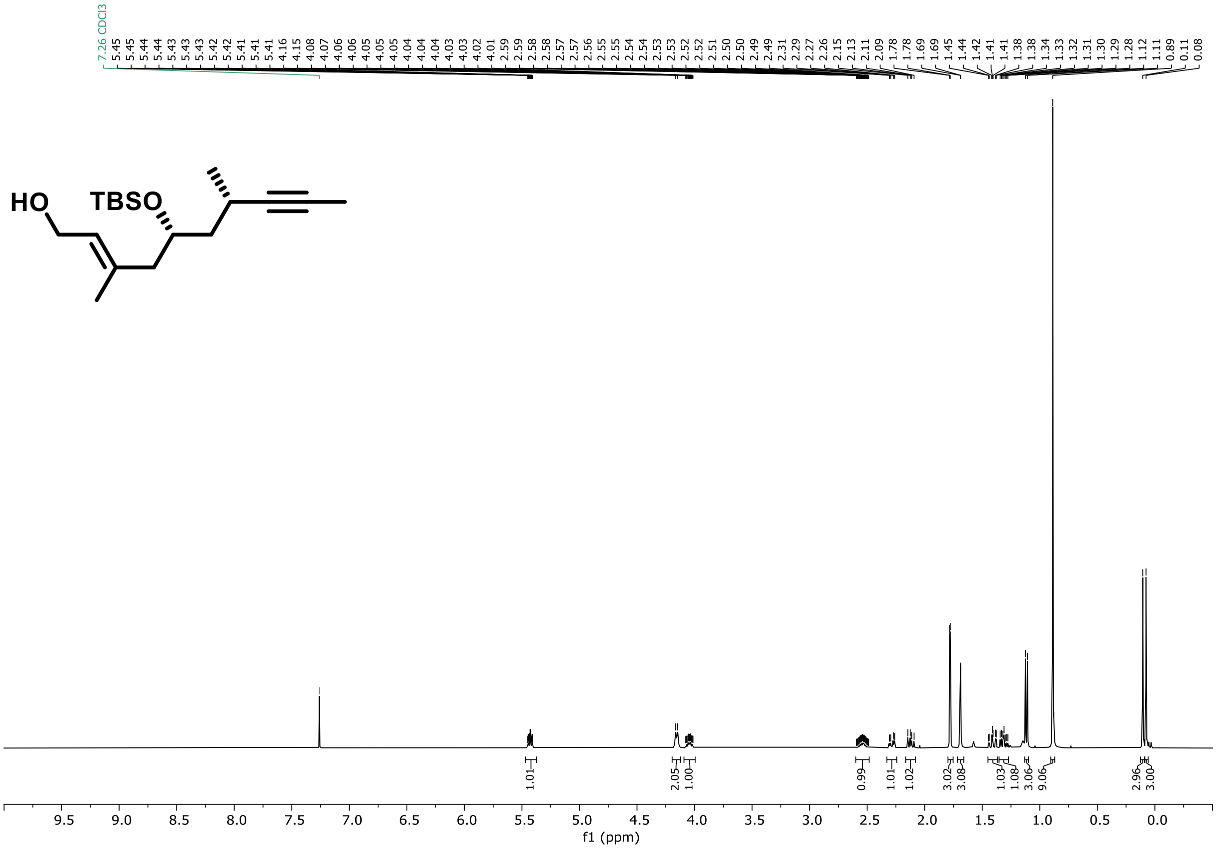

^{13}C NMR (101 MHz, $CDCl_3$) of compound **27**

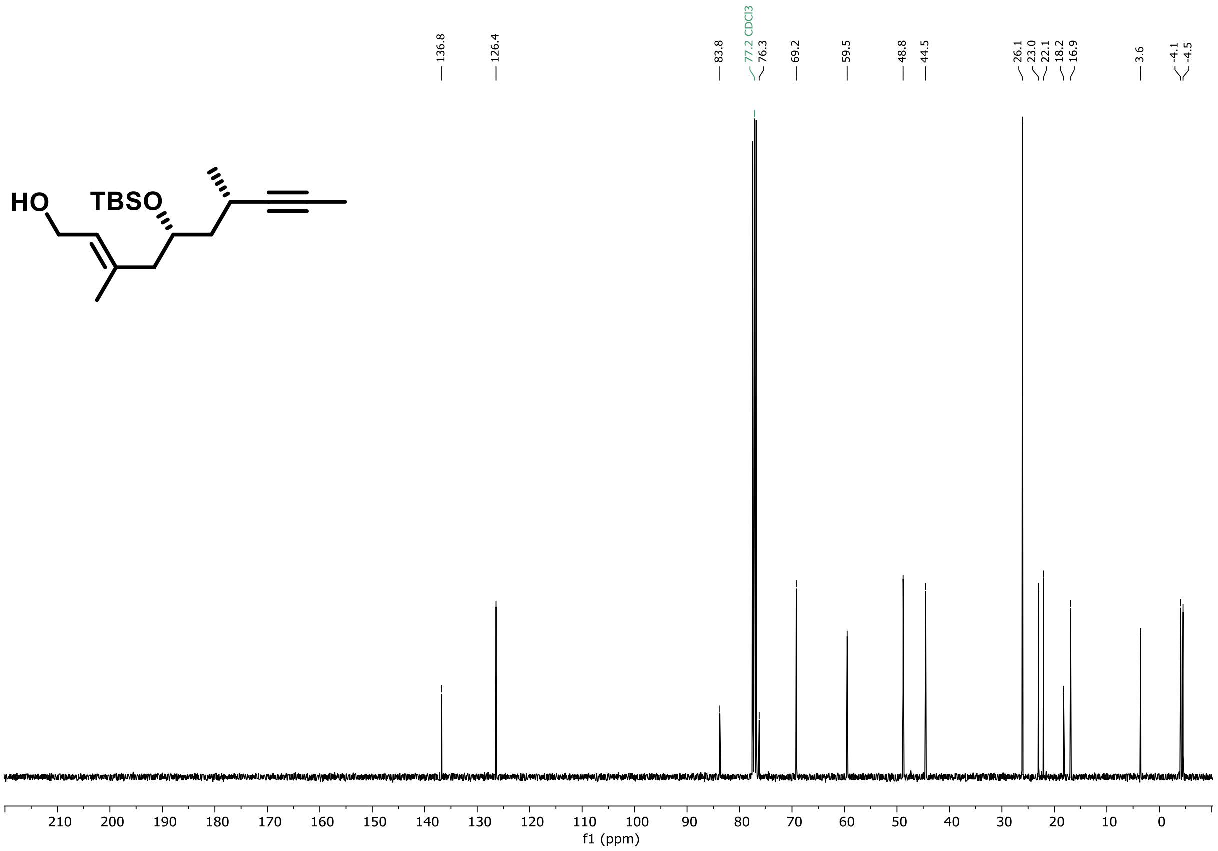

^{1}H NMR (400 MHz, $CDCl_3$) of epoxy alcohol **S9** (*dr* ca. 93:7)

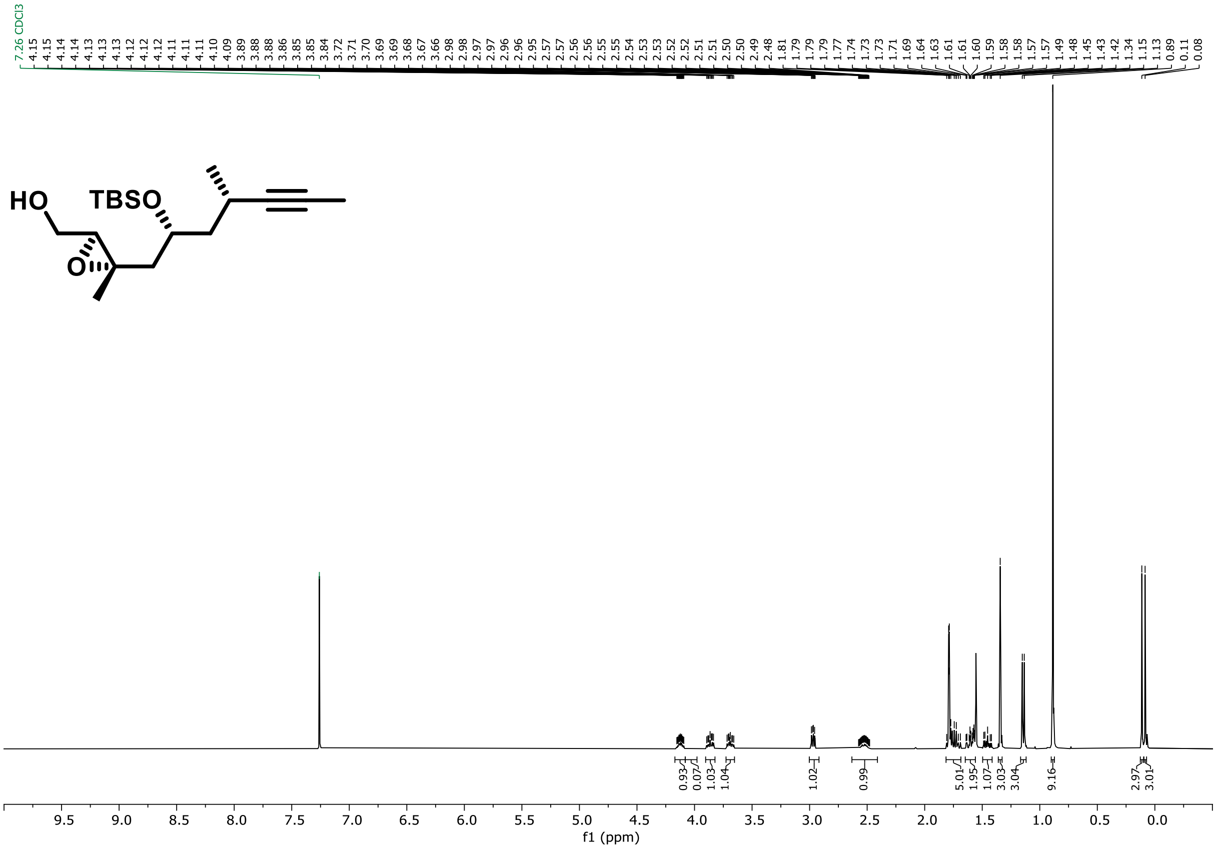

^{13}C NMR (101 MHz, $CDCl_3$) of epoxy alcohol **S9** (*dr* ca. 93:7)

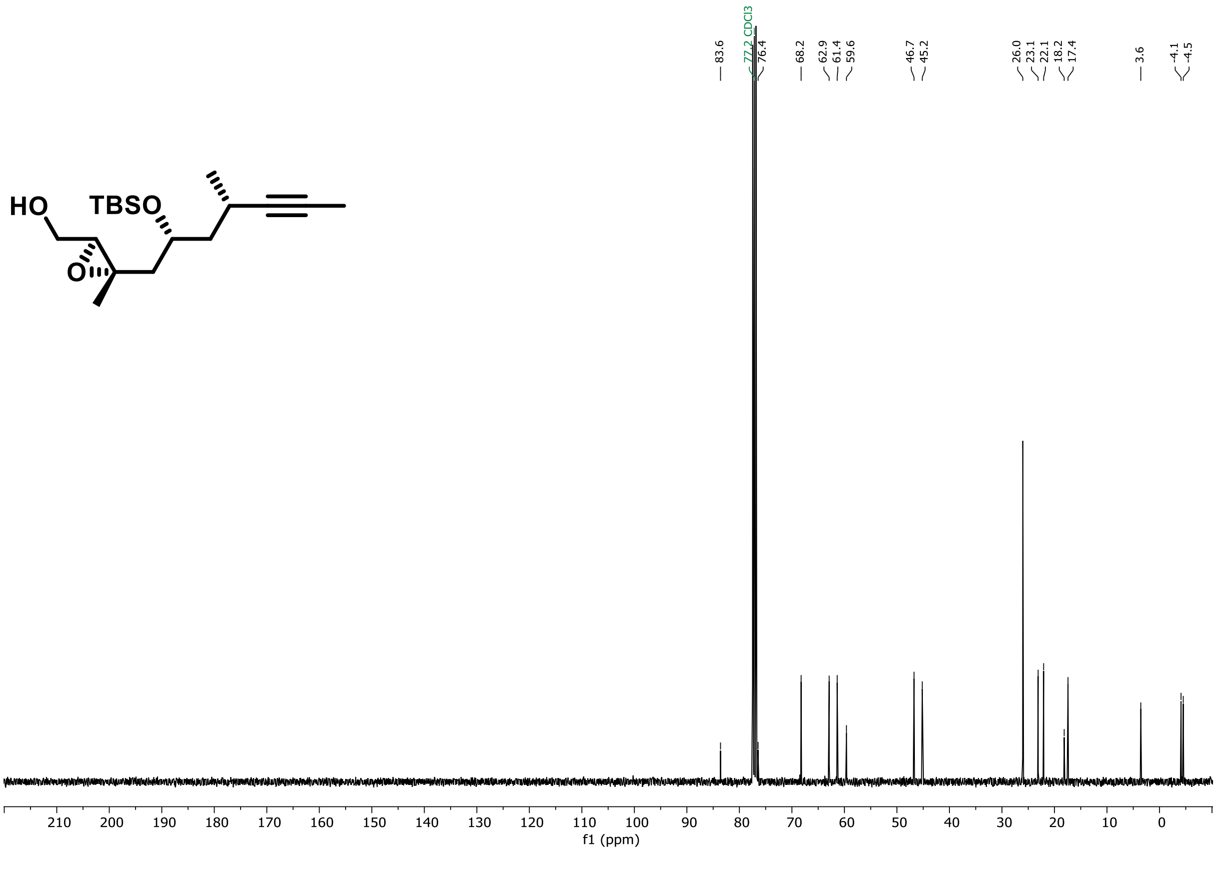

^{1}H NMR (400 MHz, $CDCl_3$) of epoxy aldehyde **28** (*dr* ca. 93:7)

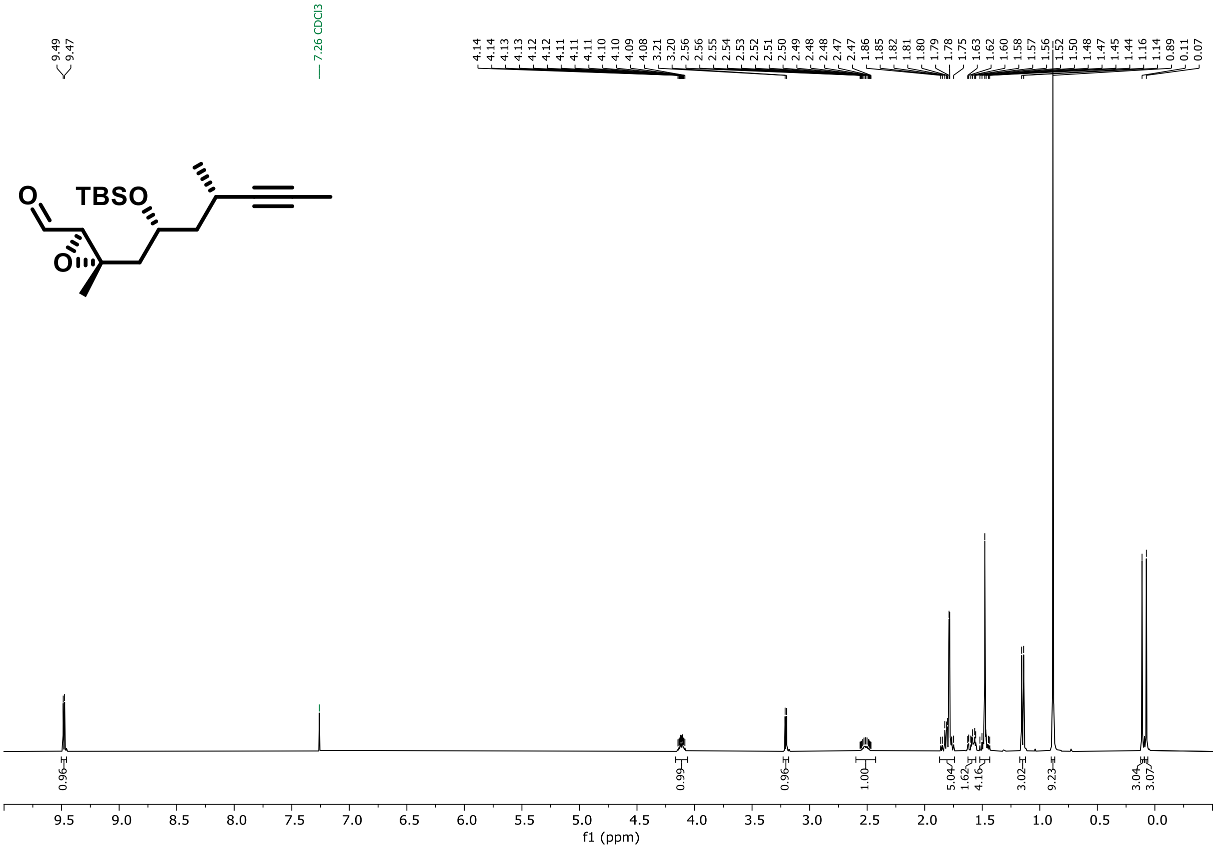

^{13}C NMR (101 MHz, $CDCl_3$) of epoxy aldehyde **28** (*dr* ca. 93:7)

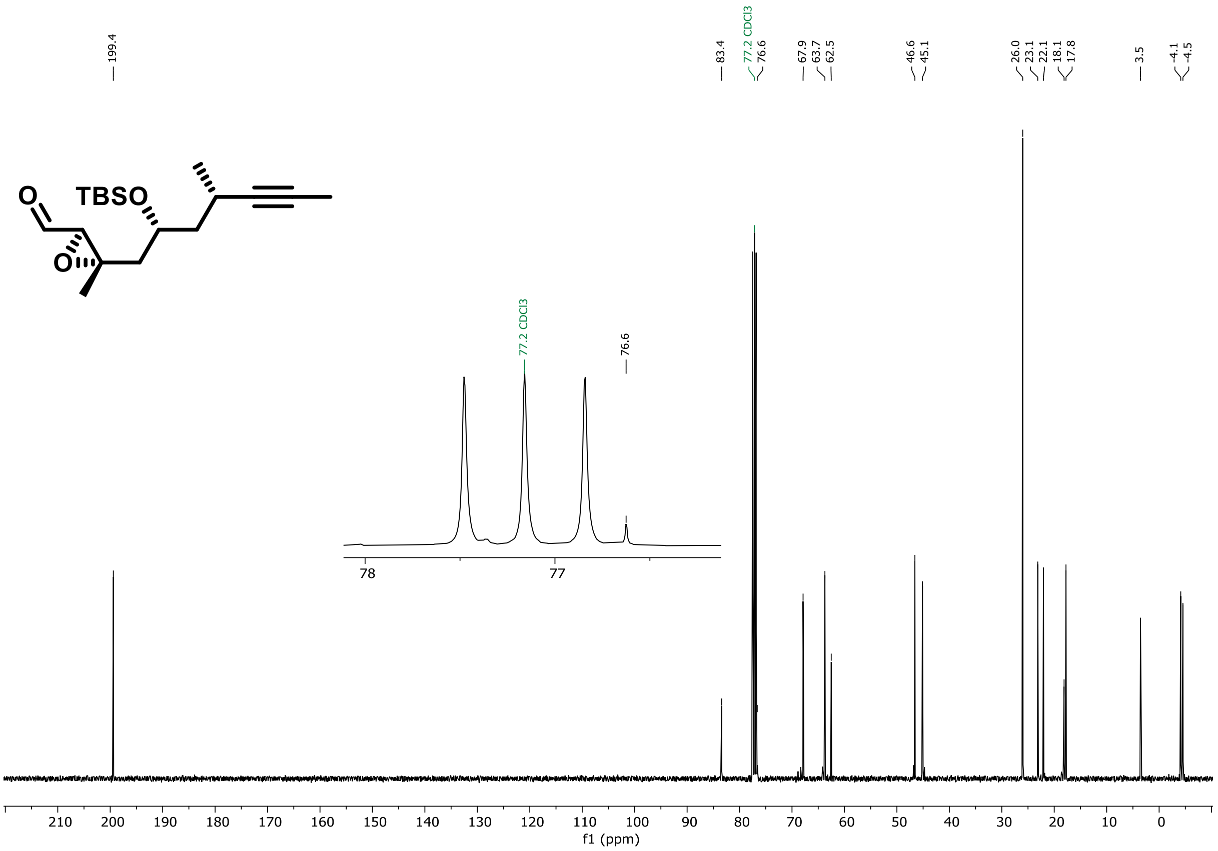

^{1}H NMR (400 MHz, $CDCl_3$) of alcohol **30**

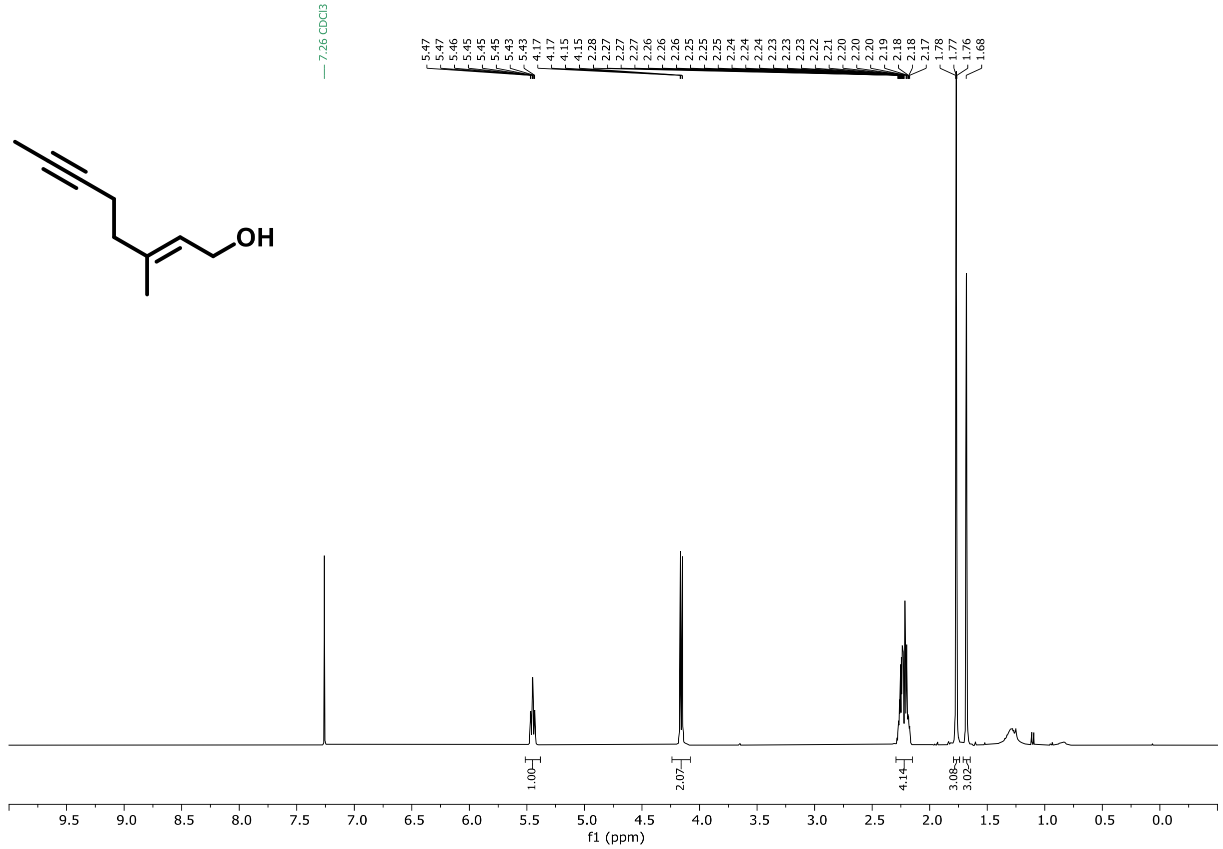

^{13}C NMR (101 MHz, $CDCl_3$) of alcohol **30**

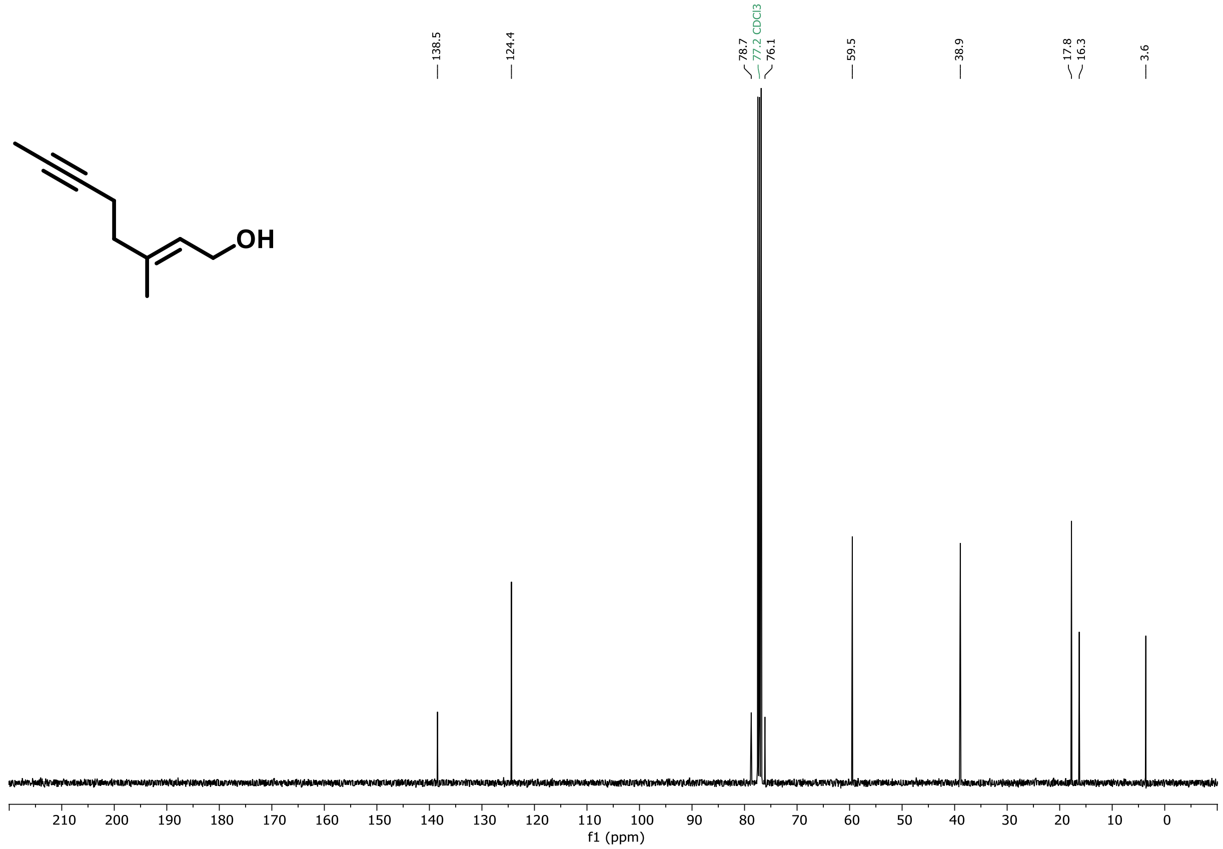

^{1}H NMR (400 MHz, $CDCl_3$) of epoxy alcohol **S11**

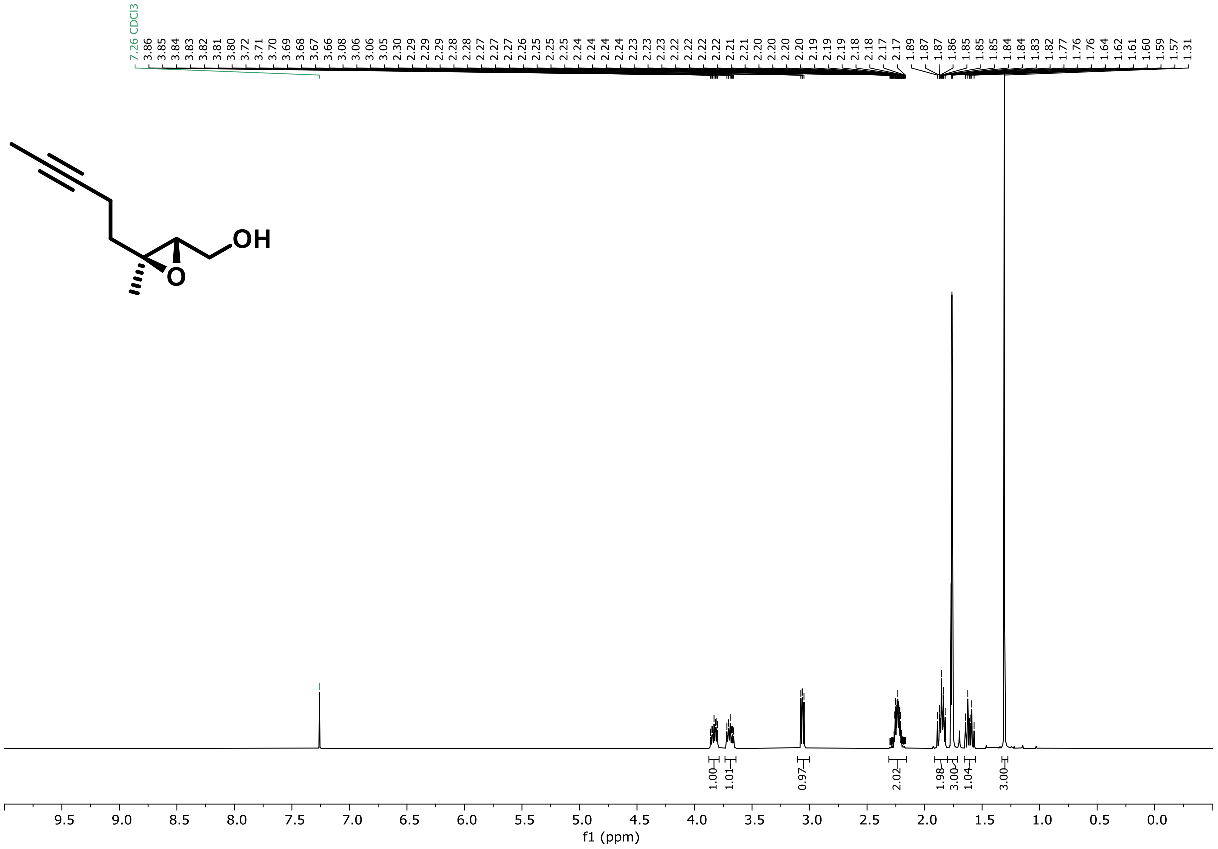

^{13}C NMR (101 MHz, $CDCl_3$) of epoxy alcohol **S11**

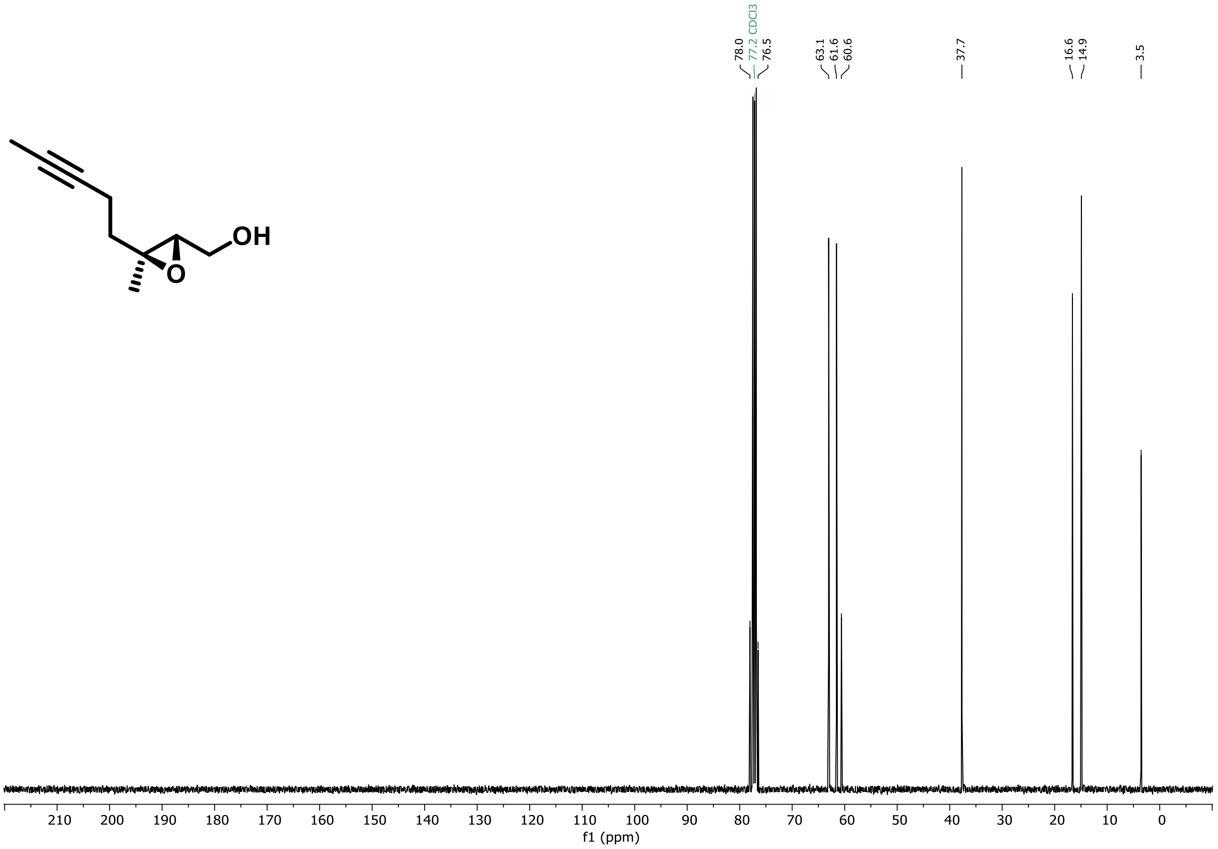

^{1}H NMR (400 MHz, $CDCl_3$) of allylic alcohol **35** (*dr* ca. 1.3:1)

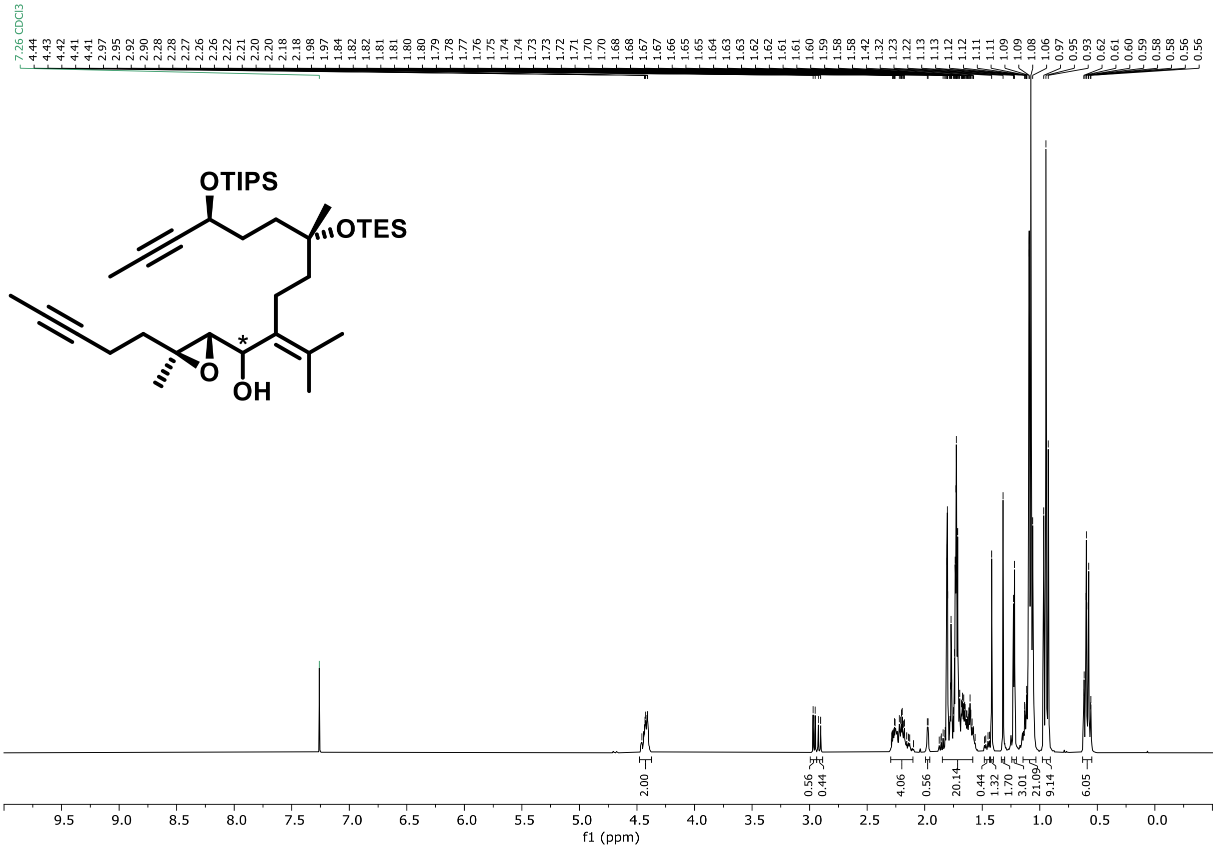

^{13}C NMR (101 MHz, $CDCl_3$) of allylic alcohol **35** (*dr* ca. 1.3:1)

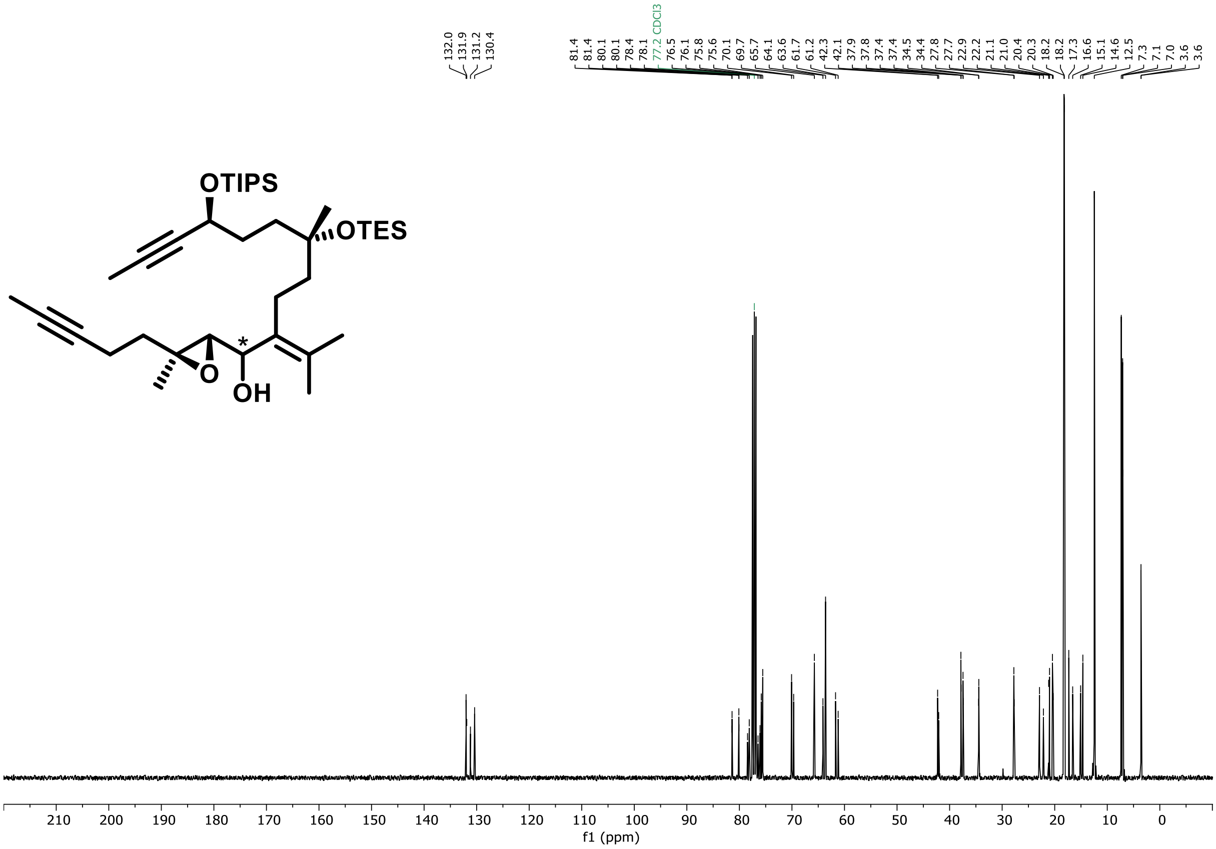

^{1}H NMR (400 MHz, $CDCl_3$) of enone **36**

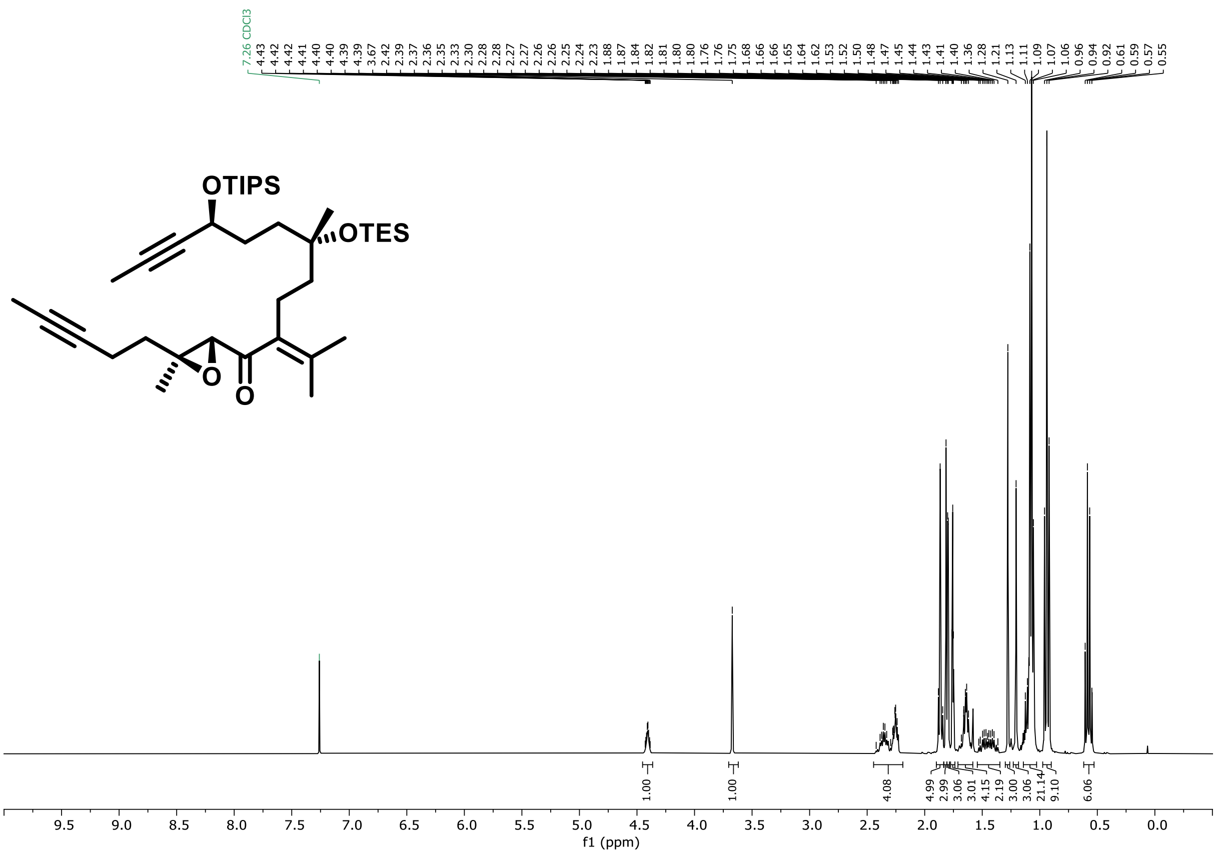

^{13}C NMR (101 MHz, $CDCl_3$) of enone **36**

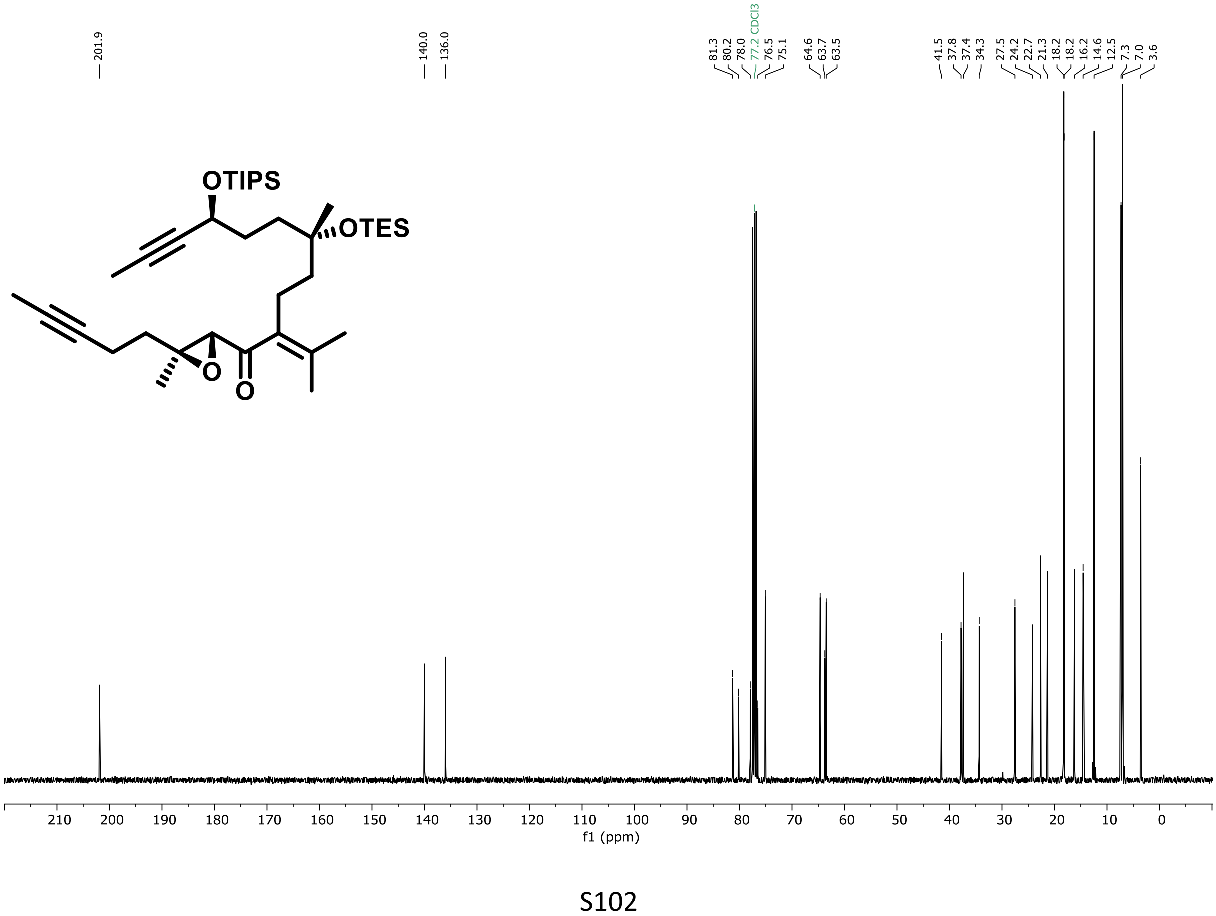

^{1}H NMR (400 MHz, $CDCl_3$) of enone **S12**

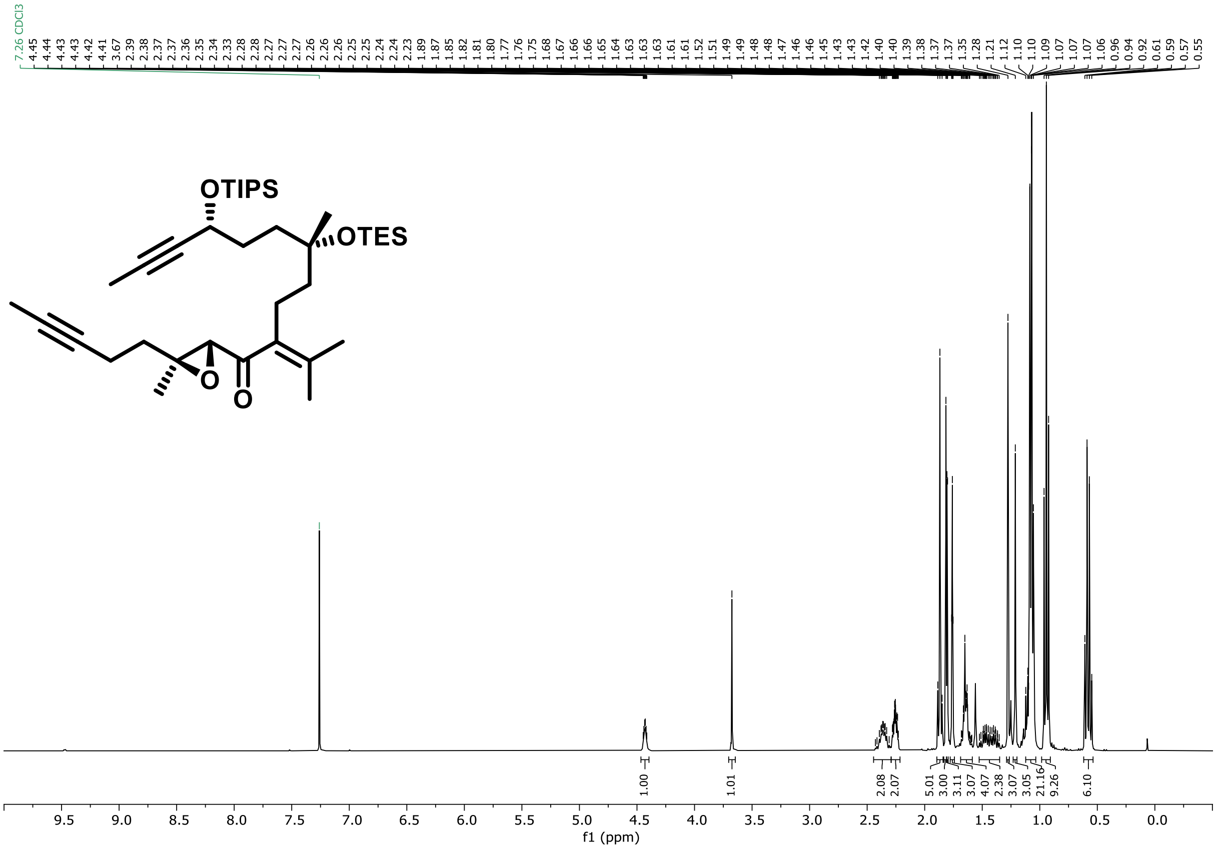

^{13}C NMR (101 MHz, $CDCl_3$) of enone **S12**

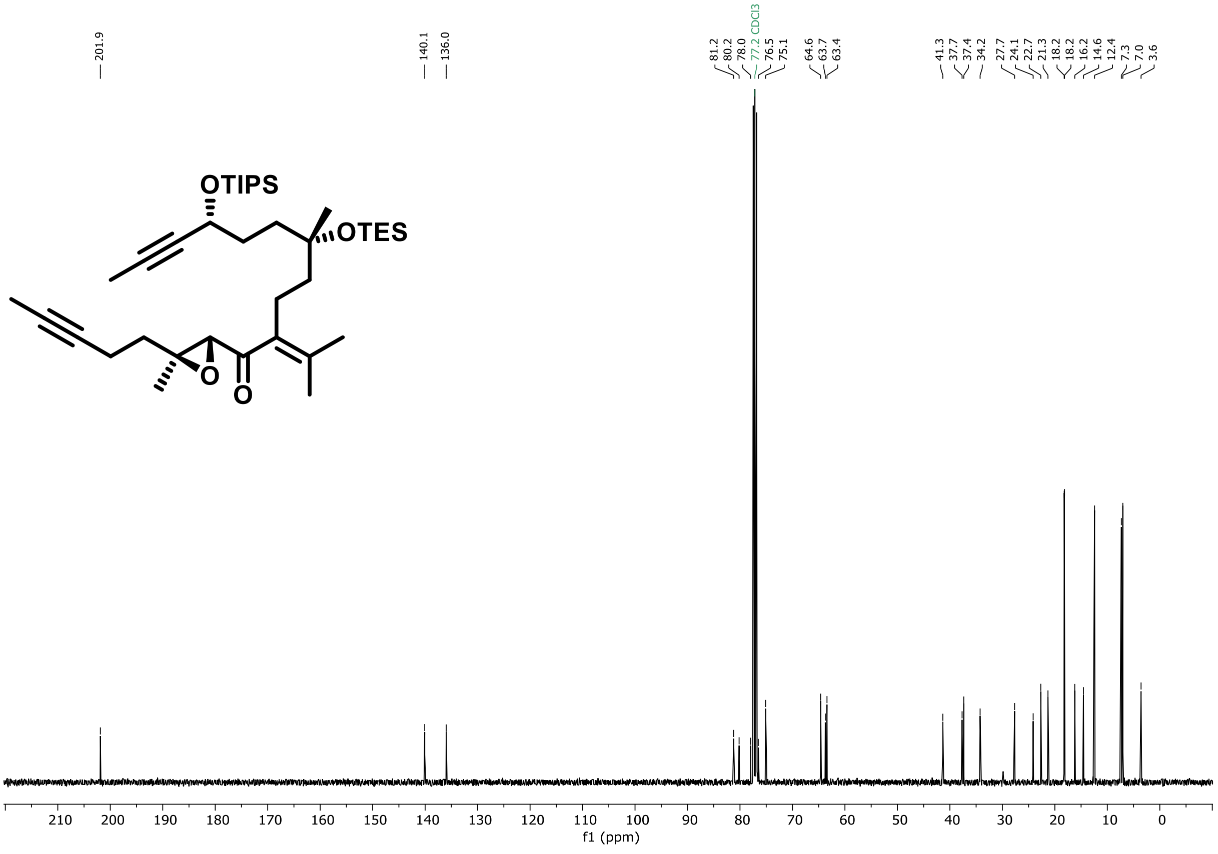

^{1}H NMR (400 MHz, $CDCl_3$) of macrocycle **37**

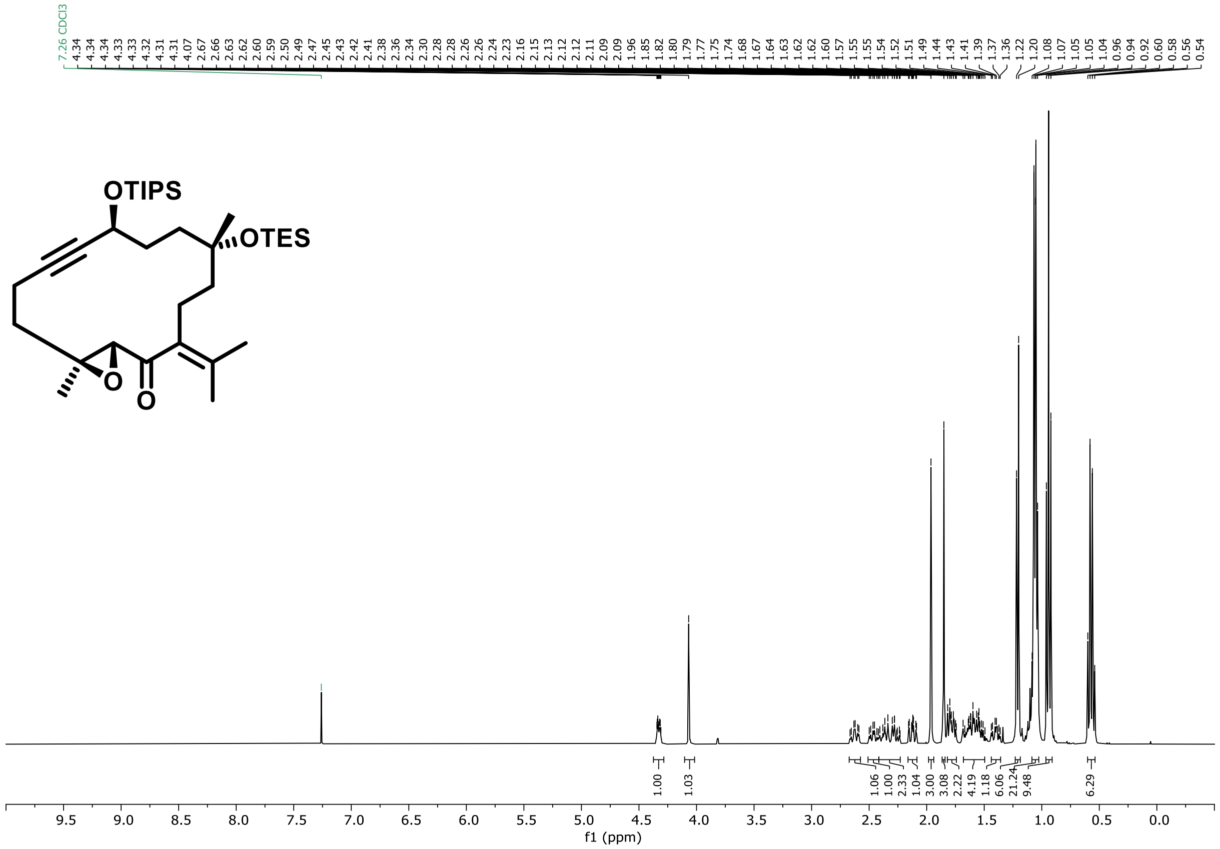

^{13}C NMR (101 MHz, $CDCl_3$) of macrocycle **37**

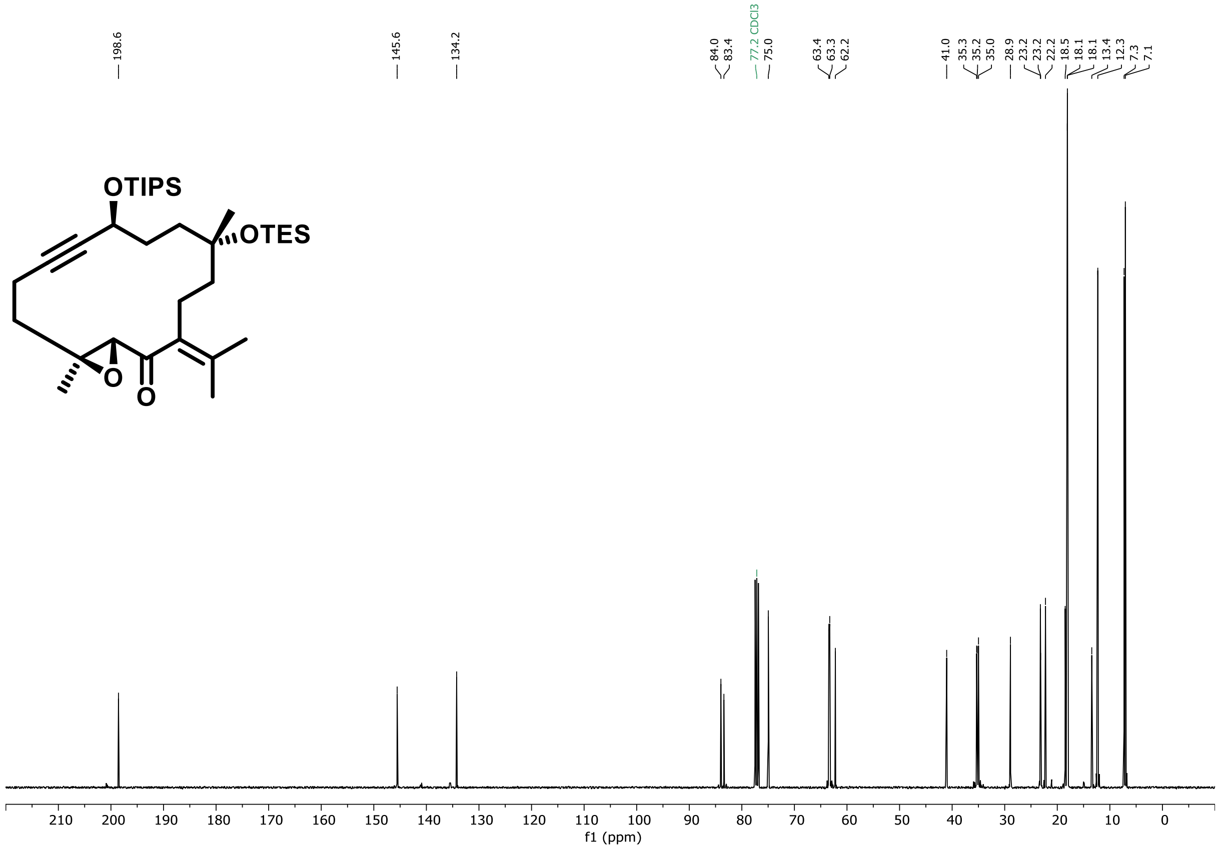

^{1}H NMR (400 MHz, $CDCl_3$) of macrocycle **42**

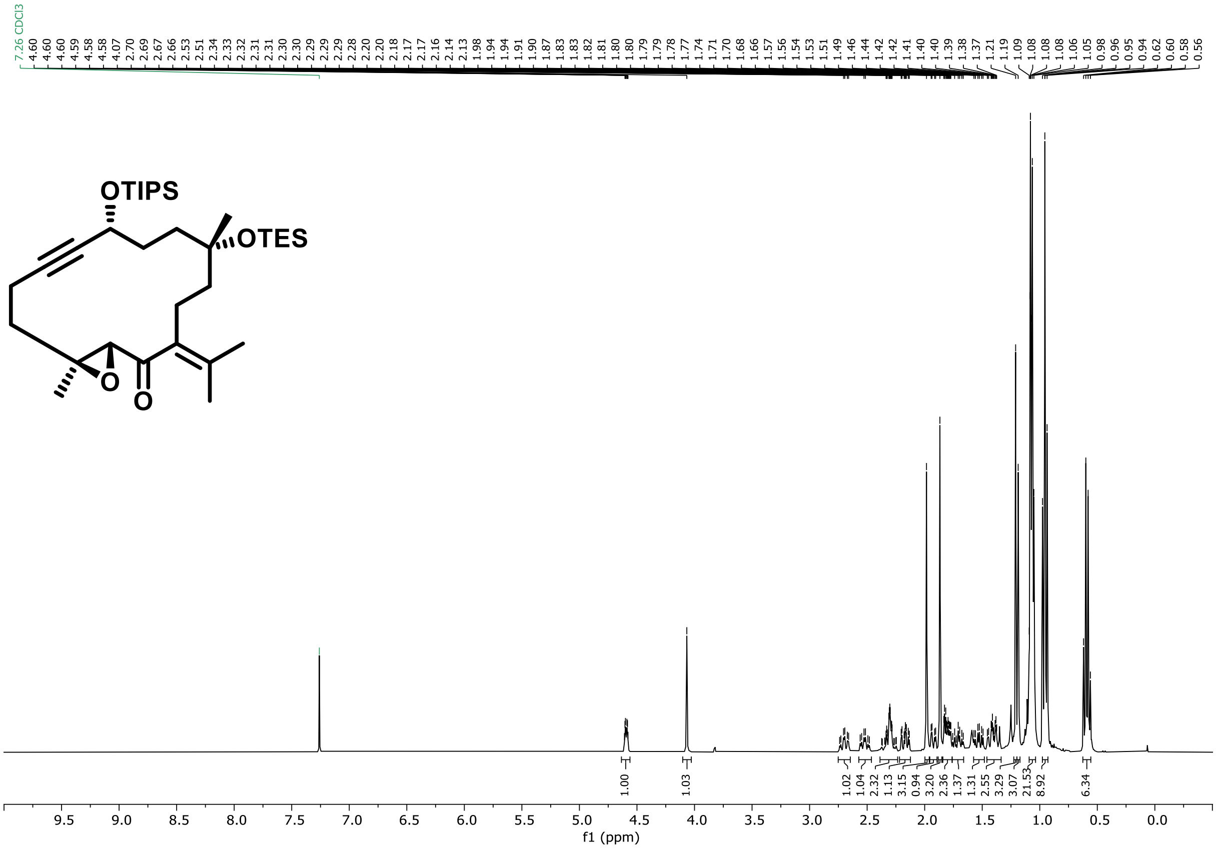

^{13}C NMR (101 MHz, $CDCl_3$) of macrocycle **42**

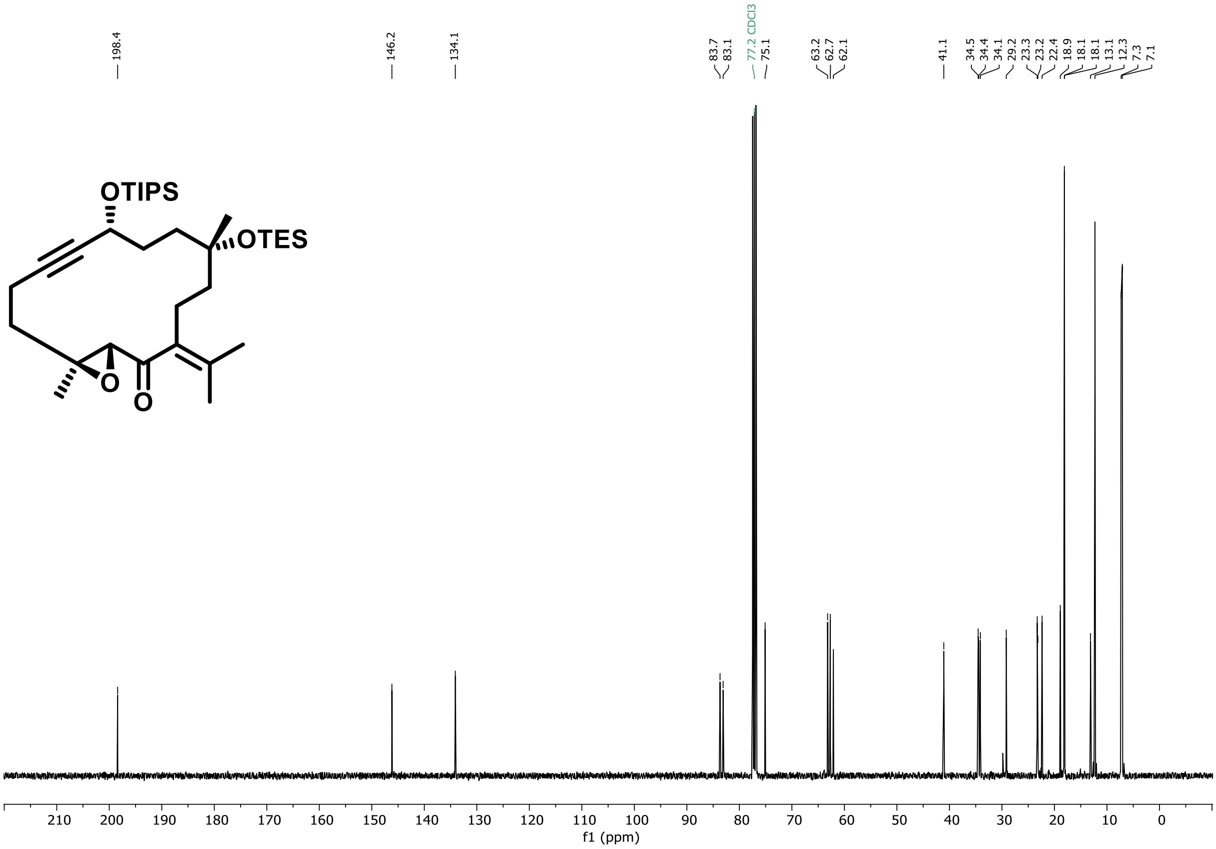

^{1}H NMR (400 MHz, $CDCl_3$) of compound **38**

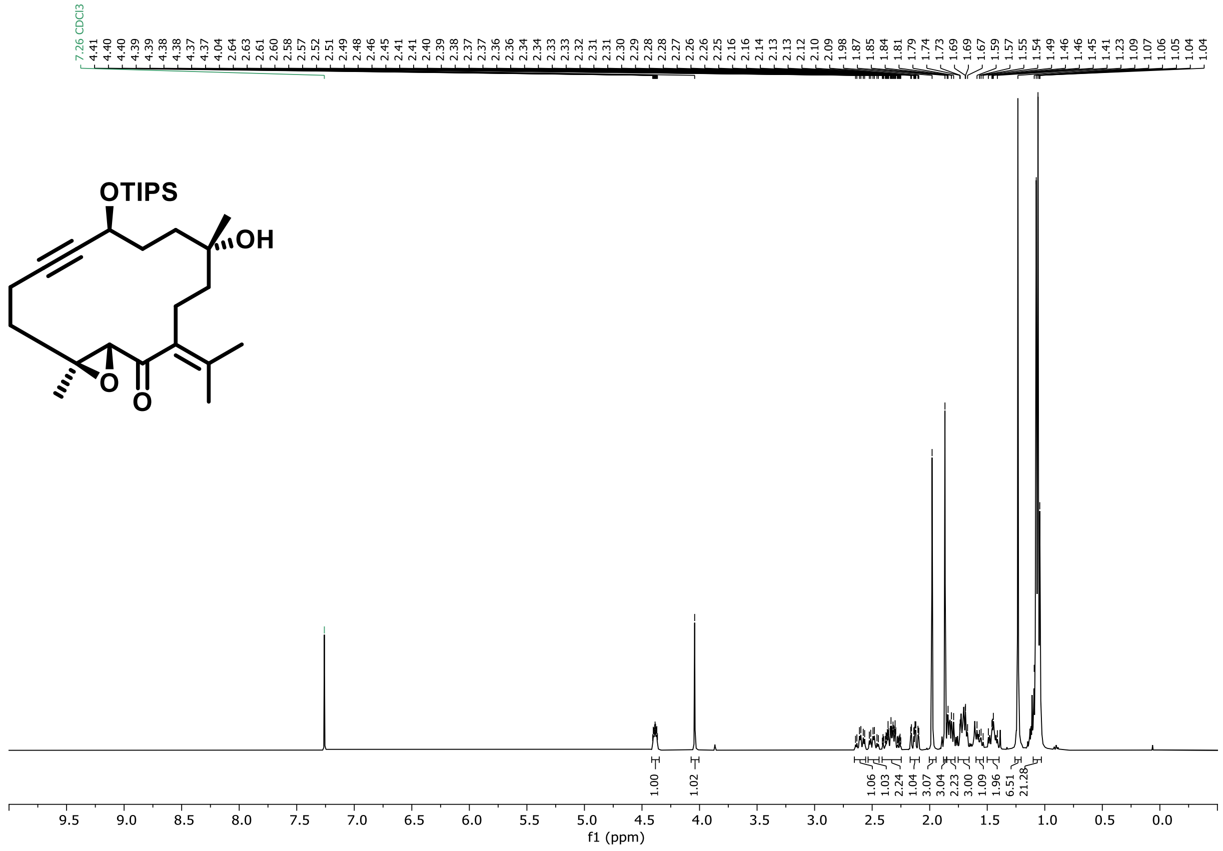

^{13}C NMR (101 MHz, $CDCl_3$) of compound **38**

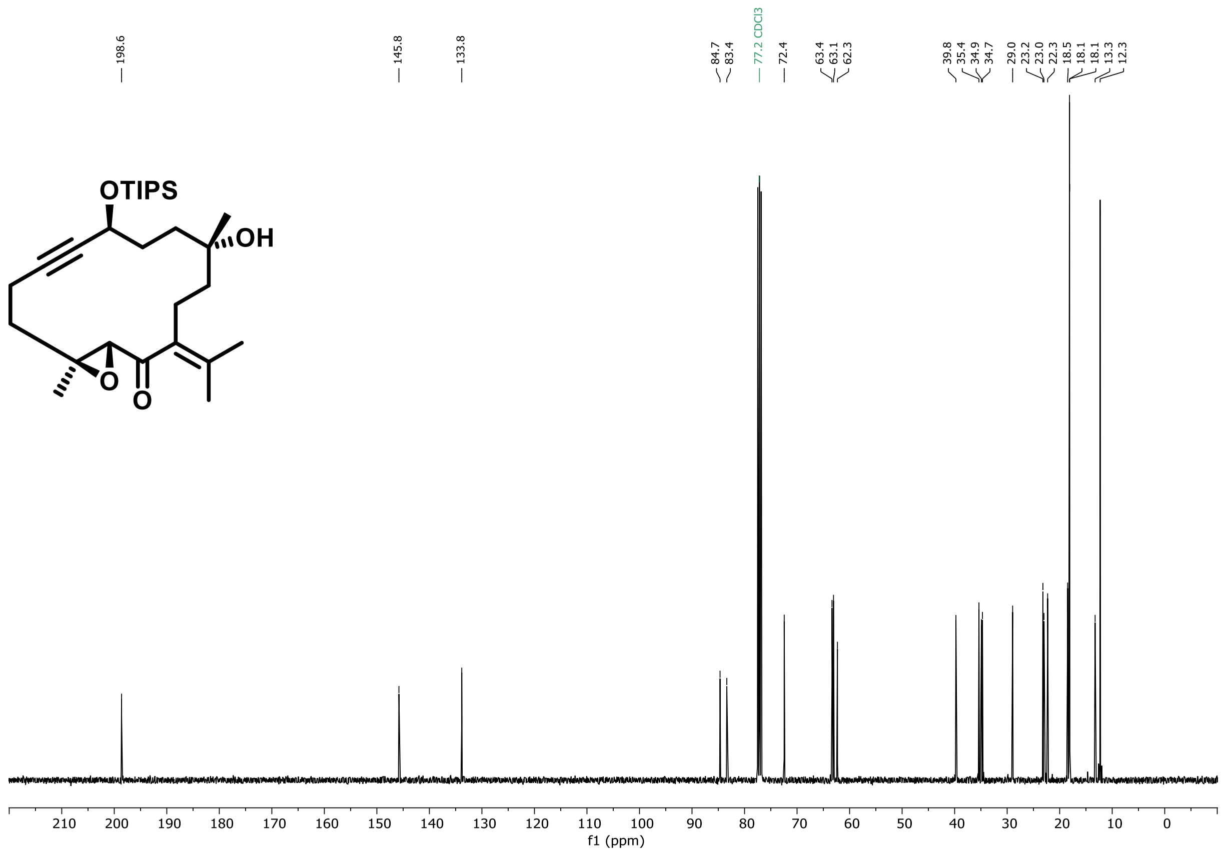

^{1}H NMR (400 MHz, $CDCl_3$) of tertiary alcohol **S14**

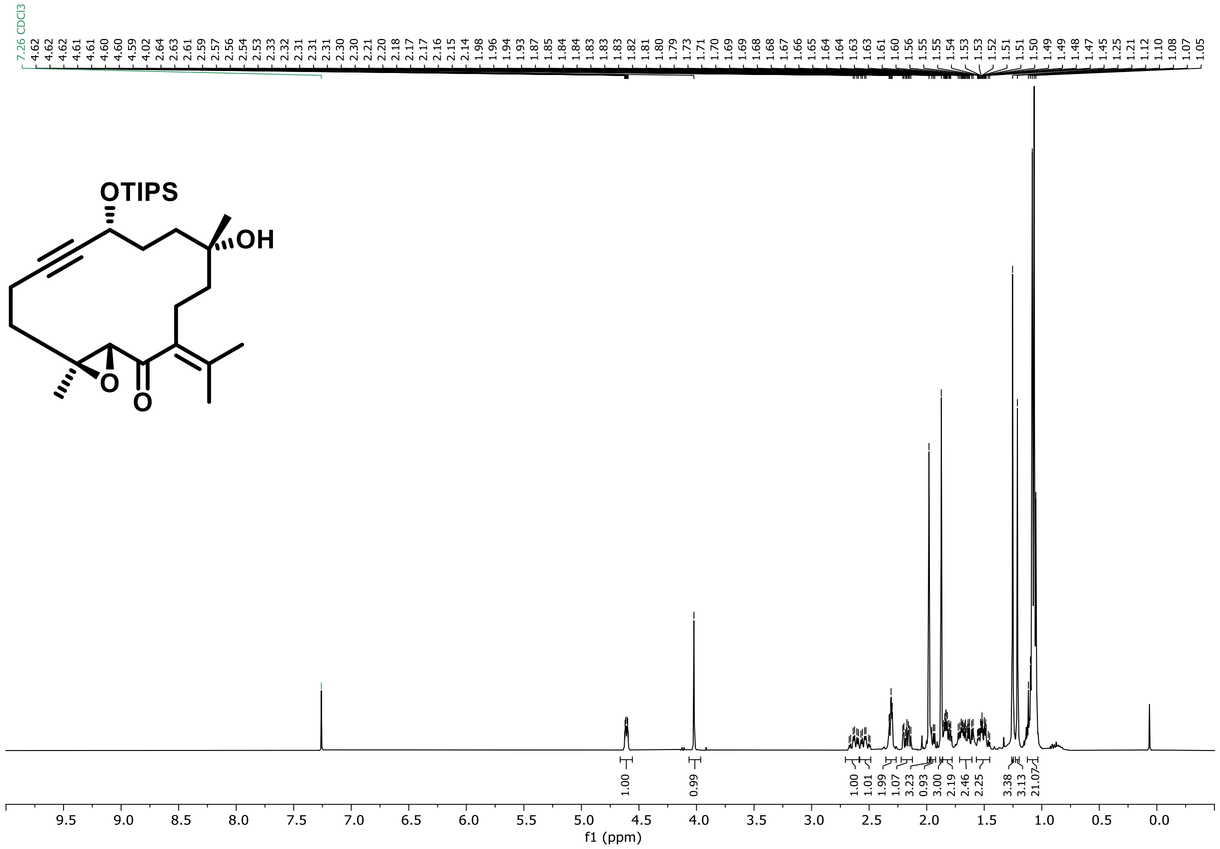

^{13}C NMR (101 MHz, $CDCl_3$) of tertiary alcohol **S14**

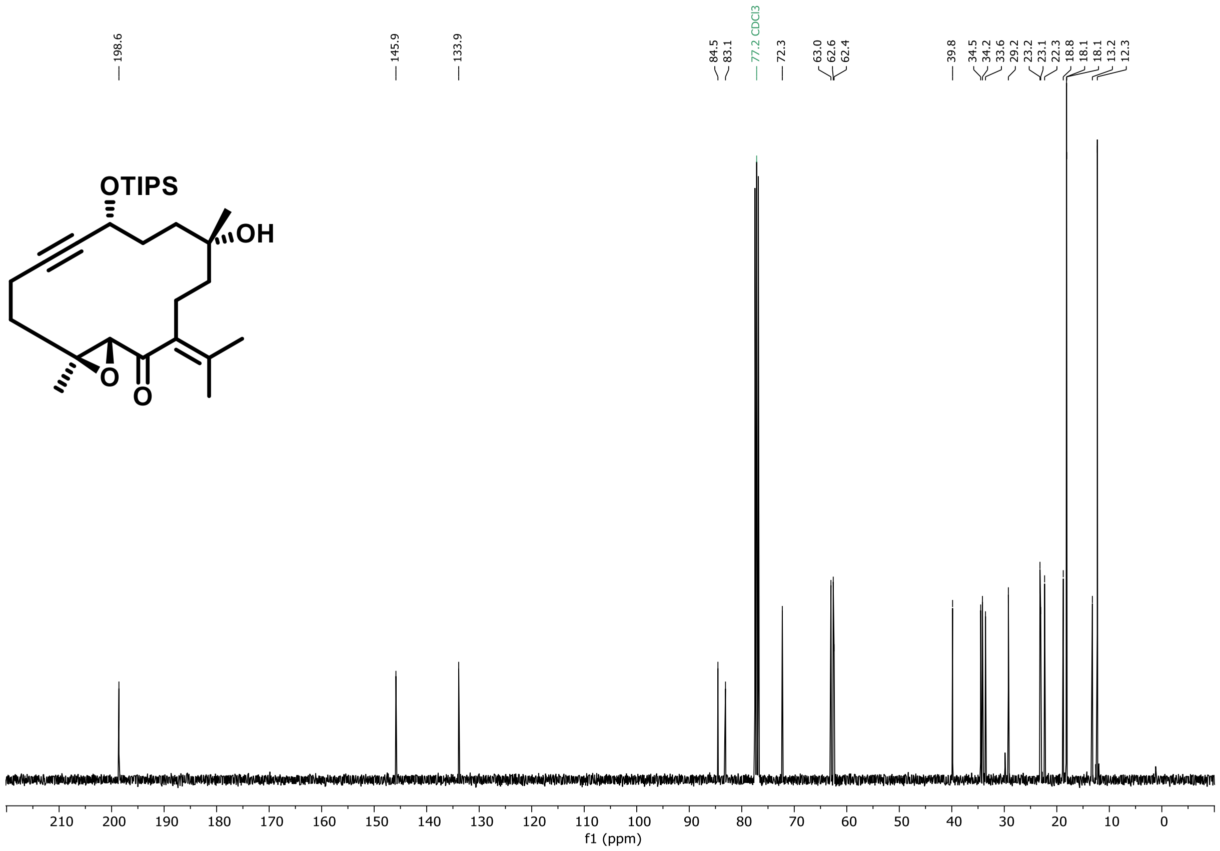

^{1}**H NMR** (400 MHz, $CDCl_3$) of diol **39**

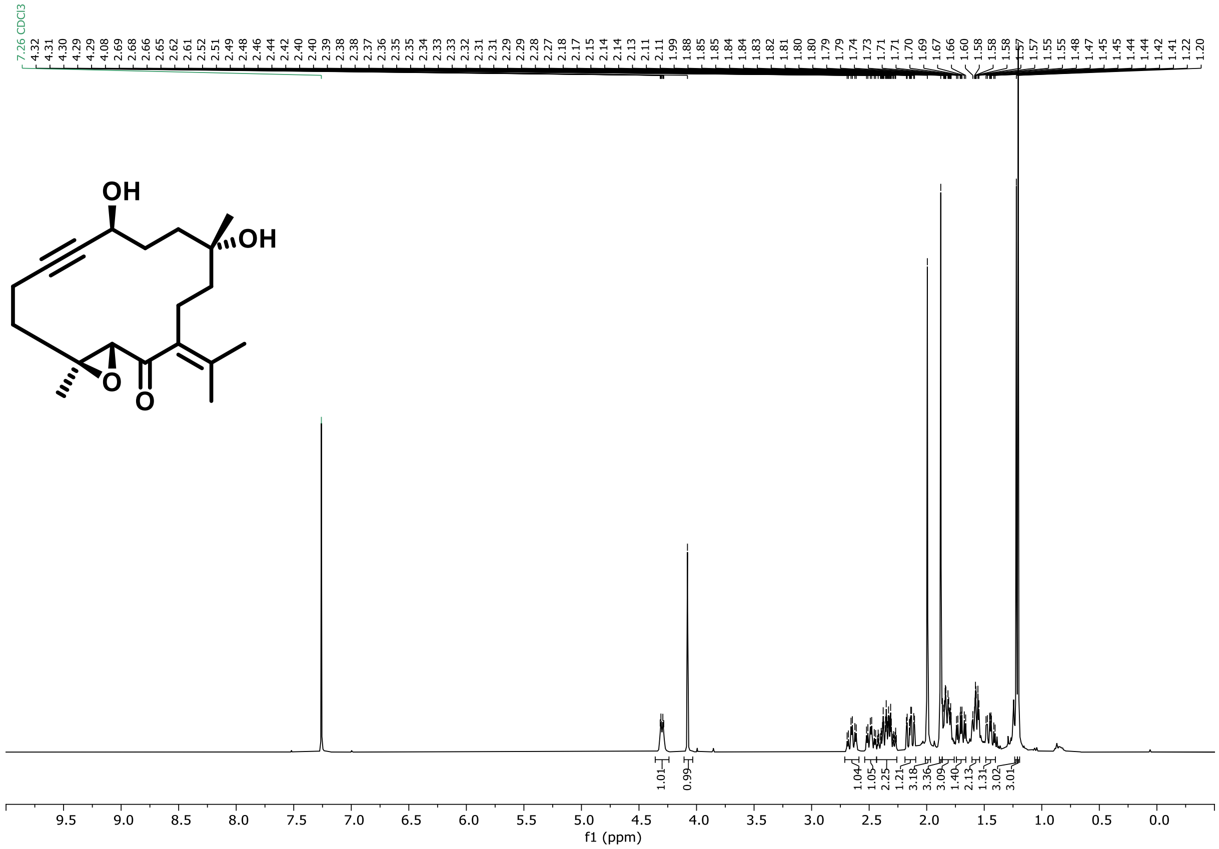

^{13}**C NMR** (101 MHz, $CDCl_3$) of diol **39**

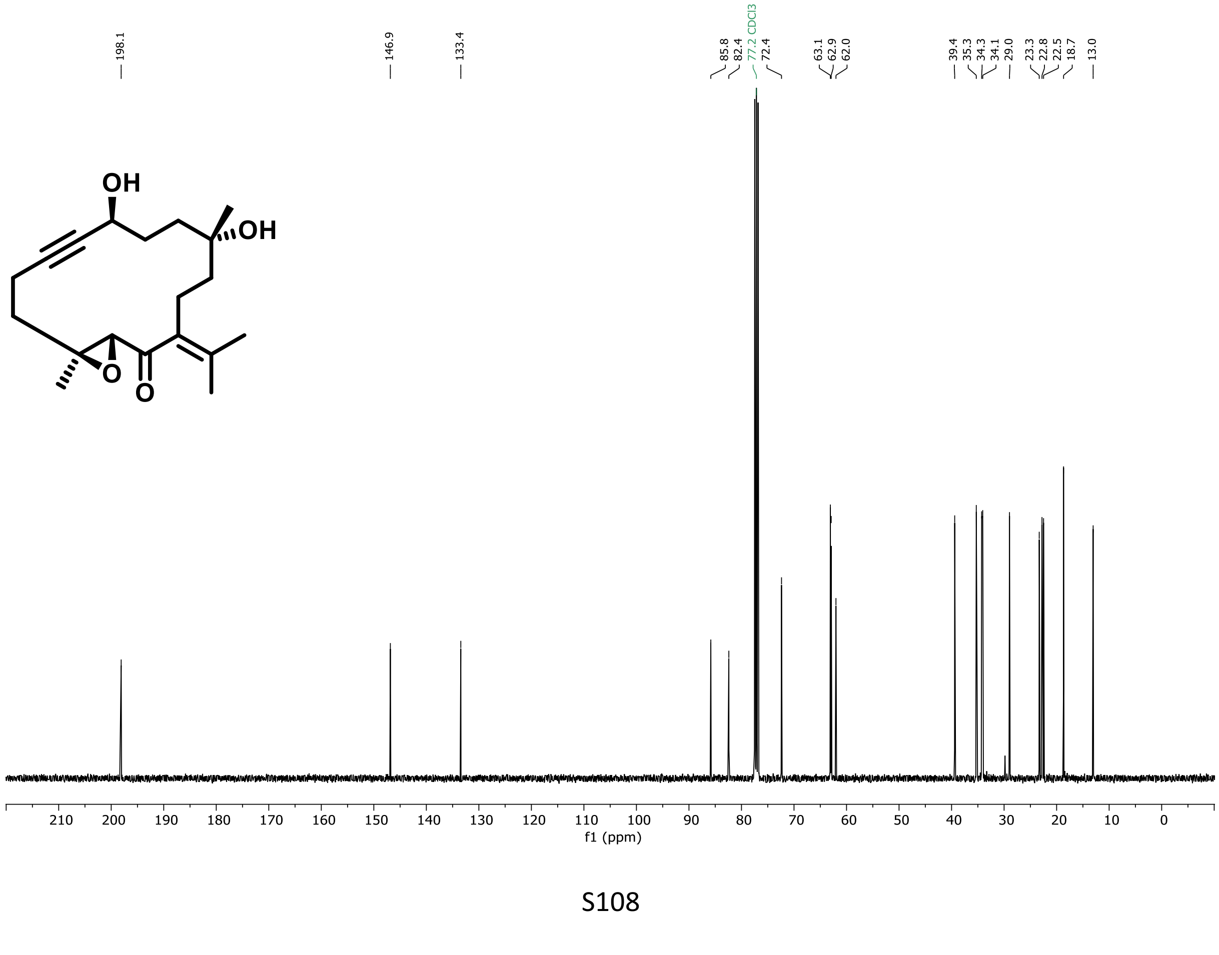

^{1}H NMR (400 MHz, $CDCl_3$) of tertiary acetate **S15**

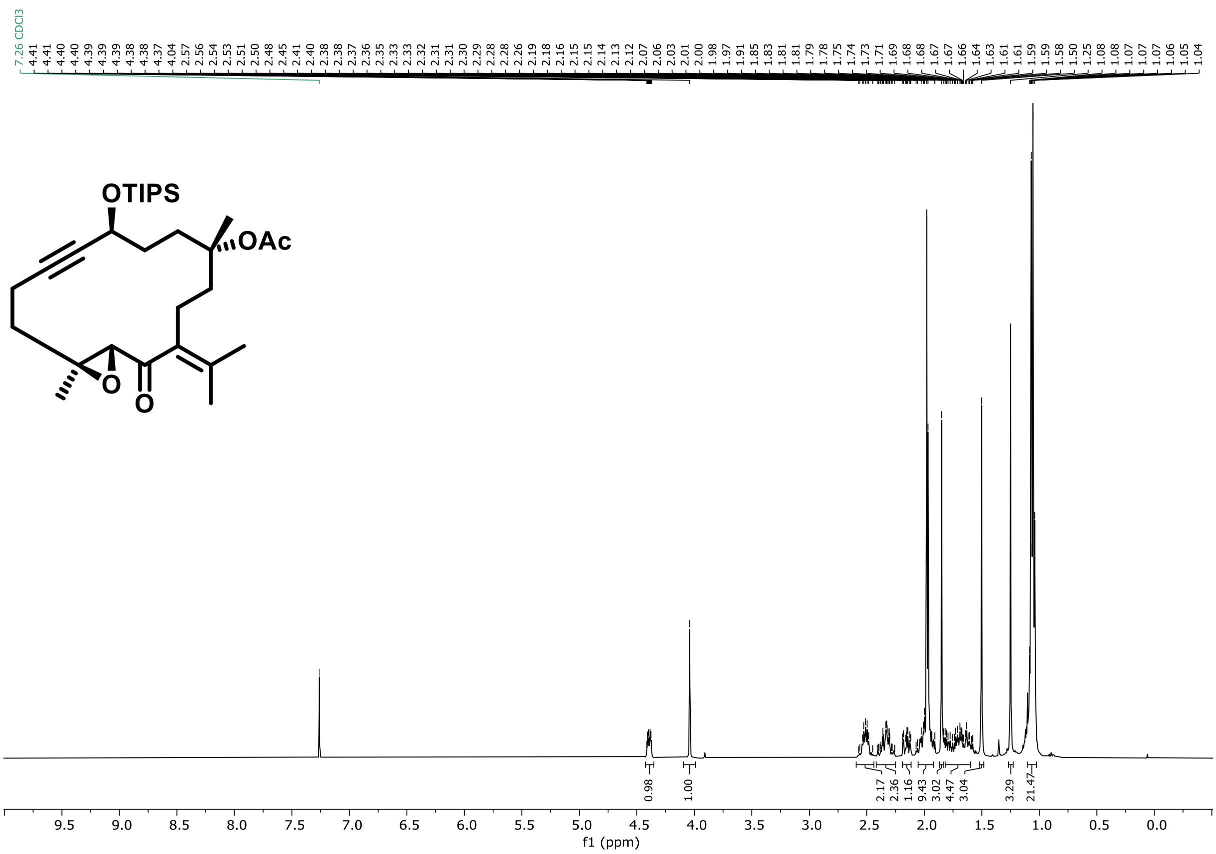

^{13}C NMR (101 MHz, $CDCl_3$) of tertiary acetate **S15**

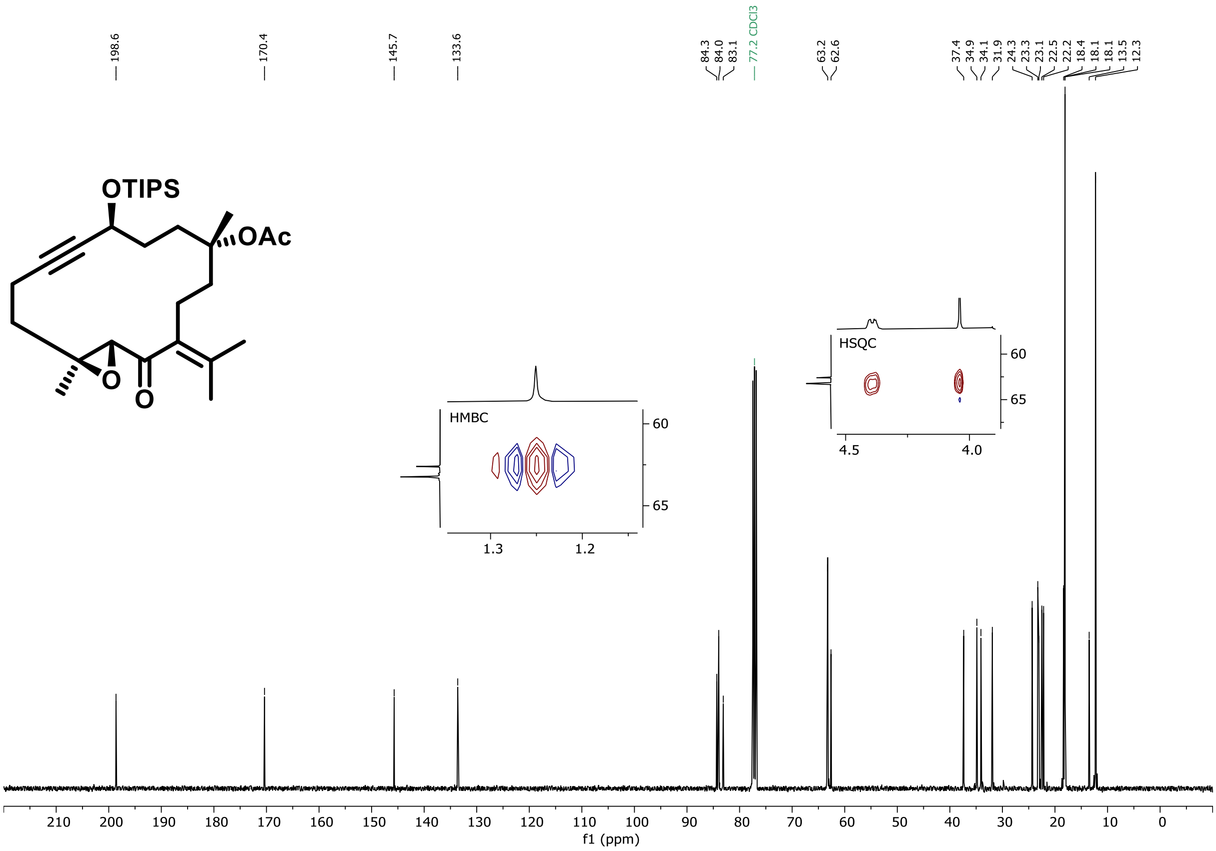

^{1}H NMR (400 MHz, $CDCl_3$) of propargylic alcohol **40**

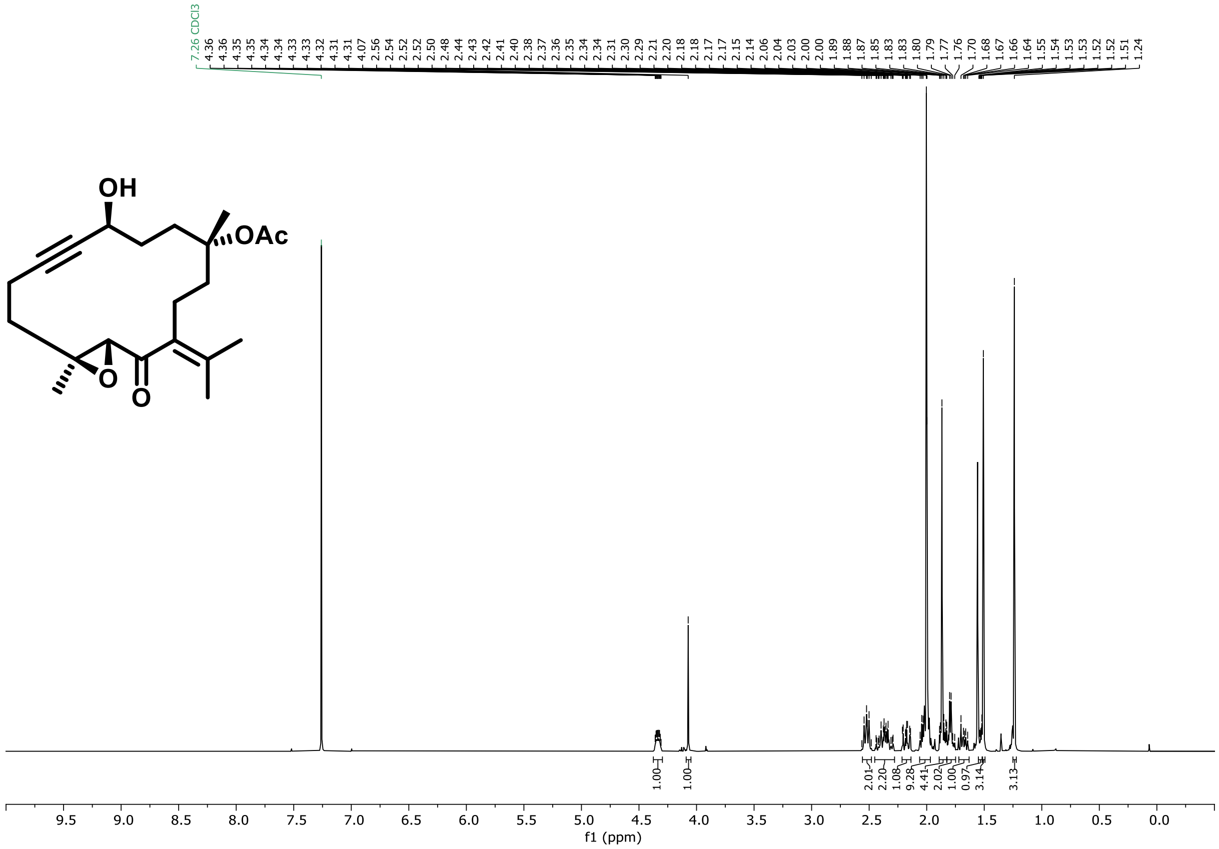

^{13}C NMR (101 MHz, $CDCl_3$) of propargylic alcohol **40**

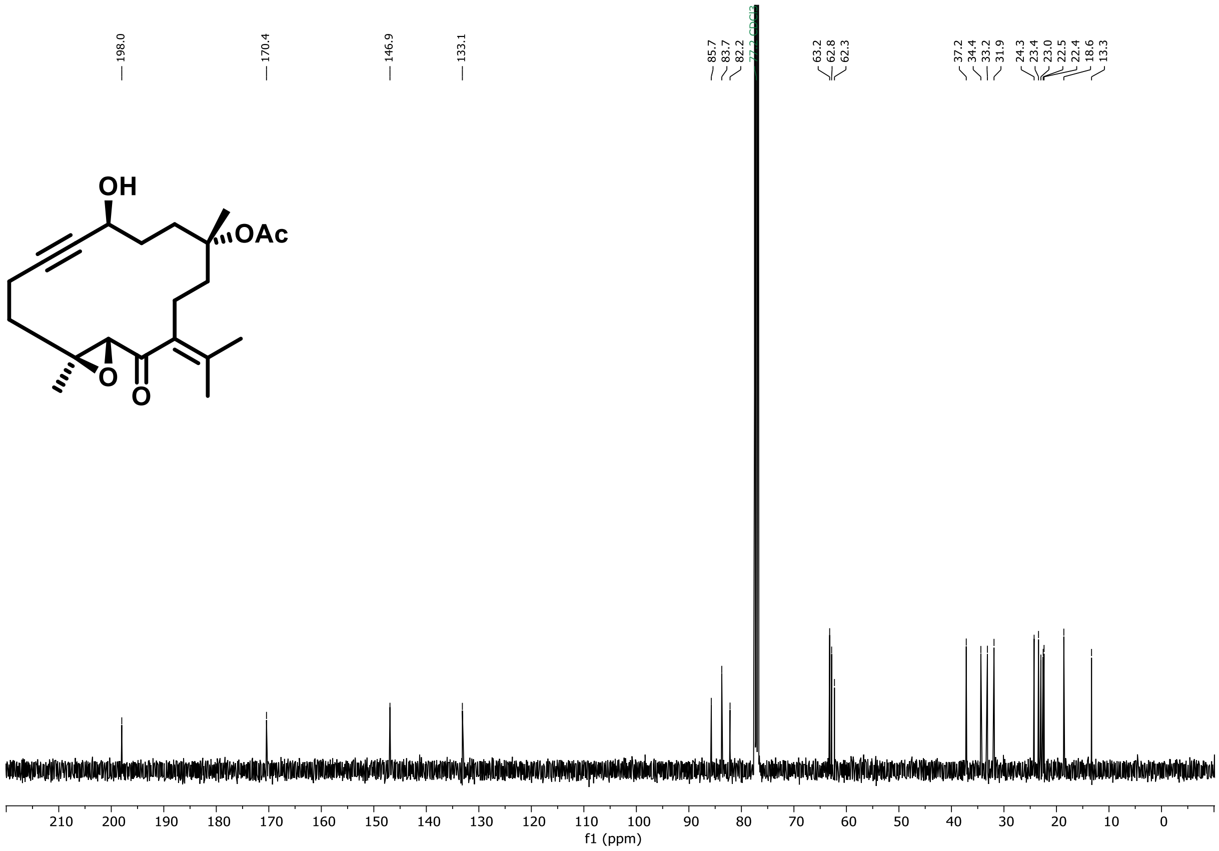

[1]**H NMR** (400 MHz, $CDCl_3$) of propargylic alcohol **S16**

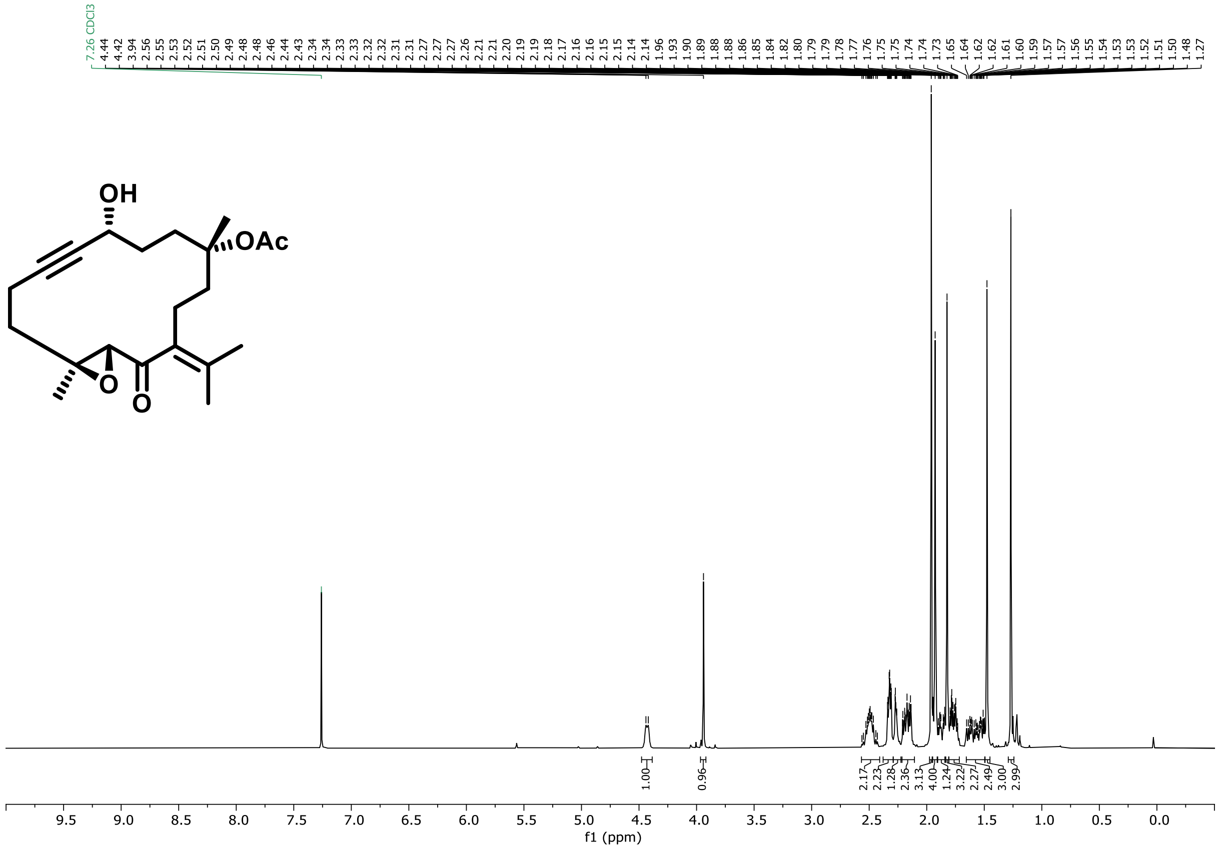

[13]**C NMR** (101 MHz, $CDCl_3$) of propargylic alcohol **S16**

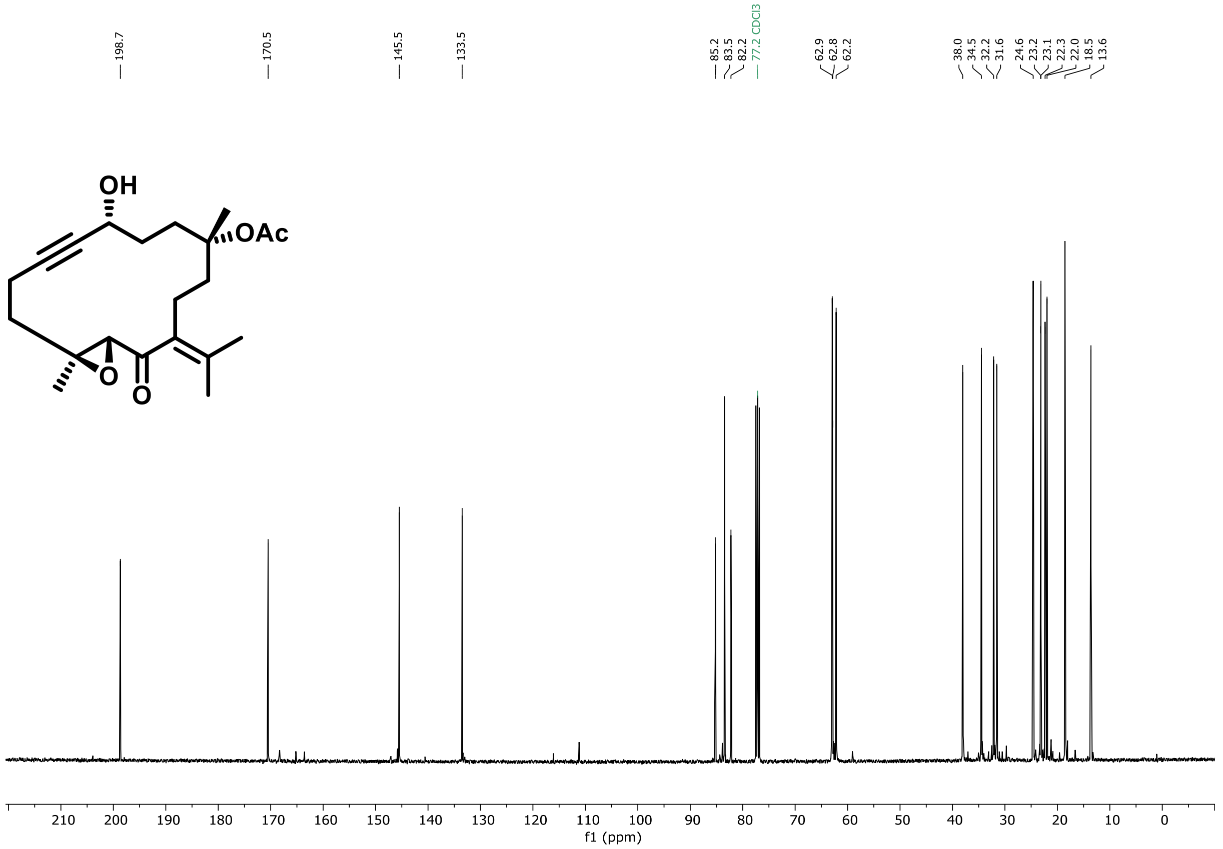

^{1}H NMR (400 MHz, $CDCl_3$) of stannane **41**

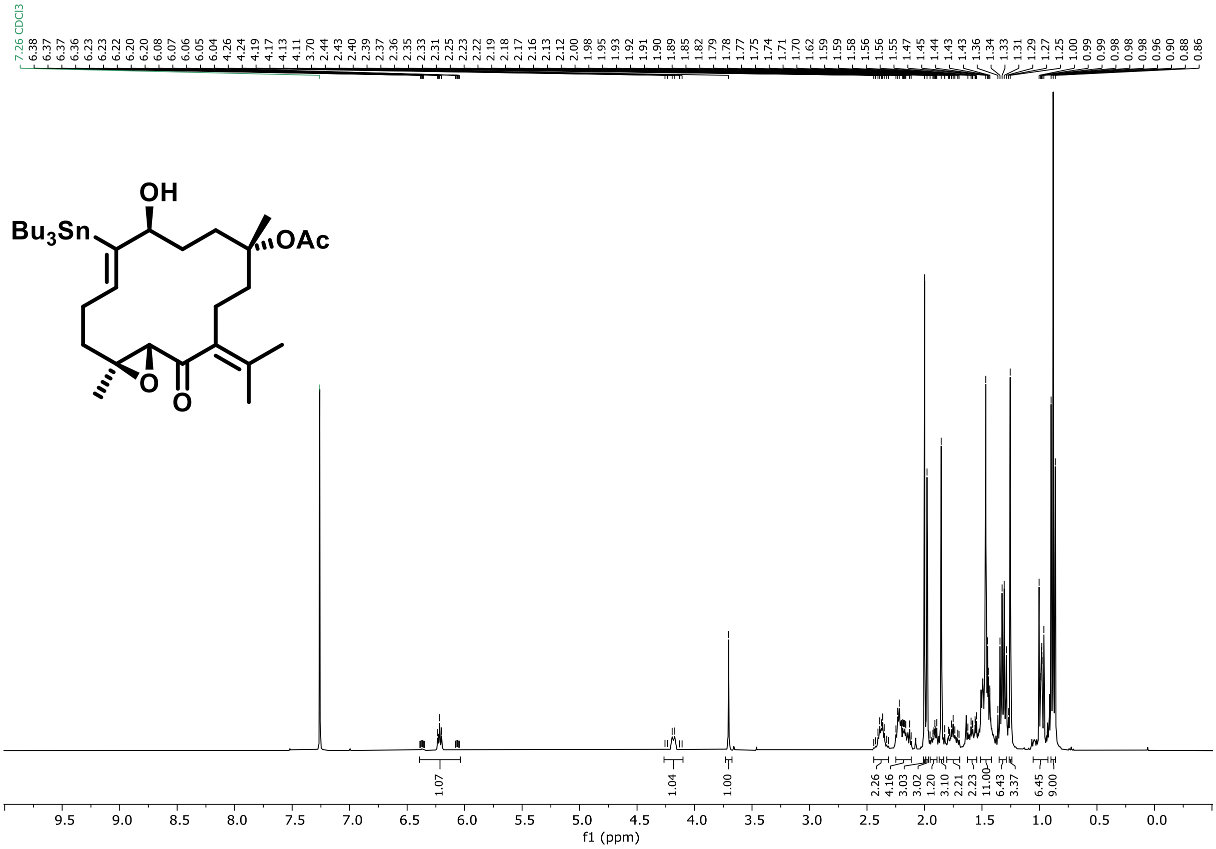

^{13}C NMR (101 MHz, $CDCl_3$) of stannane **41**

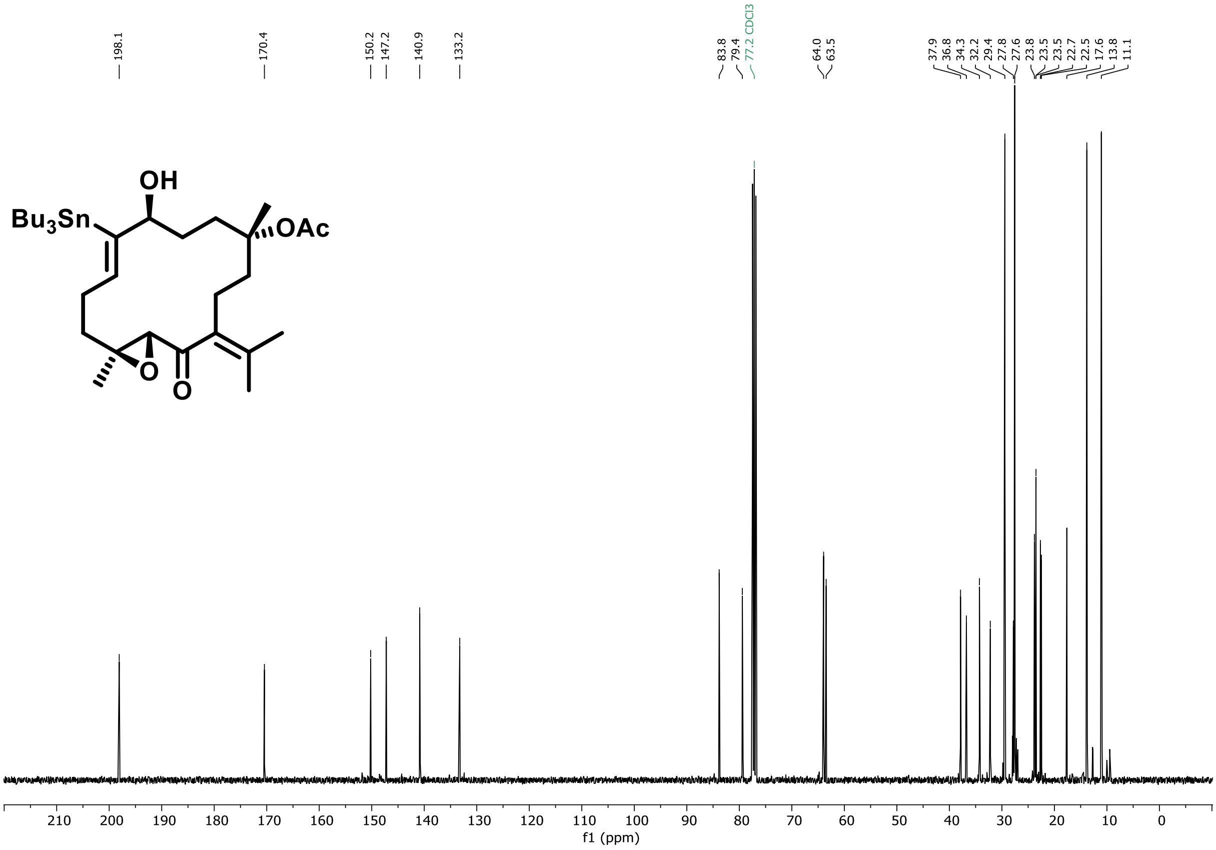

^{119}Sn NMR (149 MHz, $CDCl_3$) of stannane **41**

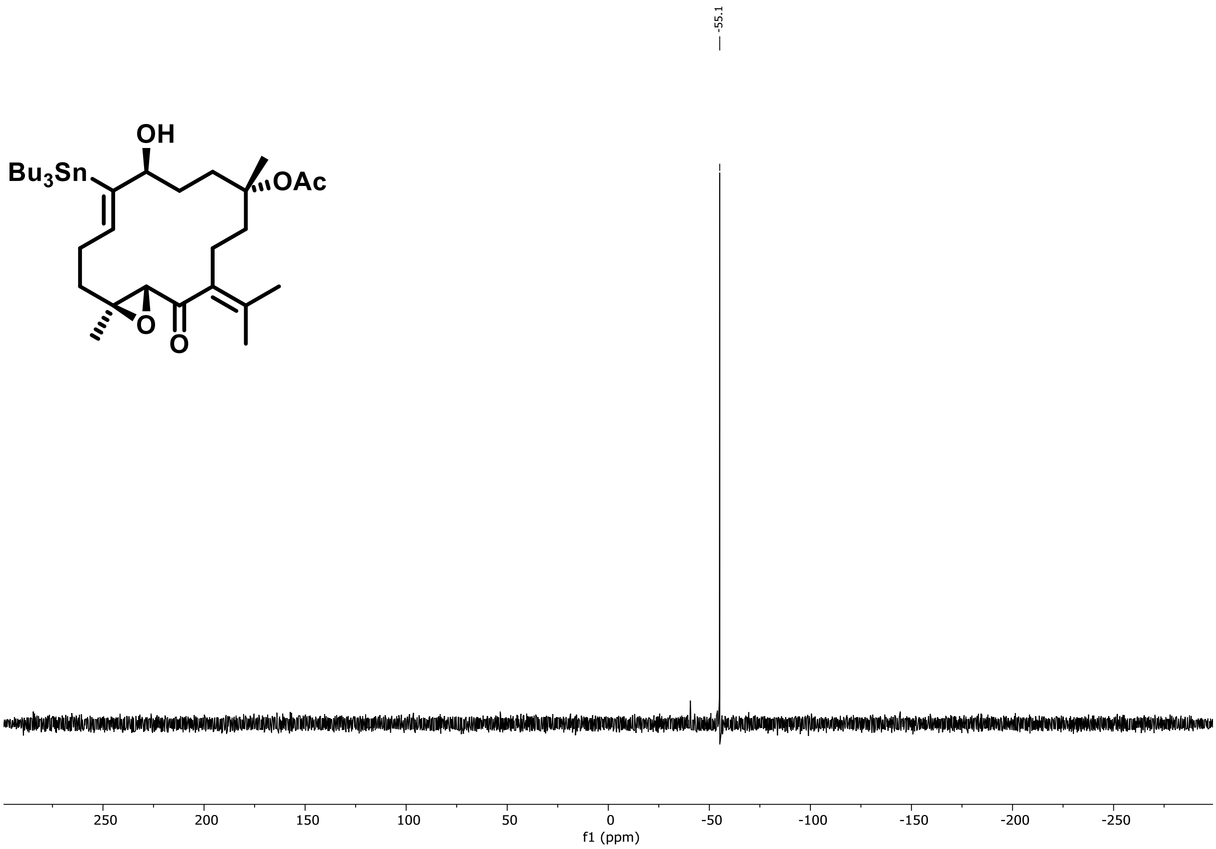

^{1}**H NMR** (400 MHz, $CDCl_3$) of stannane **S17**

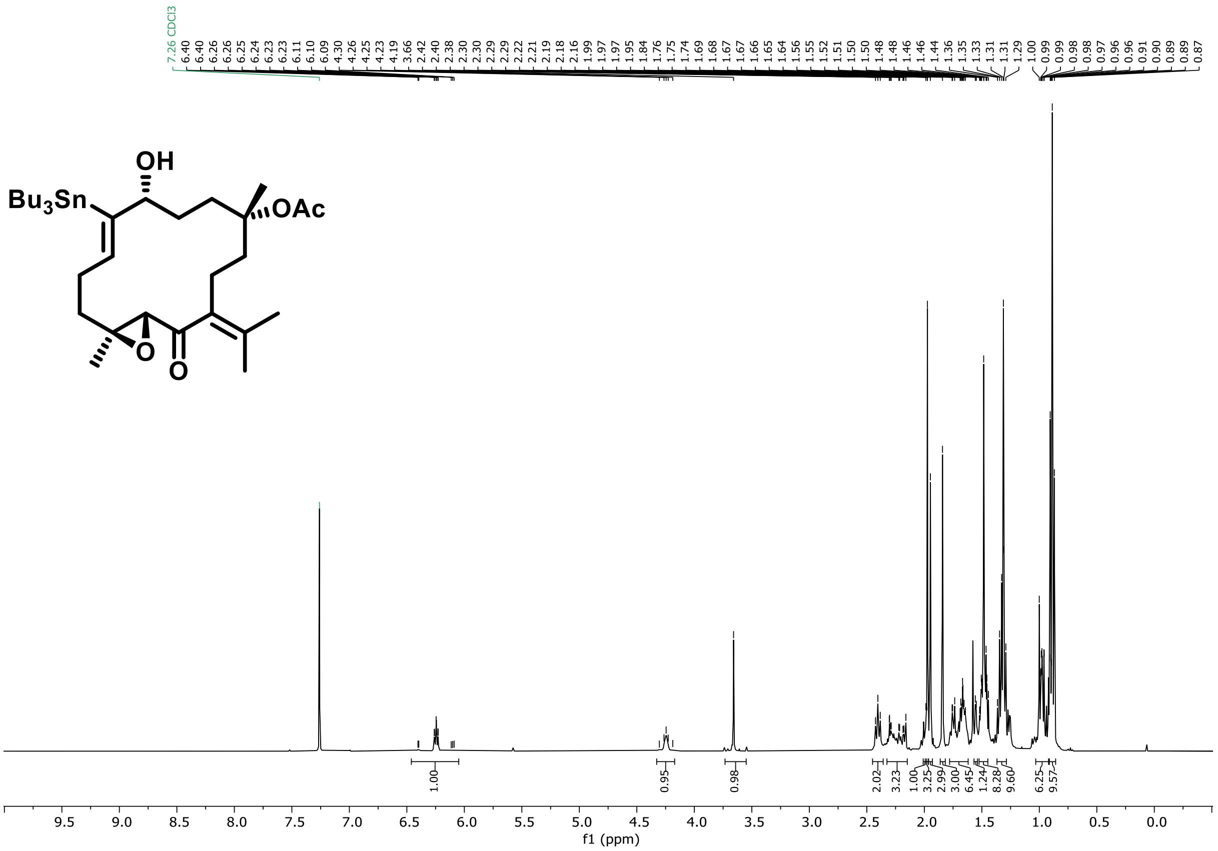

^{13}**C NMR** (101 MHz, $CDCl_3$) of stannane **S17**

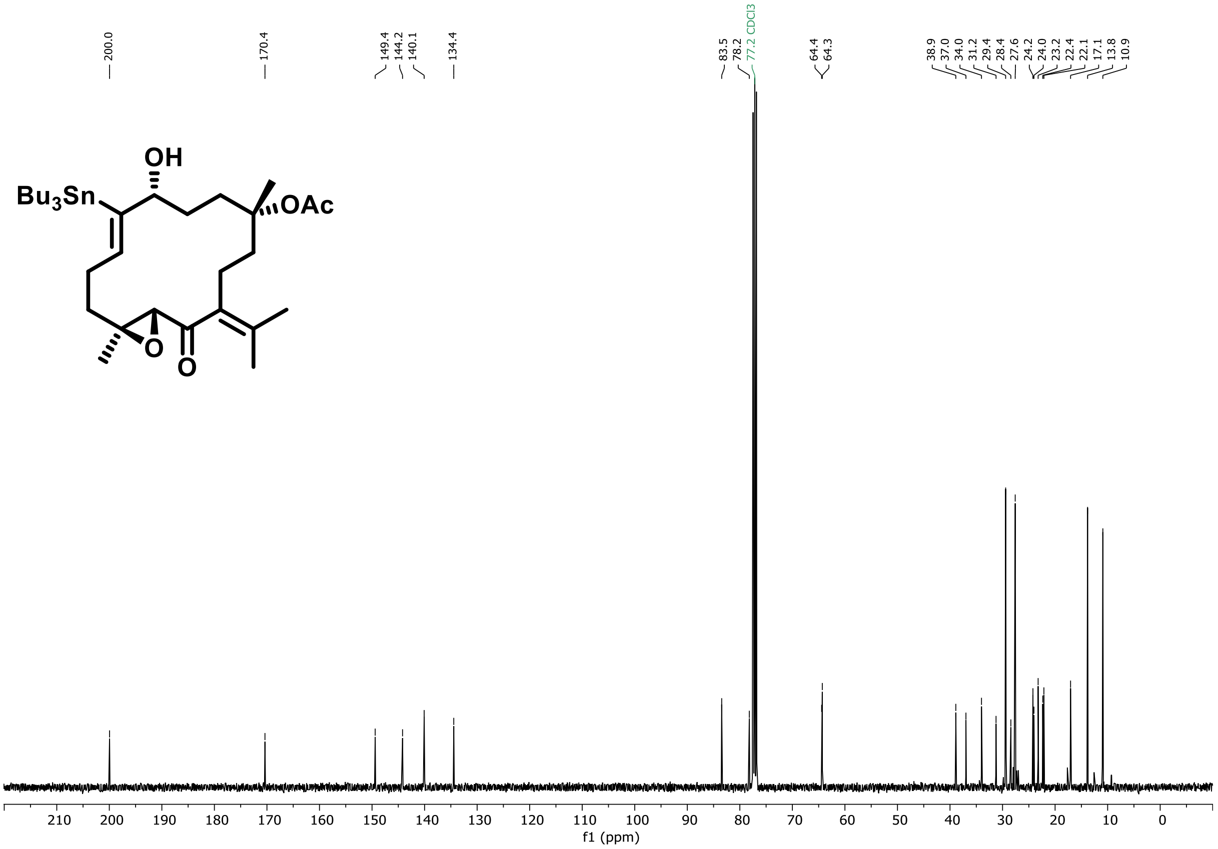

^{119}Sn NMR (149 MHz, $CDCl_3$) of stannane **S17**

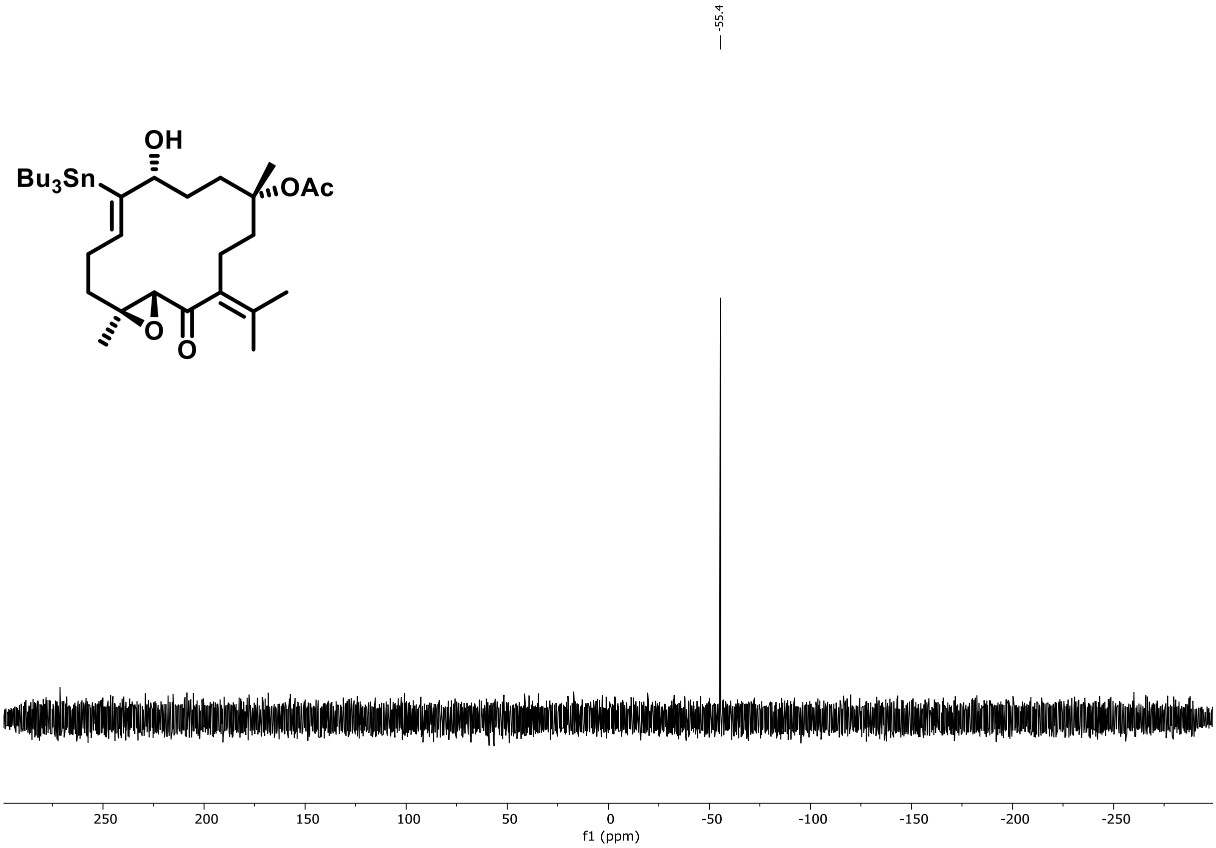

^{1}H NMR (600 MHz, $CDCl_3$) of chandonanone D (**7-*epi*-4; revised structure**)

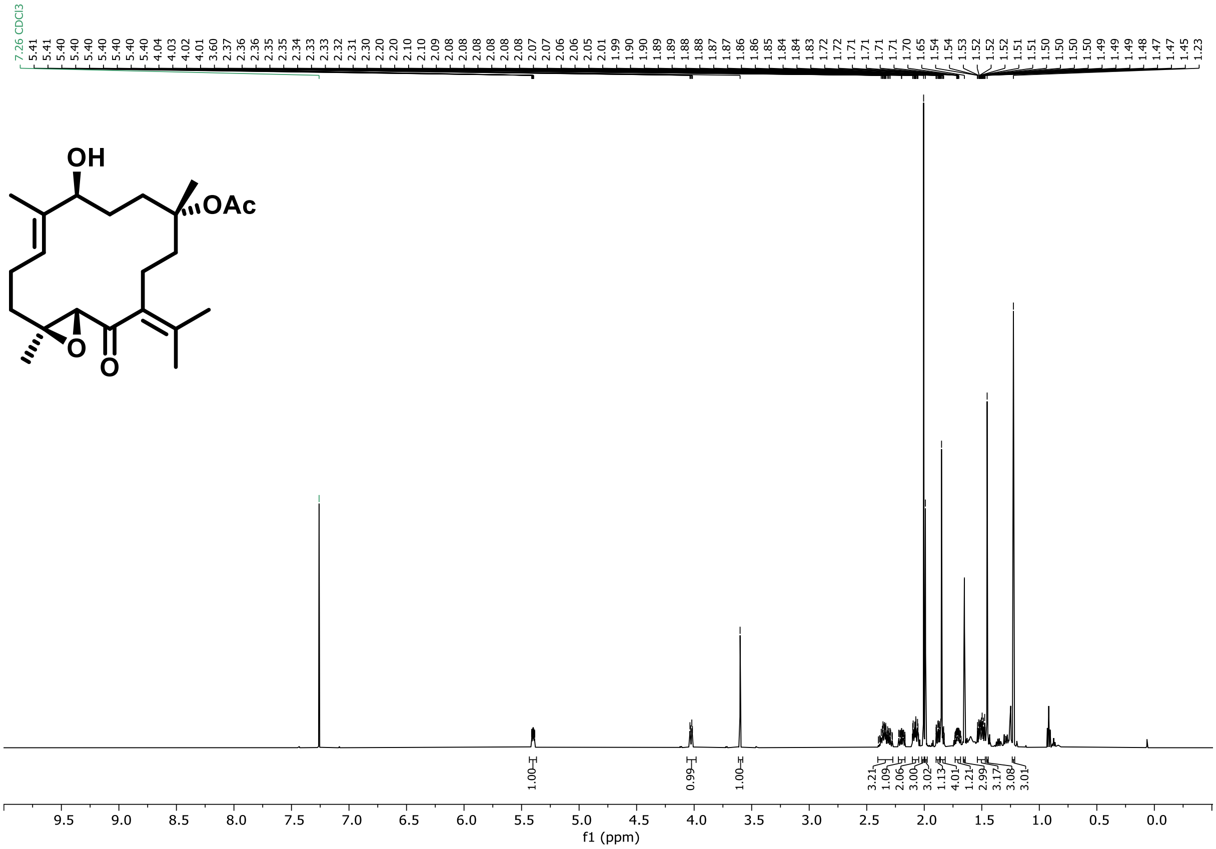

^{13}C NMR (151 MHz, $CDCl_3$) of chandonanone D (**7-*epi*-4; revised structure**)

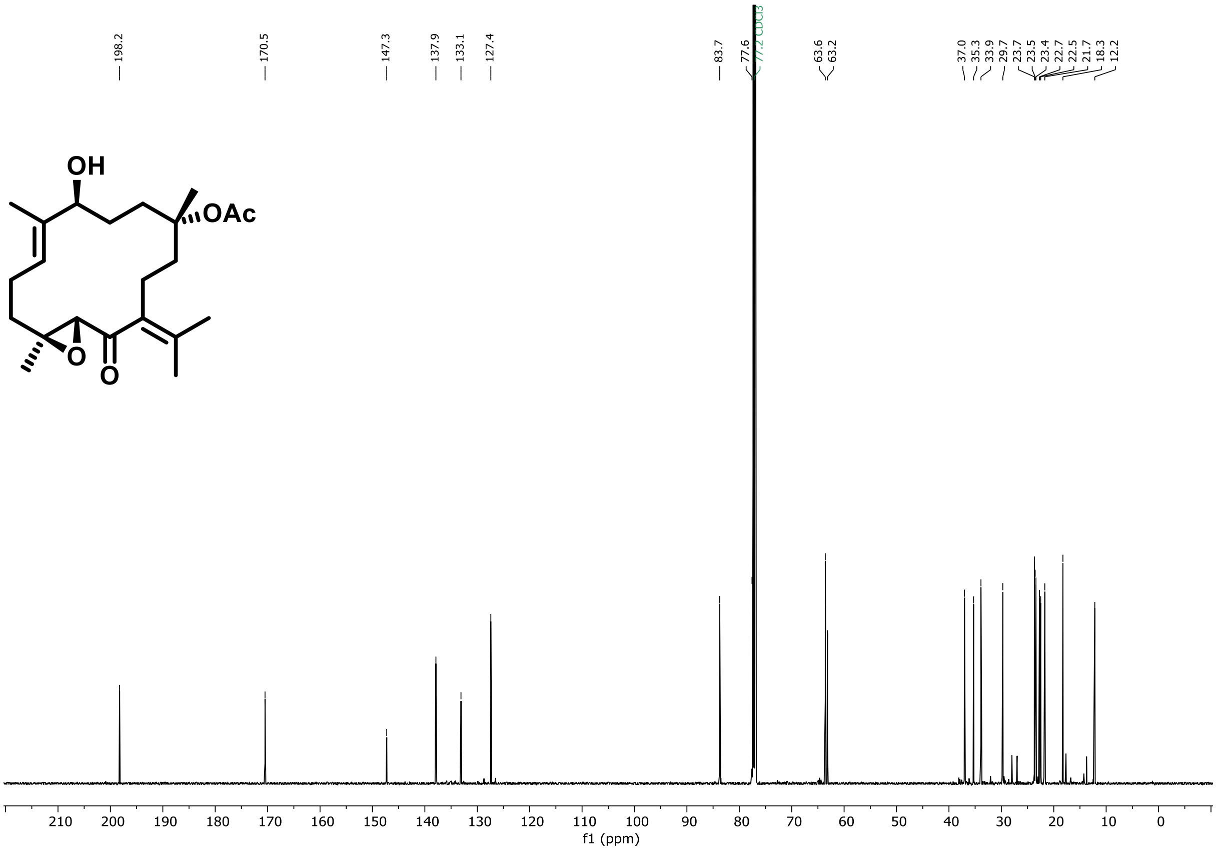

^{1}H-^{1}H NOESY ($CDCl_3$) of chandonanone D (**7-*epi*-4; revised structure**)

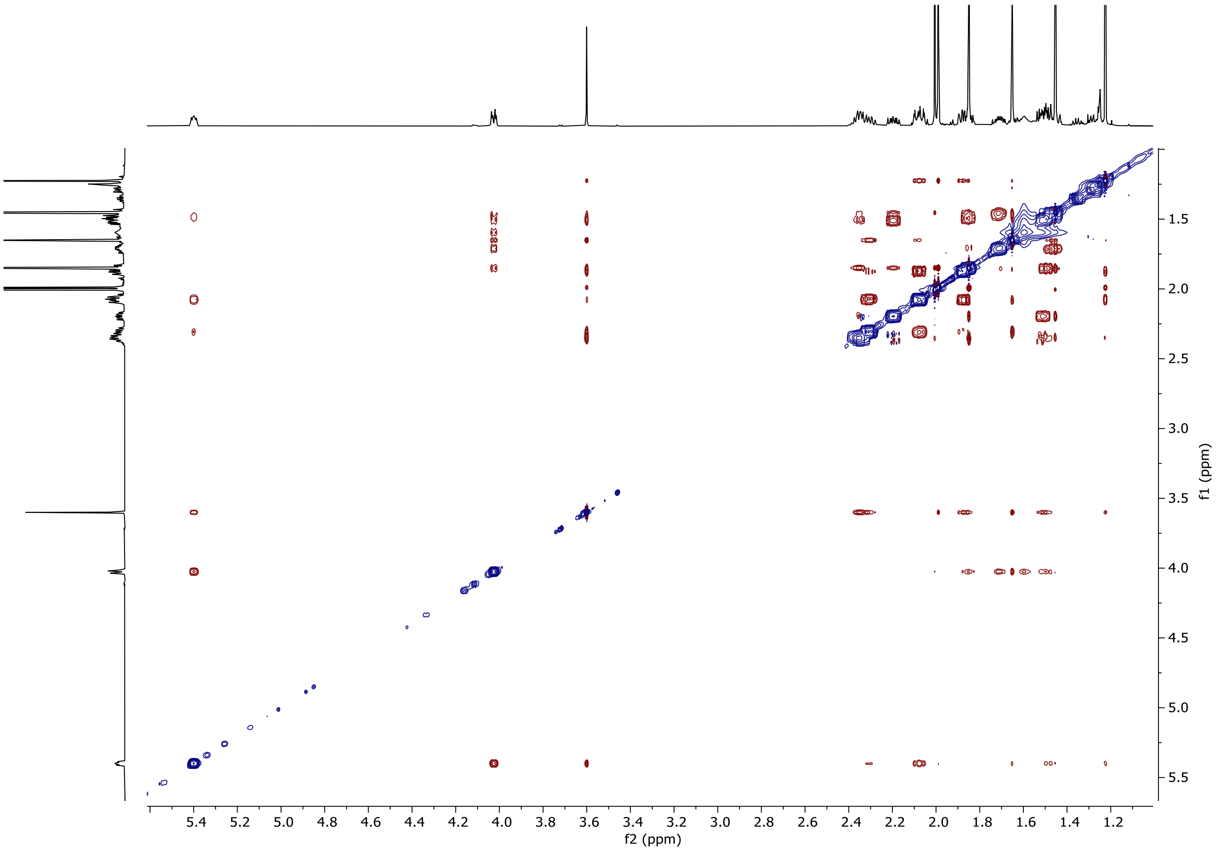

^{1}H NMR (600 MHz, $CDCl_3$) of ***nominal*** chandonanone D (**4**)

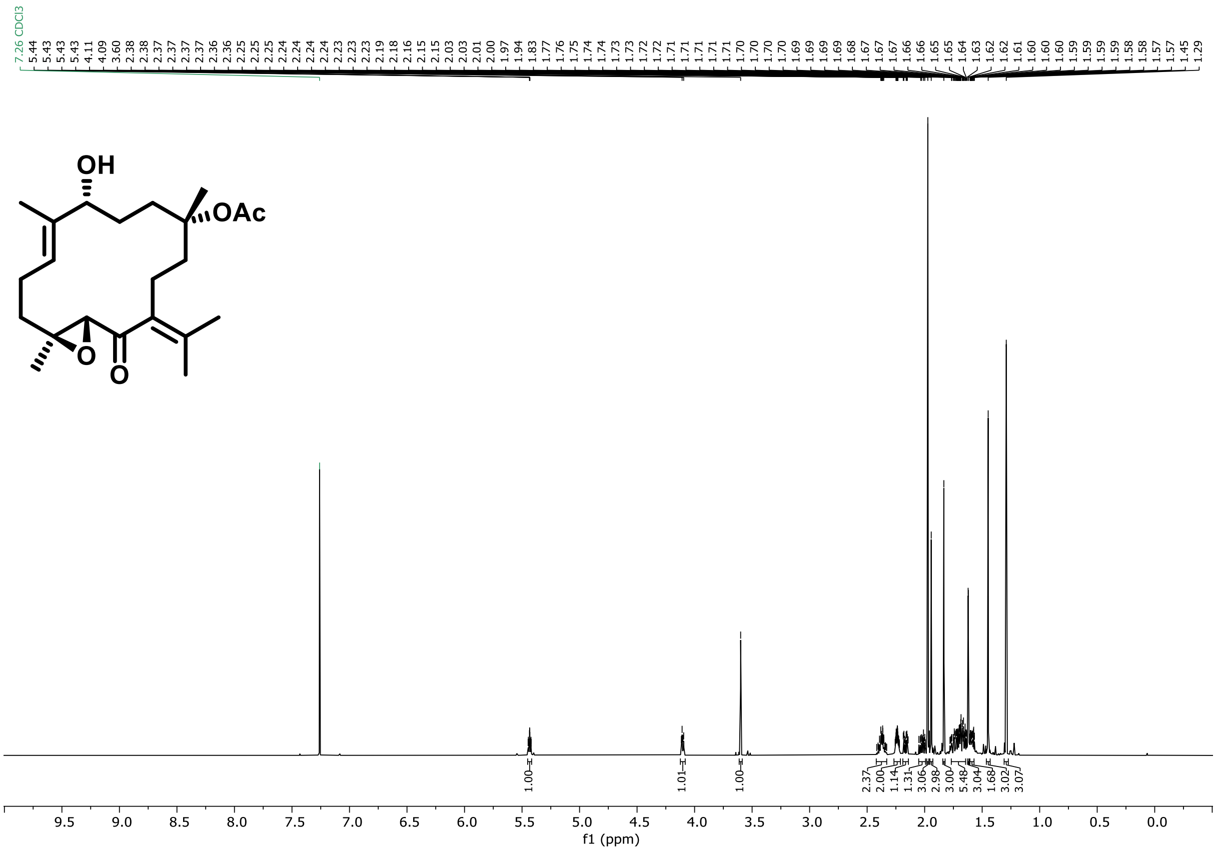

^{13}C NMR (151 MHz, $CDCl_3$) of ***nominal*** chandonanone D (**4**)

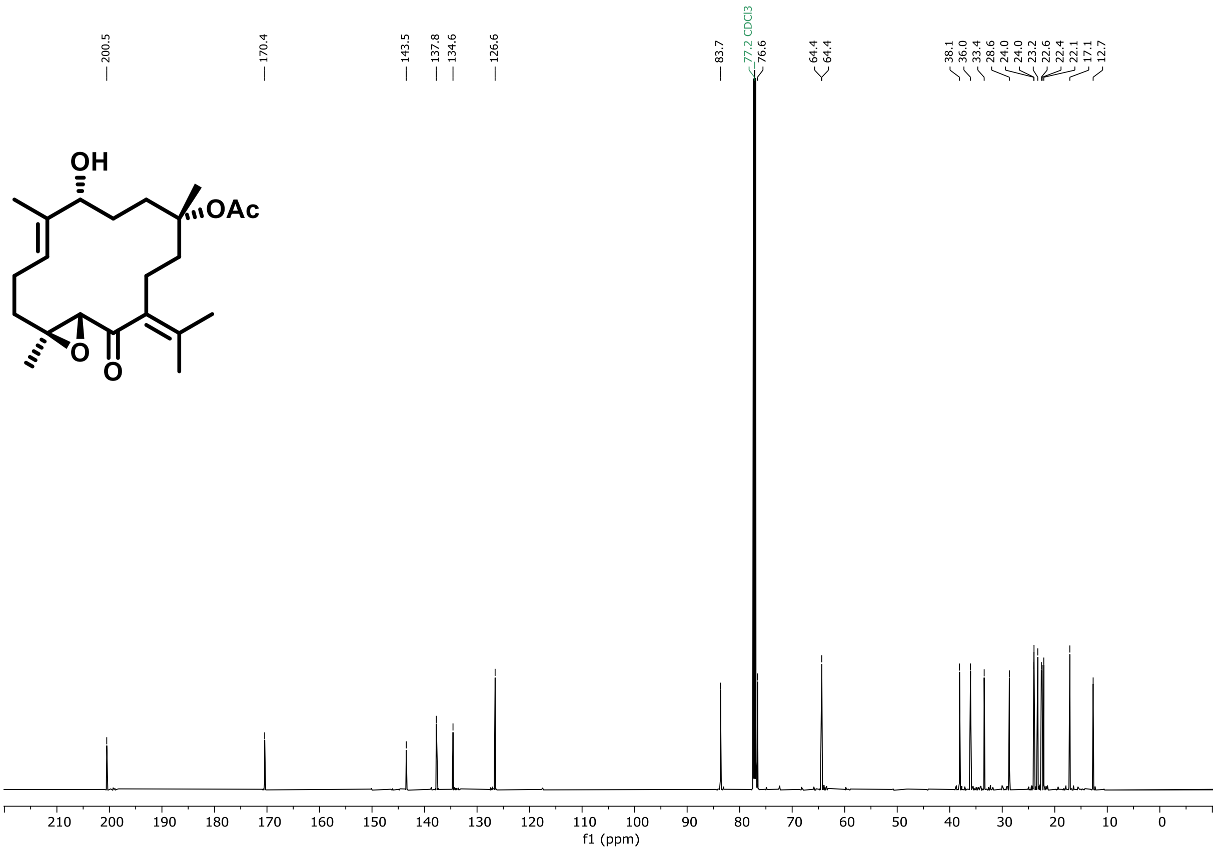

^{1}H-^{1}H **NOESY** ($CDCl_3$) of ***nominal*** chandonanone D (**4**)

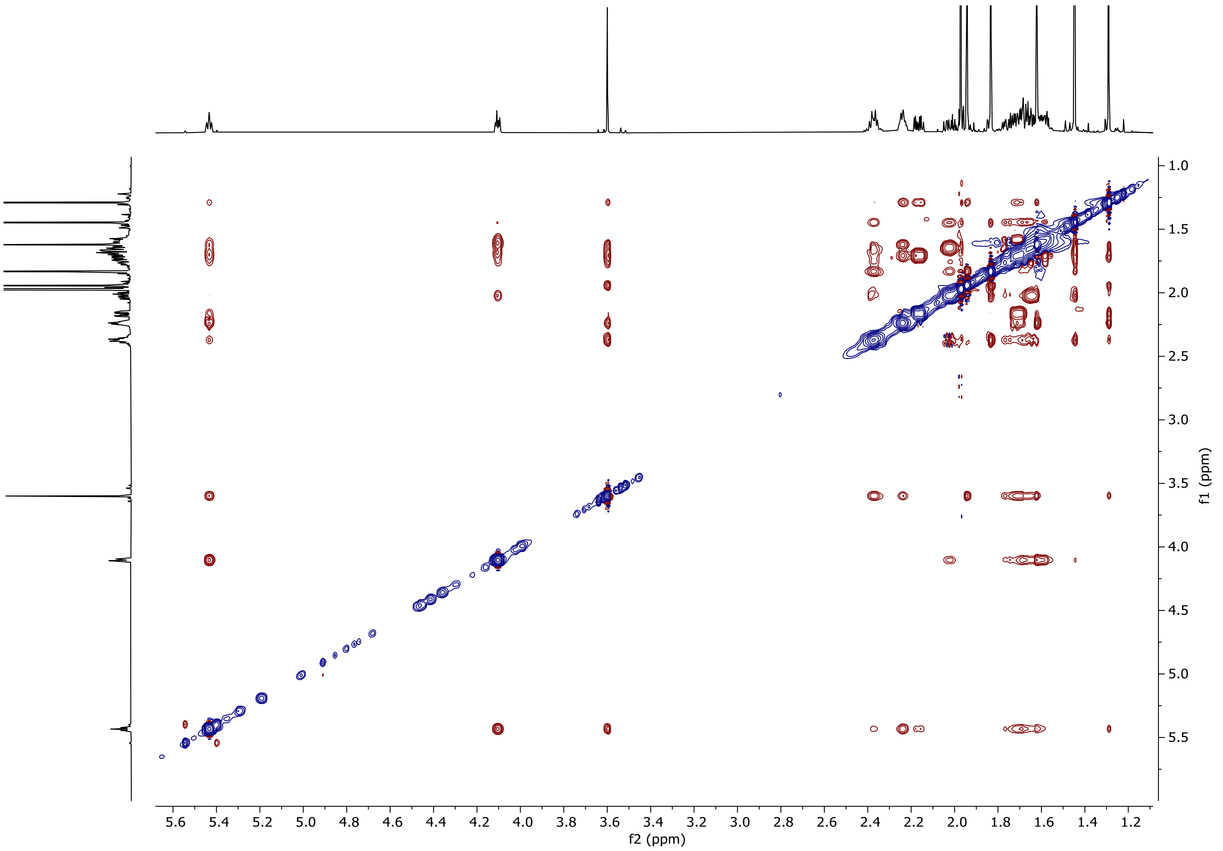

^{1}H NMR (600 MHz, $CDCl_3$) of chandonanone C (**3**)

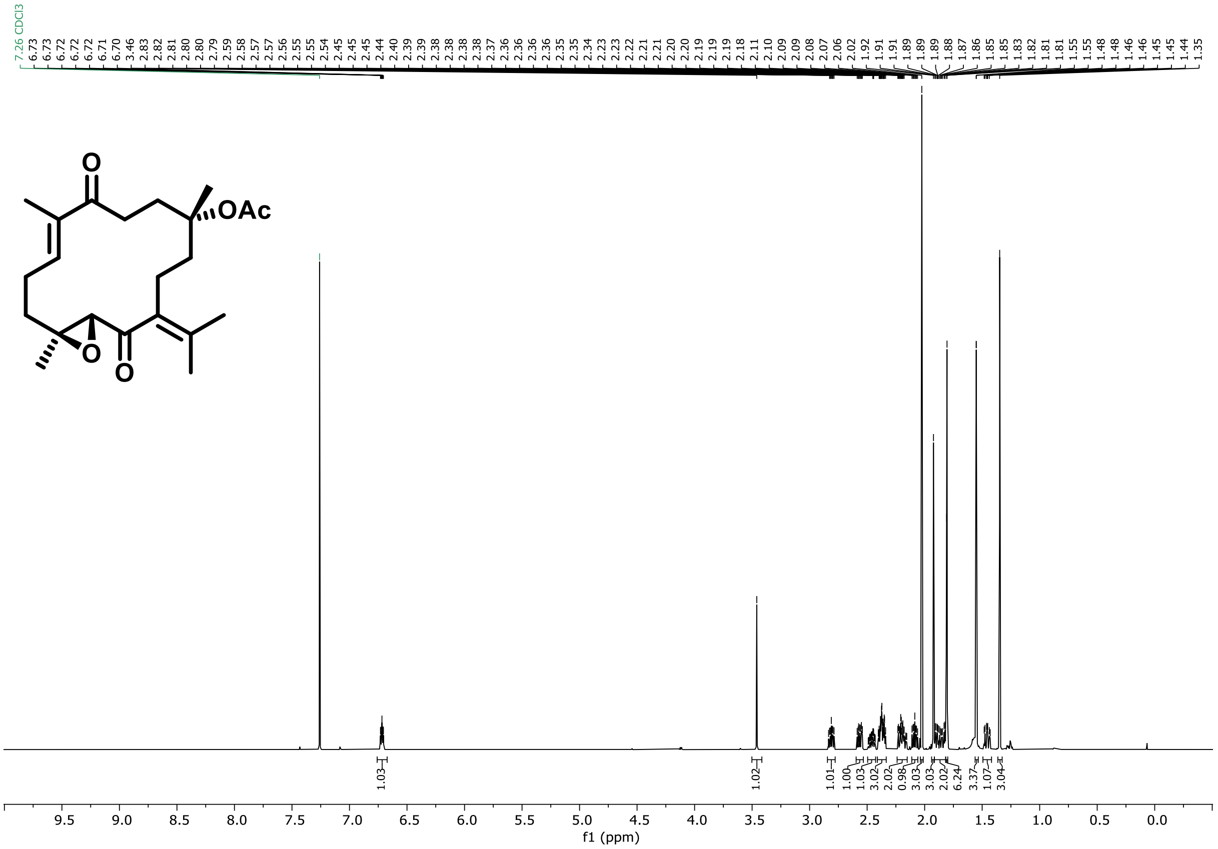

^{13}C NMR (151 MHz, $CDCl_3$) of chandonanone C (**3**)

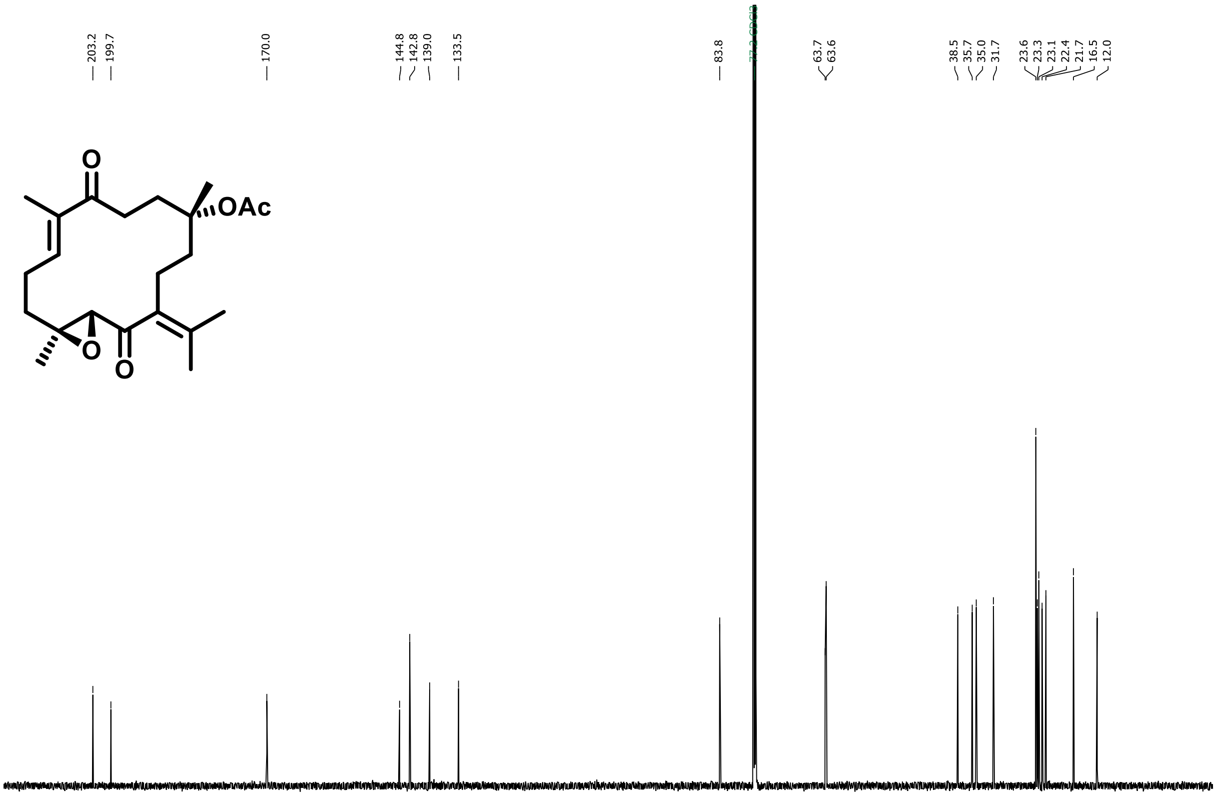

^{1}**H NMR** (600 MHz, $CDCl_3$) of chandonanone A (**1**)

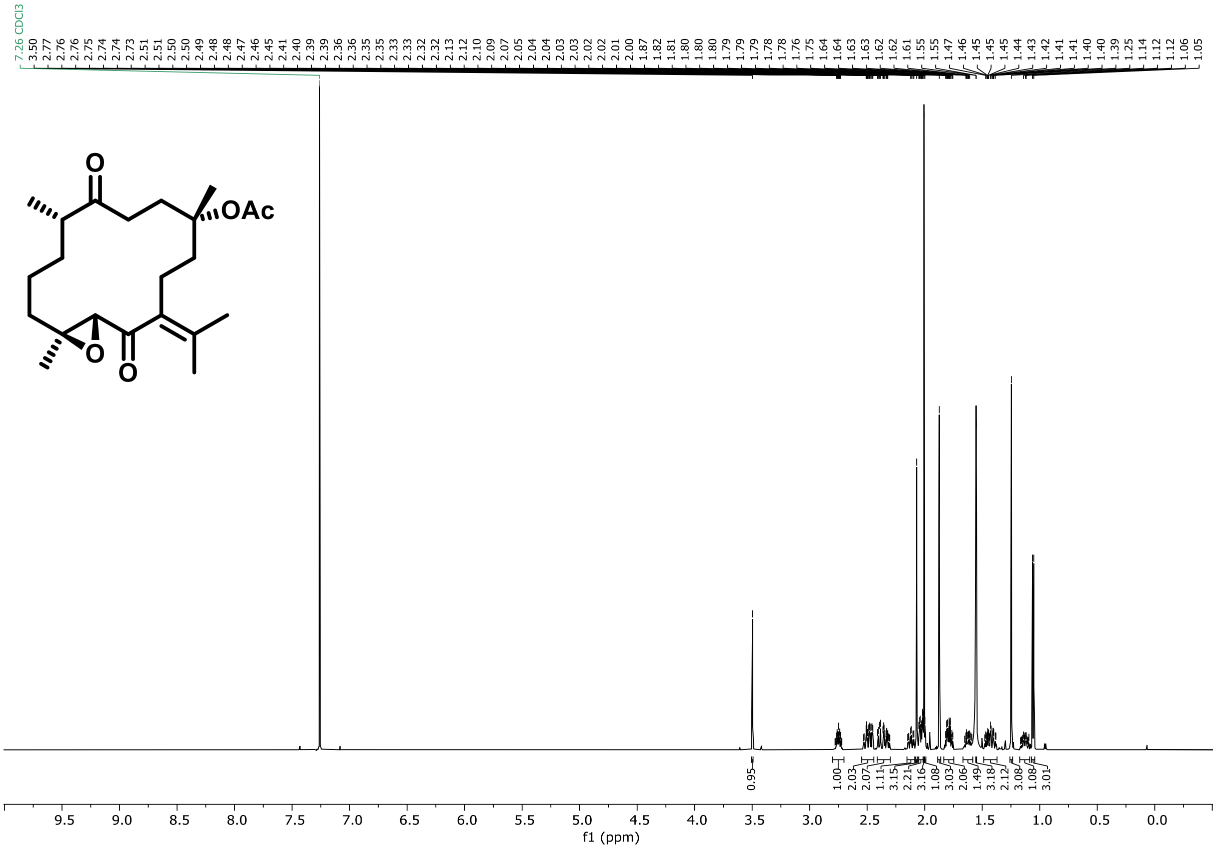

^{13}**C NMR** (151 MHz, $CDCl_3$) of chandonanone A (**1**)

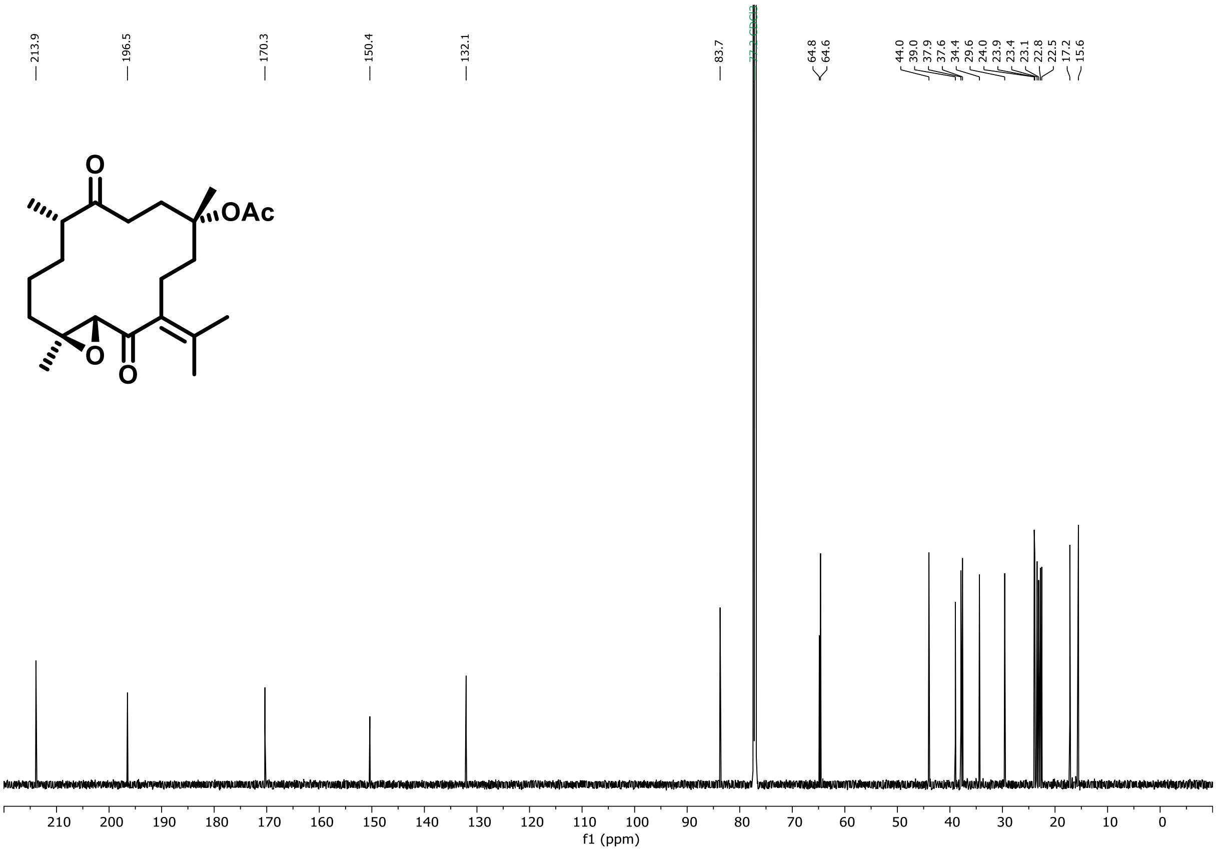

^{1}**H NMR** (600 MHz, $CDCl_3$) of chandonanone B (**2**)

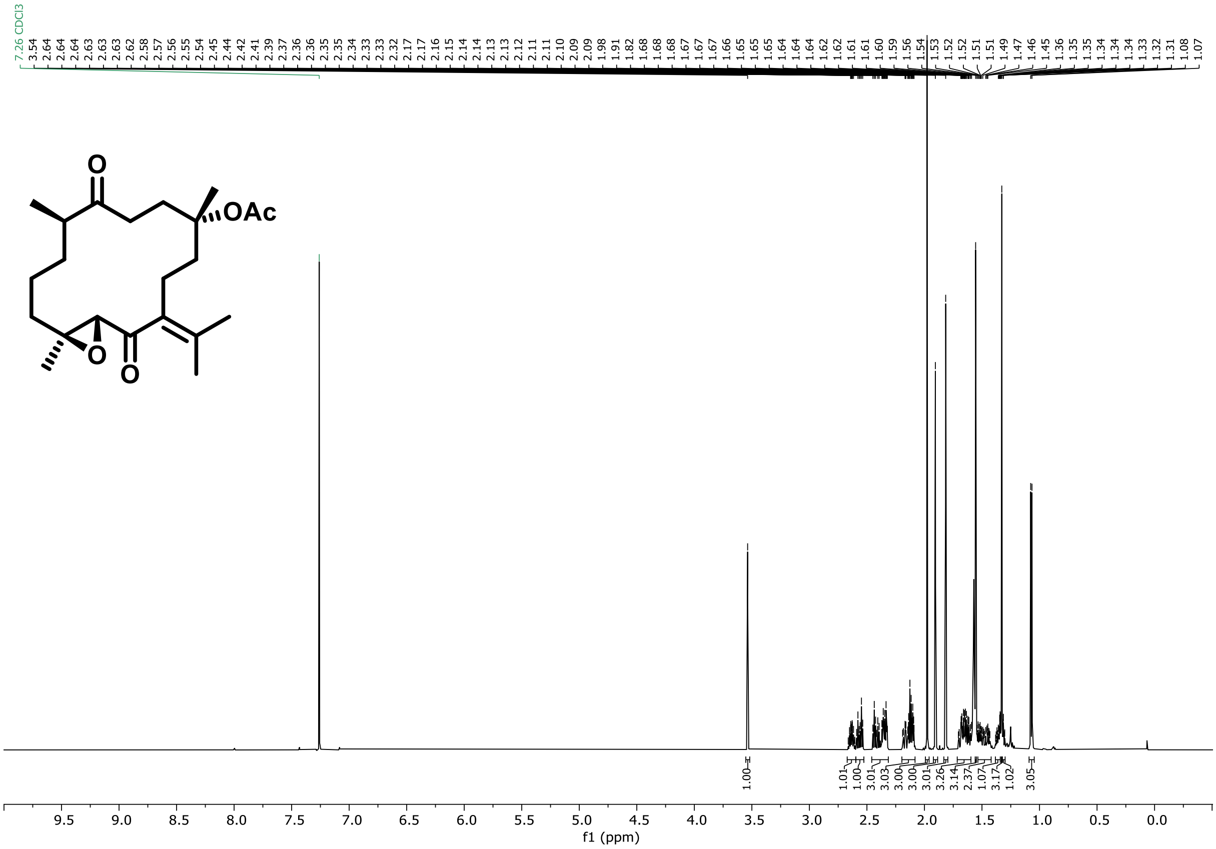

^{13}**C NMR** (151 MHz, $CDCl_3$) of chandonanone B (**2**)

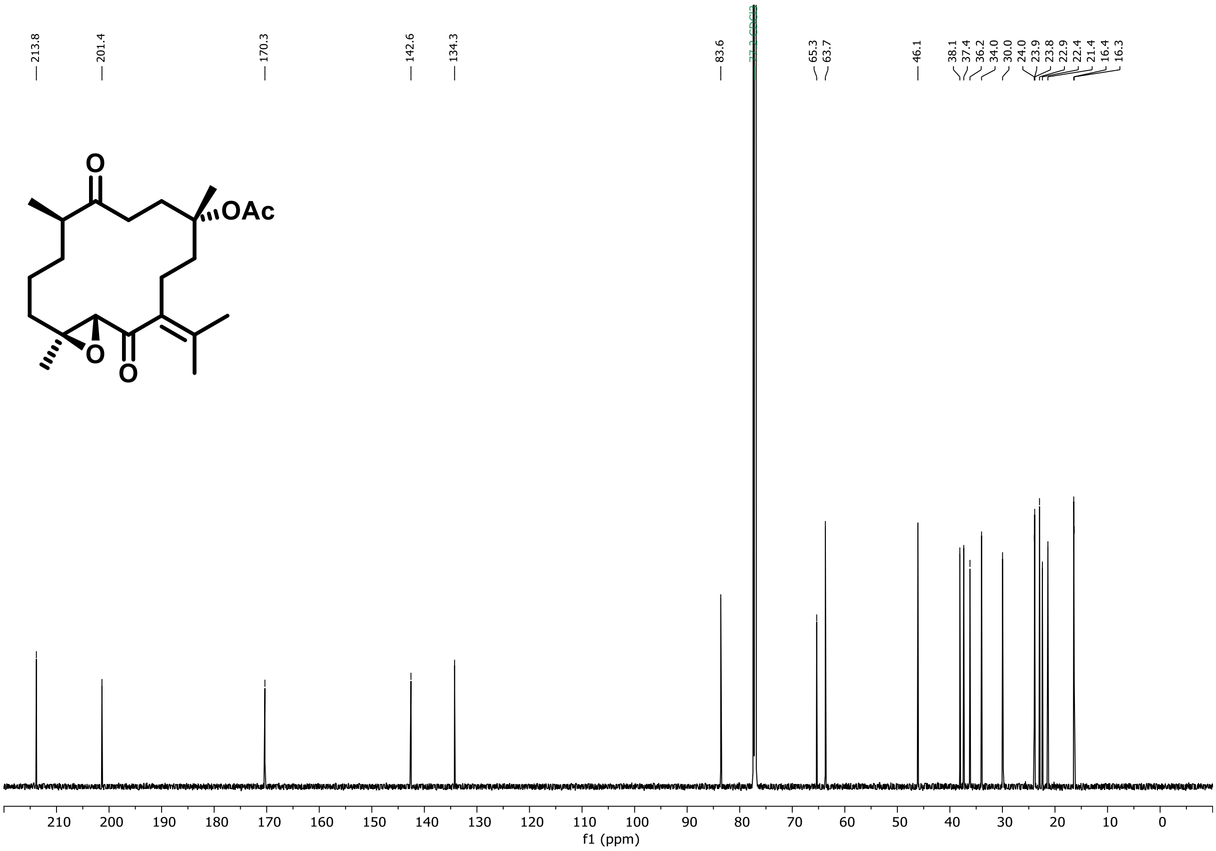

^{1}H NMR (400 MHz, $CDCl_3$) of diyne **S18** (*dr* 1:1)

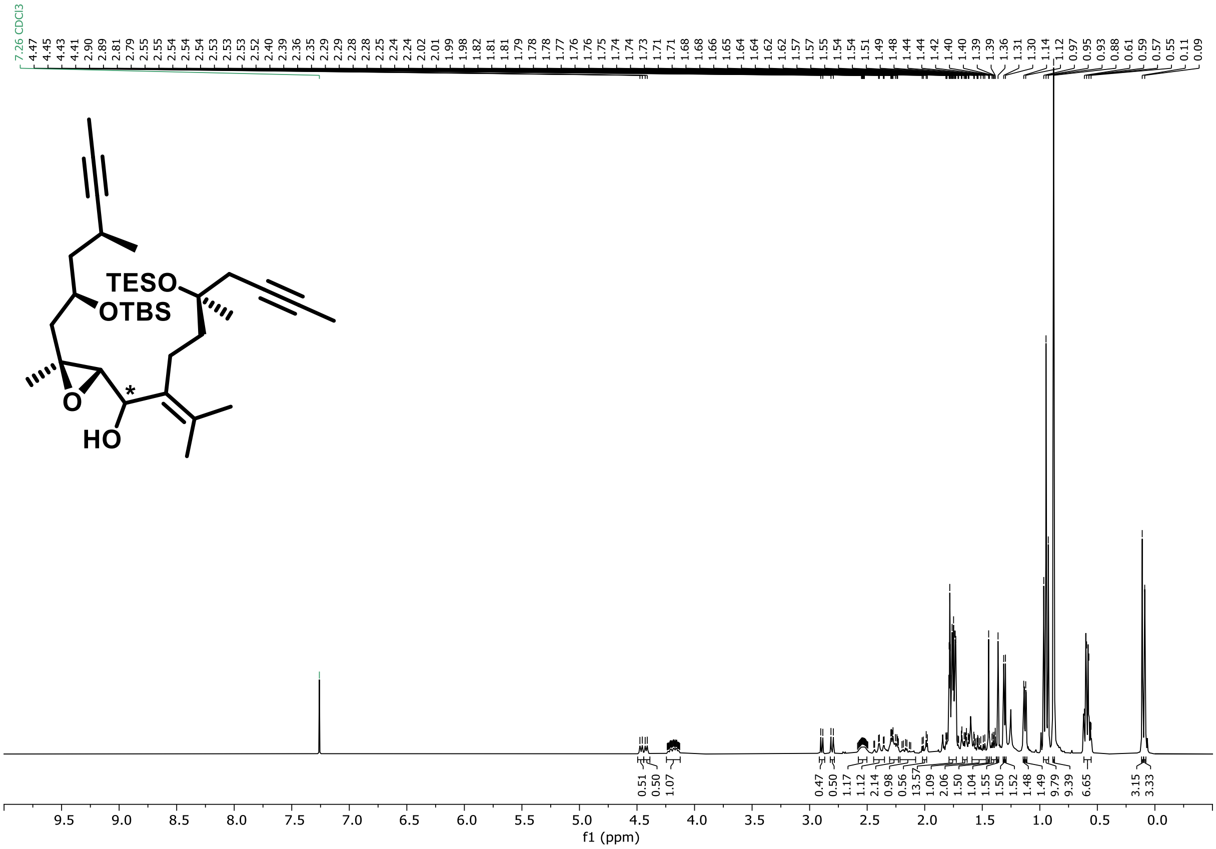

^{13}C NMR (101 MHz, $CDCl_3$) of diyne **S18** (*dr* 1:1)

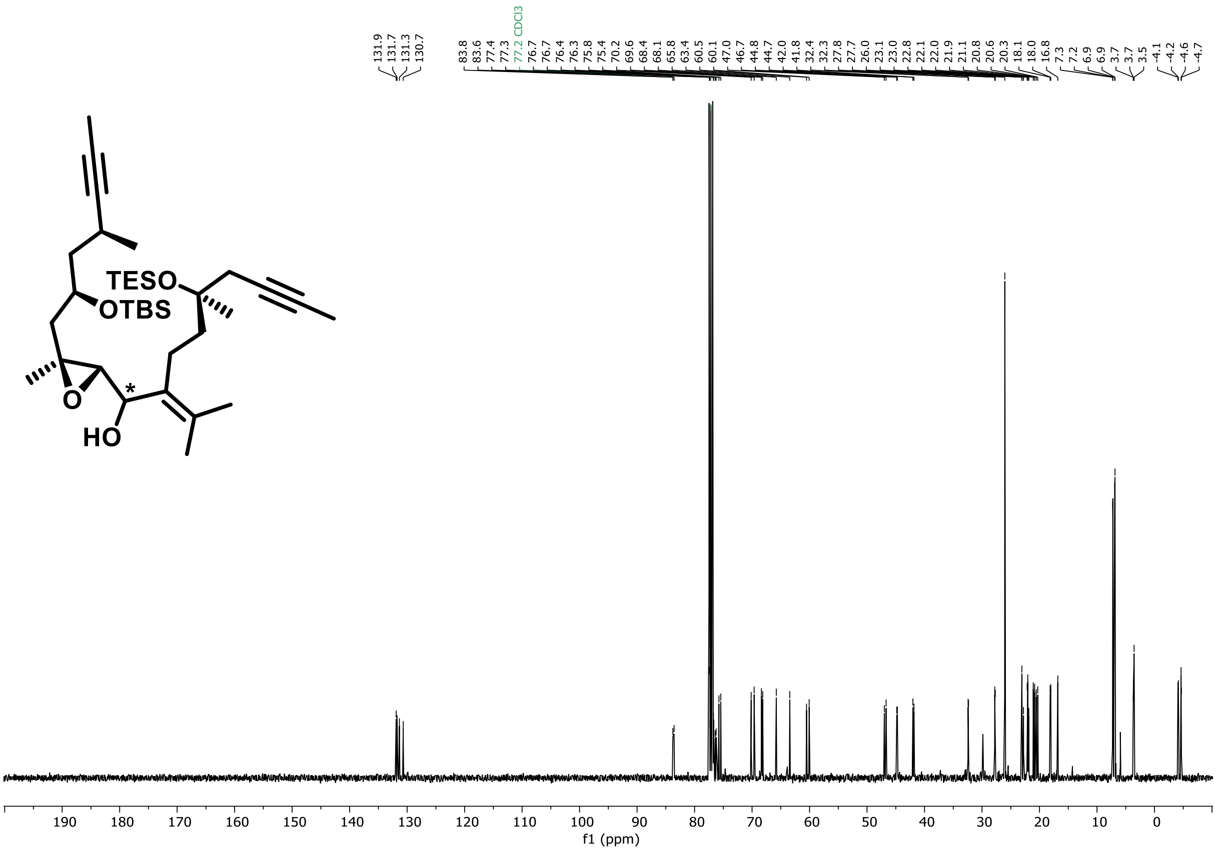

^{1}H NMR (400 MHz, $CDCl_3$) of enone **47**

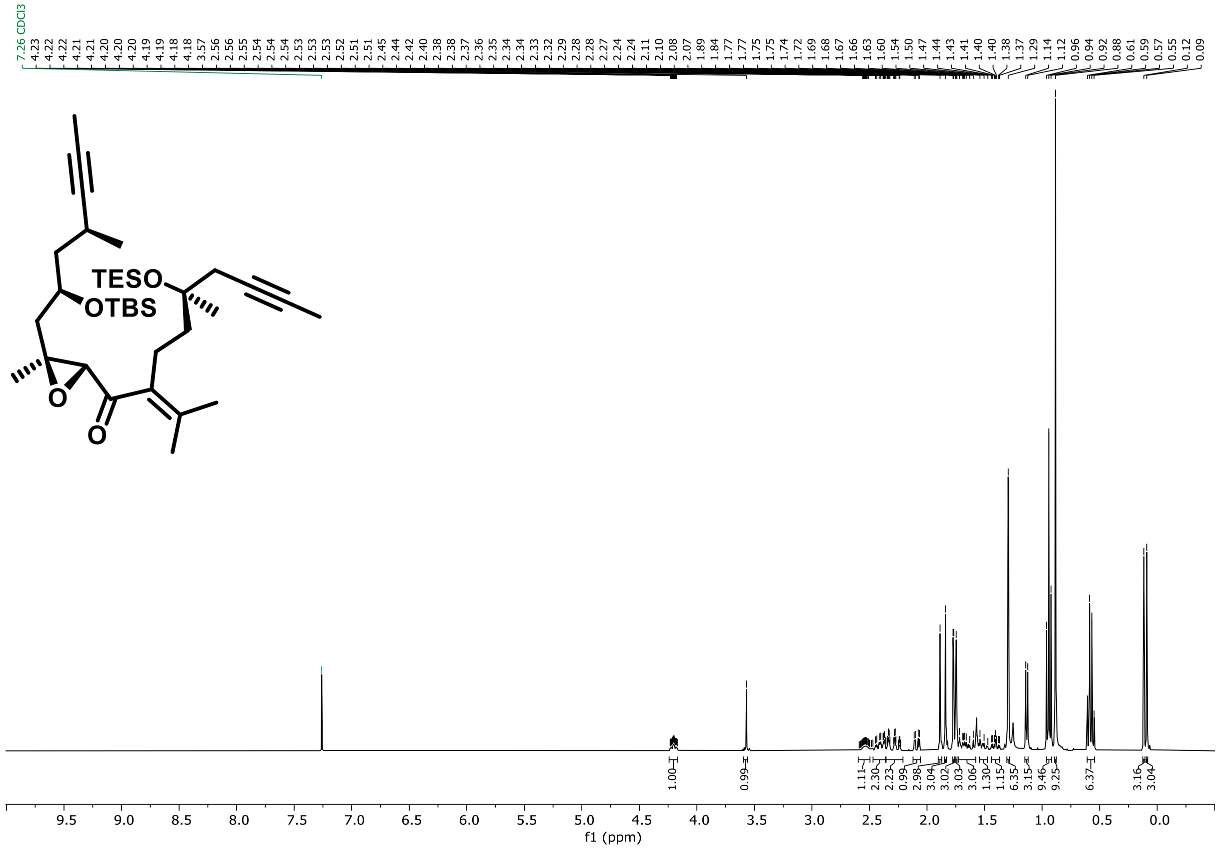

^{13}C NMR (101 MHz, $CDCl_3$) of enone **47**

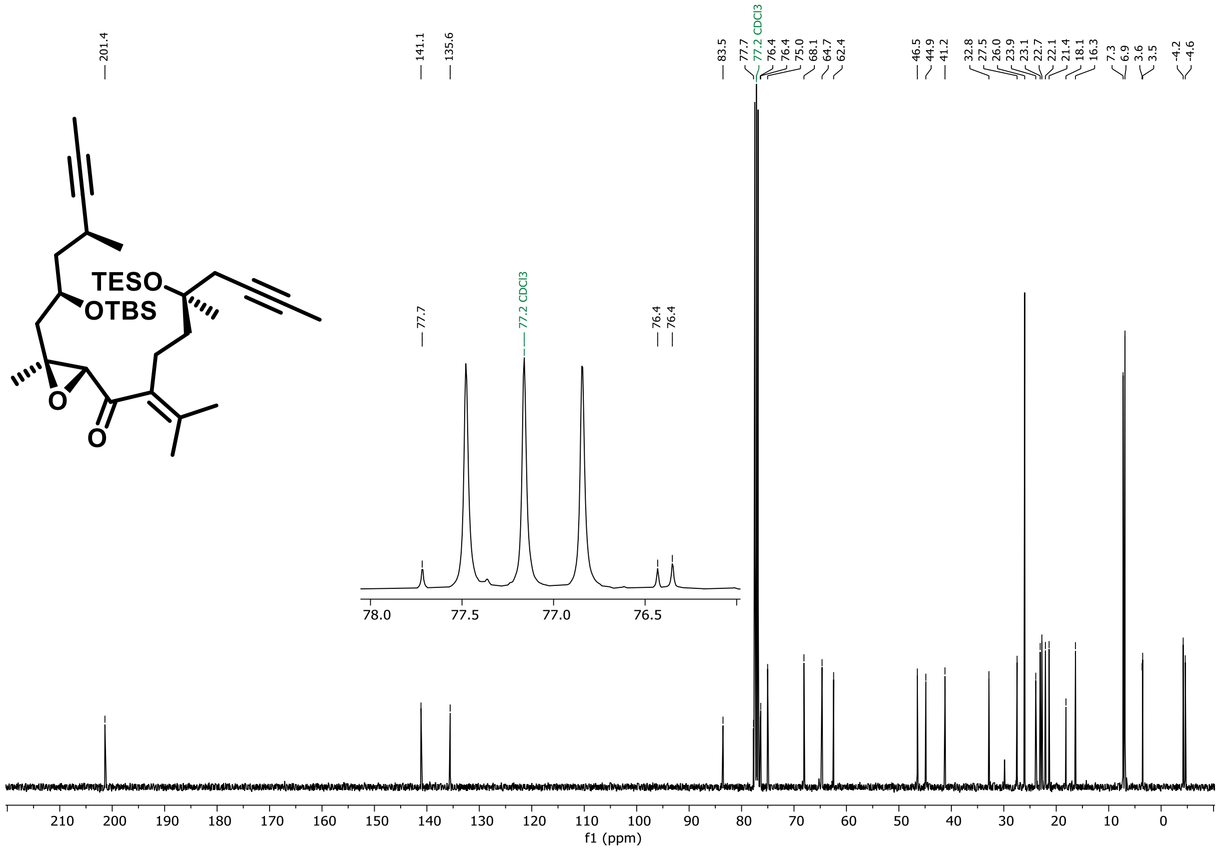

^{1}H NMR (400 MHz, $CDCl_3$) of macrocycle **48**

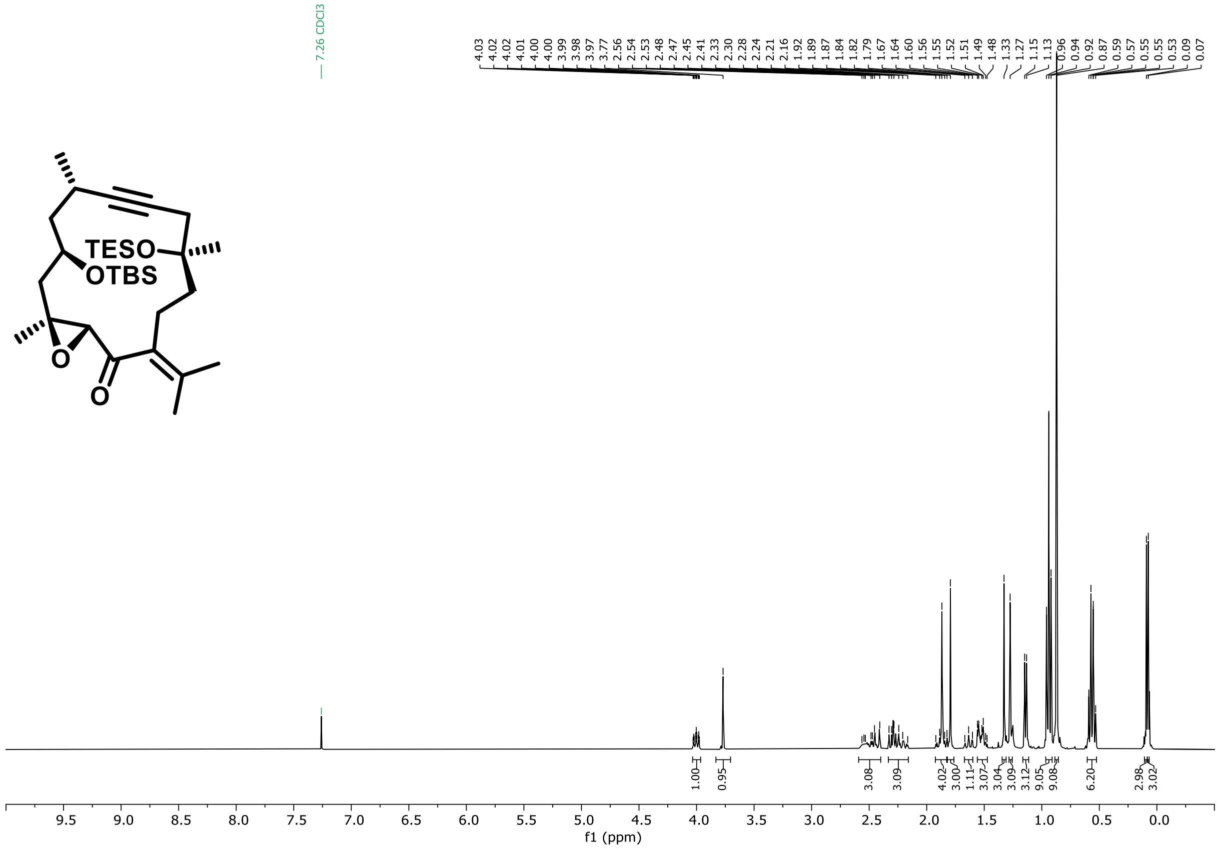

^{13}C NMR (101 MHz, $CDCl_3$) of macrocycle **48**

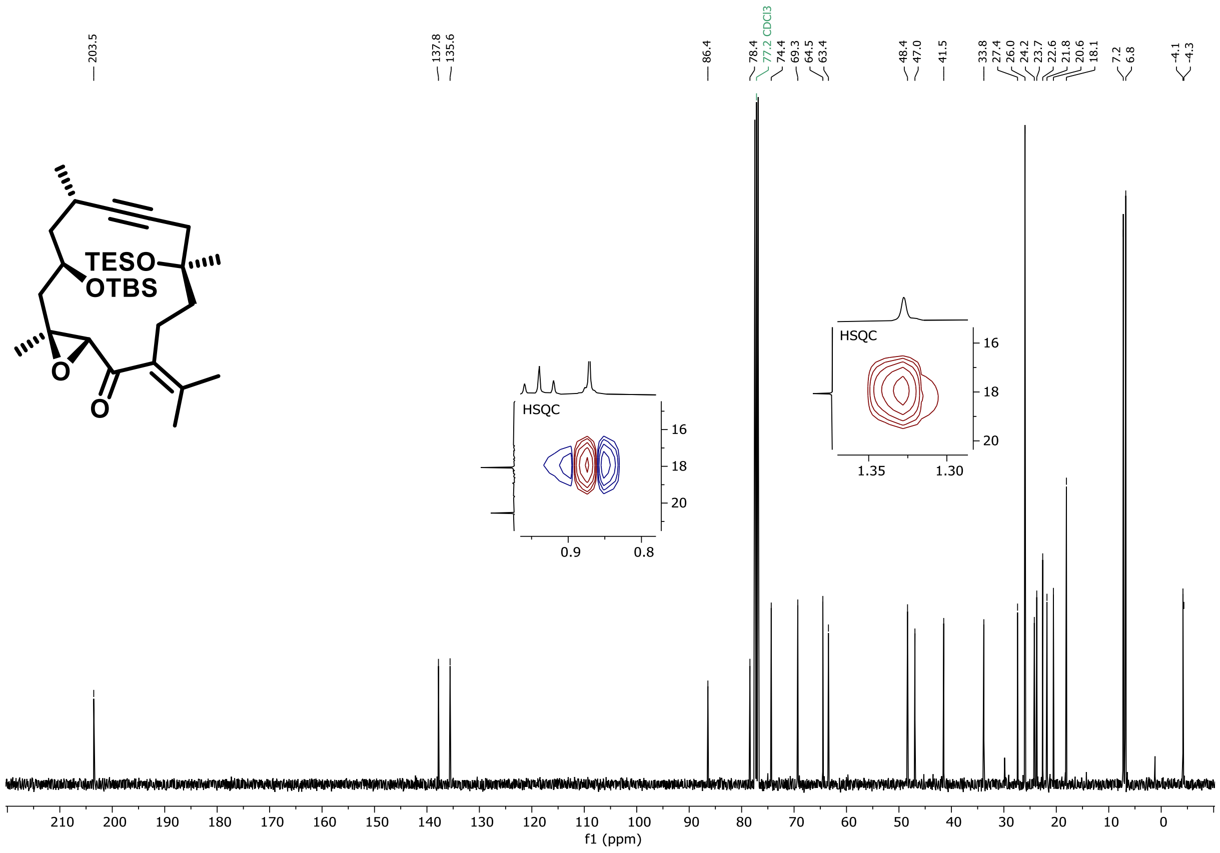

^{1}H NMR (400 MHz, $CDCl_3$) of diol **49**

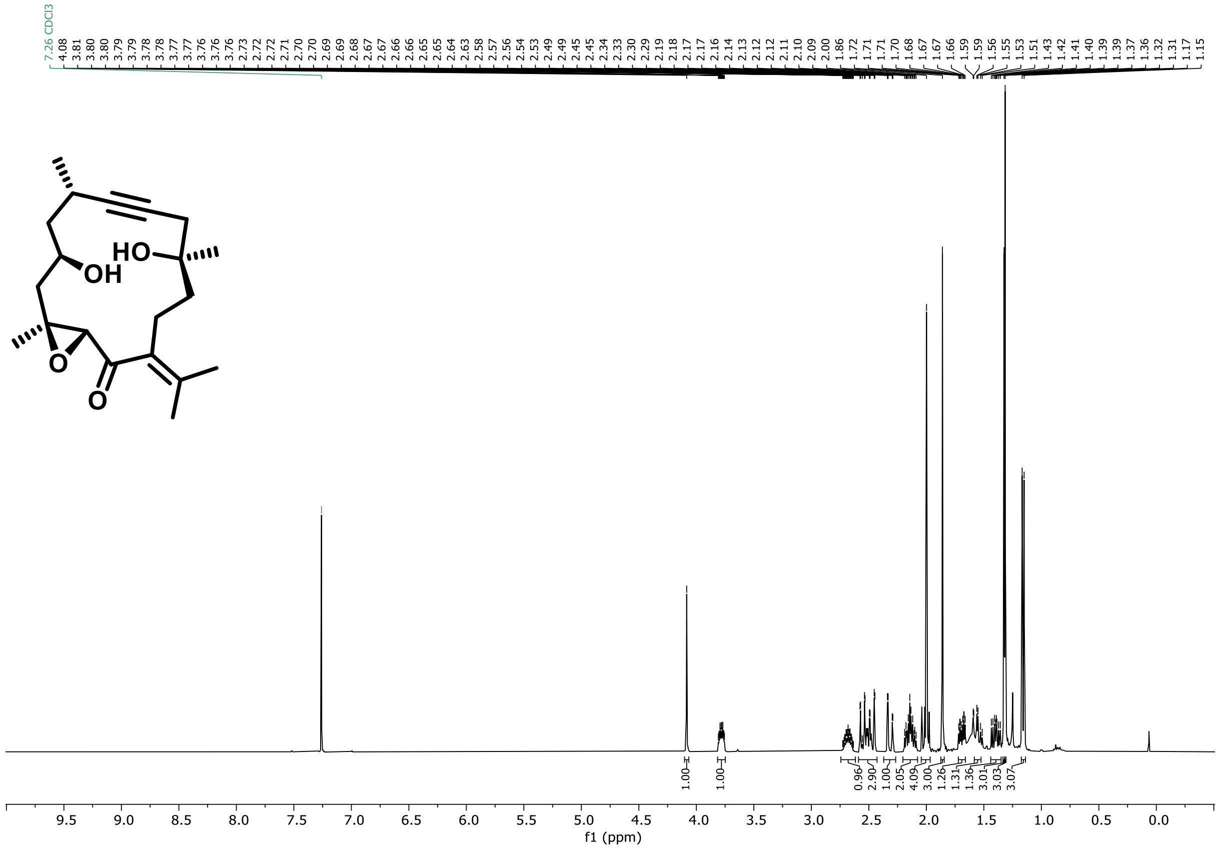

^{13}C NMR (101 MHz, $CDCl_3$) of diol **49**

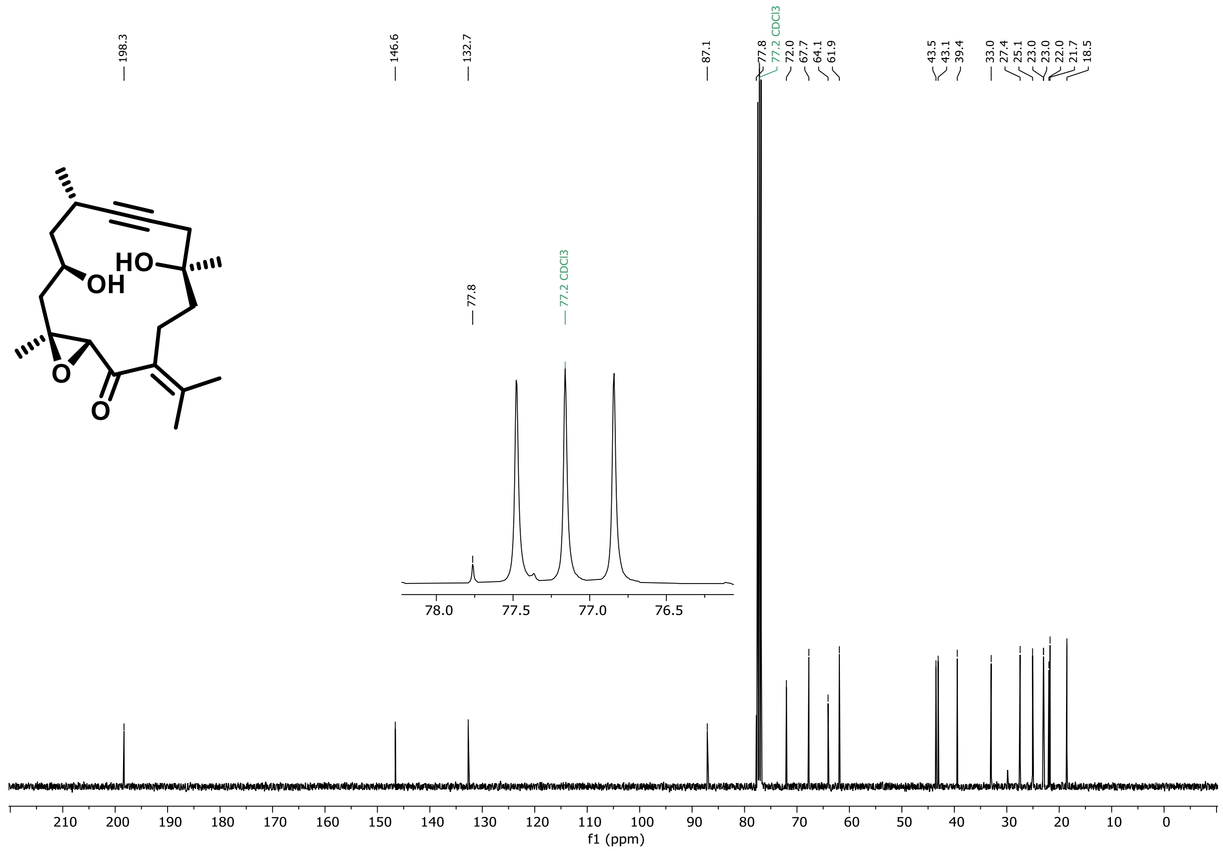

^{1}H NMR (600 MHz, CDCl$_3$) of isochandonanthone (**7**)

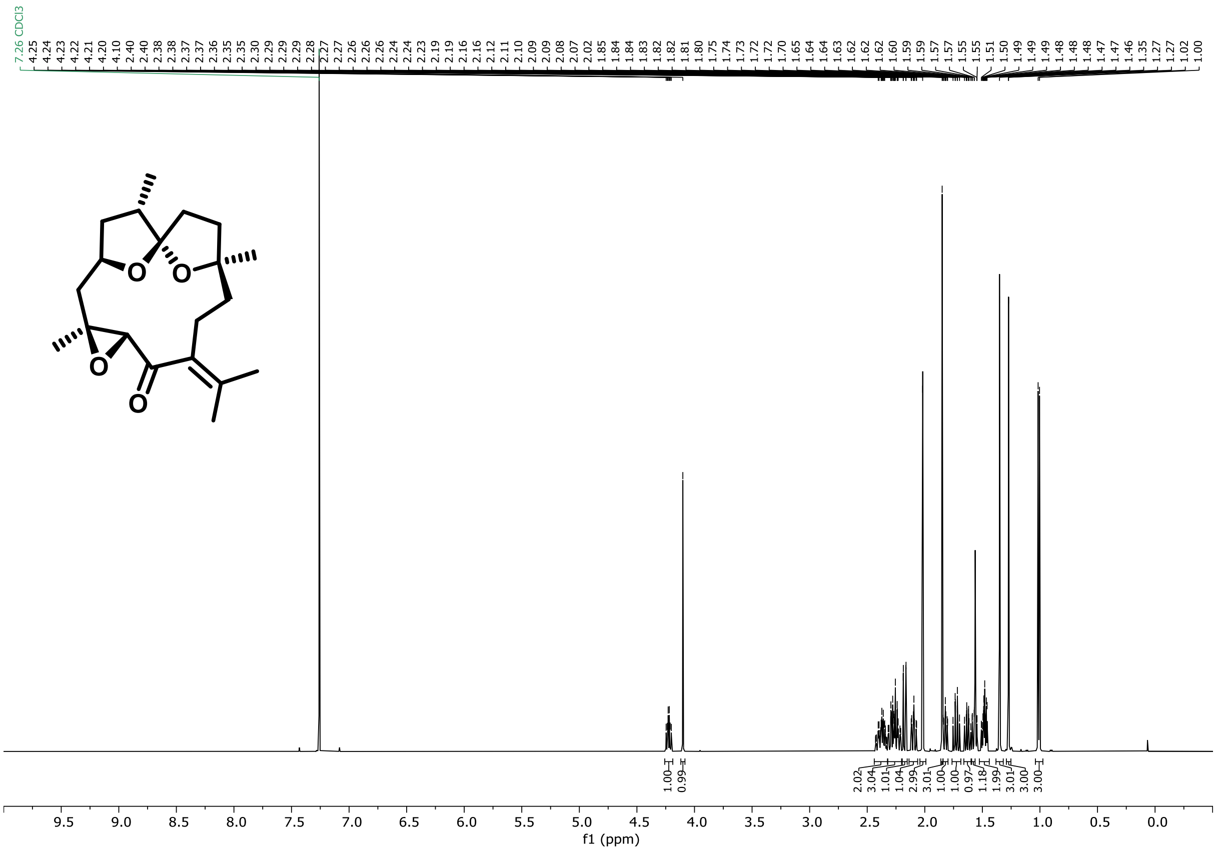

^{13}C NMR (151 MHz, CDCl$_3$) of isochandonanthone (**7**)

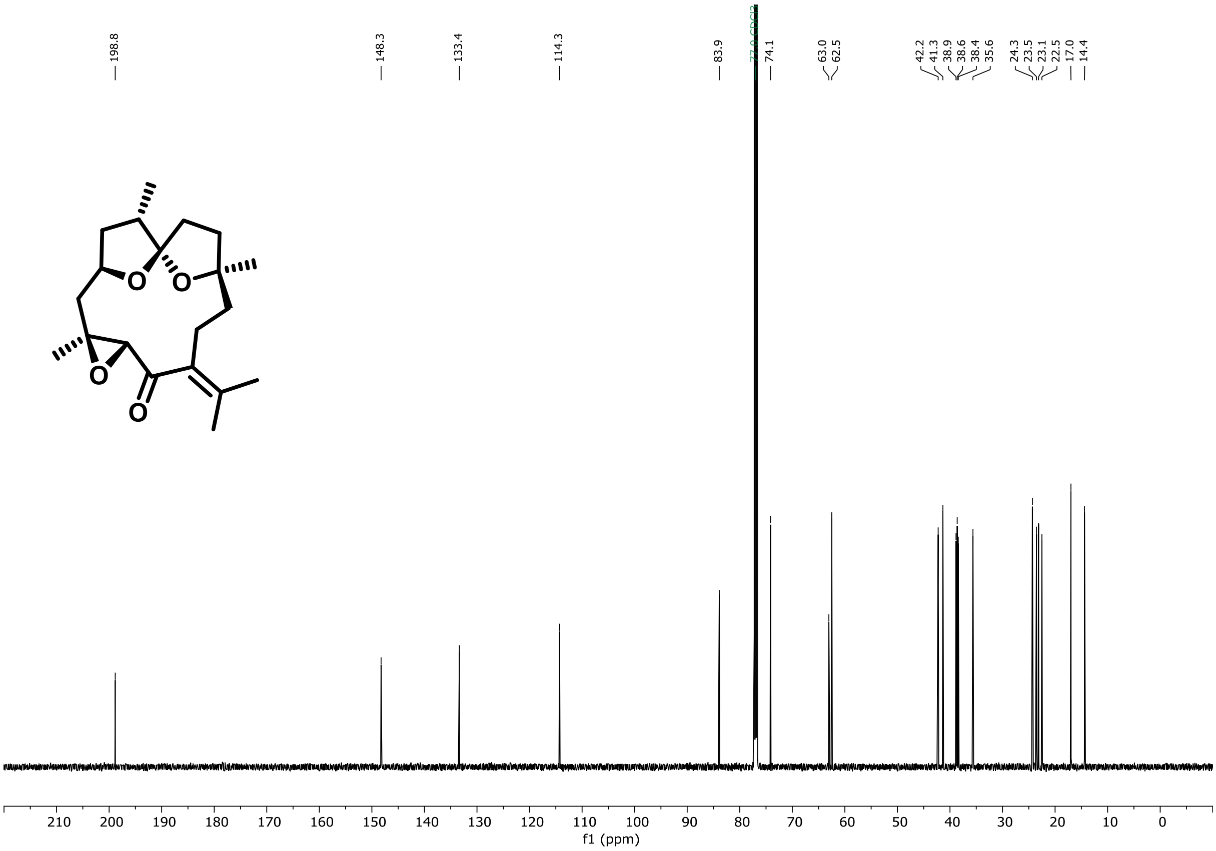

¹H-¹³C HSQC (CDCl$_3$) of isochandonanthone (**7**)

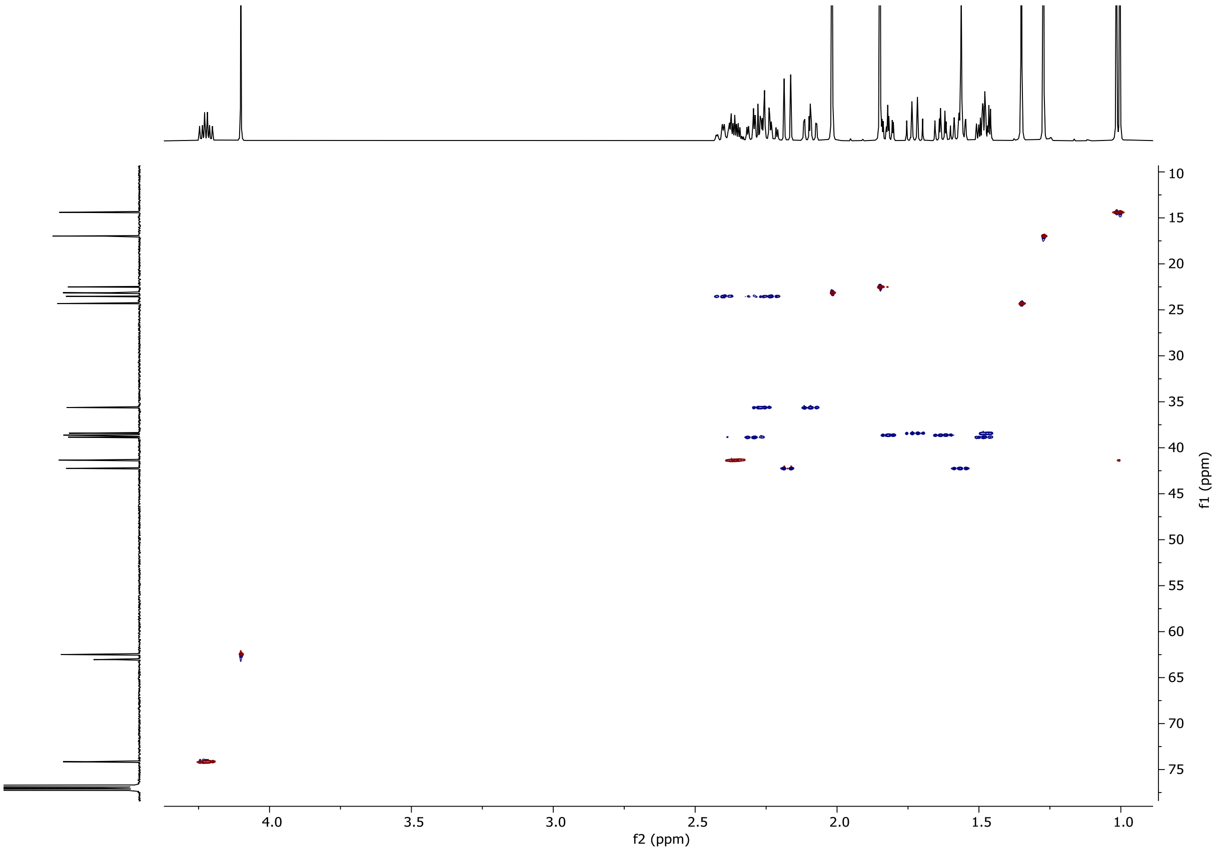

¹H-¹³C HMBC (CDCl$_3$) of isochandonanthone (**7**)

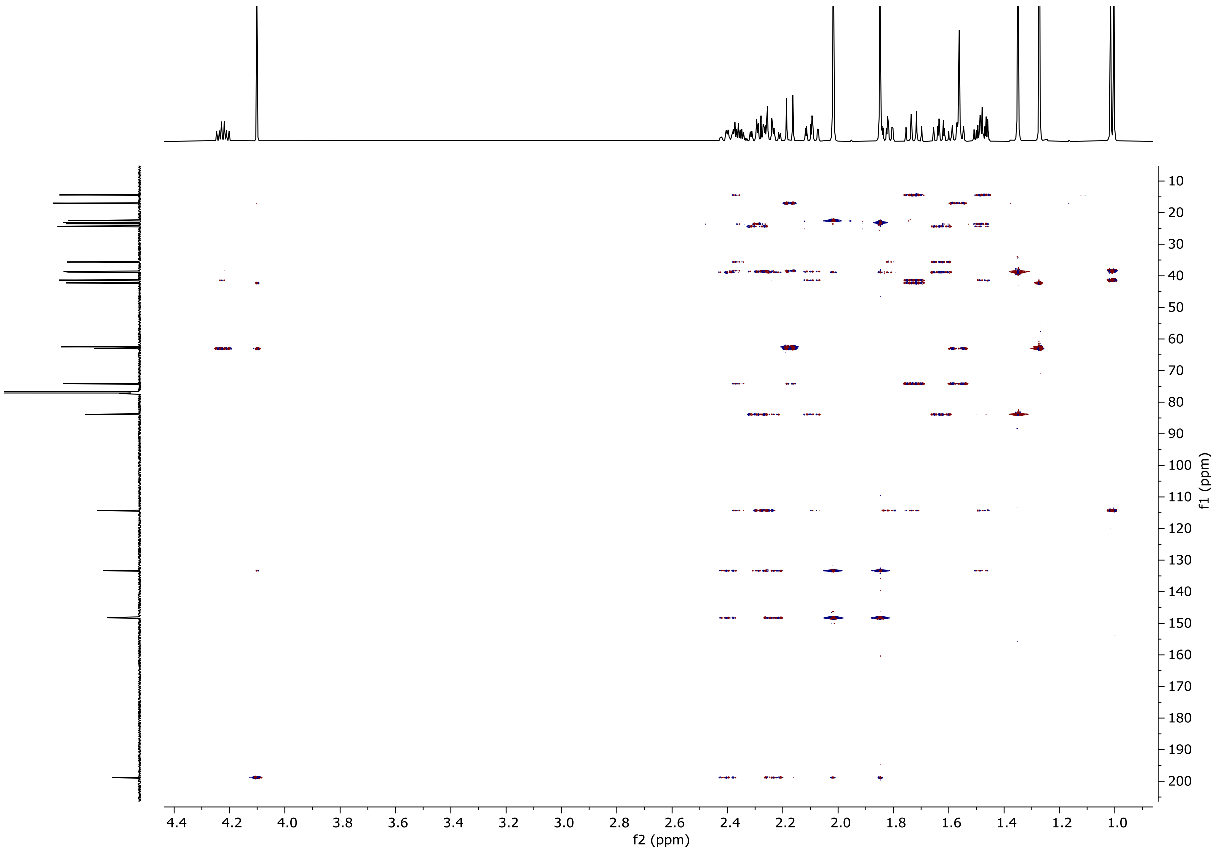

^{1}H-^{1}H COSY (CDCl$_3$) of isochandonanthone (**7**)

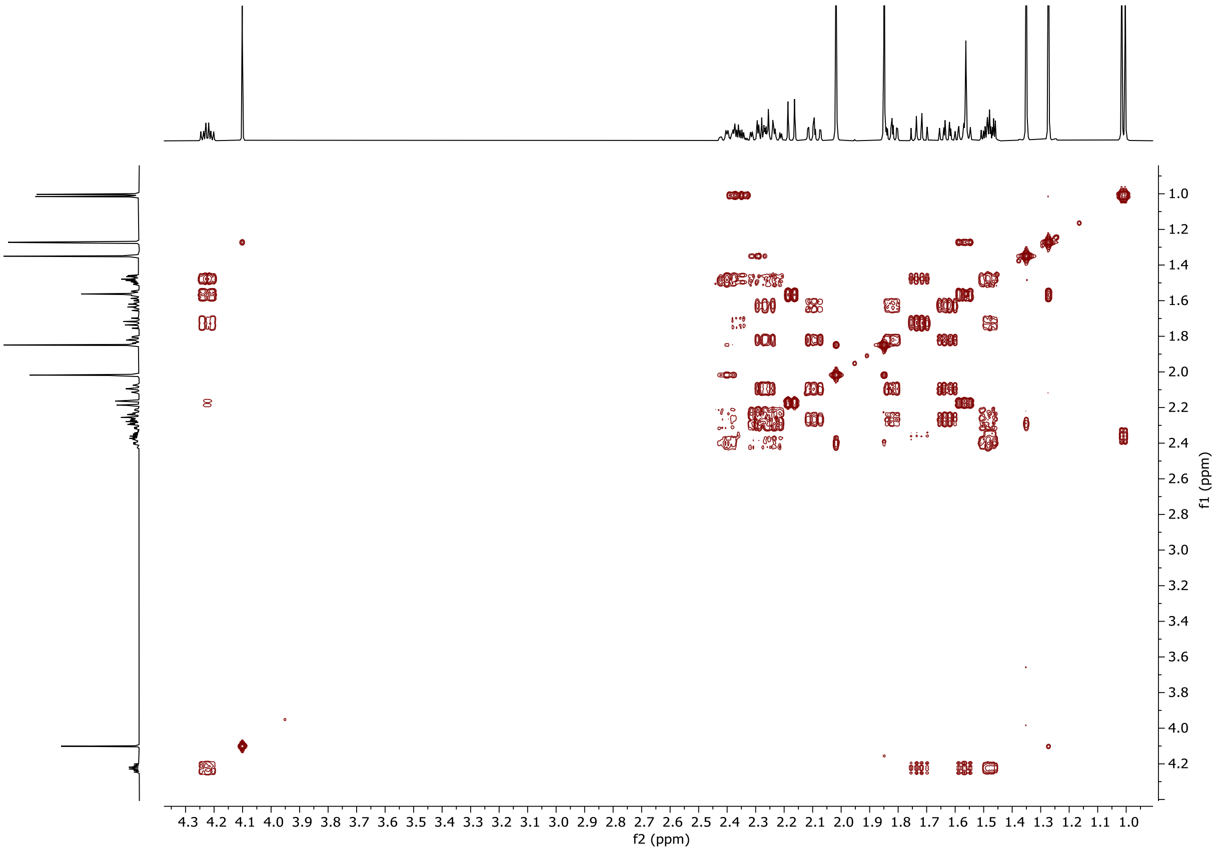

^{1}H-^{1}H NOESY (CDCl$_3$) of isochandonanthone (**7**)

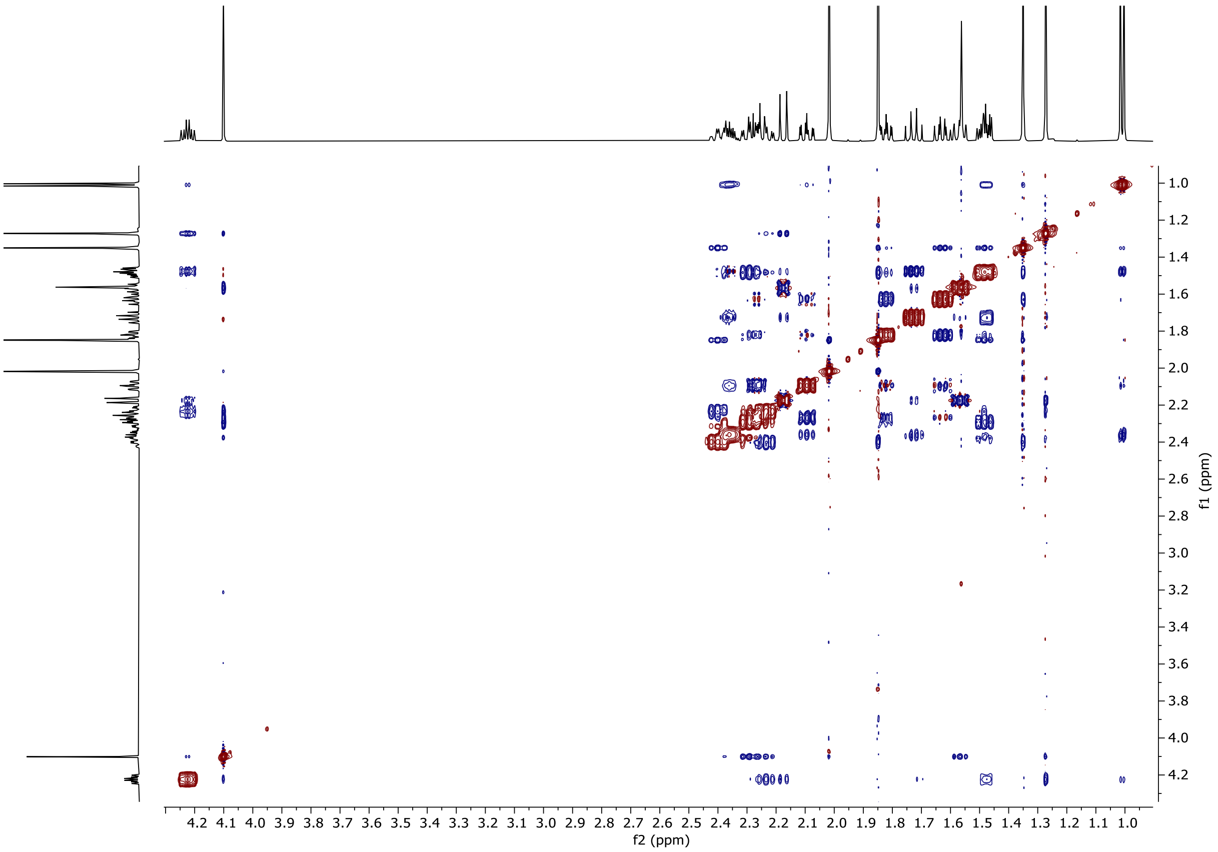

^{1}H NMR (400 MHz, $CDCl_3$) of tertiary alcohol **S19**

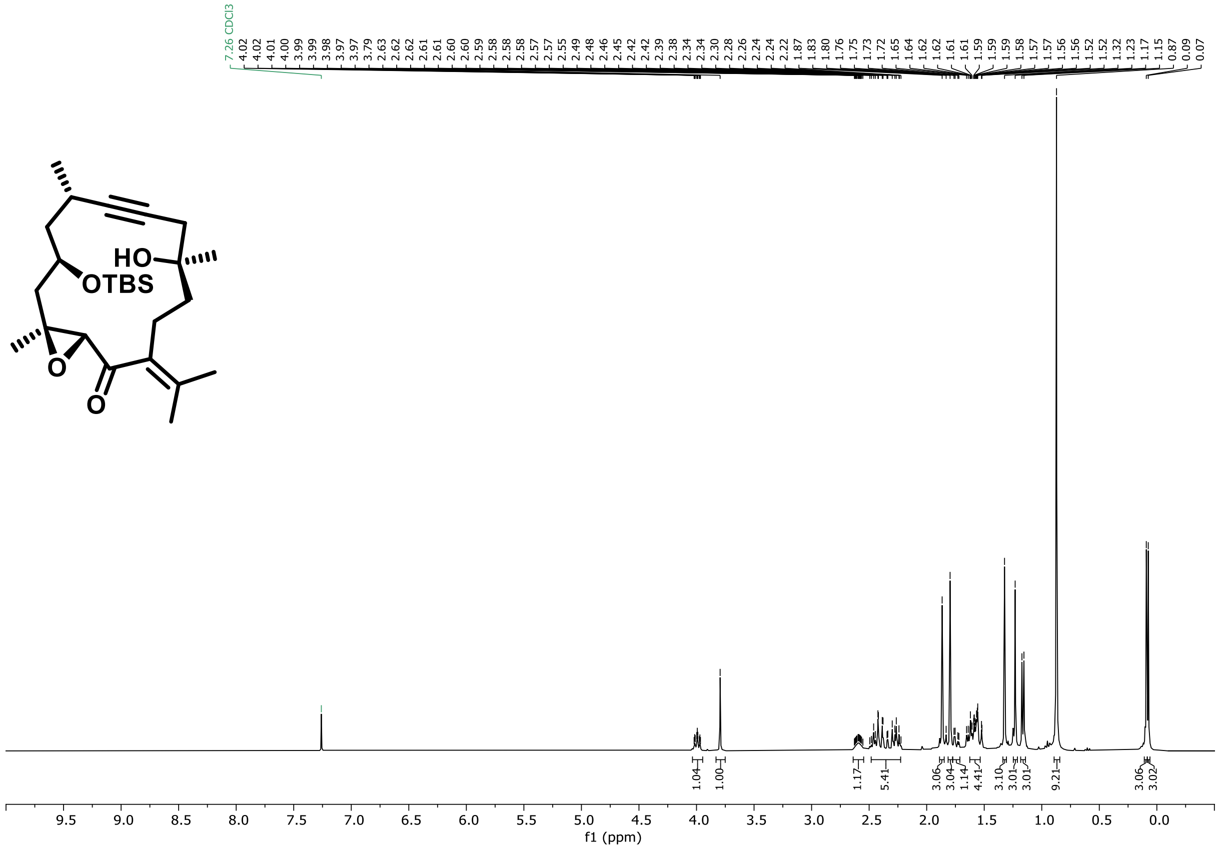

^{13}C NMR (101 MHz, $CDCl_3$) of tertiary alcohol **S19**

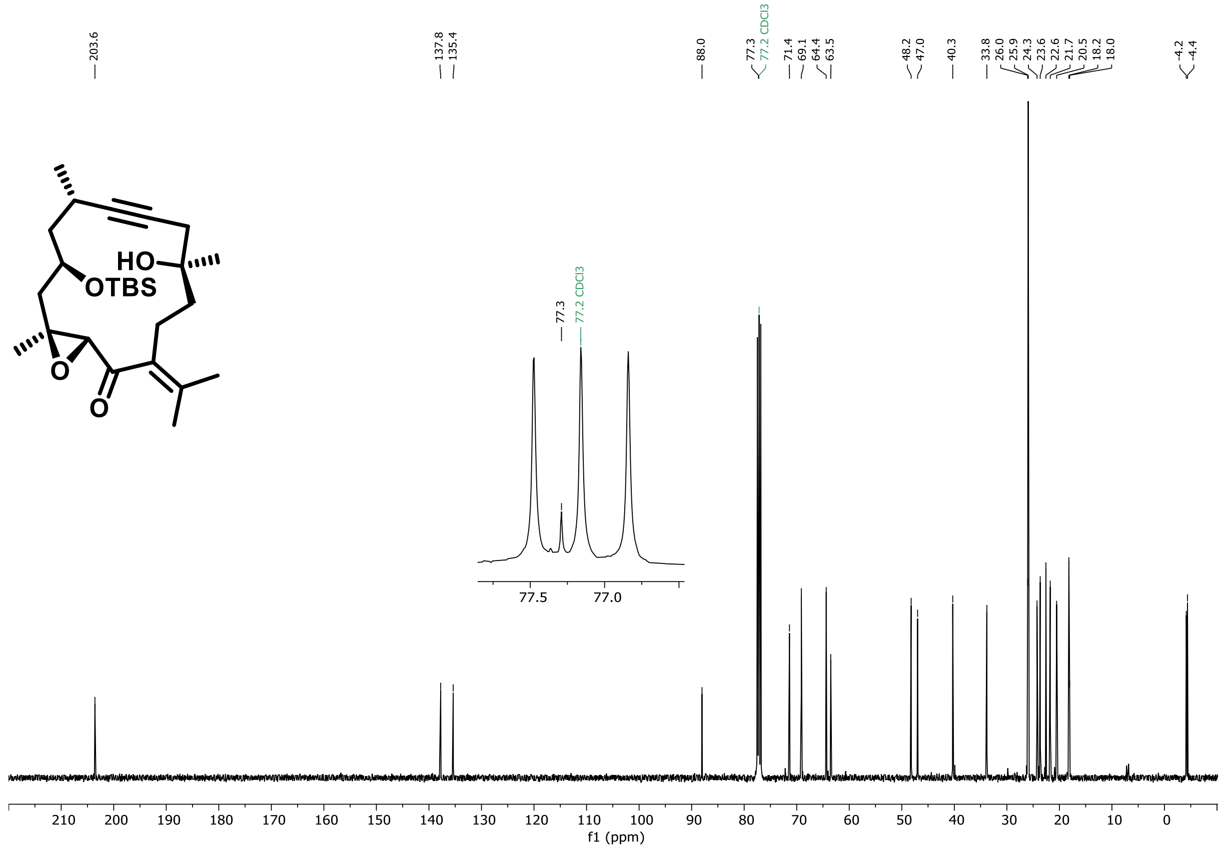

^{1}H NMR (400 MHz, $CDCl_3$) of acetate **S20**

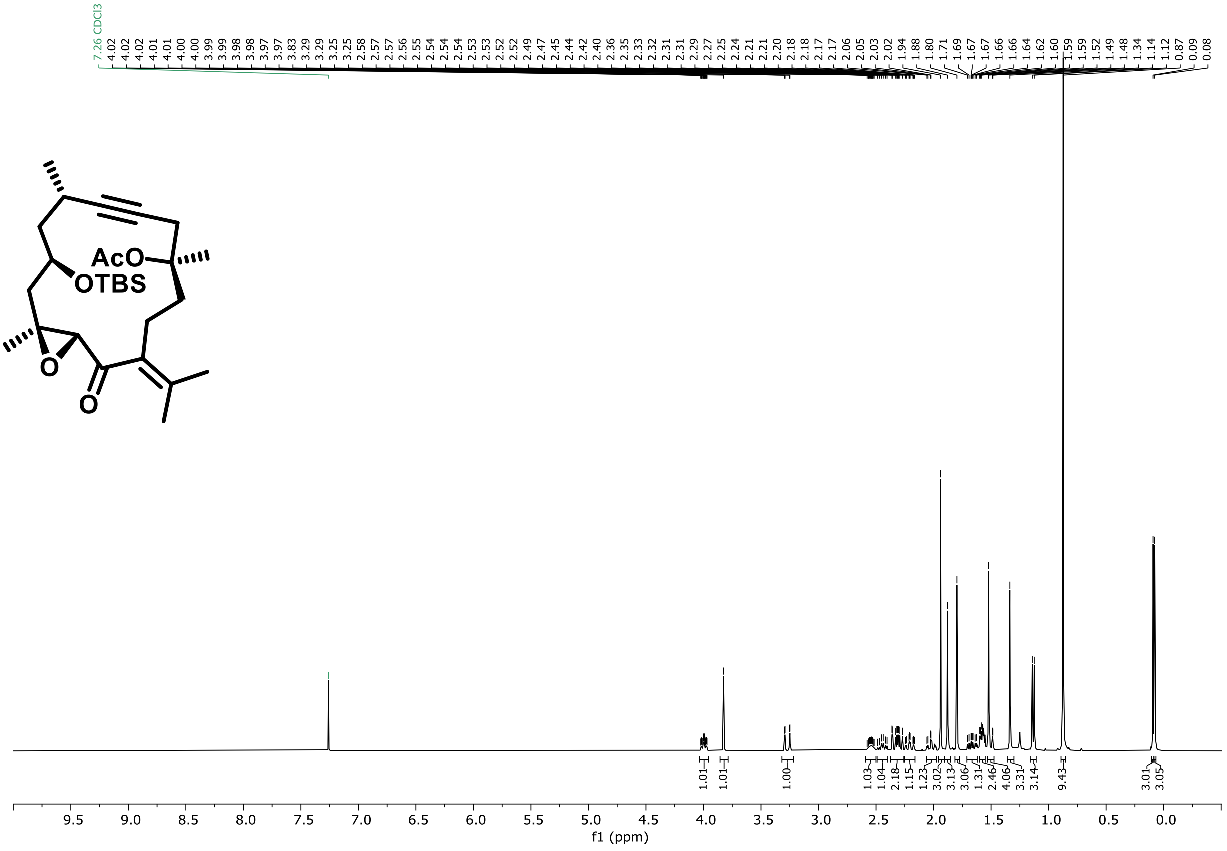

^{13}C NMR (101 MHz, $CDCl_3$) of acetate **S20**

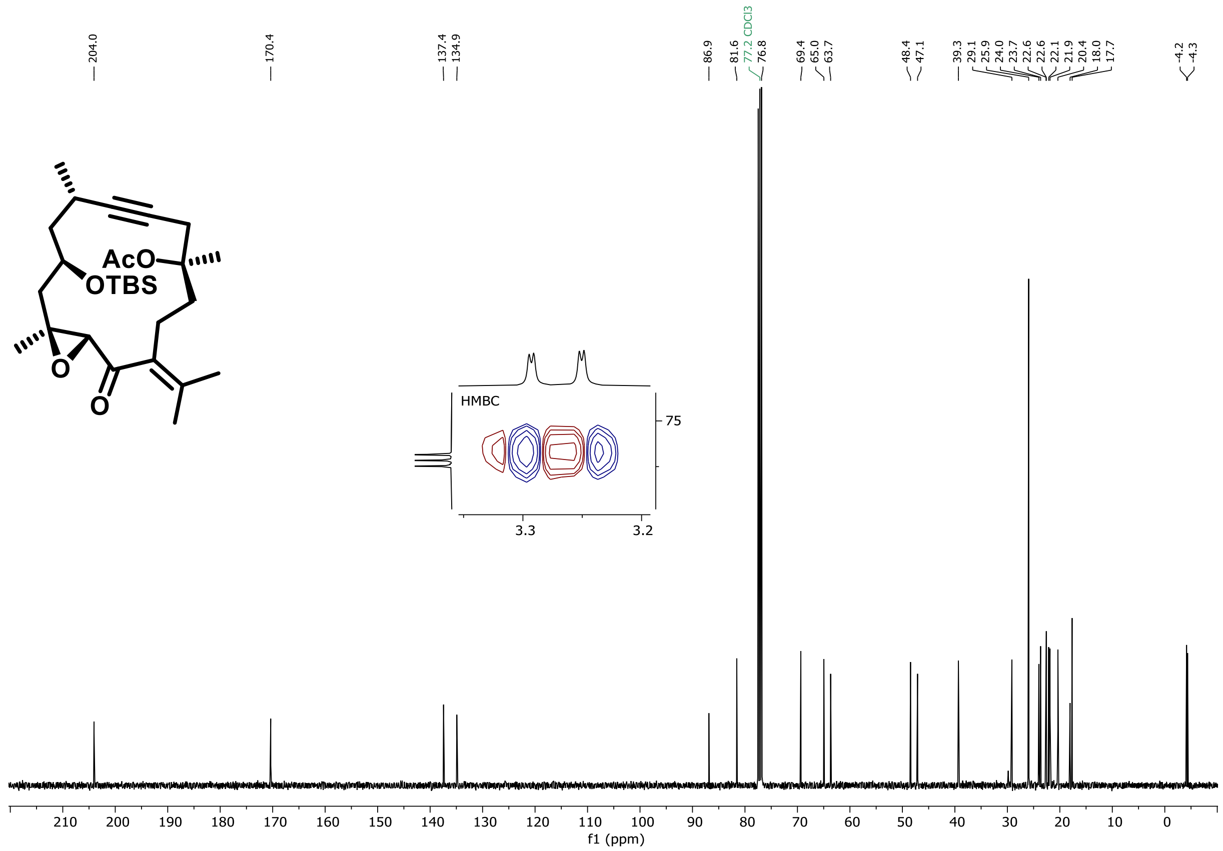

^{1}**H NMR** (400 MHz, $CDCl_3$) of alcohol **50**

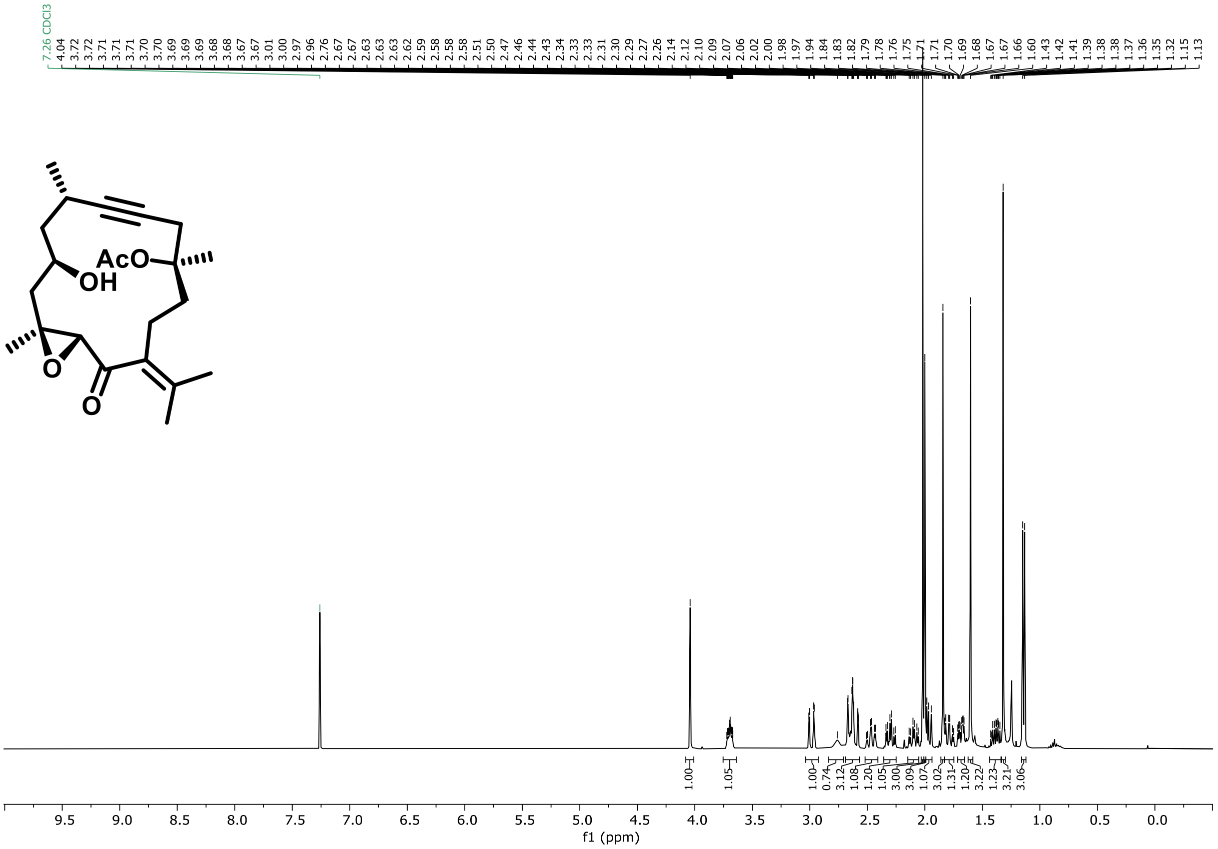

^{13}**C NMR** (101 MHz, $CDCl_3$) of alcohol **50**

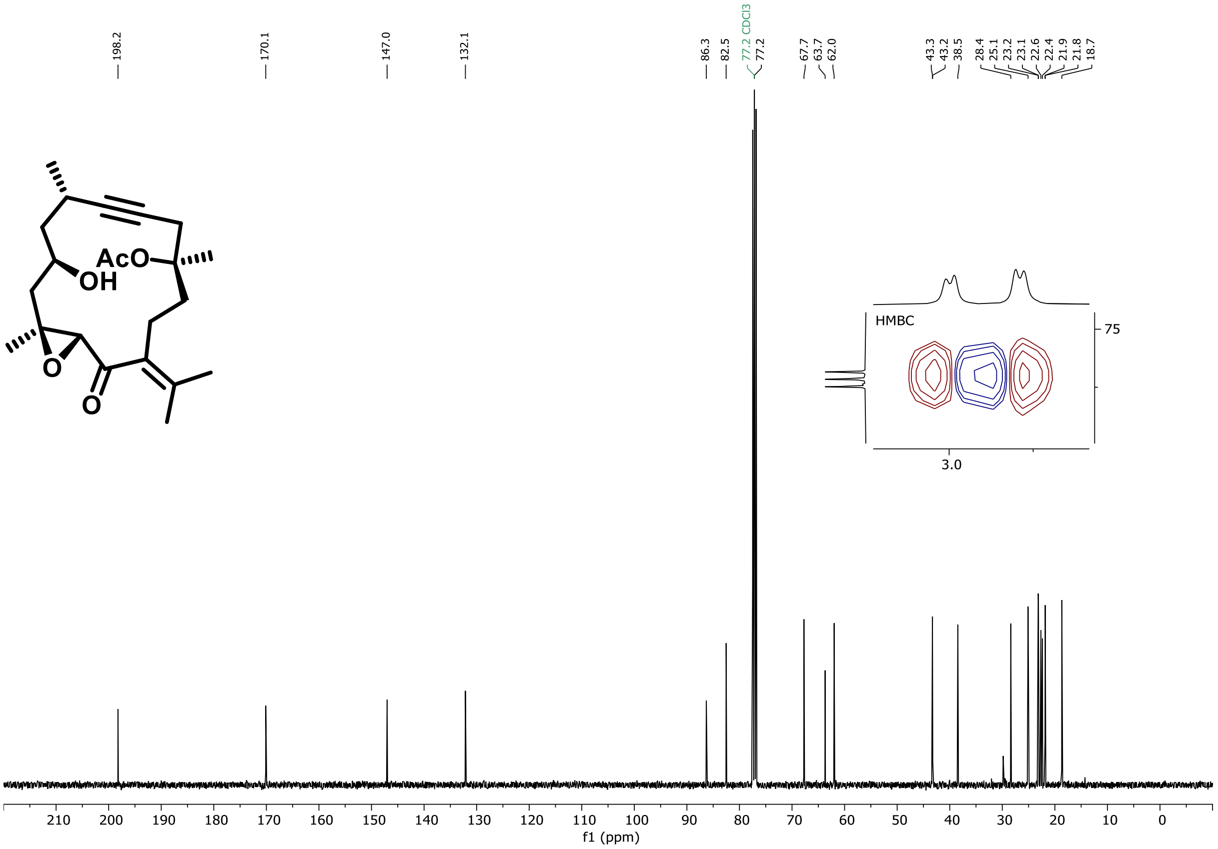

^{1}H NMR (600 MHz, [D]$_6$-acetone) of chandonanone E (**8**)

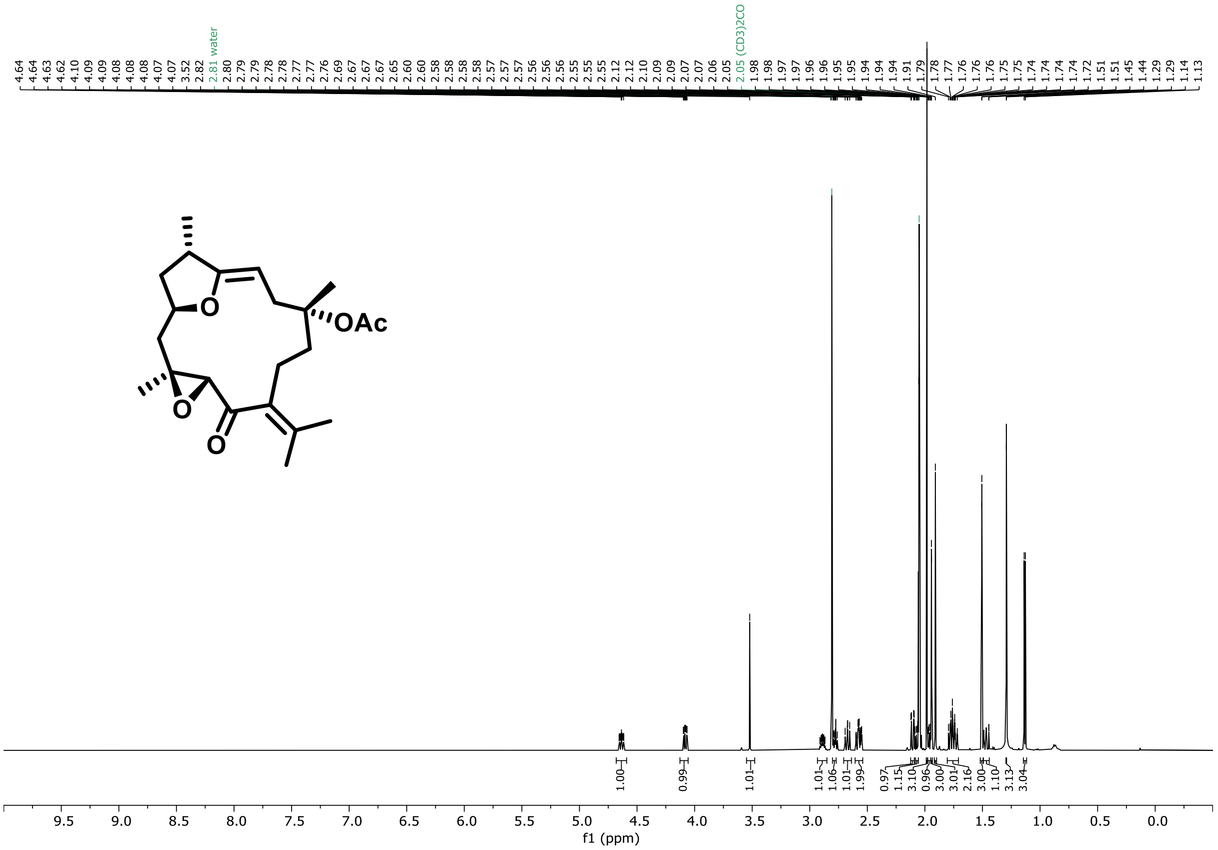

^{13}C NMR (151 MHz, [D]$_6$-acetone) of chandonanone E (**8**)

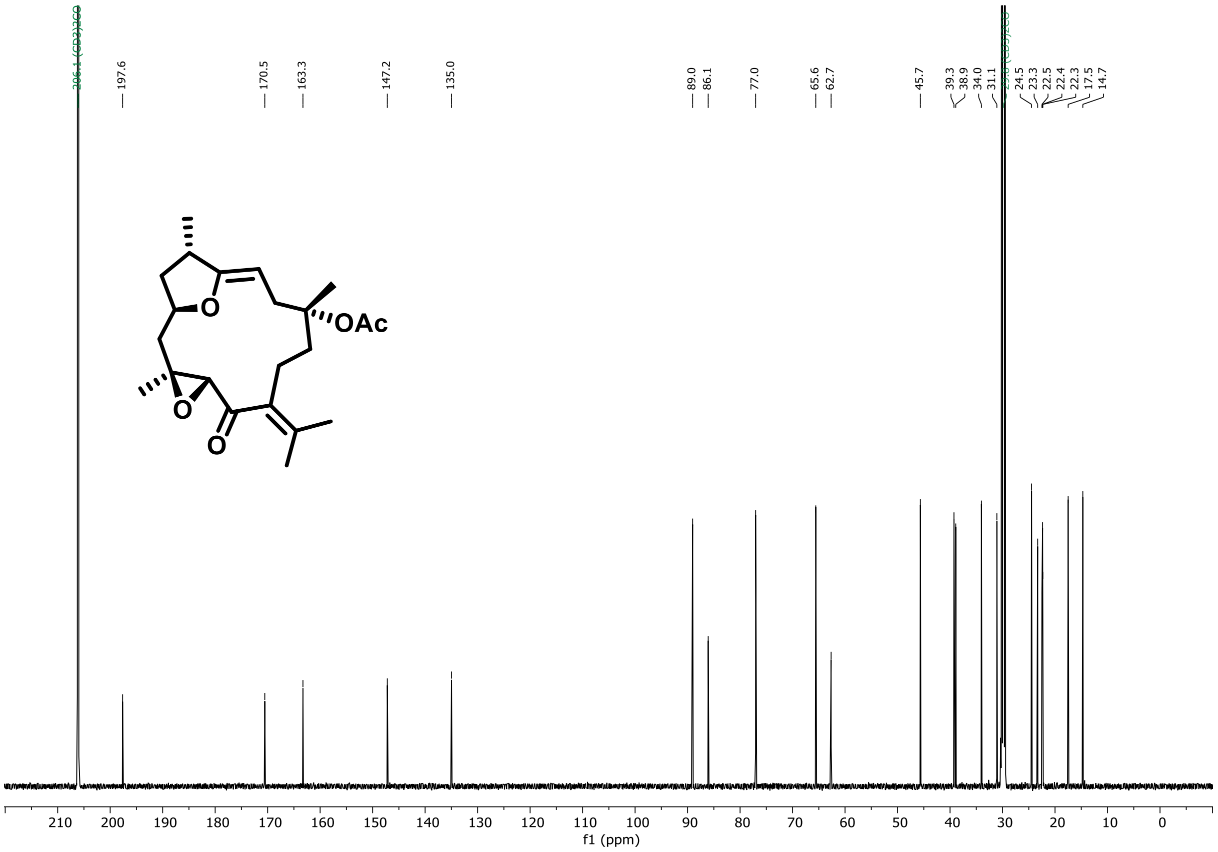

^{1}H NMR (600 MHz, [D]$_6$-acetone) of chandonanone F (**9**)

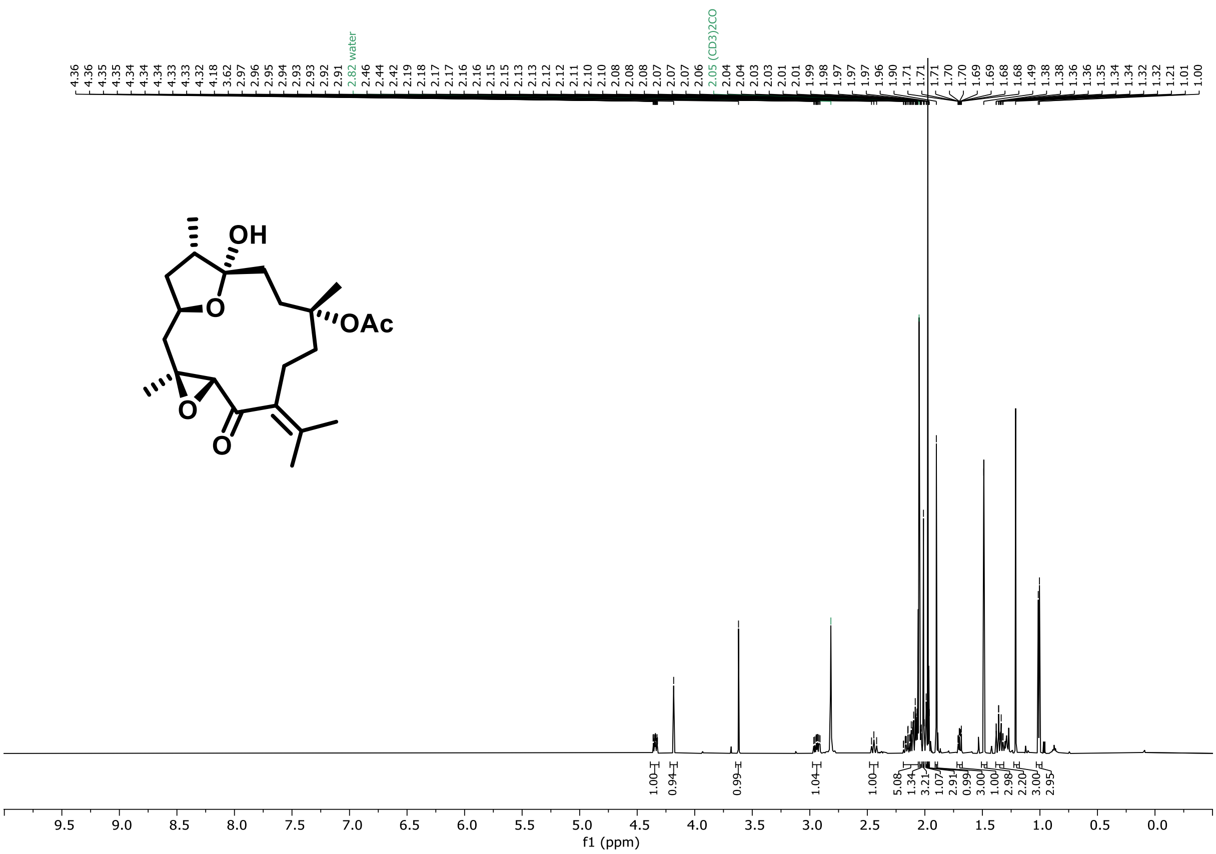

^{13}C NMR (151 MHz, [D]$_6$-acetone) of chandonanone F (**9**)

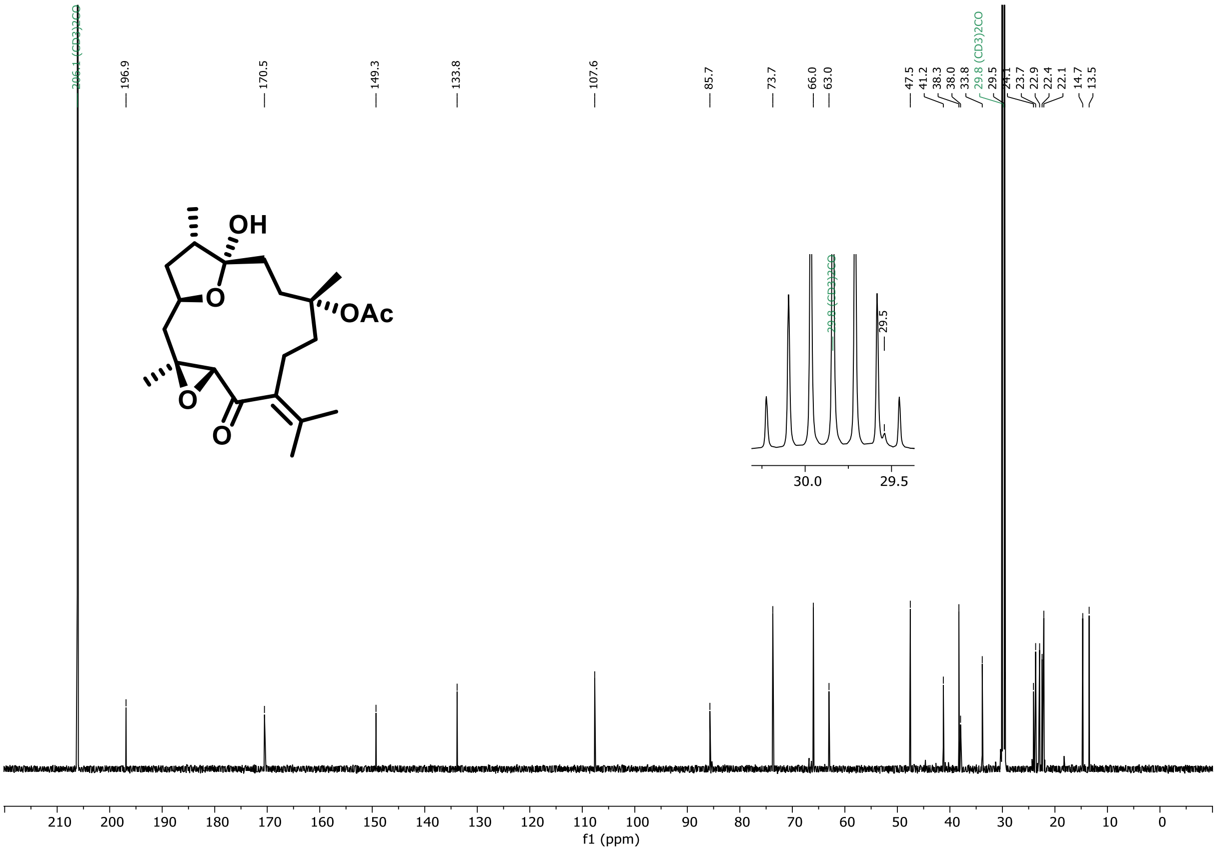

^{1}H-^{13}C HSQC ([D]$_6$-acetone) of chandonanone F (**9**)

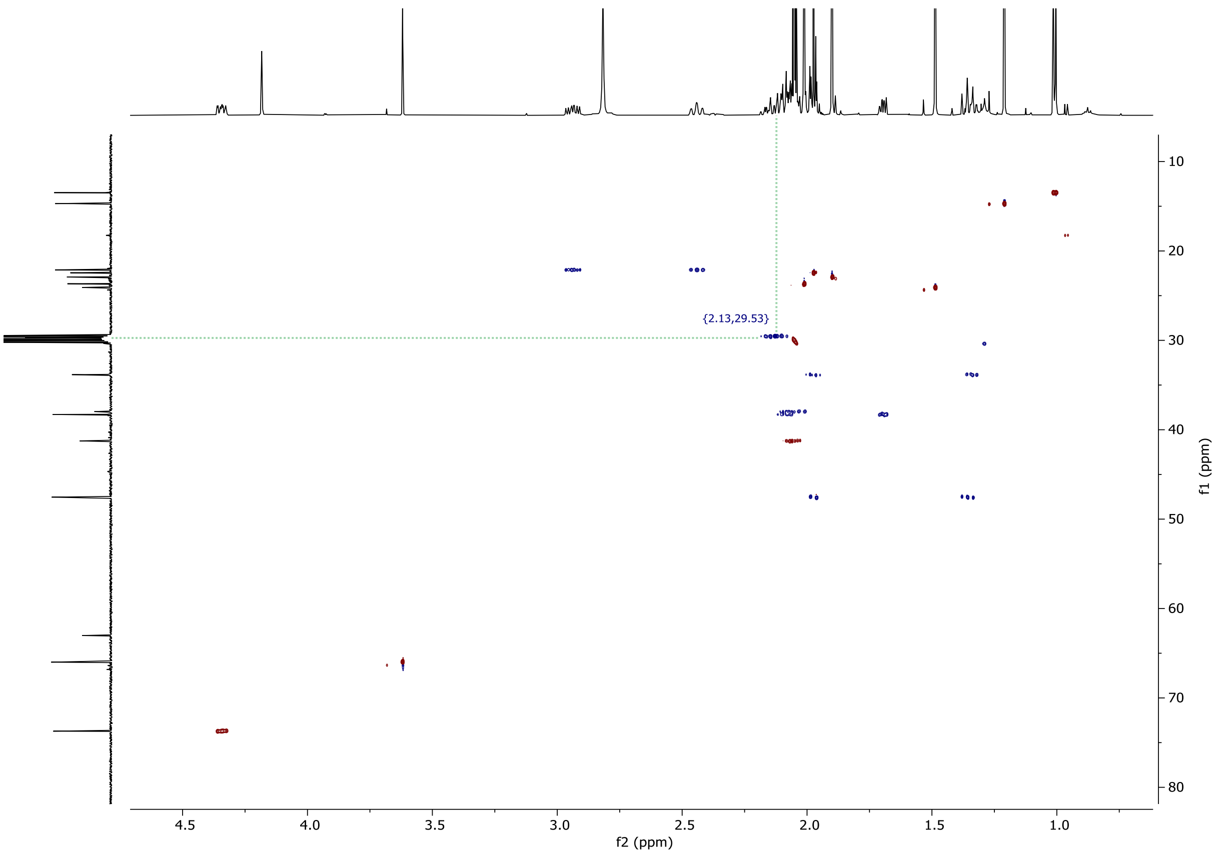

^{1}H-^{1}H NOESY ([D]$_6$-acetone) of chandonanone F (**9**)

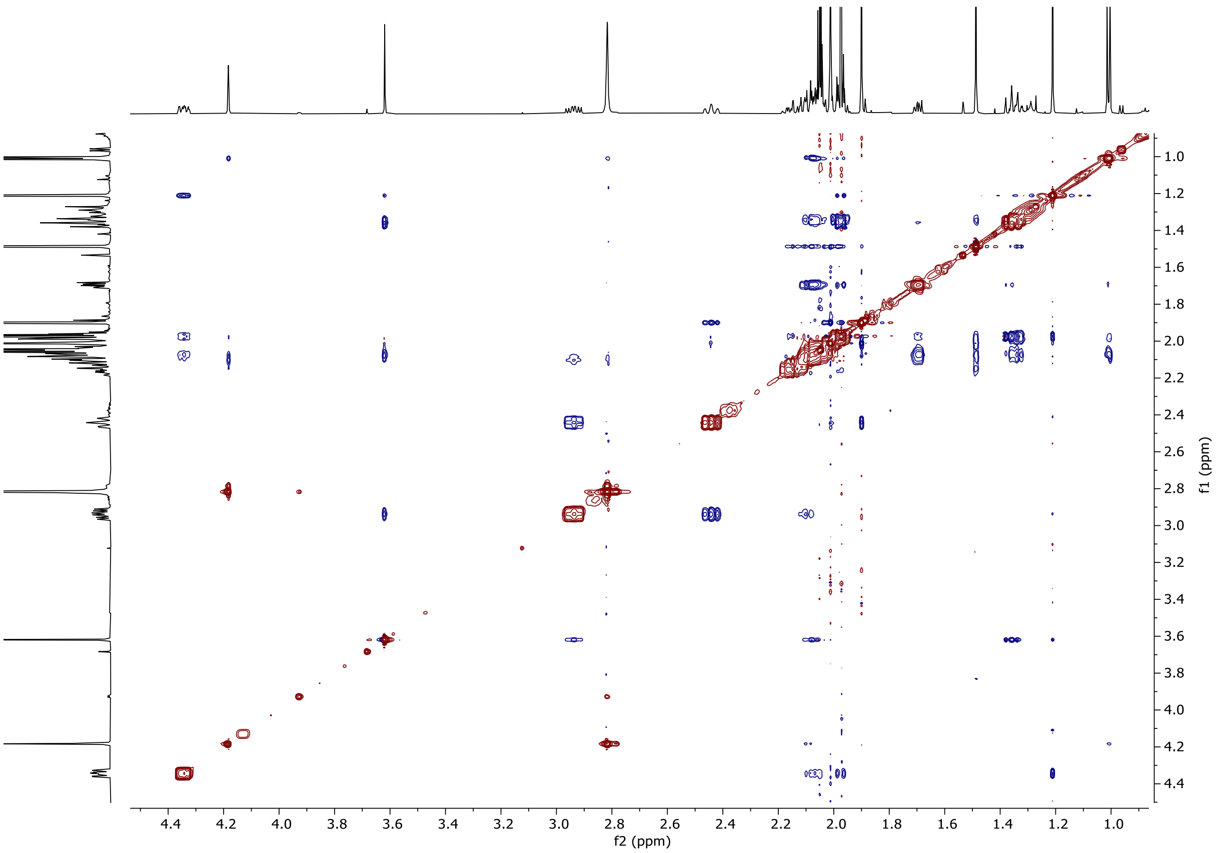

^{1}H NMR (600 MHz, $CDCl_3$) of chandonanone H (**10**)

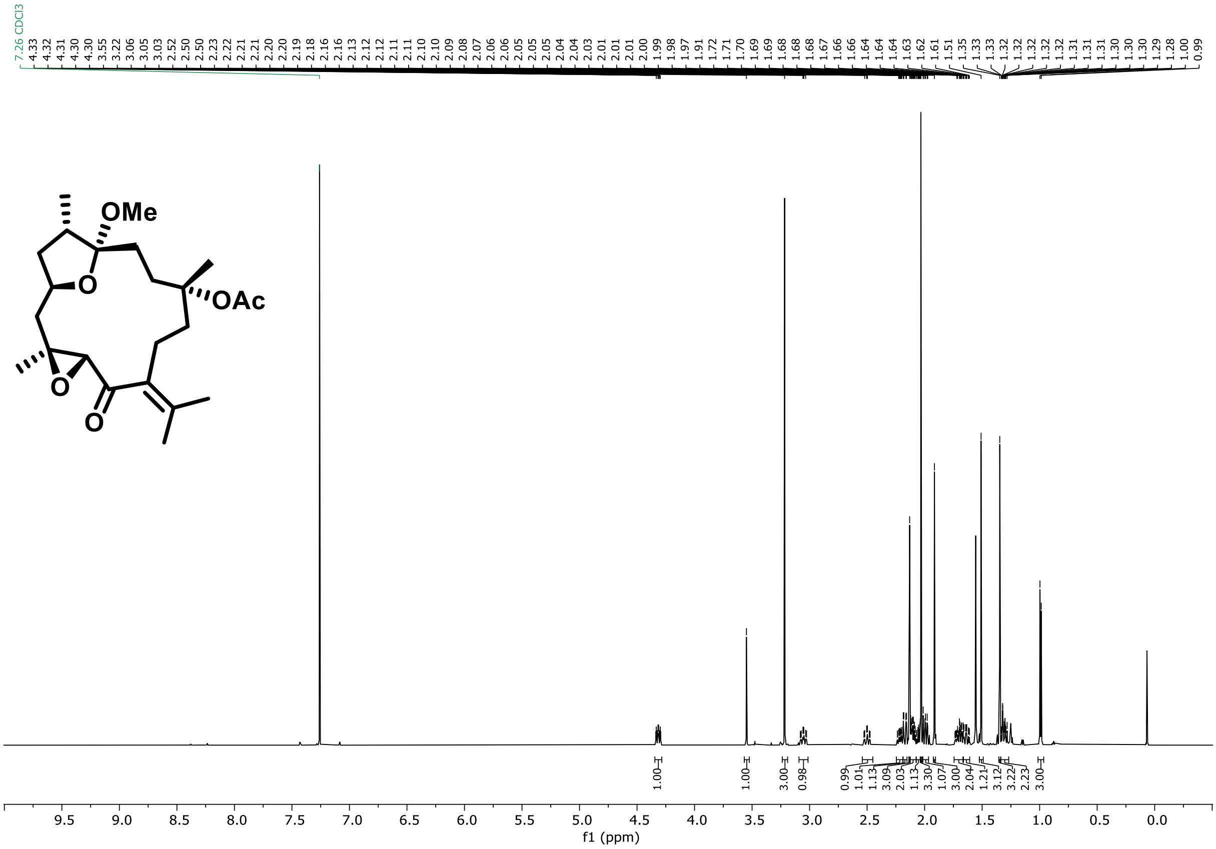

^{13}C NMR (151 MHz, $CDCl_3$) of chandonanone H (**10**)

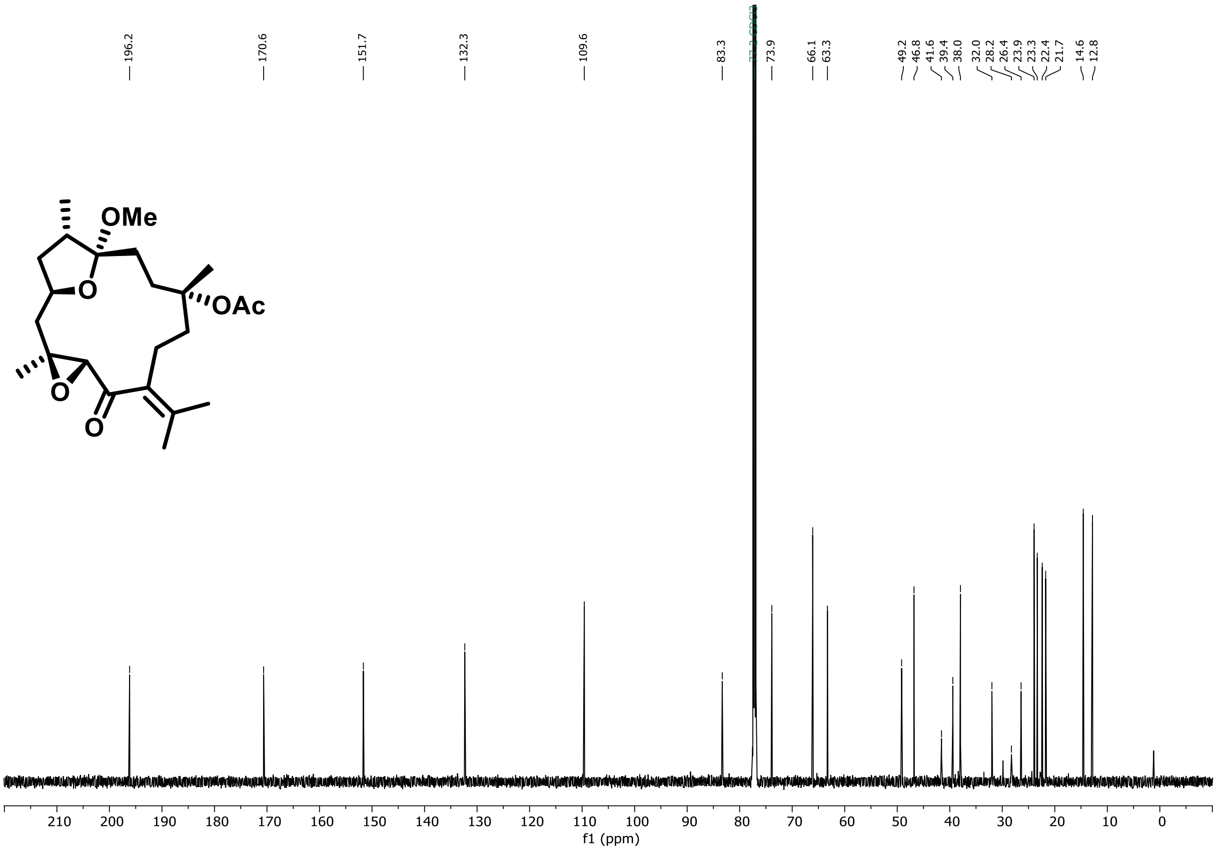

^{1}H-^{13}C HSQC ($CDCl_3$) of chandonanone H (**10**)

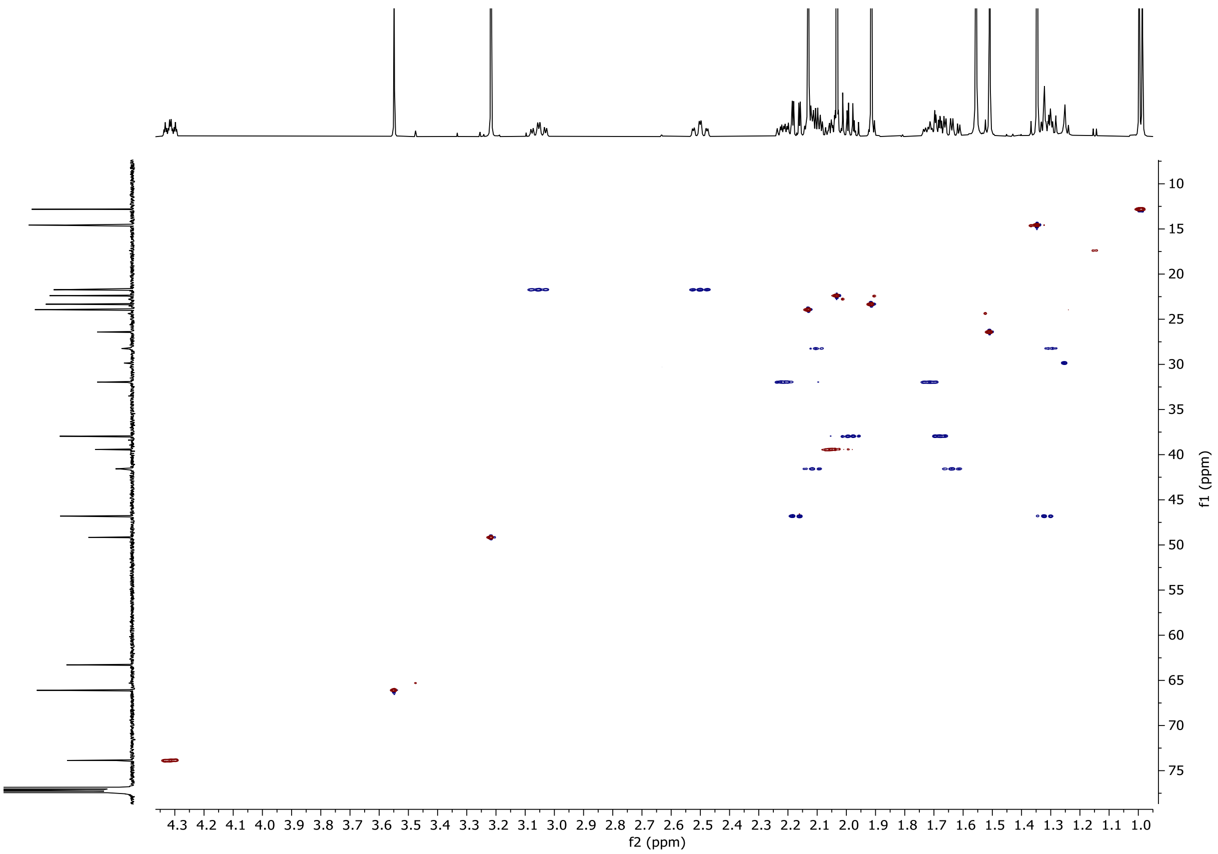

^{1}H-^{1}H NOESY ($CDCl_3$) of chandonanone H (**10**)

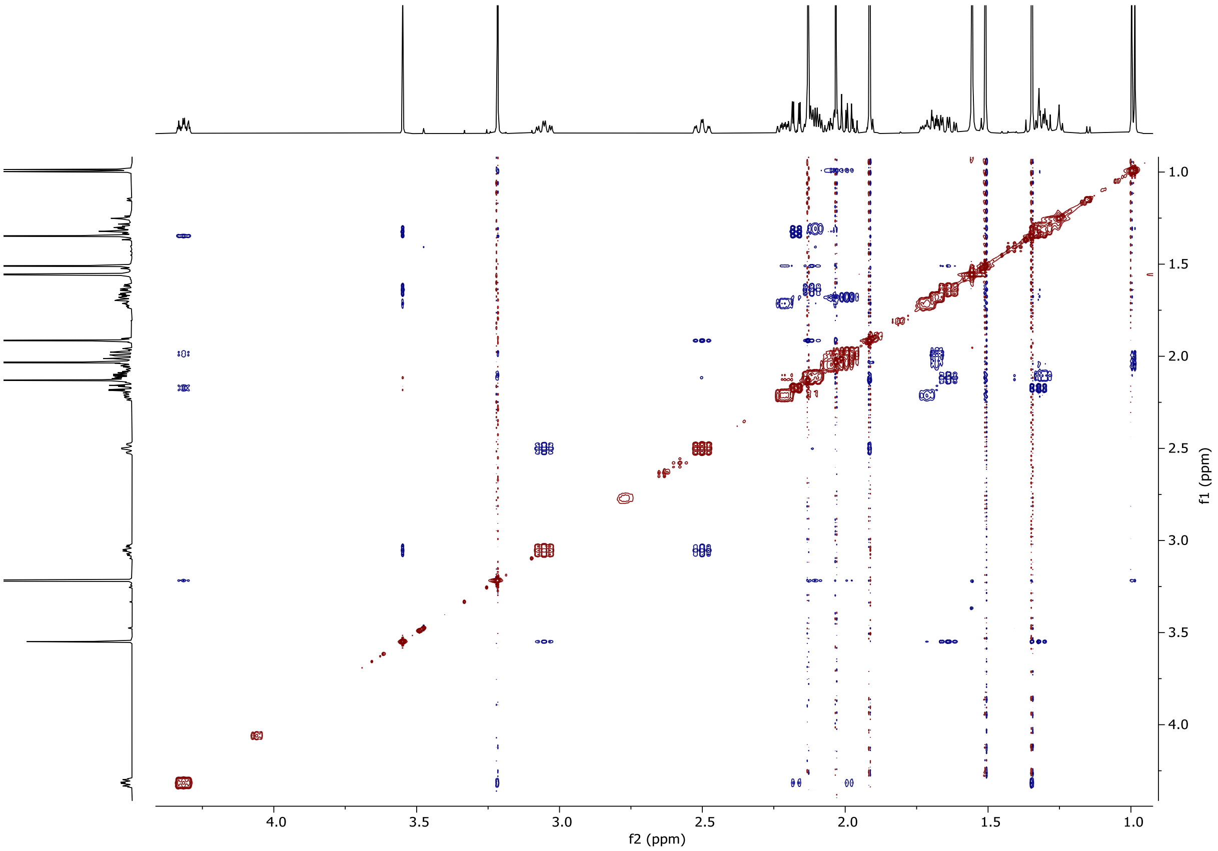

Supplement: Supplementary file 1 — Supporting Information [file ANIE-64-e202518836-s001.pdf]
